# Supplementary material for: Xianling Gubao attenuates high glucose-induced bone metabolism disorder in MG63 osteoblast-like cells
Source: PLoS One. 2022 Dec 22;17(12):e0276328. doi: 10.1371/journal.pone.0276328 (PMC9778583; doi:10.1371/journal.pone.0276328)

Fig2
A

450

OD值

	1	2	3	4	5	6	7	8	9	10	11	12
A	**1.042**	**1.164**	**1.135**	**1.382**	**0.428**	**0.498**	**0.405**	**0.446**	0.054	0.054	0.054	0.054
B	**1.087**	**1.135**	**1.241**	**1.212**	**0.289**	**0.246**	**0.205**	**0.223**	0.048	0.054	0.054	0.054
C	**1.189**	**1.063**	**1.097**	**1.104**	**0.149**	**0.173**	**0.156**	**0.148**	0.042	0.042	0.042	0.042
D	**1.157**	**1.121**	**1.109**	**1.054**	0.042	0.042	0.042	0.042	0.042	0.042	0.042	0.042
E	**0.993**	**1.054**	**0.975**	**1.012**	0.048	0.048	0.048	0.048	0.048	0.048	0.048	0.048
F	**0.998**	**1.024**	**0.953**	**0.949**	0.058	0.058	0.058	0.058	0.058	0.058	0.058	0.058
G	**0.822**	**0.737**	**0.806**	**0.801**	0.047	0.047	0.047	0.047	0.047	0.047	0.047	0.047
H	**0.678**	**0.625**	**0.668**	**0.729**	0.046	0.046	0.046	0.046	0.046	0.046	0.046	0.046

计算结果

	1	2	3	4	5	6	7	8	9	10	11	12
A	1.042	1.164	1.135	1.382	0.428	0.498	0.405	0.446	0.054	0.054	0.054	0.054
B	1.087	1.135	1.241	1.212	0.289	0.246	0.205	0.223	0.048	0.054	0.054	0.054
C	1.189	1.063	1.097	1.104	0.149	0.173	0.156	0.148	0.042	0.042	0.042	0.042
D	1.157	1.121	1.109	1.054	0.042	0.042	0.042	0.042	0.042	0.042	0.042	0.042
E	0.993	1.054	0.975	1.012	0.048	0.048	0.048	0.048	0.048	0.048	0.048	0.048
F	0.998	1.024	0.953	0.949	0.058	0.058	0.058	0.058	0.058	0.058	0.058	0.058
G	0.822	0.737	0.806	0.801	0.047	0.047	0.047	0.047	0.047	0.047	0.047	0.047
H	0.678	0.625	0.668	0.729	0.046	0.046	0.046	0.046	0.046	0.046	0.046	0.046

定性判定

	1	2	3	4	5	6	7	8	9	10	11	12
A	+	+	+	+	+	+	+	+	−	−	−	−
B	+	+	+	+	+	+	+	+	−	−	−	−
C	+	+	+	+	+	+	+	+	−	−	−	−
D	+	+	+	+	−	−	−	−	−	−	−	−
E	+	+	+	+	−	−	−	−	−	−	−	−
F	+	+	+	+	−	−	−	−	−	−	−	−
G	+	+	+	+	−	−	−	−	−	−	−	−
H	+	+	+	+	−	−	−	−	−	−	−	−

阴性均值：0.05(位置：H11,H10)[计算：Max(NC,0.05)]
阳性均值：0.046(位置：H7,H9,H8)[计算：Avrg(PC)]
空白均值：0.046(位置：H12)[计算：Avrg(B)]
Cutoff值：0.105(公式：N*2.1)
阴性判定：<0.105
阳性判定：>=0.105

整板变异：CV(OD)：114.42% 均值:0.396 SD:0.453

mM					
0	1.042	1.164	1.135	1.382	**1.18075**
2.5	1.087	1.135	1.241	1.212	**1.16875**
4	1.189	1.063	1.097	1.104	**1.11325**
5	1.157	1.121	1.109	1.054	**1.11025**
7.5	0.993	1.054	0.975	1.012	**1.0085**
10	0.998	1.024	0.953	0.949	**0.981**
15	0.822	0.737	0.806	0.801	**0.7915**
20	0.678	0.625	0.668	0.729	**0.675**
30	0.428	0.498	0.405	0.446	**0.44425**
40	0.289	0.246	0.205	0.223	**0.24075**
50	0.149	0.173	0.156	0.148	**0.1565**

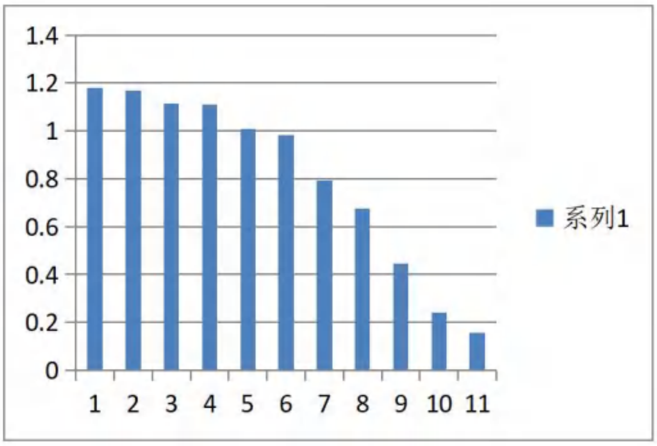

2B

450

OD值

	1	2	3	4	5	6	7	8	9	10	11	12
A	**0.873**	**0.949**	**0.836**	**0.78**	**0.629**	**0.731**	0.048	0.044	0.045	0.043	0.043	0.045
B	**0.663**	**0.836**	**0.682**	**0.7**	**0.7**	**0.765**	0.046	0.048	0.046	0.049	0.044	0.054
C	**0.446**	**0.594**	**0.42**	**0.474**	**0.442**	**0.572**	0.045	0.049	0.046	0.048	0.045	0.044
D	**0.484**	**0.566**	**0.451**	**0.171**	**0.18**	**0.182**	0.047	0.05	0.046	0.046	0.045	0.049
E	**0.609**	**0.711**	**0.613**	**0.158**	**0.165**	**0.164**	0.048	0.052	0.047	0.047	0.044	0.045
F	**0.568**	**0.71**	**0.654**	**0.163**	**0.164**	**0.165**	*0.951*	0.053	0.05	0.047	0.048	0.052
G	**0.655**	**0.673**	**0.669**	**0.168**	**0.169**	**0.172**	*0.871*	0.052	0.049	0.047	0.046	0.042
H	**0.57**	**0.72**	**0.572**	**0.182**	**0.181**	**0.18**	*0.708*	0.043	0.046	0.044	0.045	0.041

计算结果

	1	2	3	4	5	6	7	8	9	10	11	12
A	0.873	0.949	0.836	0.78	0.629	0.731	0.048	0.044	0.045	0.043	0.043	0.045
B	0.663	0.836	0.682	0.7	0.7	0.765	0.046	0.048	0.046	0.049	0.044	0.054
C	0.446	0.594	0.42	0.474	0.442	0.572	0.045	0.049	0.046	0.048	0.045	0.044
D	0.484	0.566	0.451	0.171	0.18	0.182	0.047	0.05	0.046	0.046	0.045	0.049
E	0.609	0.711	0.613	0.158	0.165	0.164	0.048	0.052	0.047	0.047	0.044	0.045
F	0.568	0.71	0.654	0.163	0.164	0.165	0.951	0.053	0.05	0.047	0.048	0.052
G	0.655	0.673	0.669	0.168	0.169	0.172	0.871	0.052	0.049	0.047	0.046	0.042
H	0.57	0.72	0.572	0.182	0.181	0.18	0.708	0.043	0.046	0.044	0.045	0.041

定性判定

	1	2	3	4	5	6	7	8	9	10	11	12
A	+	+	+	+	+	+	-	-	-	-	-	-
B	+	+	+	+	+	+	-	-	-	-	-	-
C	+	+	+	+	+	+	-	-	-	-	-	-
D	+	+	+	+	+	+	-	-	-	-	-	-
E	+	+	+	+	+	+	-	-	-	-	-	-
F	+	+	+	+	+	+	+	-	-	-	-	-
G	+	+	+	+	+	+	+	-	-	-	-	-
H	+	+	+	+	+	+	+	-	-	-	-	-

2018/5/16 11:03:58

单波长测量：F2(450)
阴性均值：0.05(位置：H11,H10)[计算：Max(NC,0.05)]
阳性均值：0.266(位置：H7,H9,H8)[计算：Avrg(PC)]
空白均值：0.041(位置：H12)[计算：Avrg(B)]
Cutoff值：0.105(公式：N*2.1)
阴性判定：<0.105
阳性判定：>=0.105

整板变异：CV(OD)：100.95% 均值:0.297 SD:0.3

母液浓度 10mg/ml (0.01mg/l)

	体积				OD	mg/l
1	正 常	0.873	0.949	0.836	0.886	
2	甘露糖	0.663	0.836	0.682	0.727	
3	0	0.446	0.594	0.42	0.486667	
	0	0.484	0.566	0.451	0.500333	0
4	2.5	0.609	0.711	0.613	0.644333	25
5	5	0.568	0.71	0.654	0.644	50
6	7.5	0.655	0.673	0.669	0.665667	75
7	10	0.57	0.72	0.572	0.620667	100
8	15	0.78	0.629	0.731	0.713333	150
9	20	0.7	0.7	0.765	0.721667	200
10	40	0.474	0.442	0.572	0.496	400
11	60	0.171	0.18	0.182	0.177667	600
12	80	0.158	0.165	0.164	0.162333	800
13	100	0.163	0.164	0.165	0.164	1000
14	150	0.168	0.169	0.172	0.169667	1500
15	200	0.182	0.181	0.18	0.181	2000

200uM高糖&200mg/l仙灵骨堡

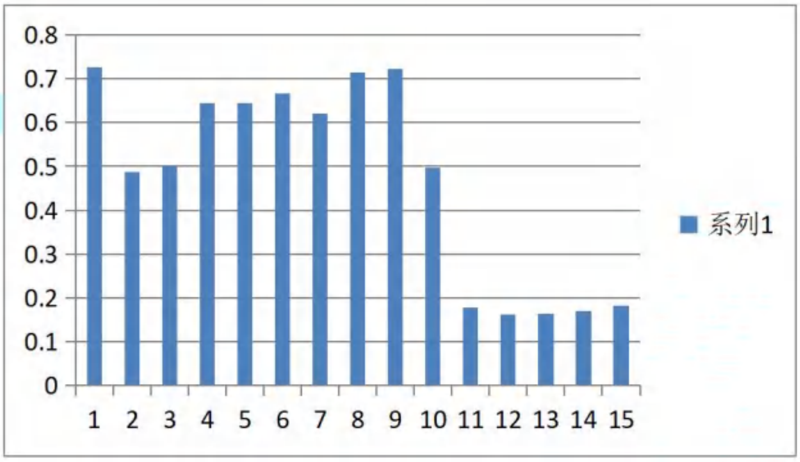

0.663	0.836	0.682
0.446	0.594	0.42
0.609	0.711	0.613
0.568	0.71	0.654
0.655	0.673	0.669
0.57	0.72	0.572
0.78	0.629	0.731
0.7	0.7	0.765
0.474	0.442	0.572
0.171	0.18	0.182
0.158	0.165	0.164
0.163	0.164	0.165
0.168	0.169	0.172
0.182	0.181	0.18

CCK8

450

OD值

	1	2	3	4	5	6	7	8	9	10	11	12
A	**0.264**	**0.279**	**0.271**	**0.275**	**0.242**	**0.269**	**0.267**	**0.262**	**0.248**	0.042	0.044	0.045
B	0.002	0.001	0.001	0.002	0.001	0.002	0.001	0.002	0.002	0.001	0.001	0.001
C	0.001	0	0.001	0.002	0	0.001	0	0.002	0.001	0	0.001	-0.001
D	0	0	0	0.001	0	0.001	-0.001	0.001	0	0	0	-0.001
E	0.001	0.001	0.001	0.001	-0.001	0.001	0	0.001	0.001	0	0	0
F	0.002	0.001	0.002	0.002	0	0.002	0.001	0.001	0.002	0	0.001	0
G	0.001	0.001	0.002	0.002	0.001	0.002	0	0.002	0.001	0.001	0.001	0
H	0.001	0.001	0.001	0.001	0	0.001	0	0.002	0.001	0	0	0

计算结果

	1	2	3	4	5	6	7	8	9	10	11	12
A	0.264	0.279	0.271	0.275	0.242	0.269	0.267	0.262	0.248	0.042	0.044	0.045
B	0.002	0.001	0.001	0.002	0.001	0.002	0.001	0.002	0.002	0.001	0.001	0.001
C	0.001	0	0.001	0.002	0	0.001	0	0.002	0.001	0	0.001	-0.001
D	0	0	0	0.001	0	0.001	-0.001	0.001	0	0	0	-0.001
E	0.001	0.001	0.001	0.001	-0.001	0.001	0	0.001	0.001	0	0	0
F	0.002	0.001	0.002	0.002	0	0.002	0.001	0.001	0.002	0	0.001	0
G	0.001	0.001	0.002	0.002	0.001	0.002	0	0.002	0.001	0.001	0.001	0
H	0.001	0.001	0.001	0.001	0	0.001	0	0.002	0.001	0	0	0

定性判定

	1	2	3	4	5	6	7	8	9	10	11	12
A	+	+	+	+	+	+	+	+	+	-	-	-
B	-	-	-	-	-	-	-	-	-	-	-	-
C	-	-	-	-	-	-	-	-	-	-	-	-
D	-	-	-	-	-	-	-	-	-	-	-	-
E	-	-	-	-	-	-	-	-	-	-	-	-
F	-	-	-	-	-	-	-	-	-	-	-	-
G	-	-	-	-	-	-	-	-	-	-	-	-
H	-	-	-	-	-	-	-	-	-	-	-	-

2018/5/17 13:52

单波长测量：F2(450)
阴性均值：0.05(位置：H11,H10)[计算：Max(NC,0.05)]
阳性均值：0.001(位置：H7,H9,H8)[计算：Avrg(PC)]
空白均值：0(位置：H12)[计算：Avrg(B)]
Cutoff值：0.105(公式：N*2.1)
阴性判定：<0.105
阳性判定：>=0.105

整板变异：CV(OD)：287.57% 均值:0.027 SD:0.077

450

OD值

	1	2	3	4	5	6	7	8	9	10	11	12
A	**0.495**	**0.523**	**0.535**	**0.4**	**0.434**	**0.422**	**0.525**	**0.545**	**0.525**	0.043	0.042	0.043
B	-0.001	0	0	-0.002	-0.002	0	-0.001	0	-0.001	0.001	-0.001	0.001
C	-0.001	0	0	-0.001	-0.002	0	-0.001	0	-0.002	0.001	-0.001	0.001
D	-0.001	0	0	-0.001	-0.001	0	-0.001	-0.001	0	0.001	-0.001	0.001
E	-0.001	0	0.001	0	0	0	0	0	0	0	-0.001	0.001
F	0	0.001	0	0	-0.001	0	0	0	0	0	-0.001	0.001
G	-0.001	0	0	-0.001	-0.001	0	-0.001	-0.001	-0.001	0	-0.001	0
H	0	0	0	-0.002	-0.001	-0.001	-0.001	-0.001	-0.001	0	-0.001	0

计算结果

	1	2	3	4	5	6	7	8	9	10	11	12
A	0.495	0.523	0.535	0.4	0.434	0.422	0.525	0.545	0.525	0.043	0.042	0.043
B	-0.001	0	0	-0.002	-0.002	0	-0.001	0	-0.001	0.001	-0.001	0.001
C	-0.001	0	0	-0.001	-0.002	0	-0.001	0	-0.002	0.001	-0.001	0.001
D	-0.001	0	0	-0.001	-0.001	0	-0.001	-0.001	0	0.001	-0.001	0.001
E	-0.001	0	0.001	0	0	0	0	0	0	0	-0.001	0.001
F	0	0.001	0	0	-0.001	0	0	0	0	0	-0.001	0.001
G	-0.001	0	0	-0.001	-0.001	0	-0.001	-0.001	-0.001	0	-0.001	0
H	0	0	0	-0.002	-0.001	-0.001	-0.001	-0.001	-0.001	0	-0.001	0

定性判定

	1	2	3	4	5	6	7	8	9	10	11	12
A	+	+	+	+	+	+	+	+	+	-	-	-
B	-	-	-	-	-	-	-	-	-	-	-	-
C	-	-	-	-	-	-	-	-	-	-	-	-
D	-	-	-	-	-	-	-	-	-	-	-	-
E	-	-	-	-	-	-	-	-	-	-	-	-
F	-	-	-	-	-	-	-	-	-	-	-	-
G	-	-	-	-	-	-	-	-	-	-	-	-
H	-	-	-	-	-	-	-	-	-	-	-	-

2018/5/18 12:02:07

单波长测量：F2(450)

阴性均值：0.05(位置：H11,H10)[计算：Max(NC,0.05)]
阳性均值：-0.001(位置：H7,H9,H8)[计算：Avrg(PC)]
空白均值：0(位置：H12)[计算：Avrg(B)]
Cutoff值：0.105(公式：N*2.1)
阴性判定：<0.105
阳性判定：>=0.105

整板变异：CV(OD)：307.45% 均值:0.047 SD:0.144

450

OD值

	1	2	3	4	5	6	7	8	9	10	11	12
A	**0.691**	**0.735**	**0.76**	**0.597**	**0.634**	**0.655**	**0.741**	**0.798**	**0.724**	0.042	0.043	0.04
B	-0.001	-0.001	-0.001	0	-0.001	-0.001	-0.001	-0.002	0	-0.001	-0.001	-0.002
C	0	-0.001	0	0.001	-0.001	-0.001	0	-0.002	0	-0.001	-0.001	-0.001
D	0	-0.001	0	0.001	-0.001	-0.001	-0.001	-0.001	0	-0.002	-0.001	-0.001
E	0	-0.001	0	0	-0.001	-0.001	-0.001	-0.001	0	-0.002	-0.001	-0.001
F	-0.001	-0.003	0	0	-0.001	-0.002	-0.001	-0.002	0	-0.003	-0.002	-0.003
G	-0.001	-0.002	0	0	-0.001	-0.002	-0.001	-0.002	0	-0.003	-0.001	-0.003
H	-0.001	-0.002	0	0	-0.001	-0.002	-0.002	-0.002	-0.001	-0.002	-0.001	-0.002

计算结果

	1	2	3	4	5	6	7	8	9	10	11	12
A	0.691	0.735	0.76	0.597	0.634	0.655	0.741	0.798	0.724	0.042	0.043	0.04
B	-0.001	-0.001	-0.001	0	-0.001	-0.001	-0.001	-0.002	0	-0.001	-0.001	-0.002
C	0	-0.001	0	0.001	-0.001	-0.001	0	-0.002	0	-0.001	-0.001	-0.001
D	0	-0.001	0	0.001	-0.001	-0.001	-0.001	-0.001	0	-0.002	-0.001	-0.001
E	0	-0.001	0	0	-0.001	-0.001	-0.001	-0.001	0	-0.002	-0.001	-0.001
F	-0.001	-0.003	0	0	-0.001	-0.002	-0.001	-0.002	0	-0.003	-0.002	-0.003
G	-0.001	-0.002	0	0	-0.001	-0.002	-0.001	-0.002	0	-0.003	-0.001	-0.003
H	-0.001	-0.002	0	0	-0.001	-0.002	-0.002	-0.002	-0.001	-0.002	-0.001	-0.002

定性判定

	1	2	3	4	5	6	7	8	9	10	11	12
A	+	+	+	+	+	+	+	+	+	-	-	-
B	-	-	-	-	-	-	-	-	-	-	-	-
C	-	-	-	-	-	-	-	-	-	-	-	-
D	-	-	-	-	-	-	-	-	-	-	-	-
E	-	-	-	-	-	-	-	-	-	-	-	-
F	-	-	-	-	-	-	-	-	-	-	-	-
G	-	-	-	-	-	-	-	-	-	-	-	-
H	-	-	-	-	-	-	-	-	-	-	-	-

2018/5/19 18:39:33

单波长测量：F2(450)
阴性均值：0.05(位置：H11,H10)[计算：Max(NC,0.05)]
阳性均值：-0.002(位置：H7,H9,H8)[计算：Avrg(PC)]
空白均值：-0.002(位置：H12)[计算：Avrg(B)]
Cutoff值：0.105(公式：N*2.1)
阴性判定：<0.105
阳性判定：>=0.105

整板变异：CV(OD)：311.93% 均值:0.066 SD:0.207

450

OD值

	1	2	3	4	5	6	7	8	9	10	11	12
A	0.04	0.041	0.038	0.04	0.041	0.04	0.039	0.04	0.039	0.038	0.038	0.039
B	0.045	0.046	0.045	0.047	0.047	0.047	0.045	0.046	0.048	0.048	0.038	0.041
C	0.047	0.054	0.047	0.054	0.048	0.046	0.048	0.051	0.053	0.053	0.04	0.046
D	0.042	0.044	0.04	0.042	0.043	0.041	0.039	0.042	0.041	0.043	0.039	0.04
E	0.043	**1.431**	**1.449**	**1.528**	**1.056**	**1.186**	**1.077**	**1.346**	**1.312**	**1.397**	0.04	0.043
F	0.043	0.038	0.042	0.043	0.044	0.044	0.042	0.043	0.042	0.043	0.042	0.043
G	0.042	0.038	0.038	0.039	0.041	0.04	0.04	0.041	0.038	0.041	0.04	0.042
H	0.039	0.039	0.037	0.038	0.04	0.038	0.037	0.041	0.038	0.037	0.037	0.038

计算结果

	1	2	3	4	5	6	7	8	9	10	11	12
A	0.04	0.041	0.038	0.04	0.041	0.04	0.039	0.04	0.039	0.038	0.038	0.039
B	0.045	0.046	0.045	0.047	0.047	0.047	0.045	0.046	0.048	0.048	0.038	0.041
C	0.047	0.054	0.047	0.054	0.048	0.046	0.048	0.051	0.053	0.053	0.04	0.046
D	0.042	0.044	0.04	0.042	0.043	0.041	0.039	0.042	0.041	0.043	0.039	0.04
E	0.043	1.431	1.449	1.528	1.056	1.186	1.077	1.446	1.412	1.497	0.04	0.043
F	0.043	0.038	0.042	0.043	0.044	0.044	0.042	0.043	0.042	0.043	0.042	0.043
G	0.042	0.038	0.038	0.039	0.041	0.04	0.04	0.041	0.038	0.041	0.04	0.042
H	0.039	0.039	0.037	0.038	0.04	0.038	0.037	0.041	0.038	0.037	0.037	0.038

定性判定

	1	2	3	4	5	6	7	8	9	10	11	12
A	-	-	-	-	-	-	-	-	-	-	-	-
B	-	-	-	-	-	-	-	-	-	-	-	-
C	-	-	-	-	-	-	-	-	-	-	-	-
D	-	-	-	-	-	-	-	-	-	-	-	-
E	-	+	+	+	+	+	+	+	+	+	-	-
F	-	-	-	-	-	-	-	-	-	-	-	-
G	-	-	-	-	-	-	-	-	-	-	-	-
H	-	-	-	-	-	-	-	-	-	-	-	-

2018/5/20 16:41

单波长测量：F2(450)
阴性均值：0.05(位置：H11,H10)[计算：Max(NC,0.05)]
阳性均值：0.039(位置：H7,H9,H8)[计算：Avrg(PC)]
空白均值：0.038(位置：H12)[计算：Avrg(B)]
Cutoff值：0.105(公式：N*2.1)
阴性判定：<0.105
阳性判定：>=0.105

整板变异：CV(OD)：234.42% 均值:0.164 SD:0.385

450

OD值

	1	2	3	4	5	6	7	8	9	10	11	12
A	**0.264**	**0.279**	**0.271**	**0.275**	**0.242**	**0.269**	**0.267**	**0.262**	**0.248**	0.042	0.044	0.045
B	0.002	0.001	0.001	0.002	0.001	0.002	0.001	0.002	0.002	0.001	0.001	0.001
C	0.001	0	0.001	0.002	0	0.001	0	0.002	0.001	0	0.001	-0.001
D	0	0	0	0.001	0	0.001	-0.001	0.001	0	0	0	-0.001
E	0.001	0.001	0.001	0.001	-0.001	0.001	0	0.001	0.001	0	0	0
F	0.002	0.001	0.002	0.002	0	0.002	0.001	0.001	0.002	0	0.001	0
G	0.001	0.001	0.002	0.002	0.001	0.002	0	0.002	0.001	0.001	0.001	0
H	0.001	0.001	0.001	0.001	0	0.001	0	0.002	0.001	0	0	0

计算结果

	1	2	3	4	5	6	7	8	9	10	11	12
A	0.264	0.279	0.271	0.275	0.242	0.269	0.267	0.262	0.248	0.042	0.044	0.045
B	0.002	0.001	0.001	0.002	0.001	0.002	0.001	0.002	0.002	0.001	0.001	0.001
C	0.001	0	0.001	0.002	0	0.001	0	0.002	0.001	0	0.001	-0.001
D	0	0	0	0.001	0	0.001	-0.001	0.001	0	0	0	-0.001
E	0.001	0.001	0.001	0.001	-0.001	0.001	0	0.001	0.001	0	0	0
F	0.002	0.001	0.002	0.002	0	0.002	0.001	0.001	0.002	0	0.001	0
G	0.001	0.001	0.002	0.002	0.001	0.002	0	0.002	0.001	0.001	0.001	0
H	0.001	0.001	0.001	0.001	0	0.001	0	0.002	0.001	0	0	0

定性判定

	1	2	3	4	5	6	7	8	9	10	11	12
A	+	+	+	+	+	+	+	+	+	-	-	-
B	-	-	-	-	-	-	-	-	-	-	-	-
C	-	-	-	-	-	-	-	-	-	-	-	-
D	-	-	-	-	-	-	-	-	-	-	-	-
E	-	-	-	-	-	-	-	-	-	-	-	-
F	-	-	-	-	-	-	-	-	-	-	-	-
G	-	-	-	-	-	-	-	-	-	-	-	-
H	-	-	-	-	-	-	-	-	-	-	-	-

单波长测量：F2(450)
阴性均值：0.05(位置：H11,H10)[计算：Max(NC,0.05)]
阳性均值：0.001(位置：H7,H9,H8)[计算：Avrg(PC)]
空白均值：0(位置：H12)[计算：Avrg(B)]
Cutoff值：0.105(公式：N*2.1)
阴性判定：<0.105
阳性判定：>=0.105

整板变异：CV(OD)：287.57% 均值:0.027 SD:0.077

		NC				HG		
0h	0.264	0.279	0.271	0.271333	0.275	0.242	0.269	0.262
24h	0.495	0.523	0.535	0.517667	0.4	0.434	0.422	0.418667
48h	0.691	0.735	0.76	0.728667	0.597	0.634	0.655	0.628667
72h	1.431	1.449	1.528	1.469333	1.056	1.186	1.077	1.106333

	NC	HG	HG+仙
0h	0.271333	0.262	0.259
24h	0.517667	0.418667	0.531667
48h	0.728667	0.628667	0.754333
72h	1.469333	1.106333	1.351667

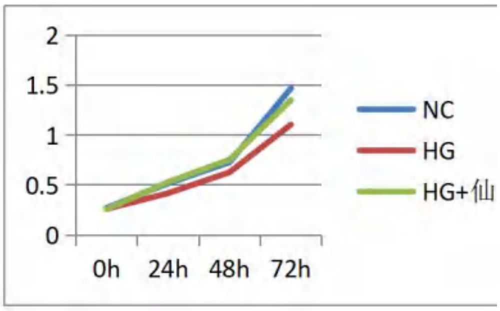

0.264	0.279	0.271	0.275	0.242	0.269	0.267	0.262
0.495	0.523	0.535	0.4	0.434	0.422	0.525	0.545
0.691	0.735	0.76	0.597	0.634	0.655	0.741	0.798
1.431	1.449	1.528	1.056	1.186	1.077	1.346	1.312

	HG+仙		
0.267	0.262	0.248	0.259
0.525	0.545	0.525	0.531667
0.741	0.798	0.724	0.754333
1.346	1.312	1.397	1.351667

0.248
0.525
0.724
1.397

	Control			Glucose			Glucose+XLGB	
0h	0.265	0.271	0.297	0.278	0.284	0.288	0.285	0.282
24h	0.534	0.521	0.509	0.468	0.485	0.487	0.511	0.487
48h	0.932	0.921	0.952	0.759	0.792	0.793	0.897	0.914
72h	1.403	1.385	1.375	1.047	1.102	1.048	1.287	1.293

0.279
0.532
0.899
1.294

	Control			Glucose			Glucose+XLG	
0h	0.305	0.314	0.315	0.321	0.311	0.318	0.308	0.316
24h	0.618	0.612	0.593	0.515	0.504	0.516	0.602	0.593
48h	0.955	0.931	0.918	0.708	0.724	0.737	0.887	0.914
72h	1.432	1.387	1.337	0.995	1.086	0.954	1.269	1.303

GB
0.322
0.553
0.901
1.282

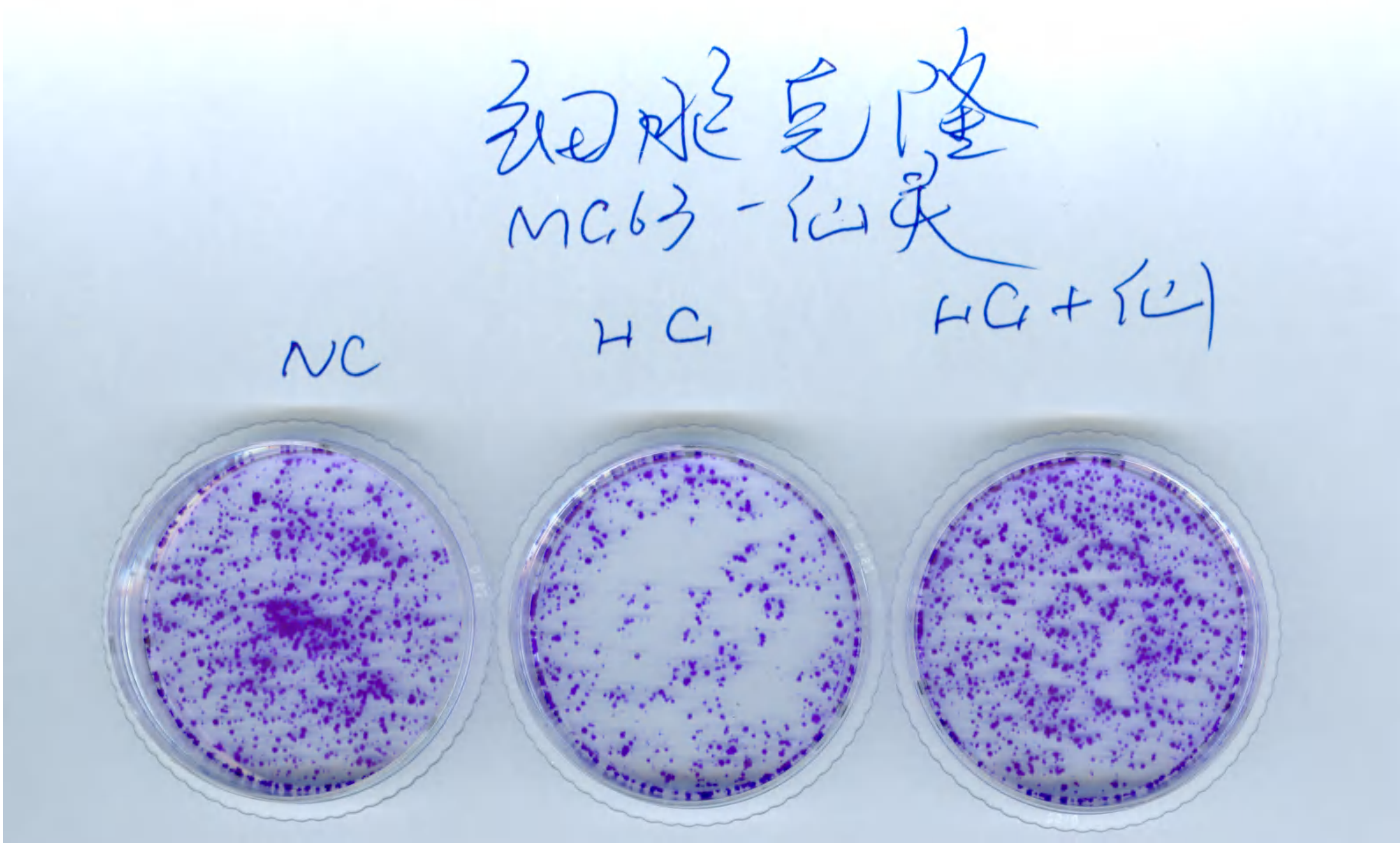
细胞克隆
MG63-仙灵
NC
HG
HG+仙

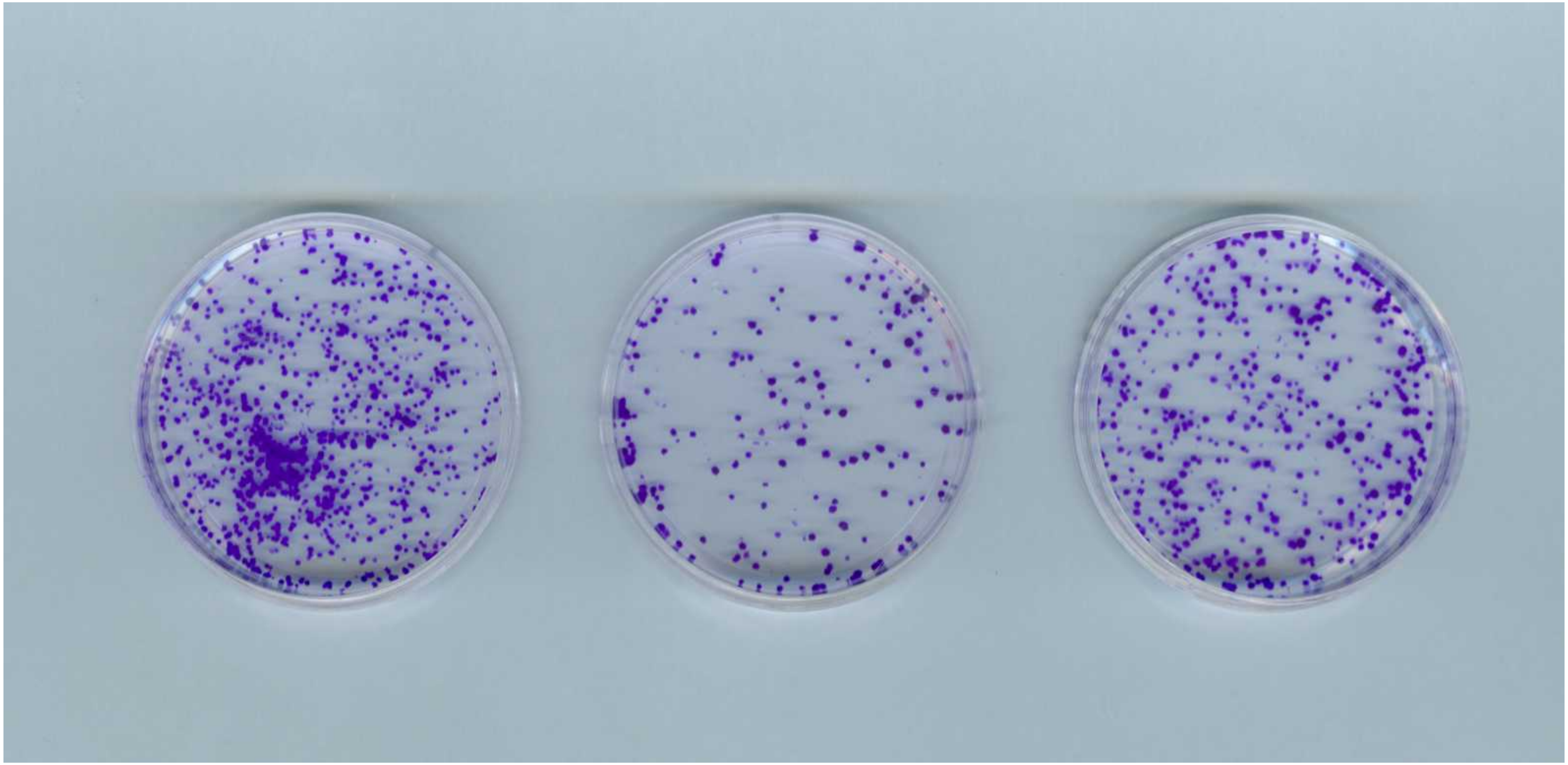

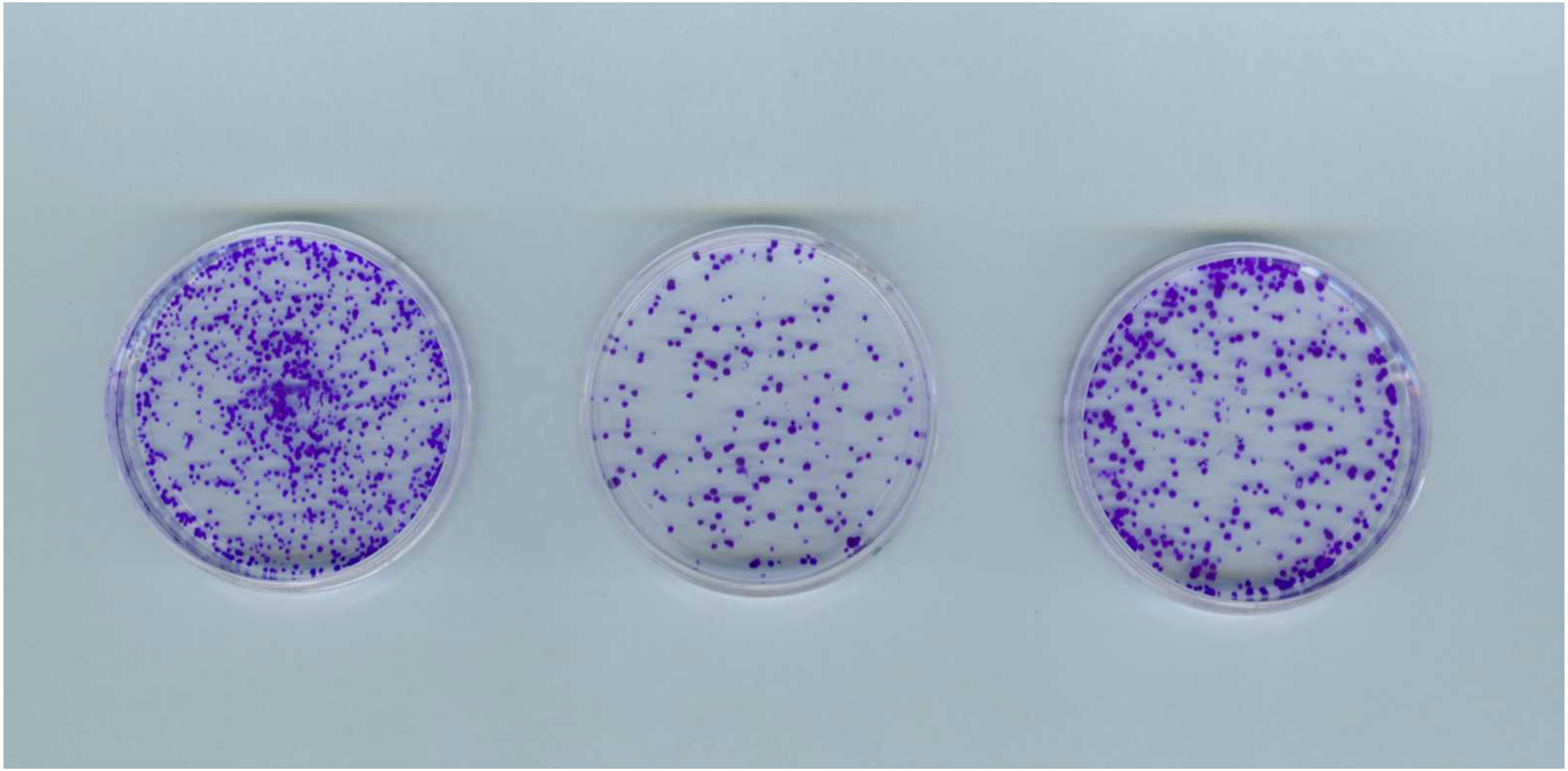

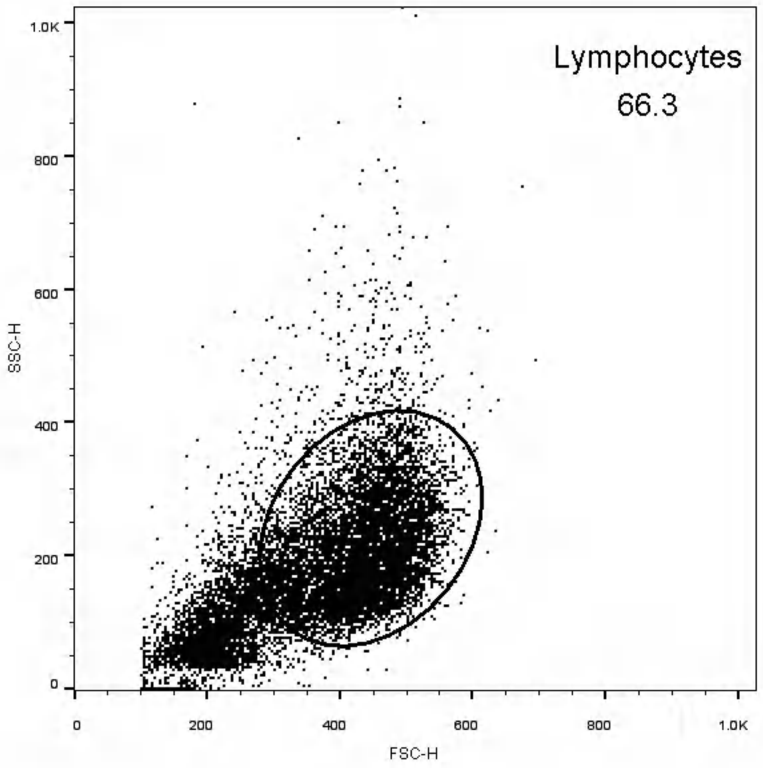

Lymphocytes
66.3
1.0K
800
600
400
200
0
SSC-H
0
200
400
600
800
1.0K
FSC-H

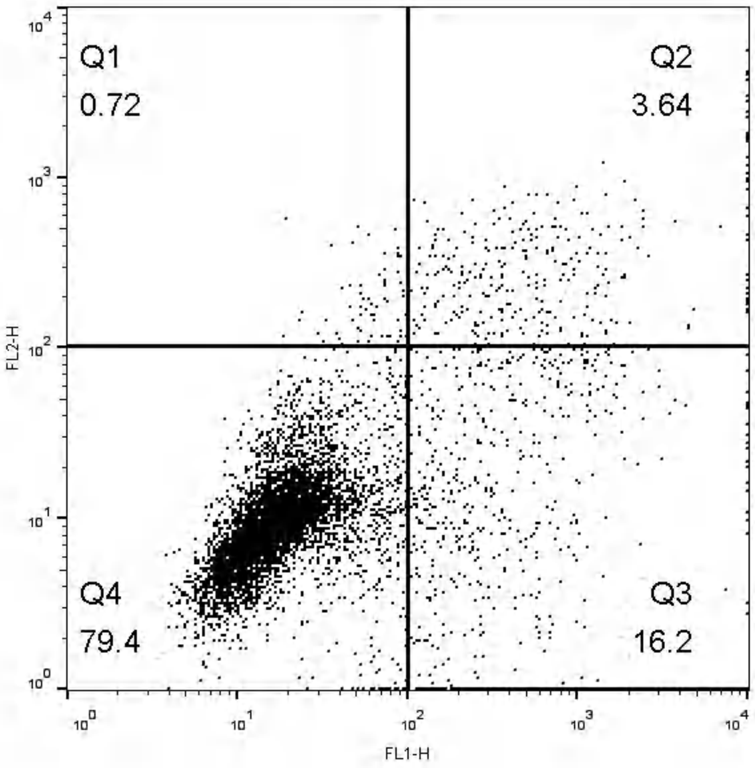

Q1
0.72
Q2
3.64
Q4
79.4
Q3
16.2
FL2-H
FL1-H

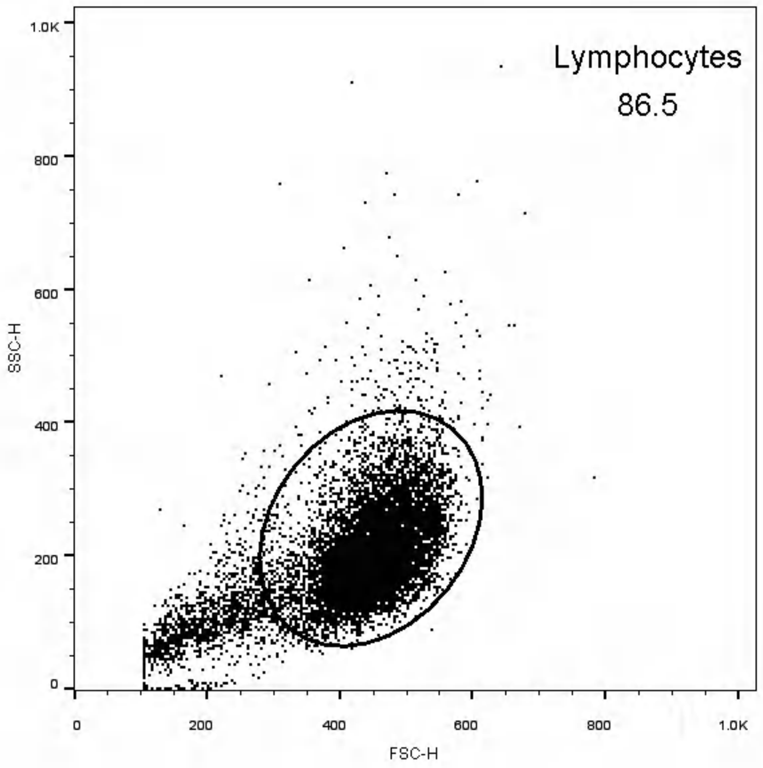

Lymphocytes
86.5
1.0K
800
600
400
200
0
SSC-H
0
200
400
600
800
1.0K
FSC-H

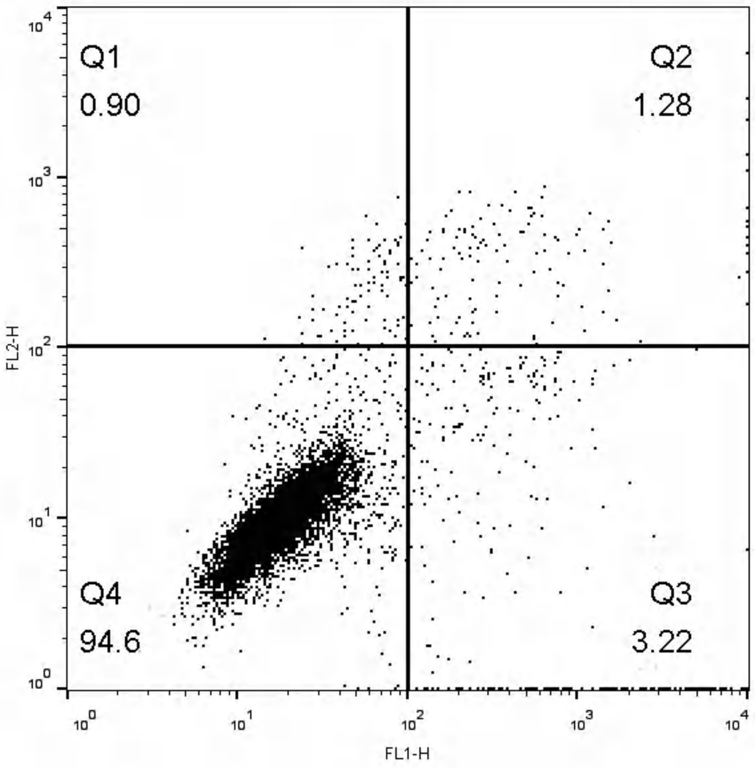

Q1
0.90
Q2
1.28
Q4
94.6
Q3
3.22
FL2-H
FL1-H

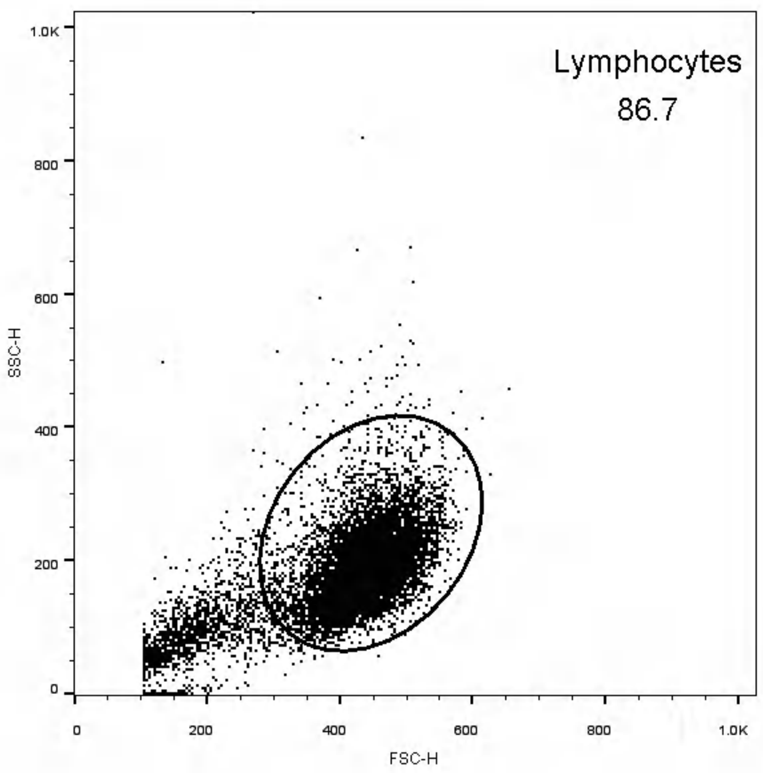
Lymphocytes
86.7
1.0K
800
600
400
200
0
SSC-H
0
200
400
600
800
1.0K
FSC-H

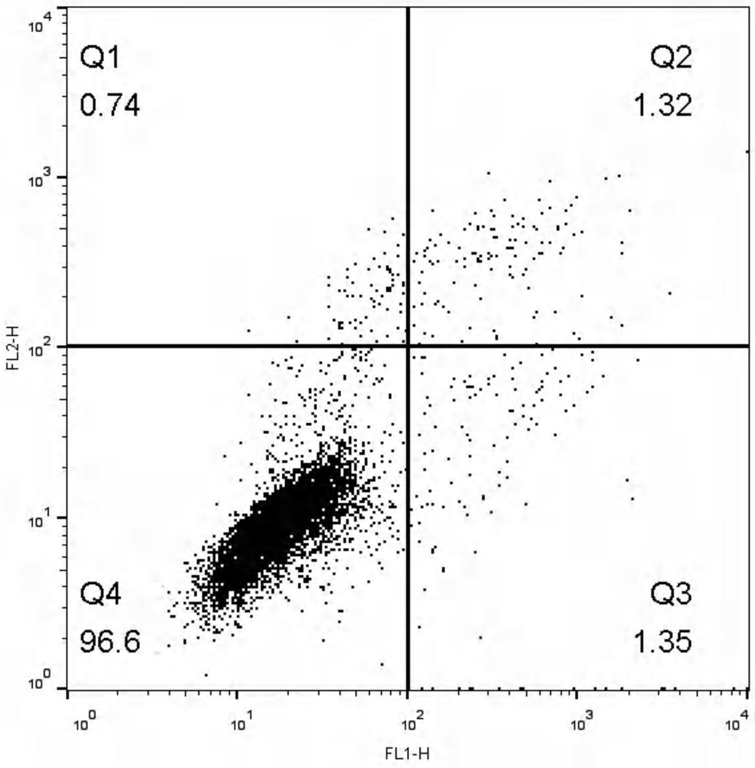
Q1
0.74
Q2
1.32
Q4
96.6
Q3
1.35
FL2-H
FL1-H
10^0
10^1
10^2
10^3
10^4

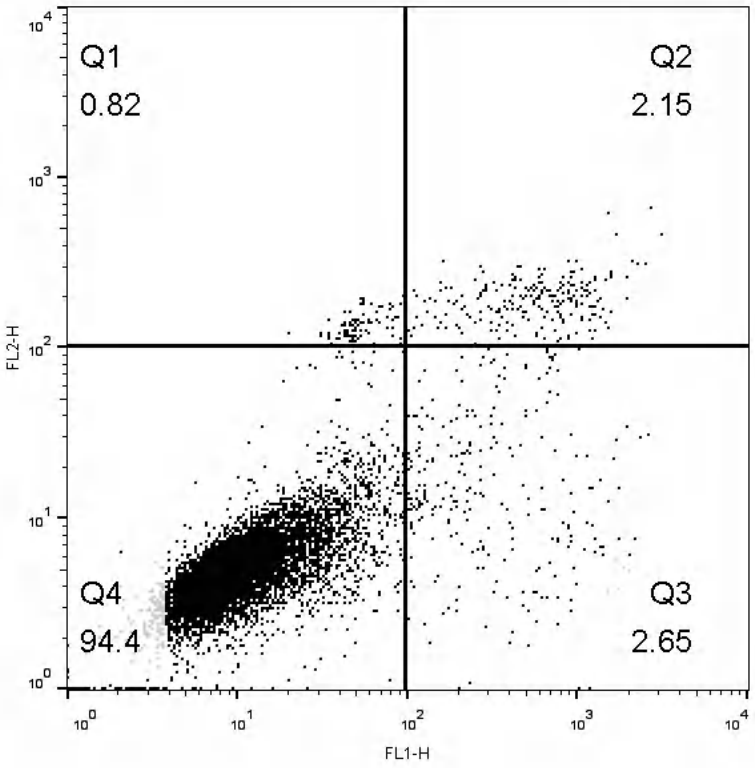
Q1
0.82
Q2
2.15
Q4
94.4
Q3
2.65
FL2-H
FL1-H
10^0
10^1
10^2
10^3
10^4

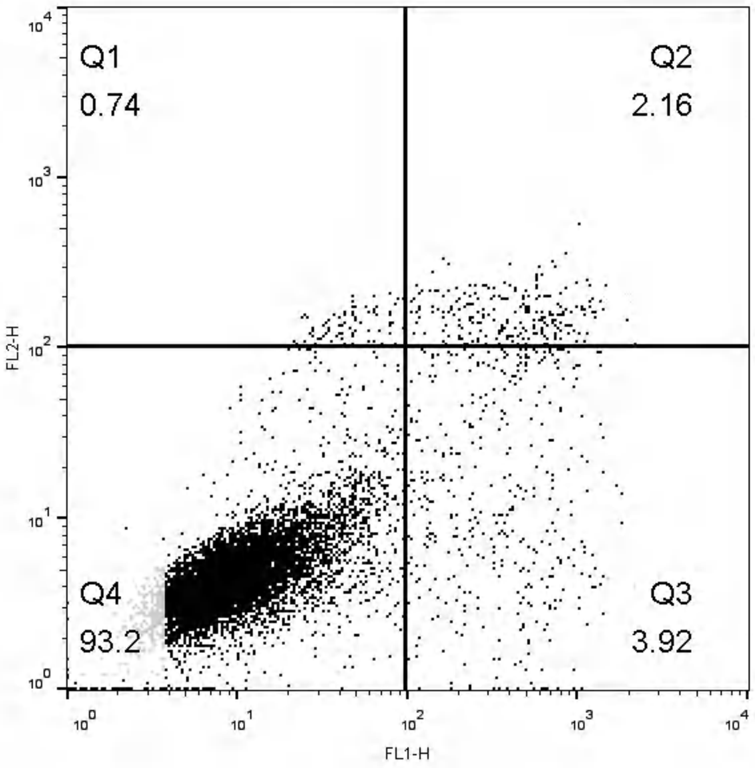
Q1
0.74
Q2
2.16
Q4
93.2
Q3
3.92
FL2-H
FL1-H
10^0
10^1
10^2
10^3
10^4

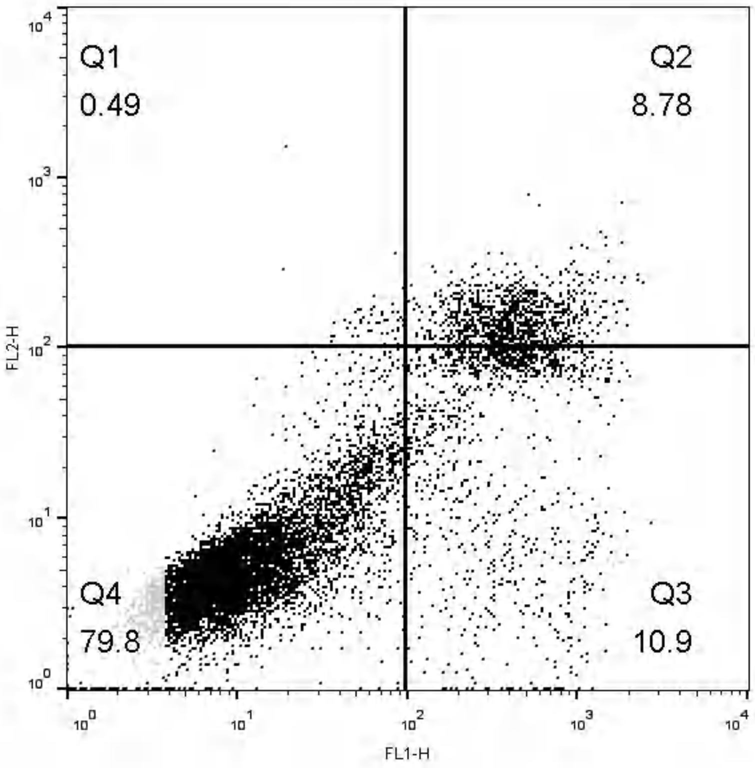
Q1
0.49
Q2
8.78
Q4
79.8
Q3
10.9
FL2-H
FL1-H

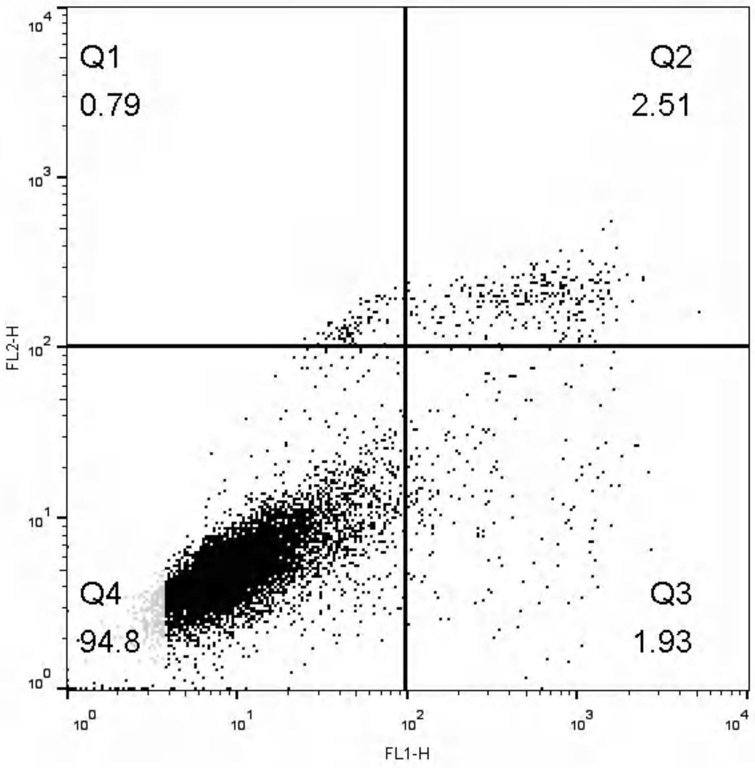
Q1
0.79
Q2
2.51
Q4
94.8
Q3
1.93
FL2-H
FL1-H

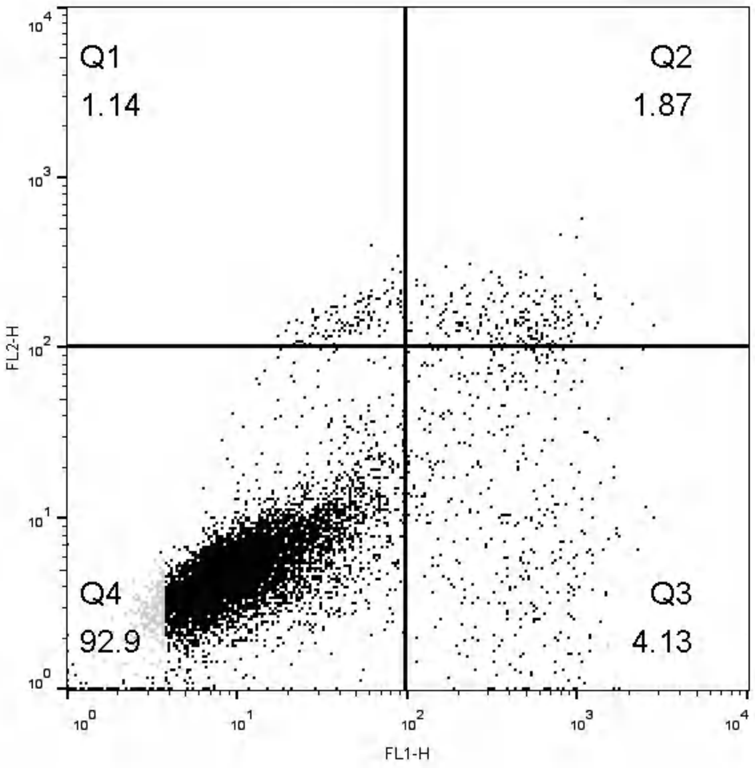
Q1
1.14
Q2
1.87
Q3
4.13
Q4
92.9
FL1-H
FL2-H

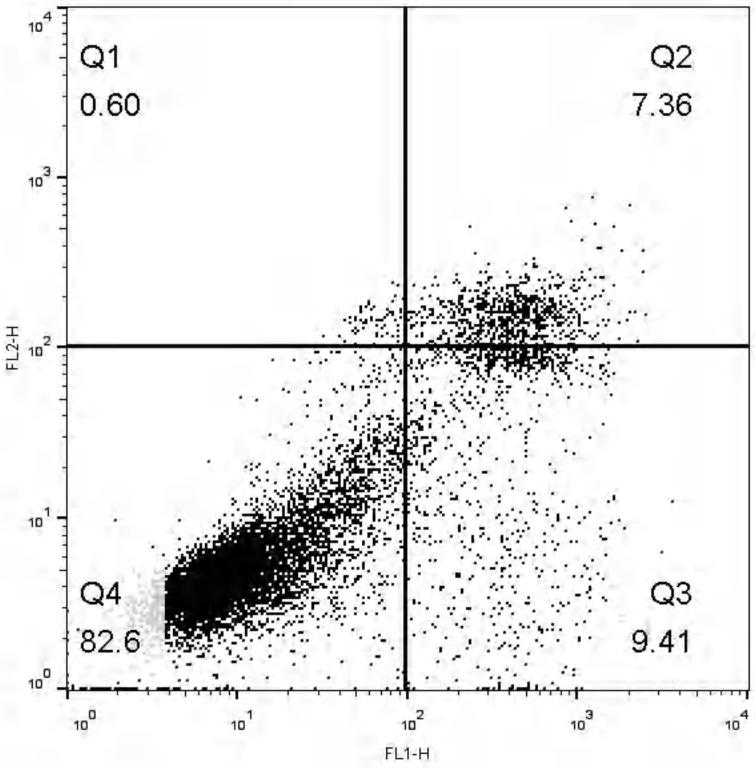
Q1
0.60
Q2
7.36
Q4
82.6
Q3
9.41
FL2-H
FL1-H

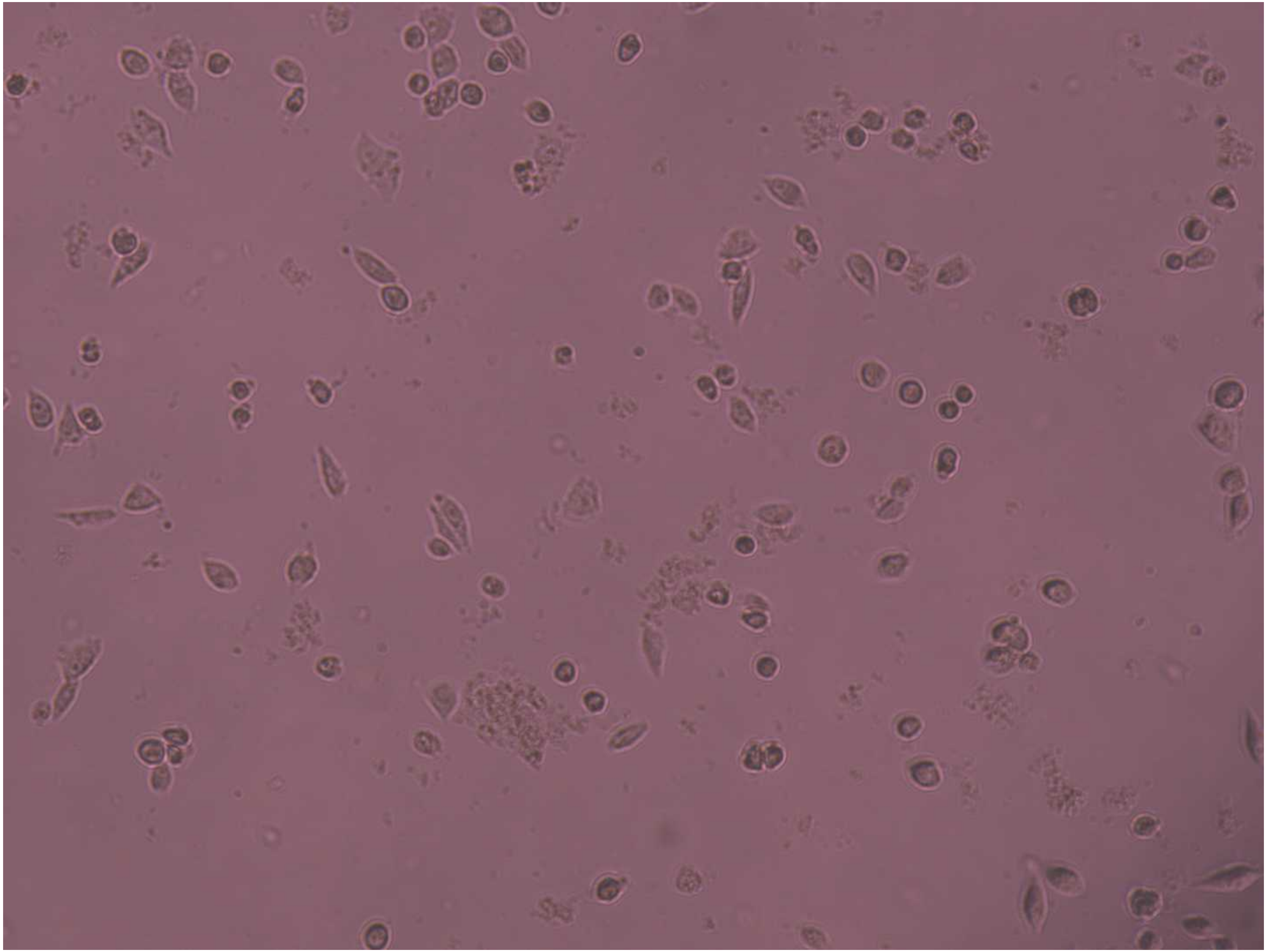

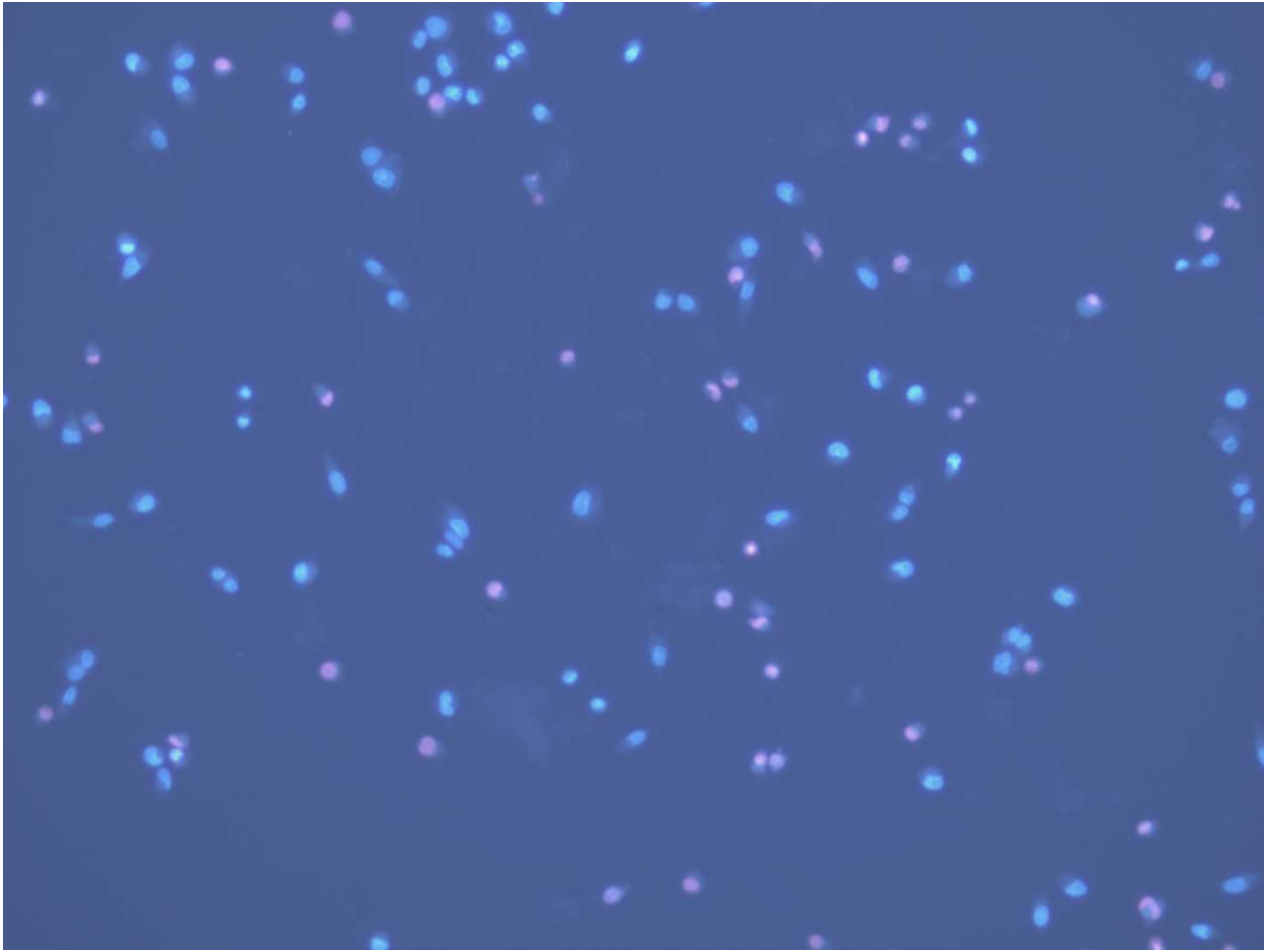

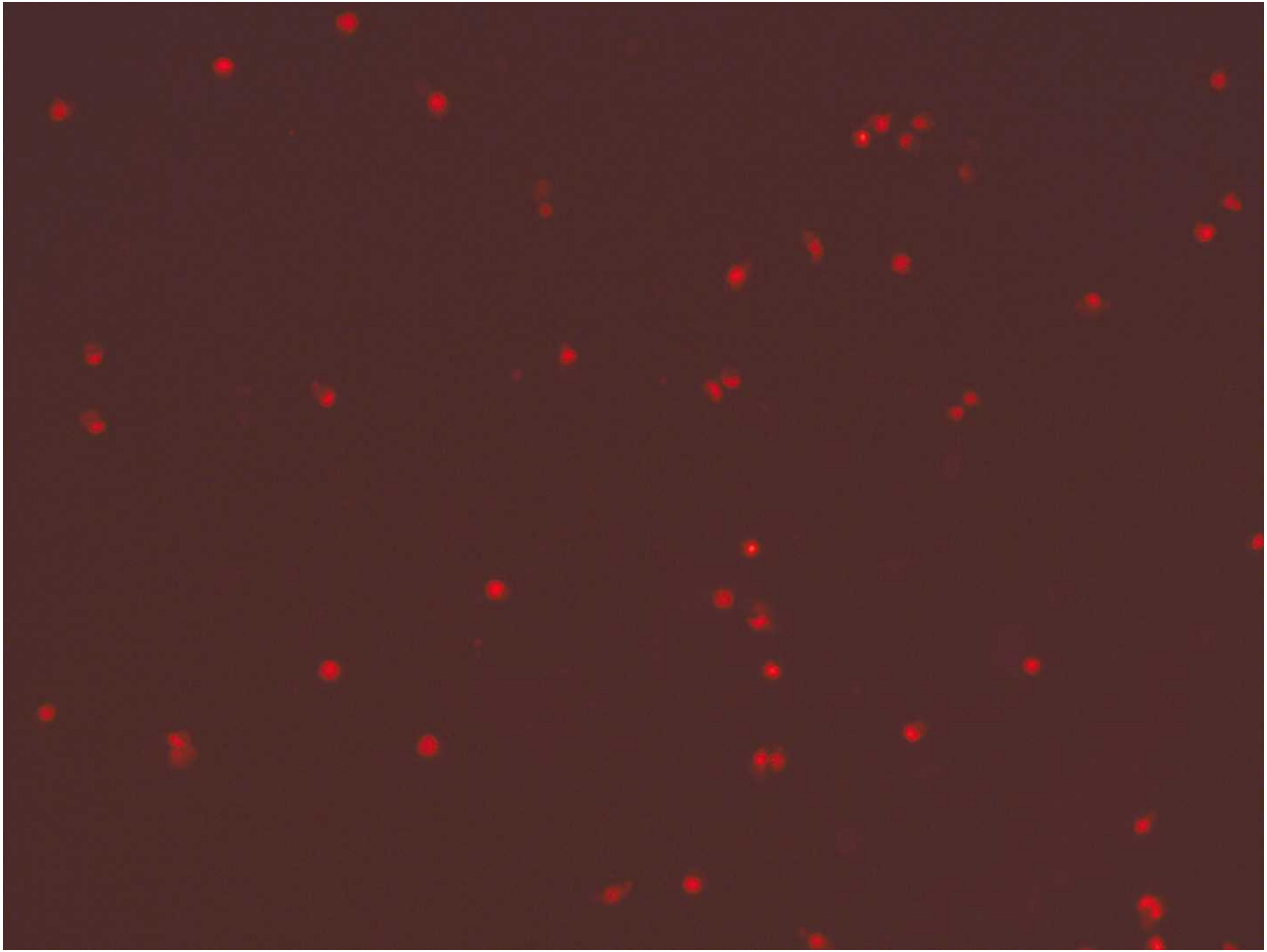

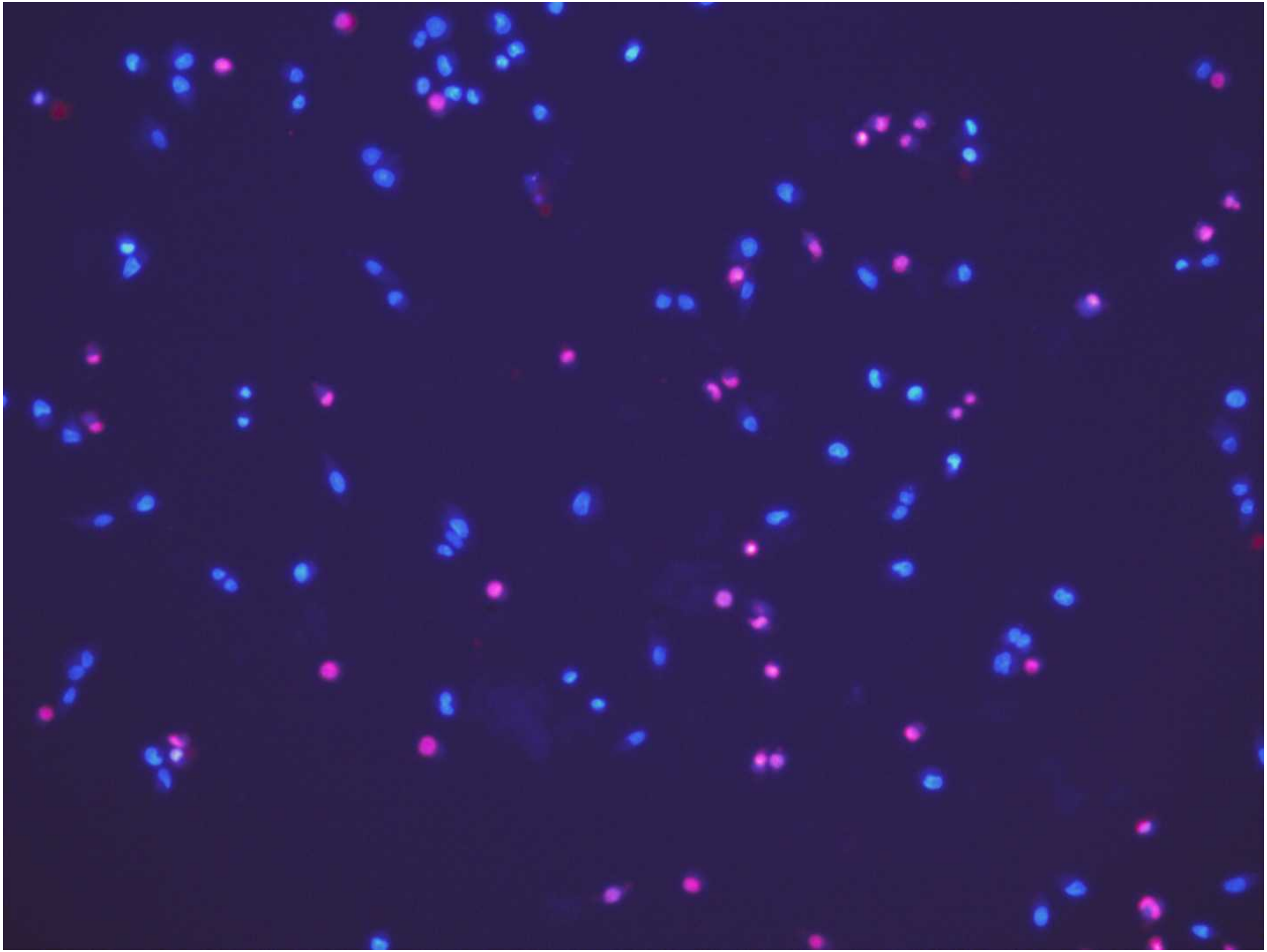

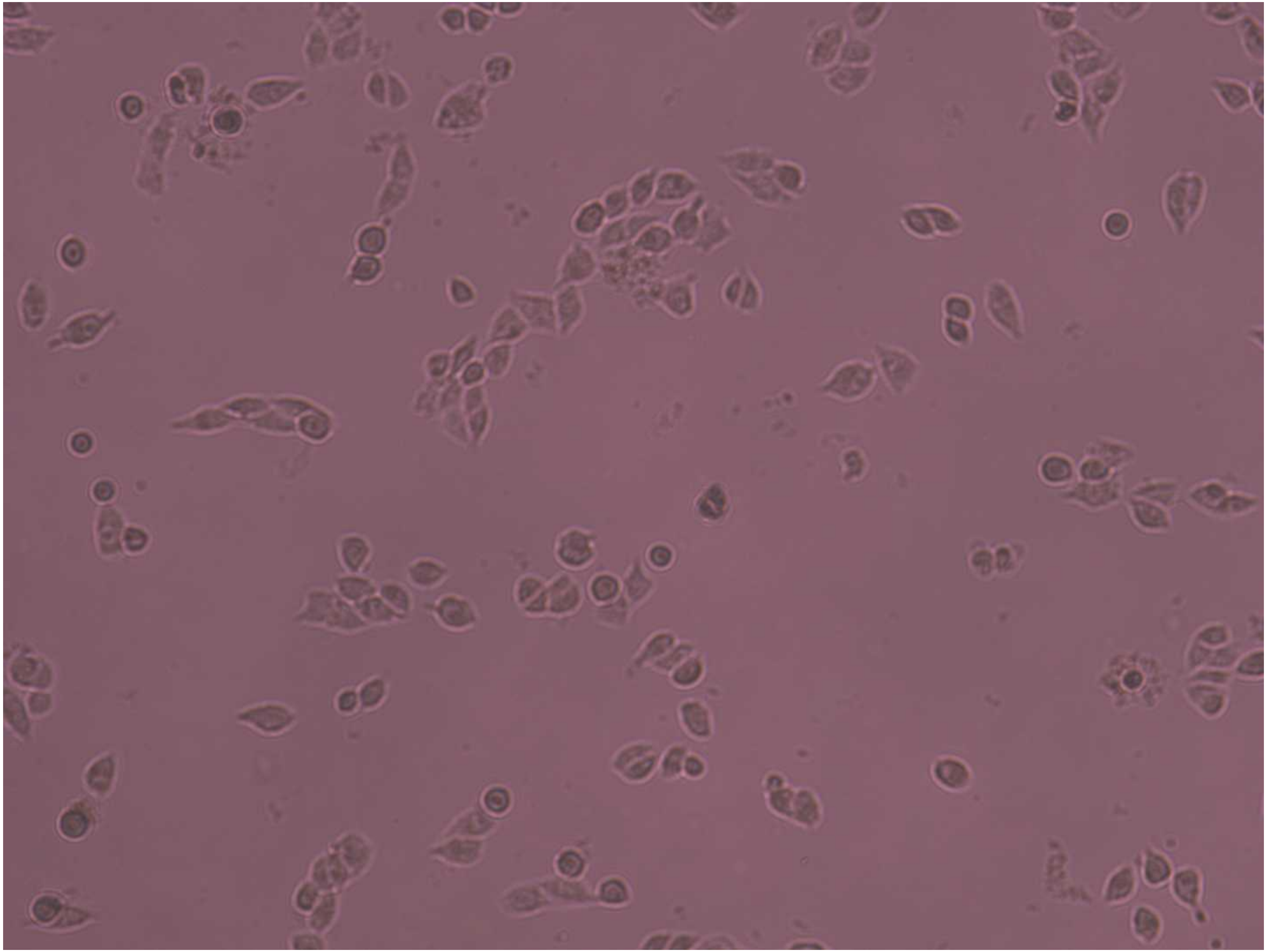

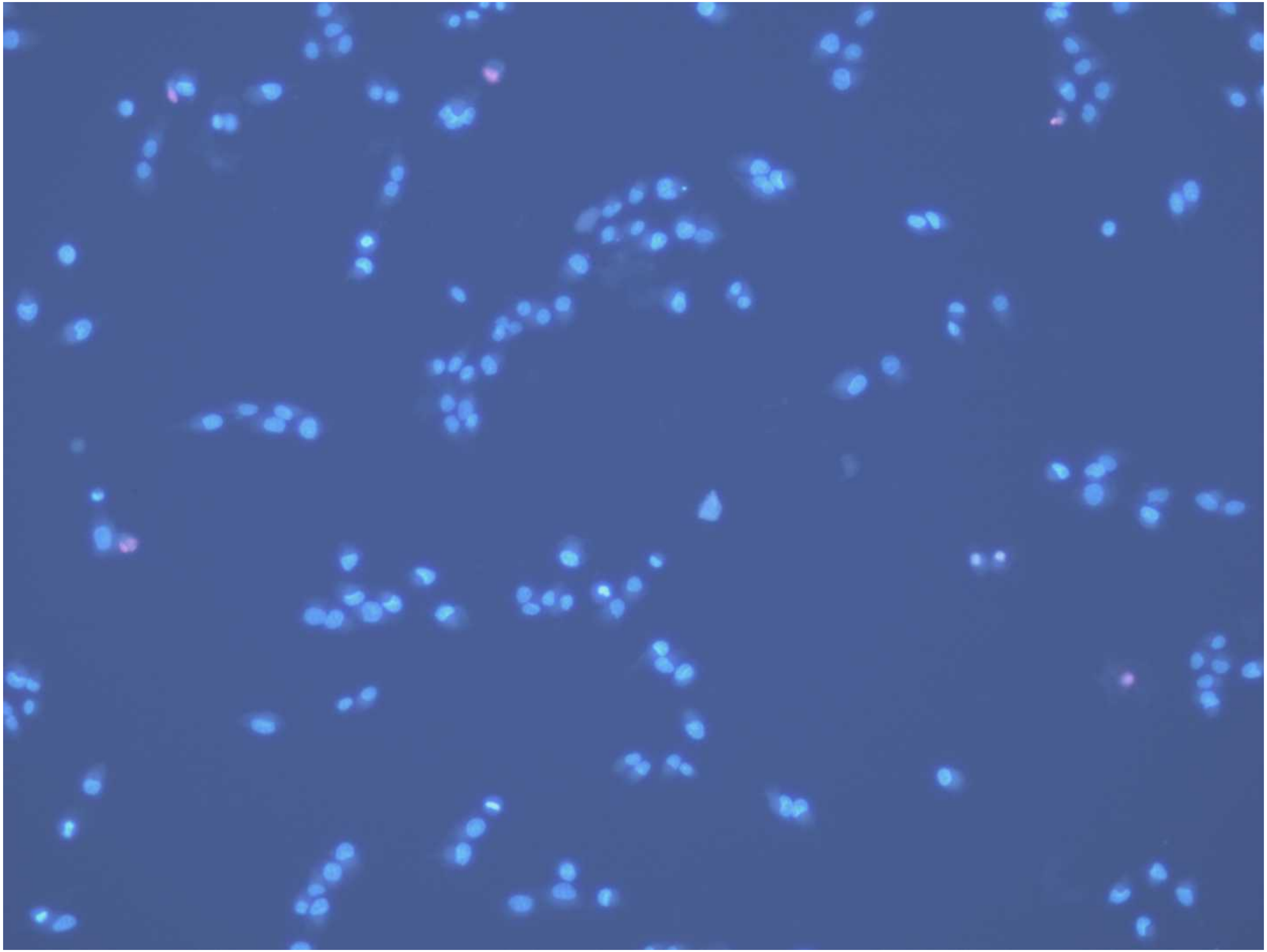

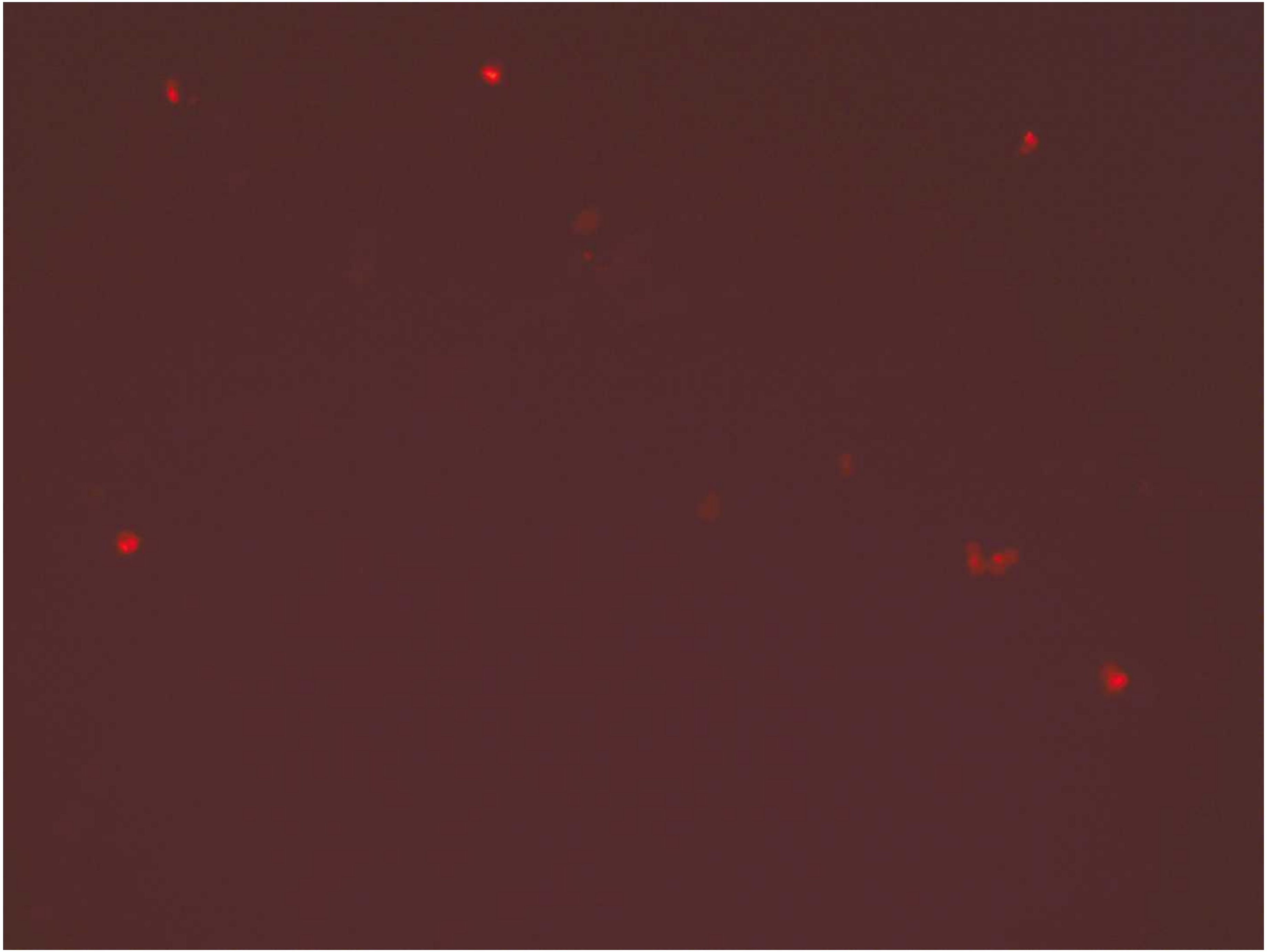

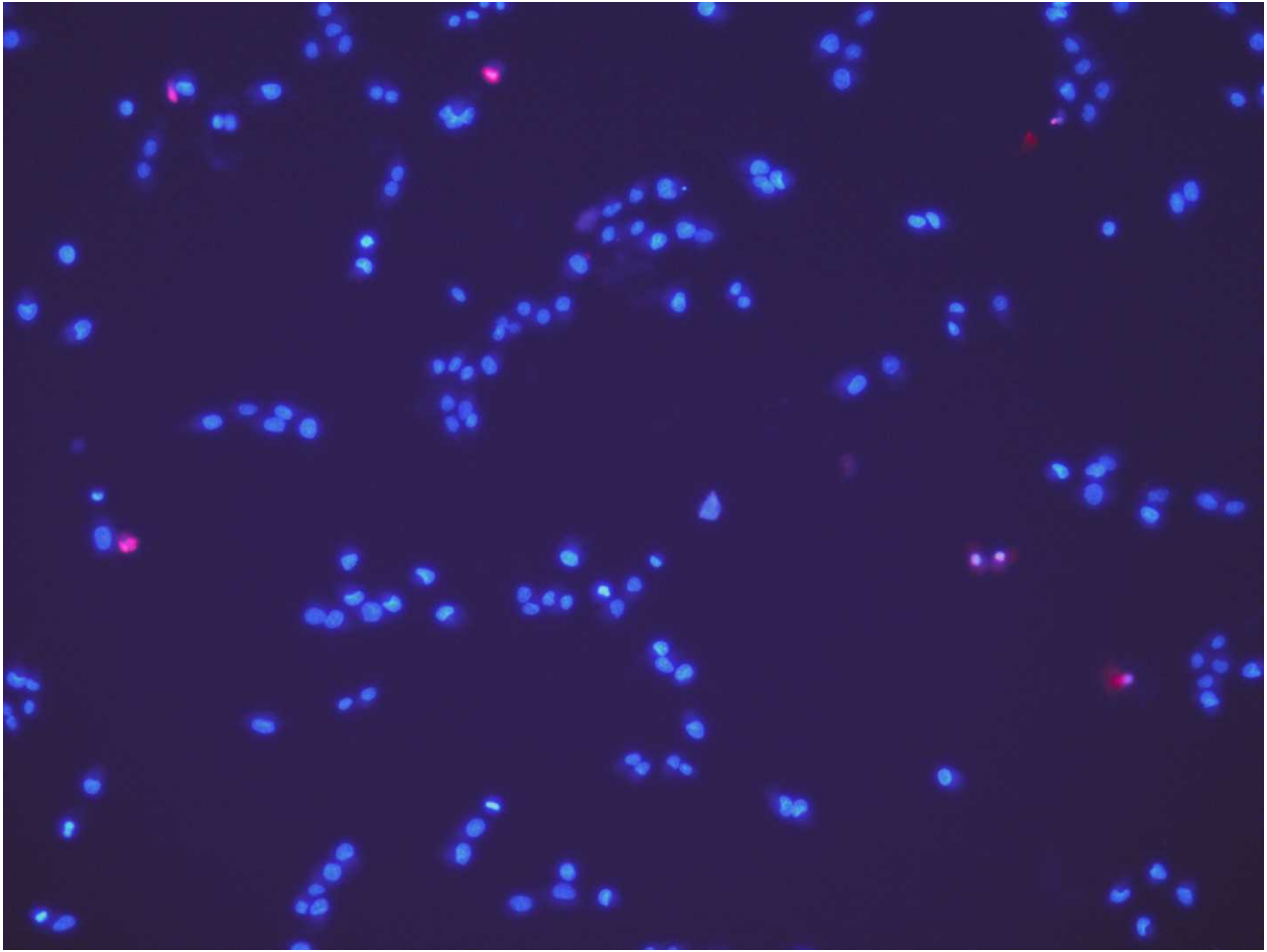

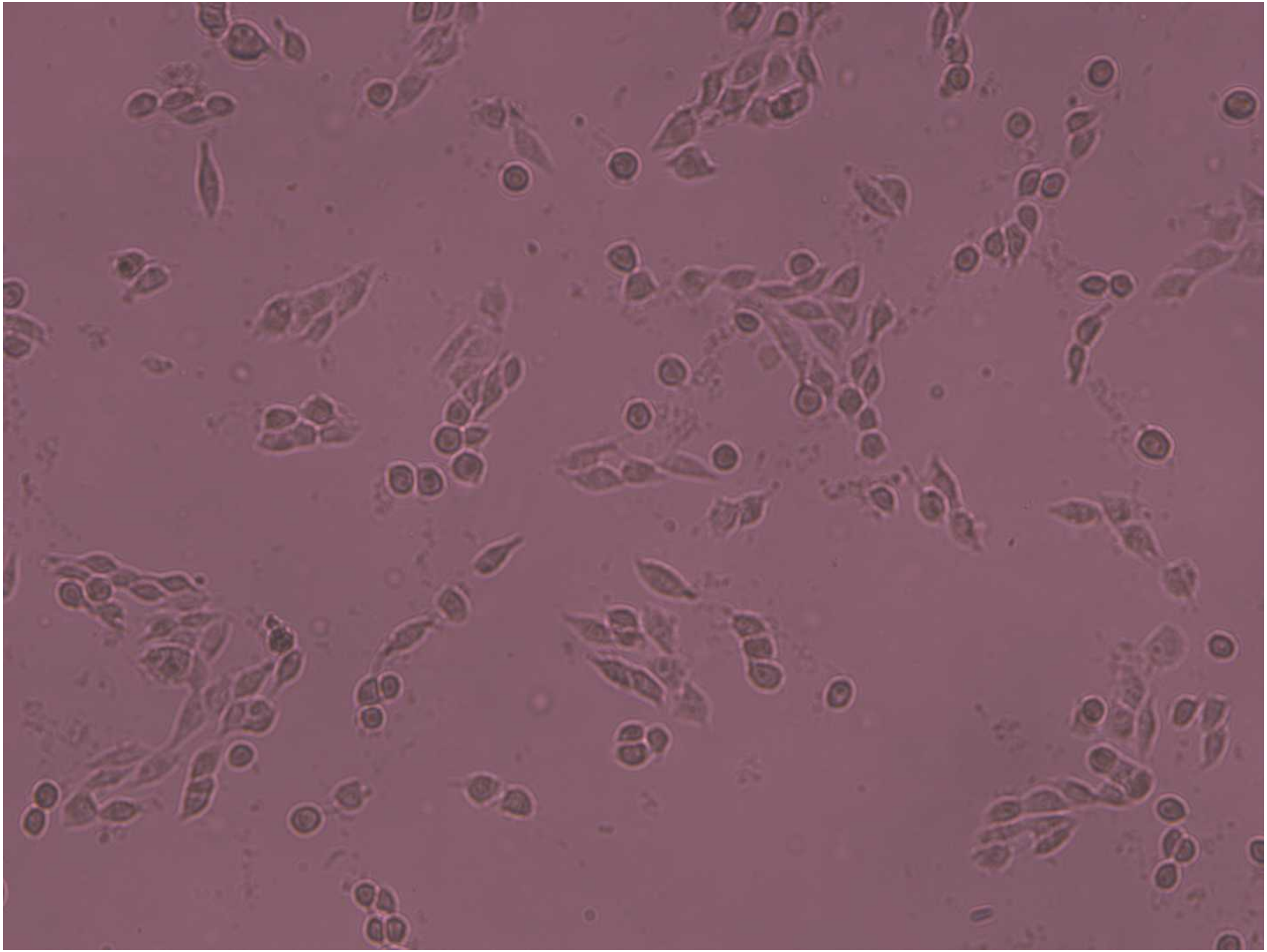

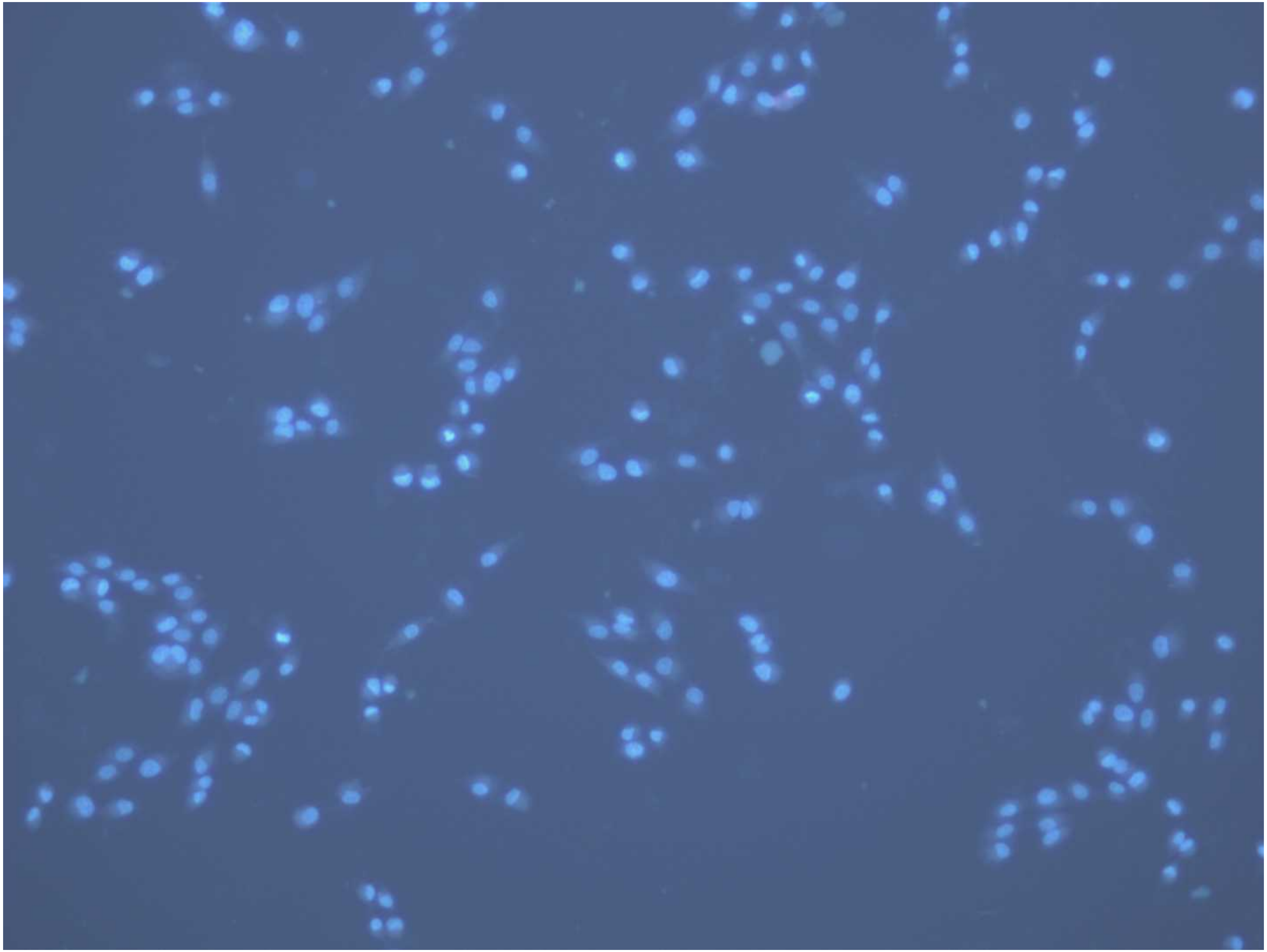

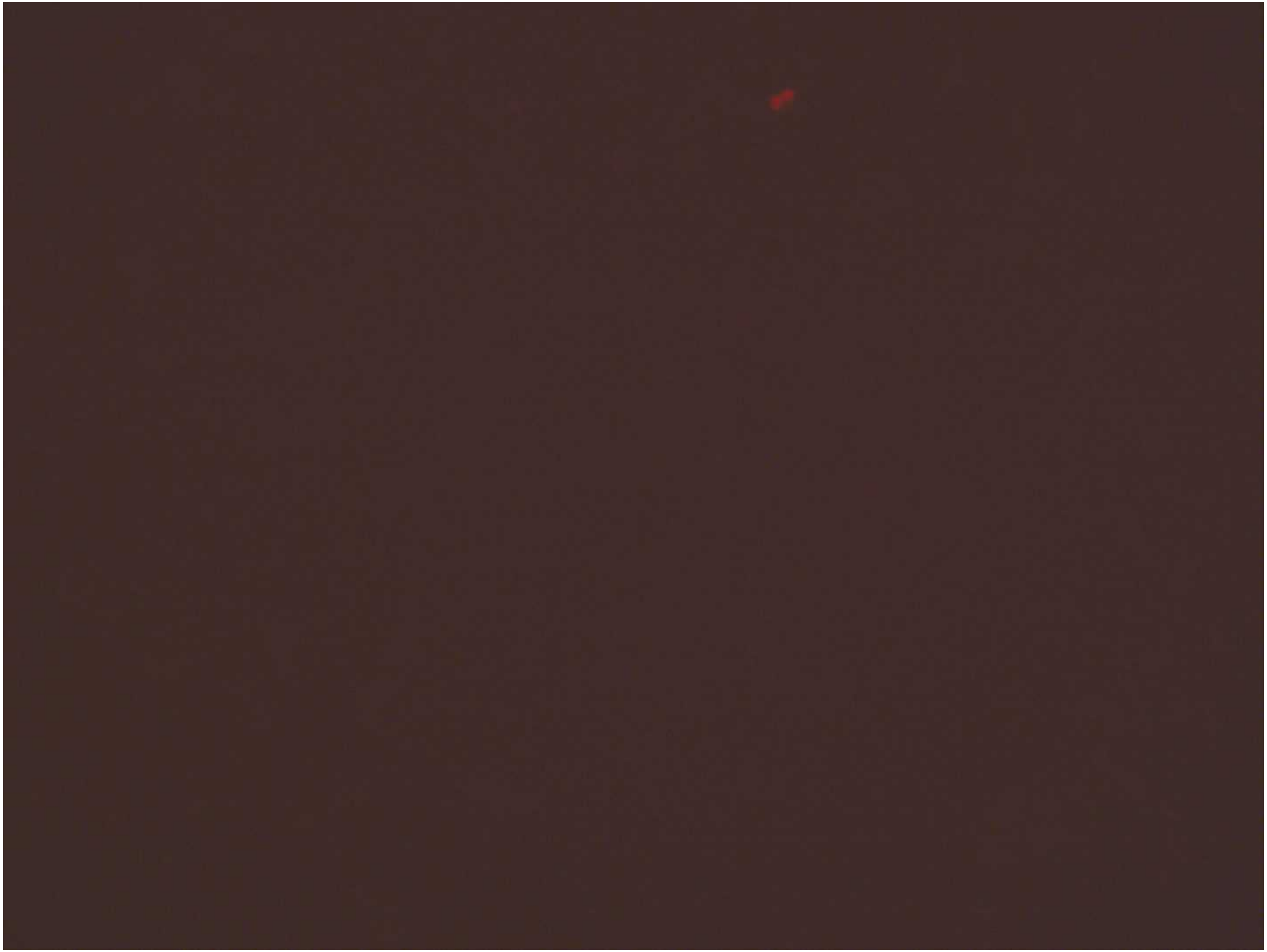

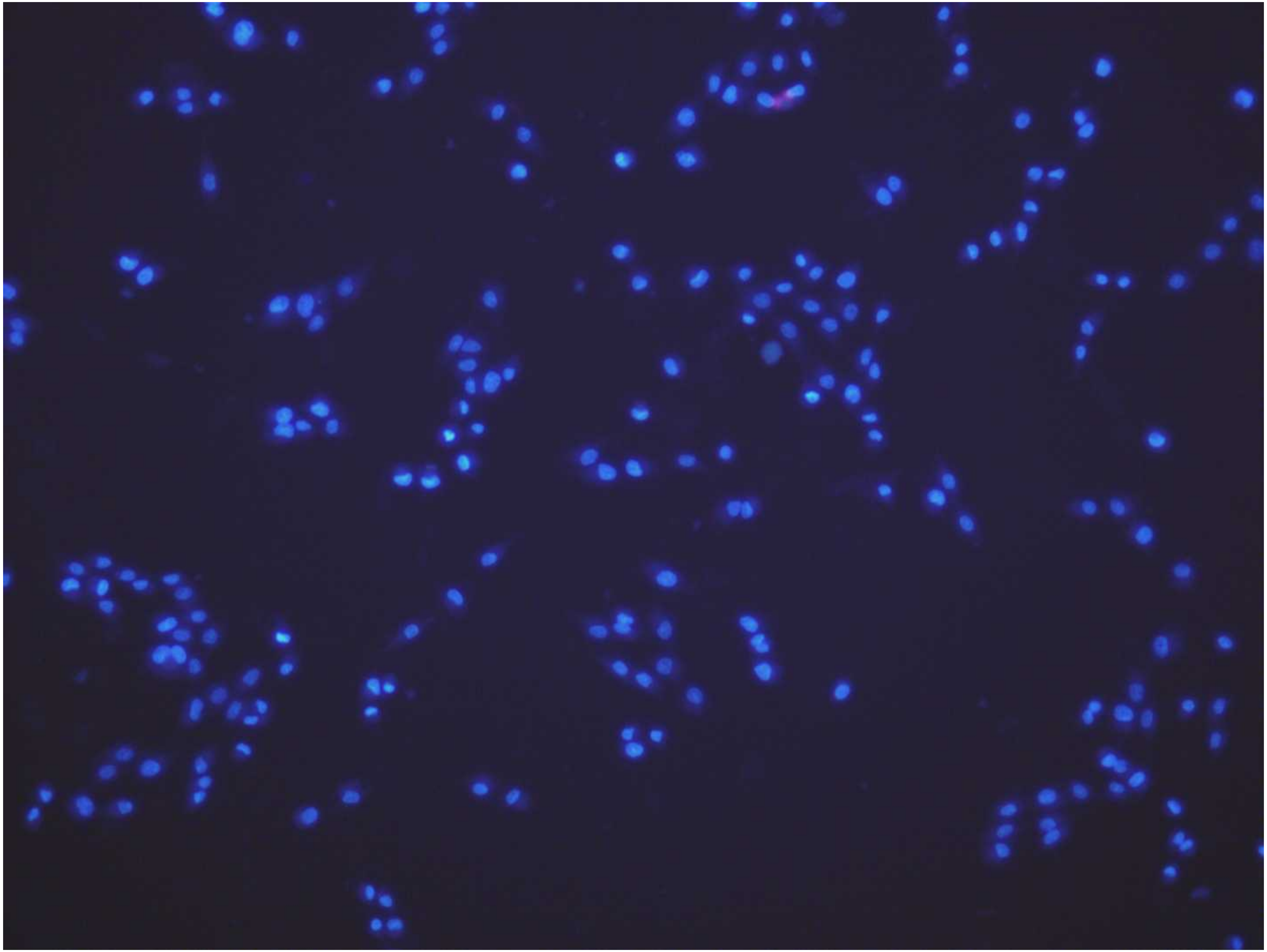

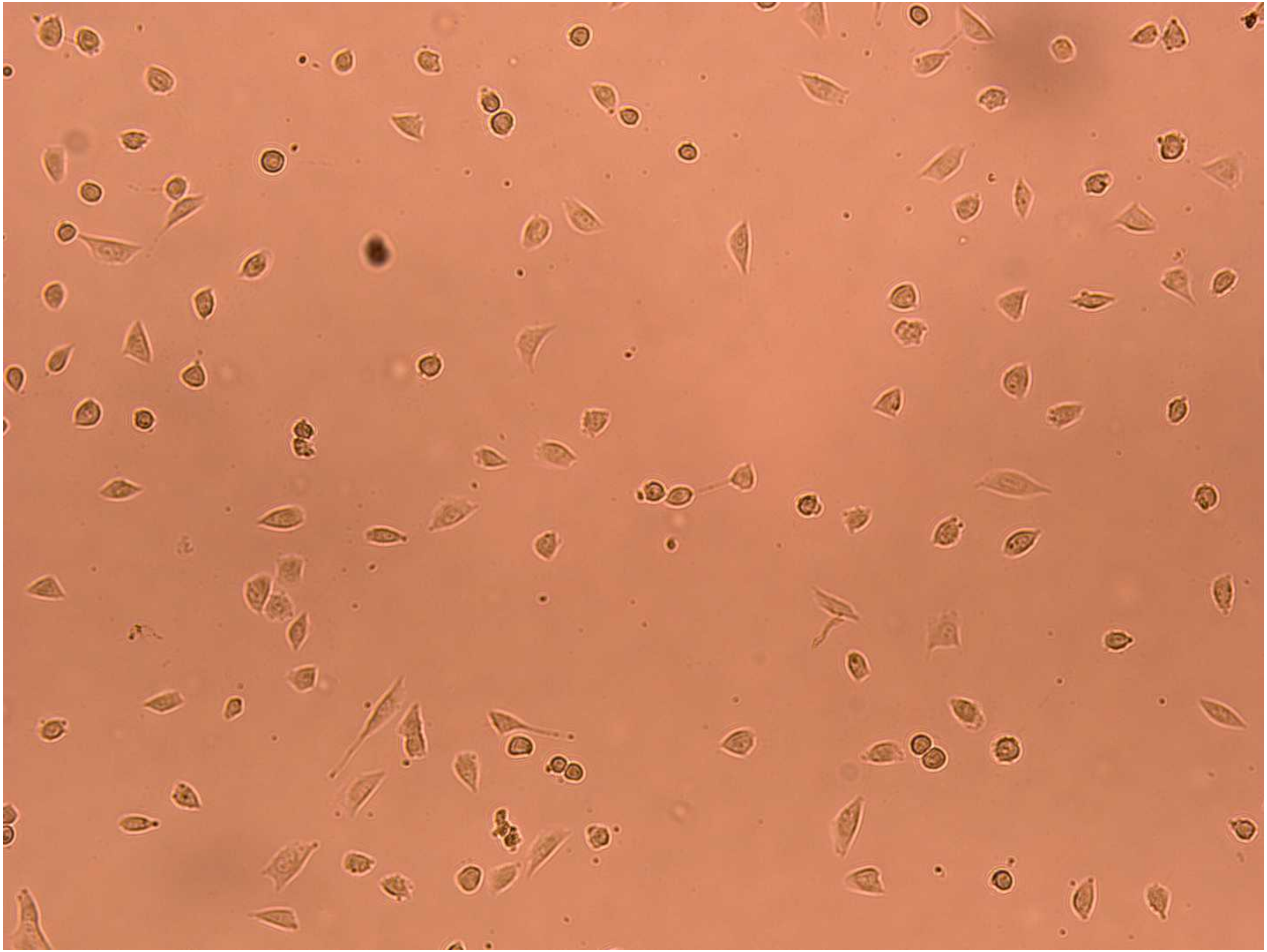

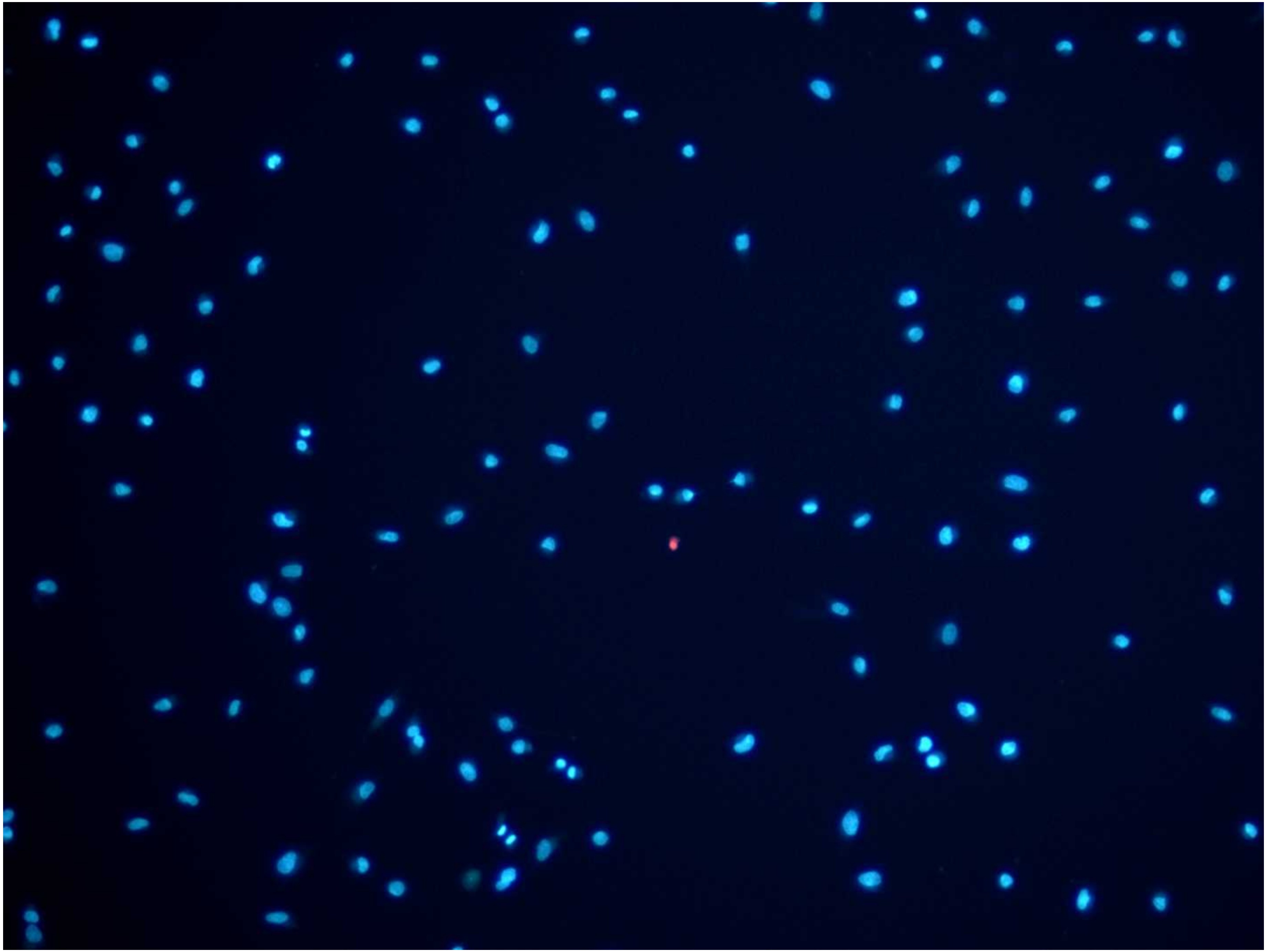

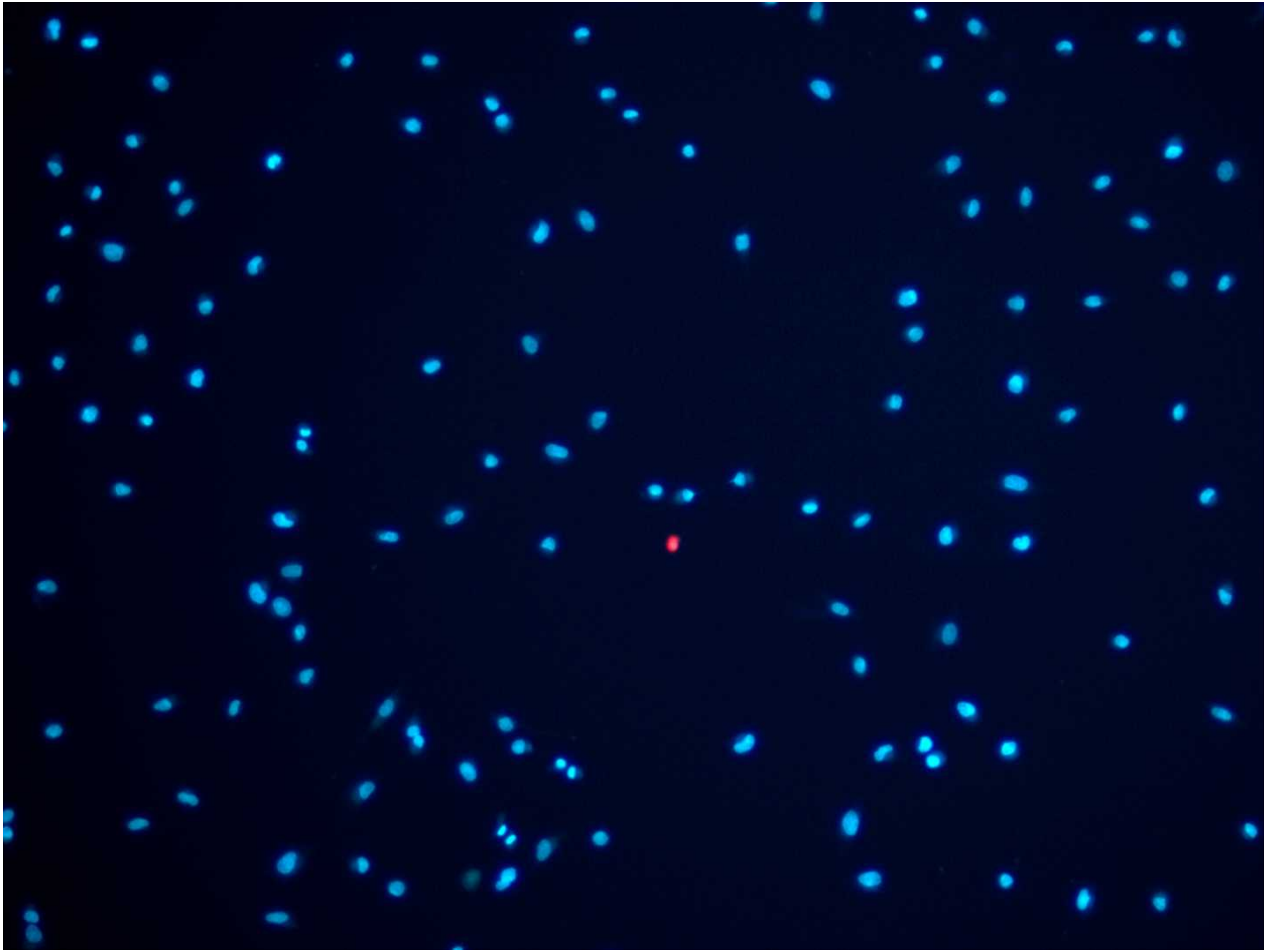

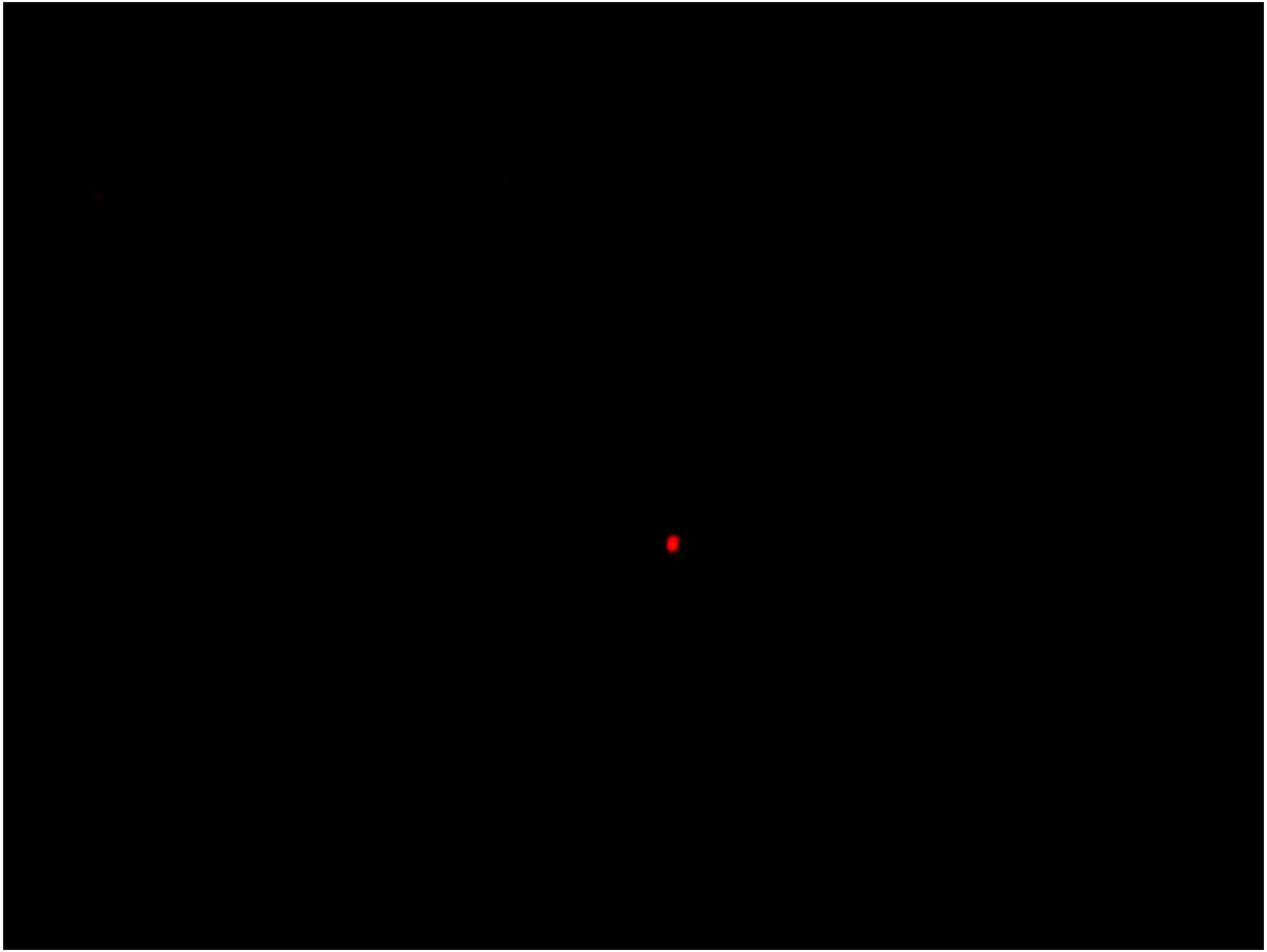

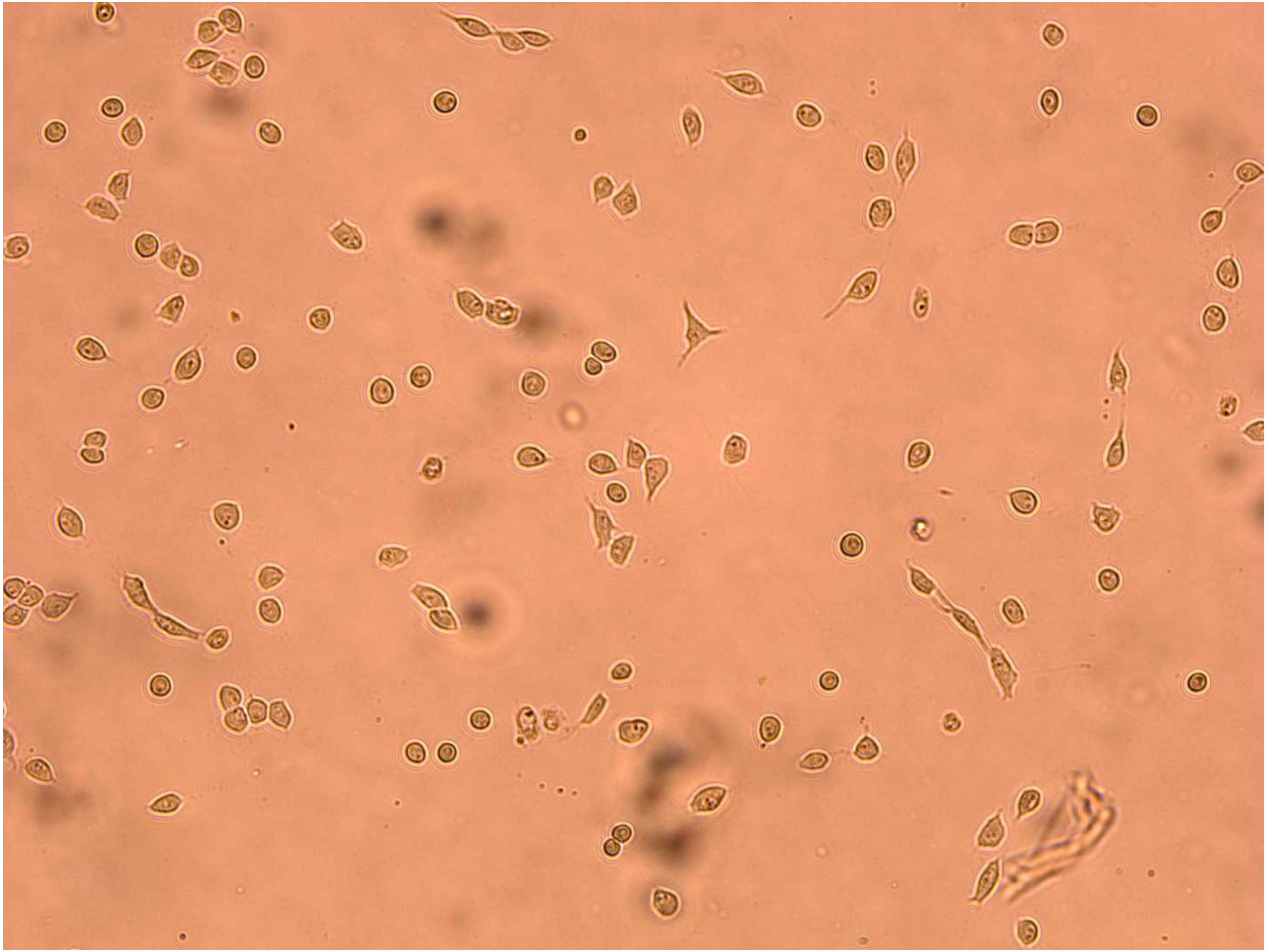

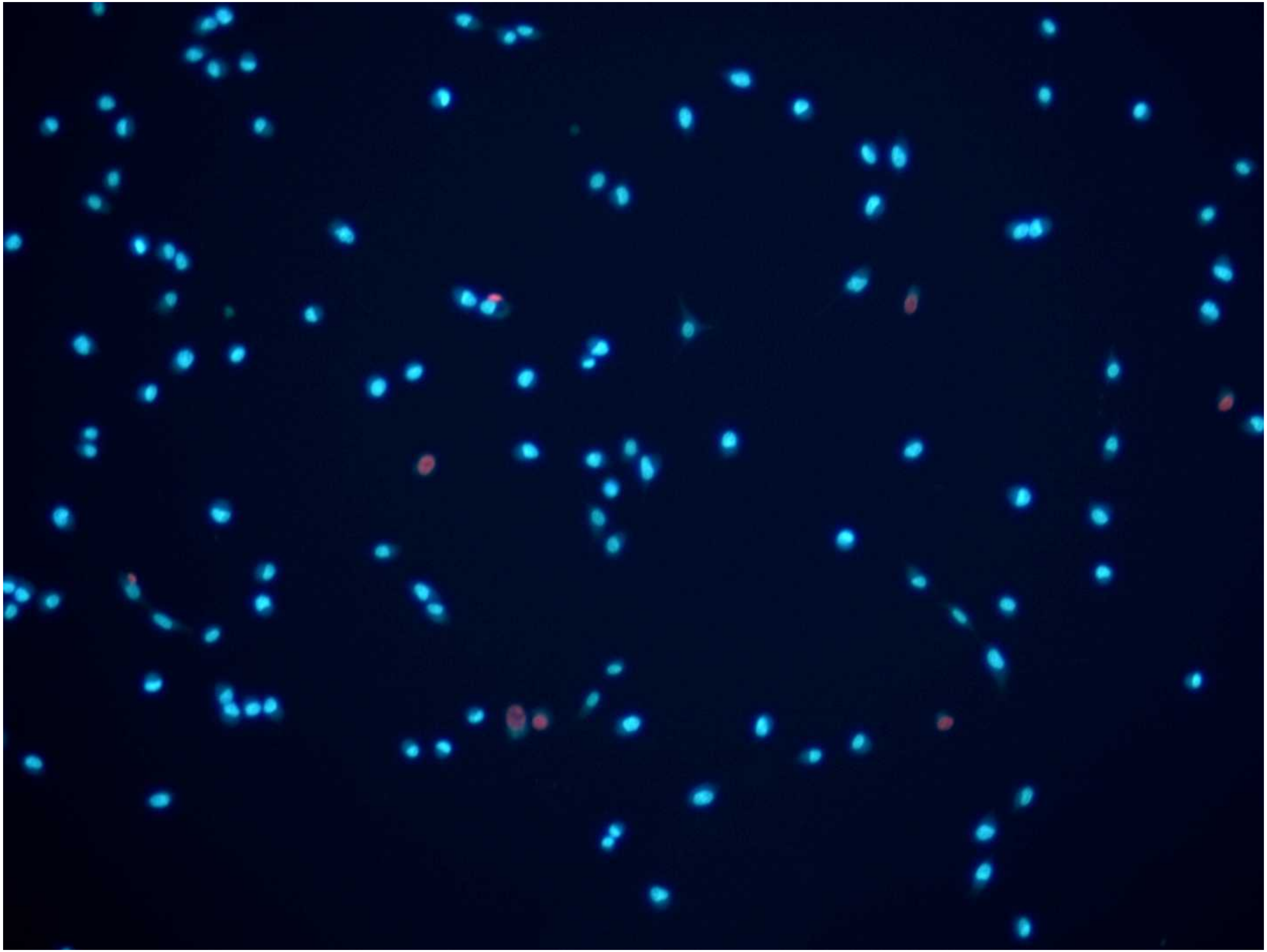

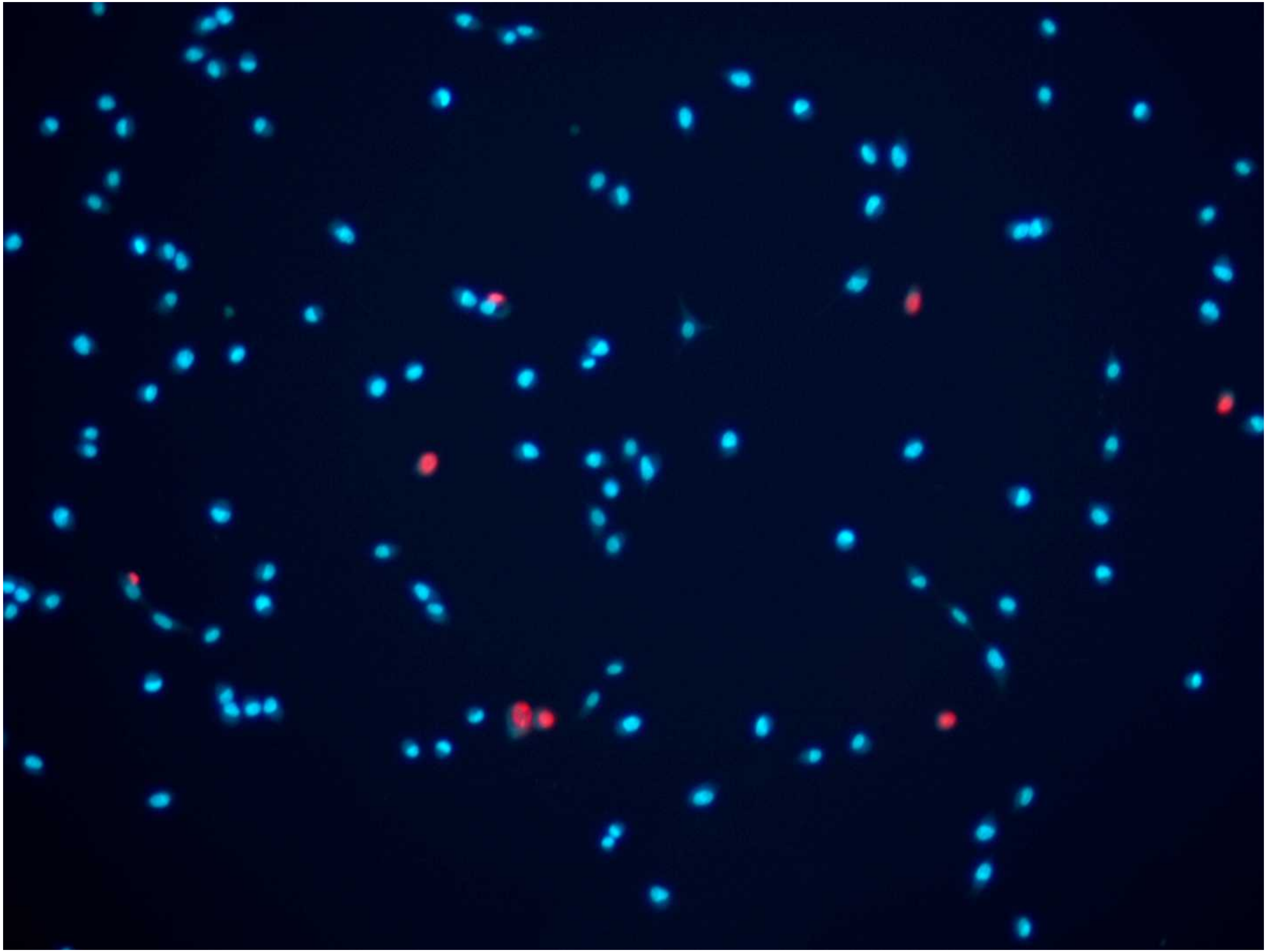

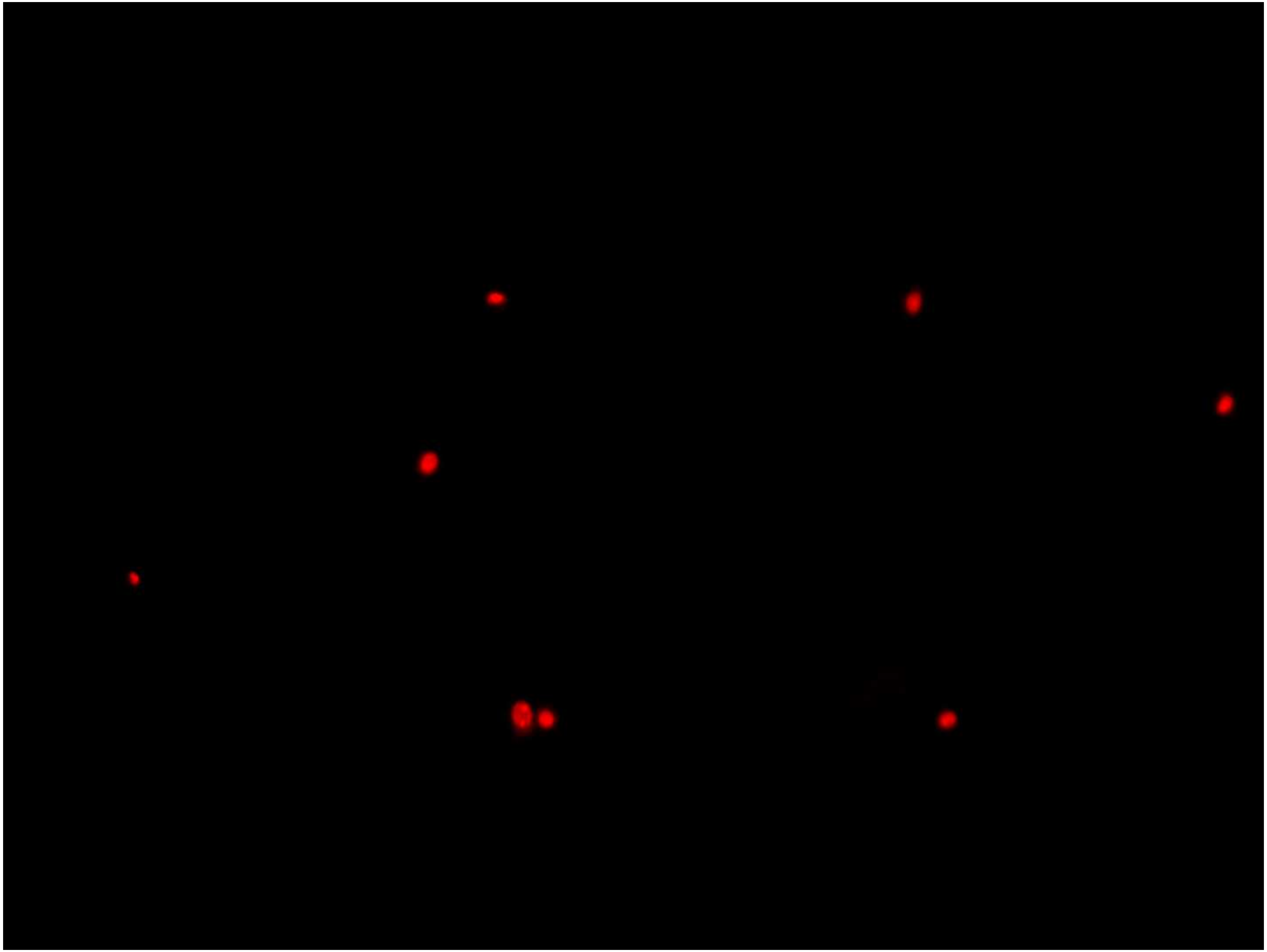

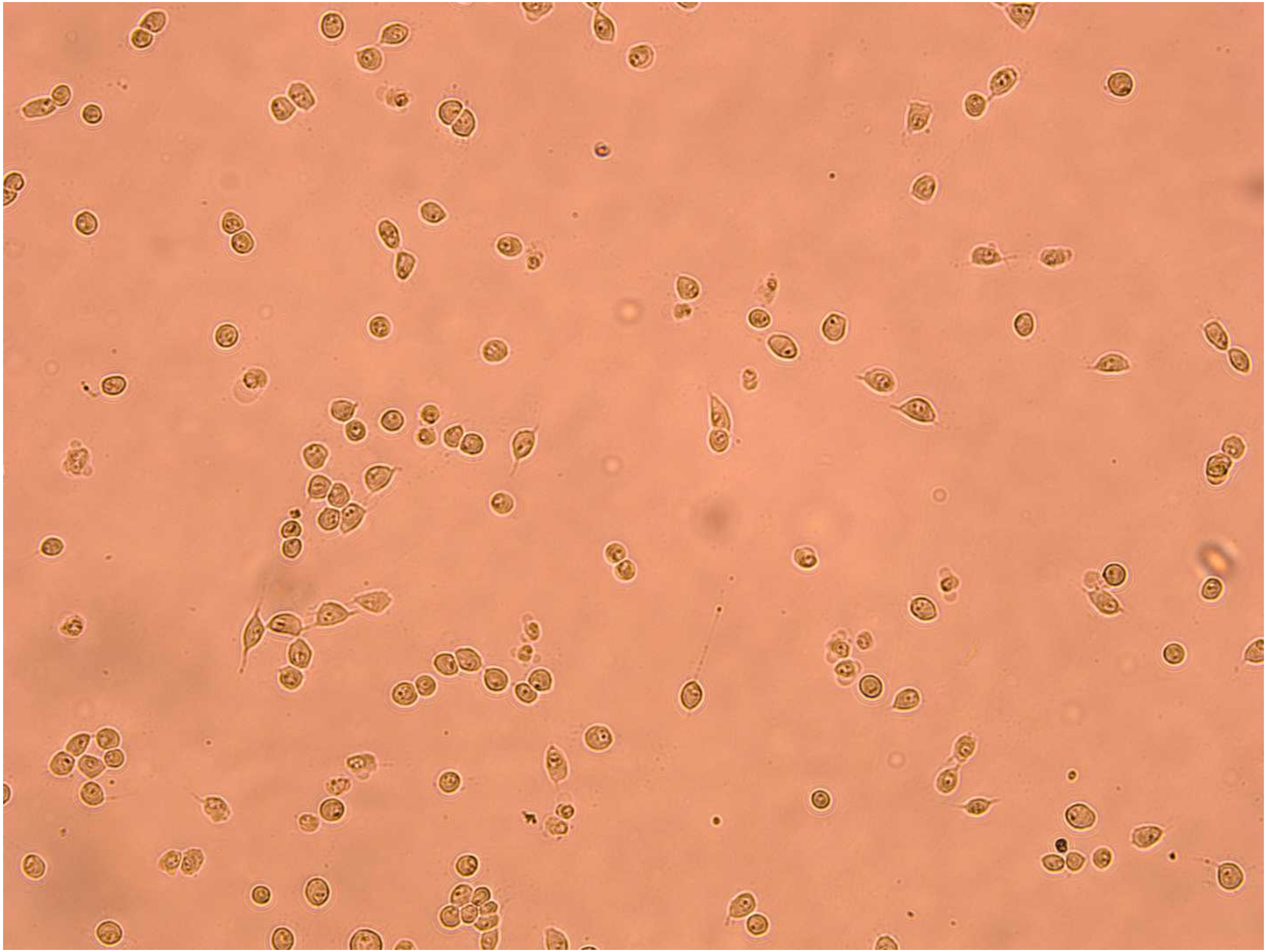

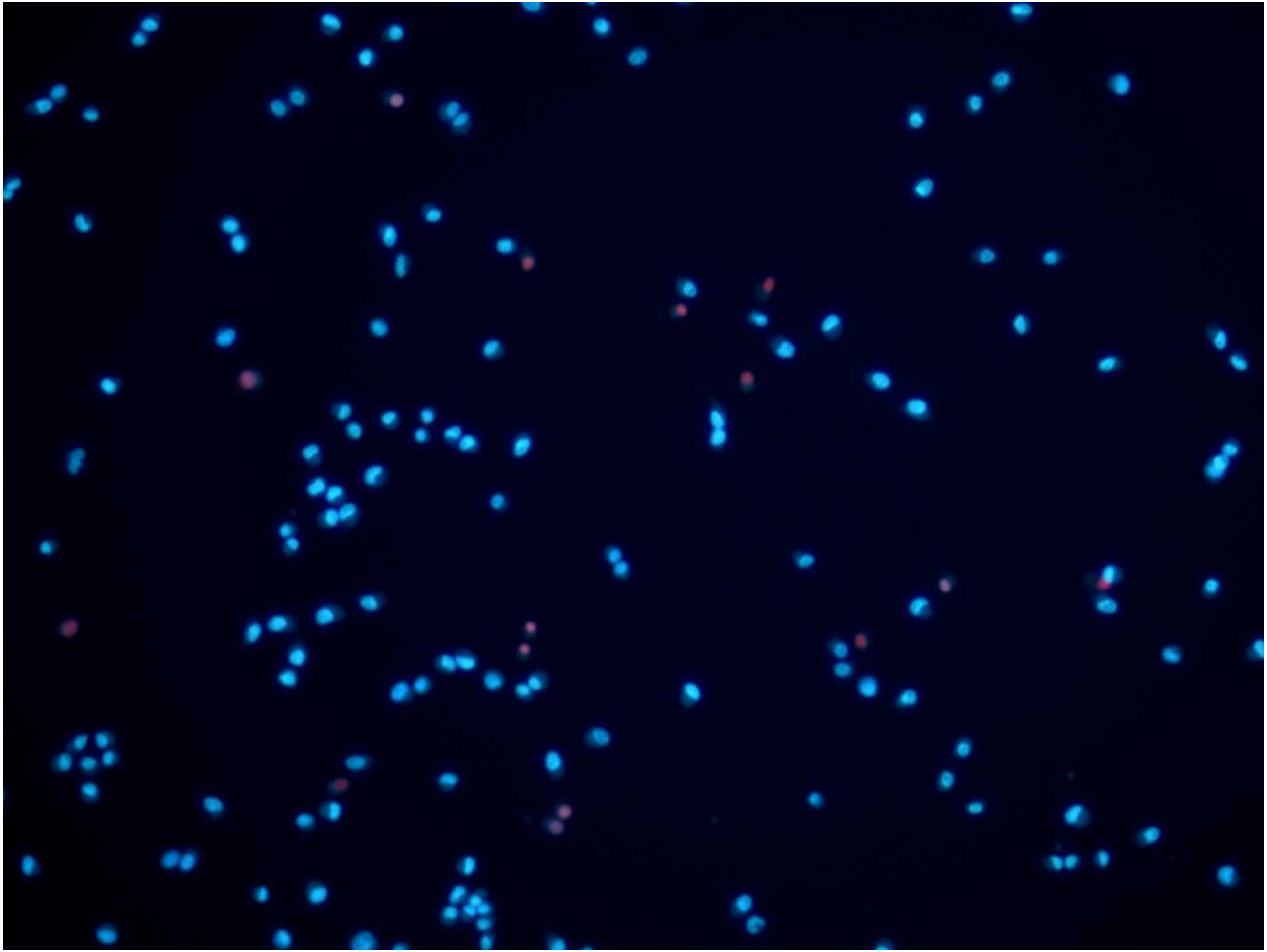

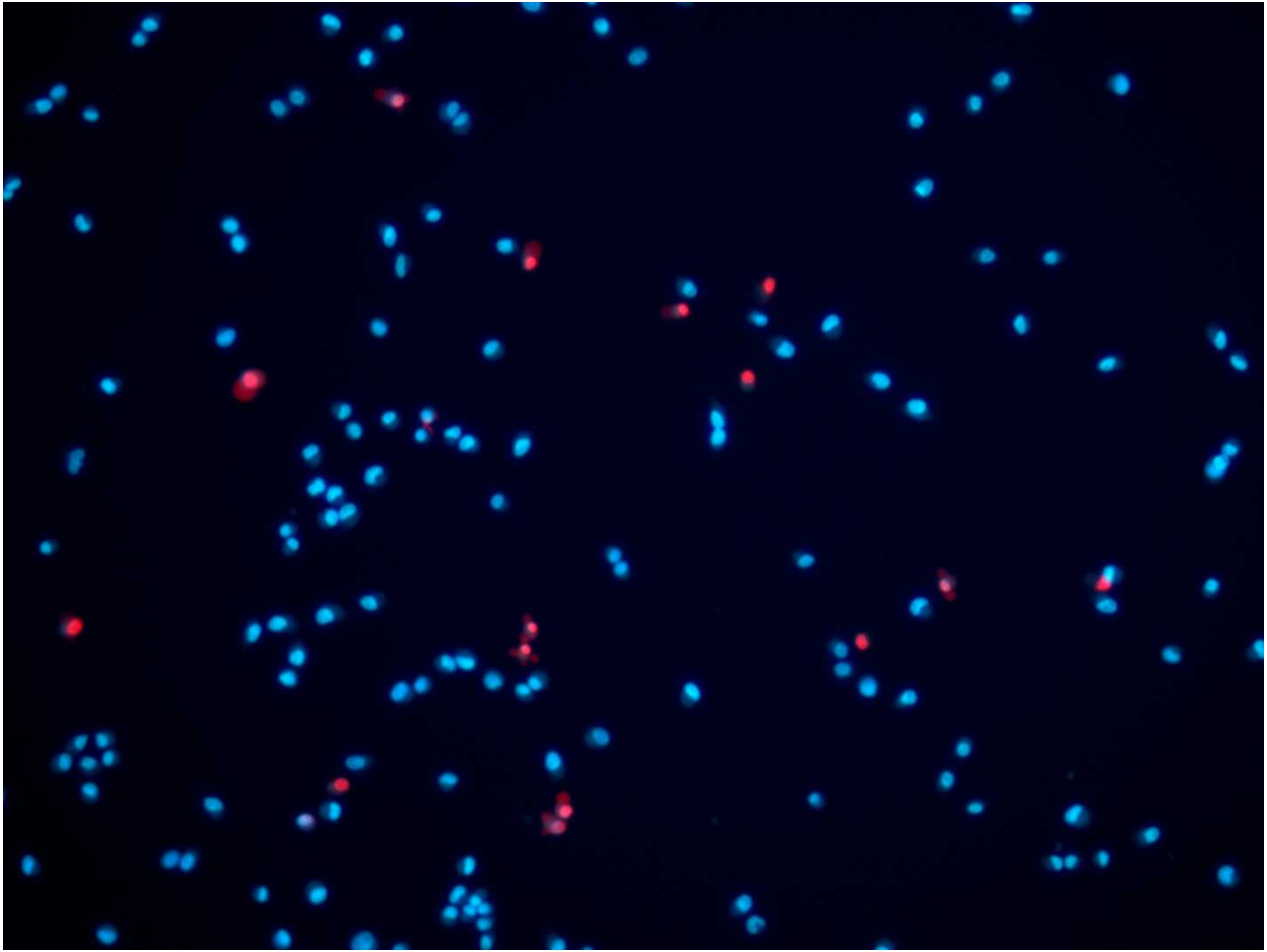

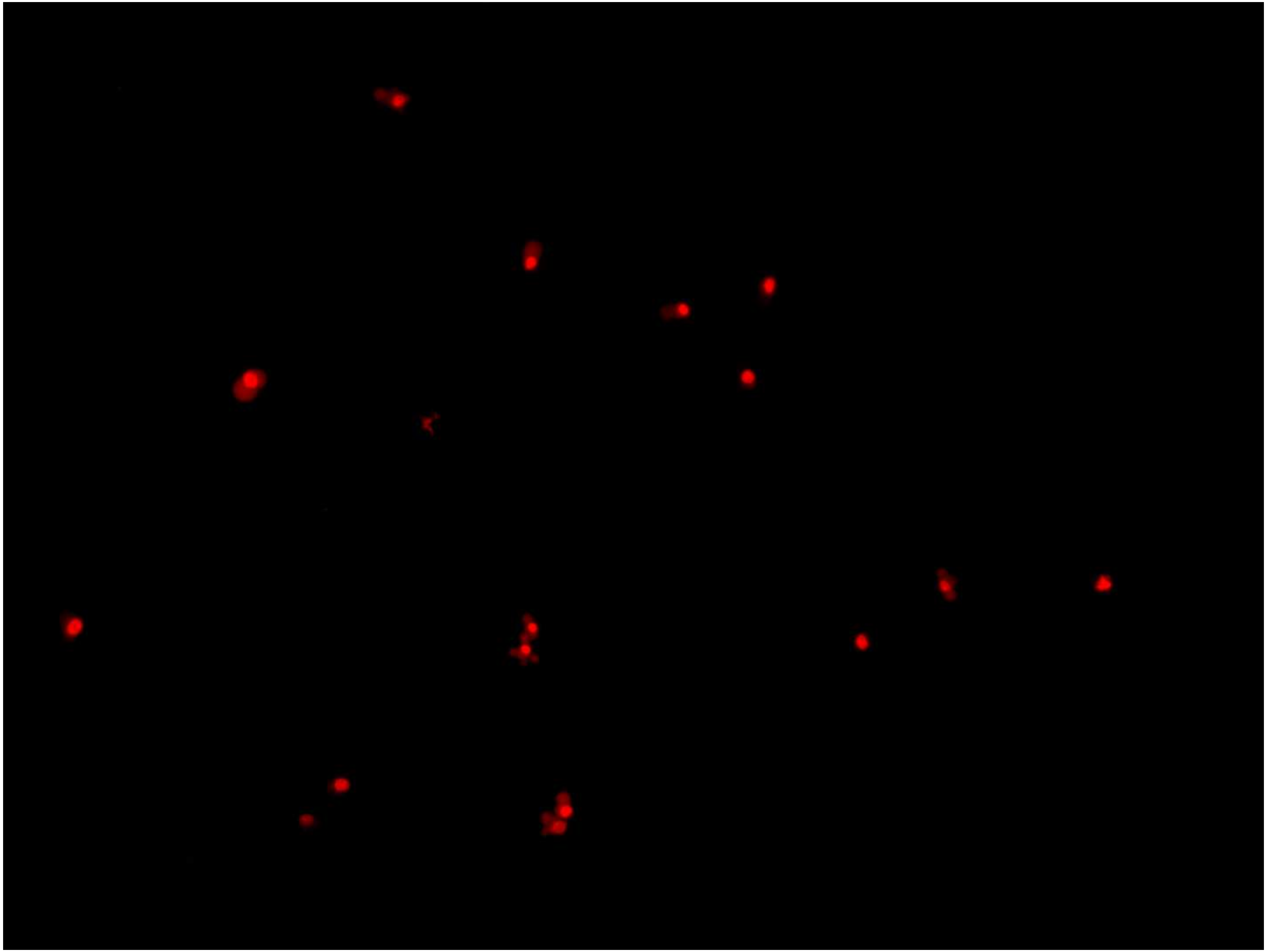

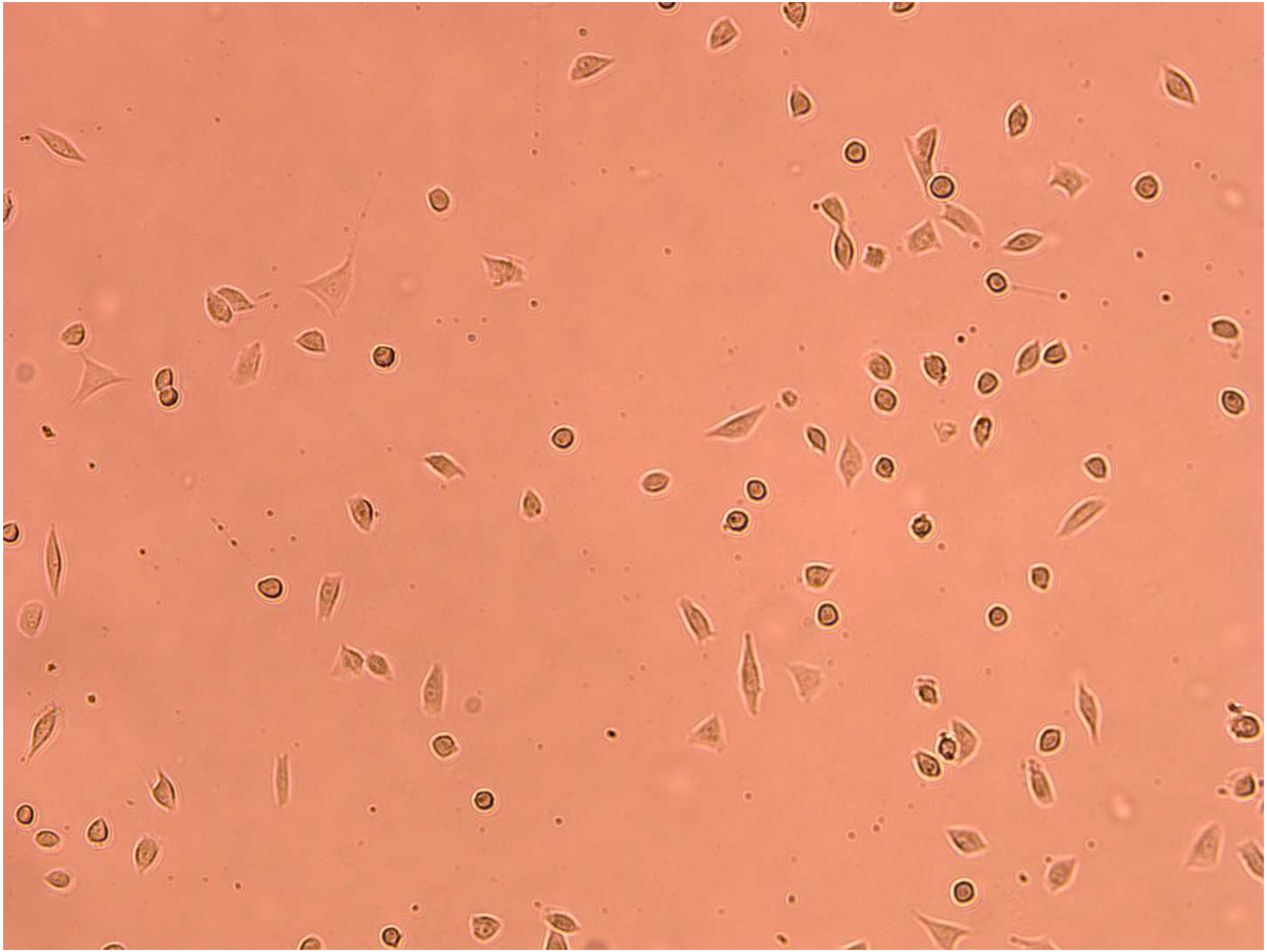

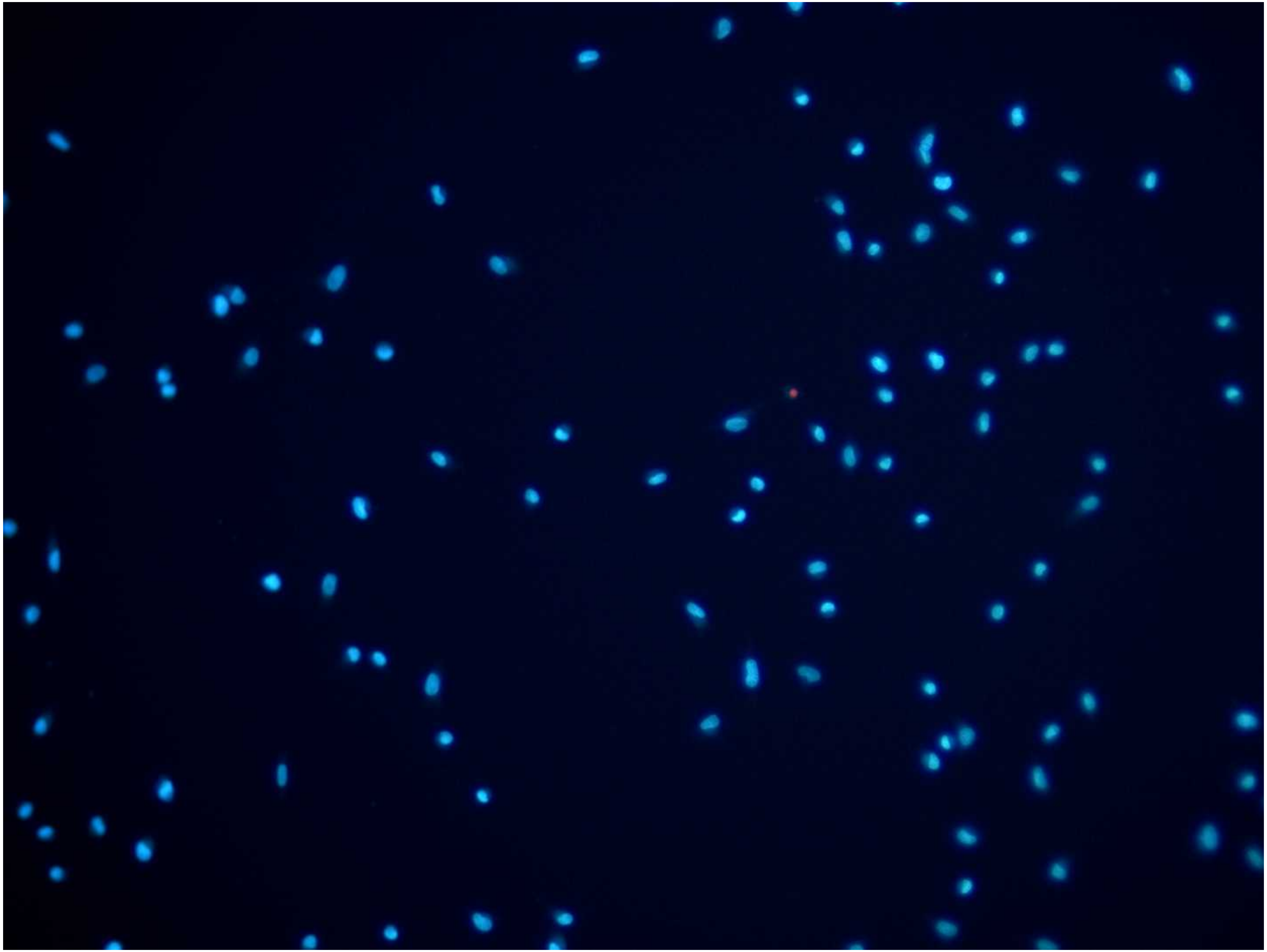

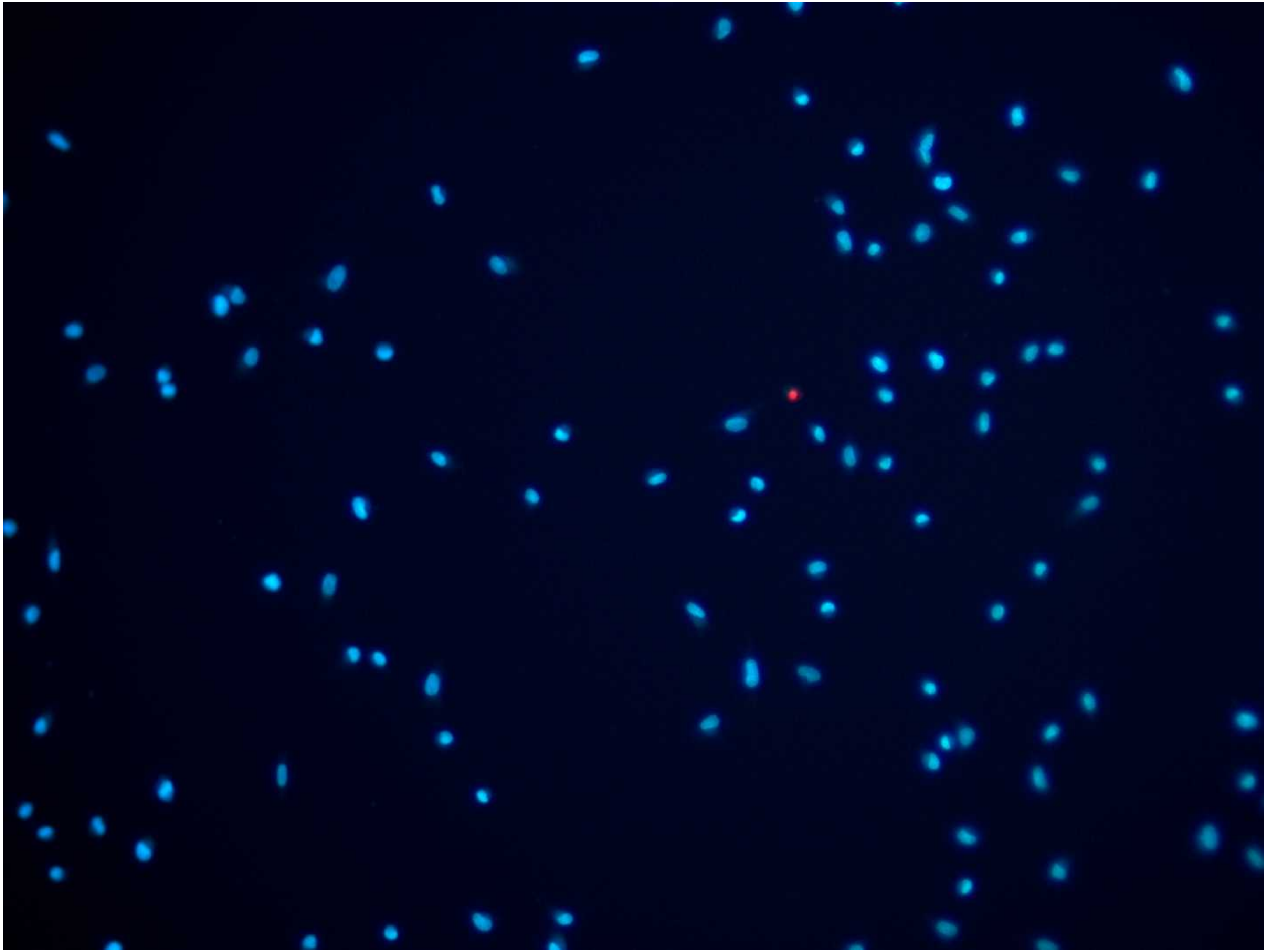

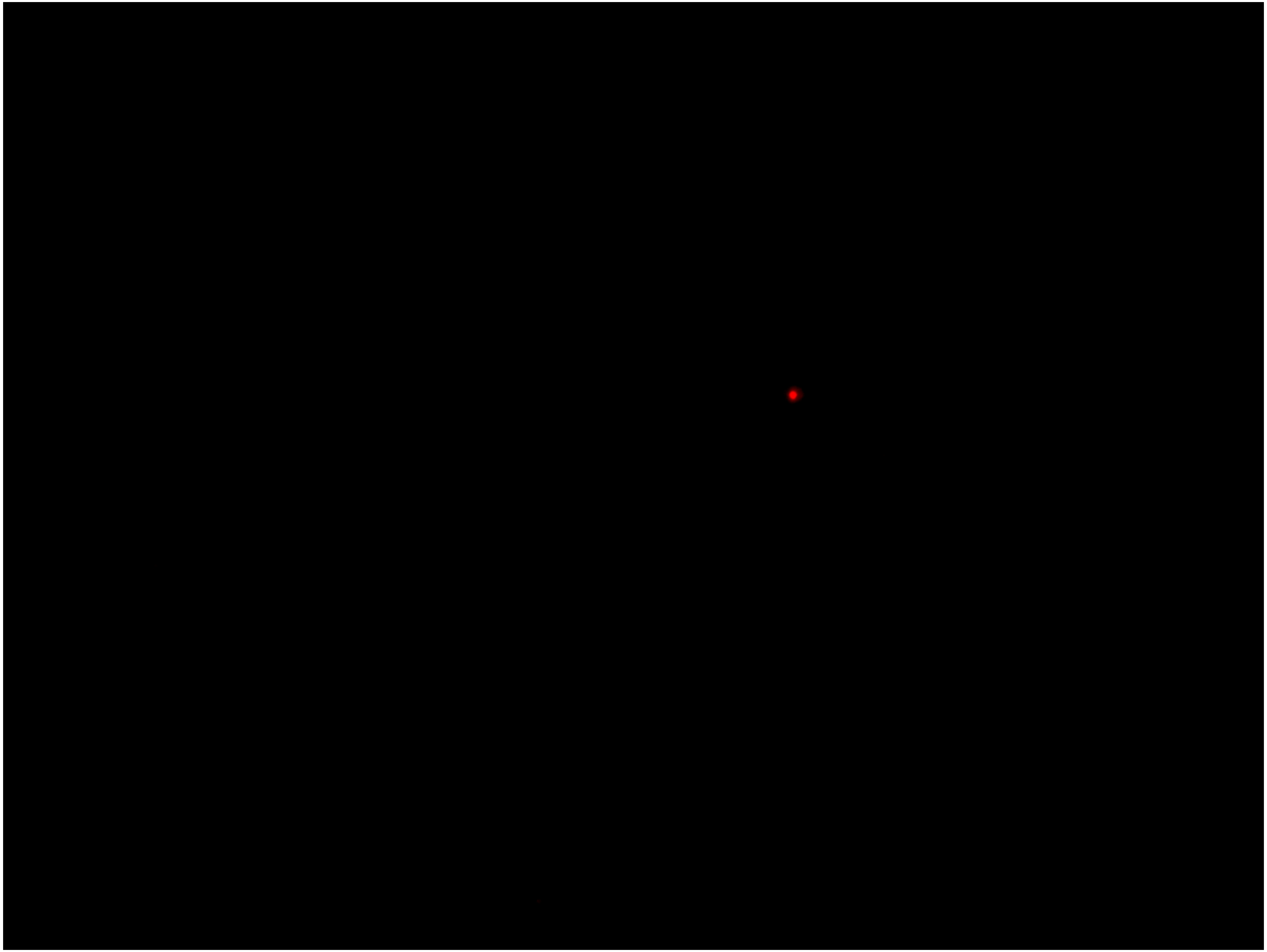

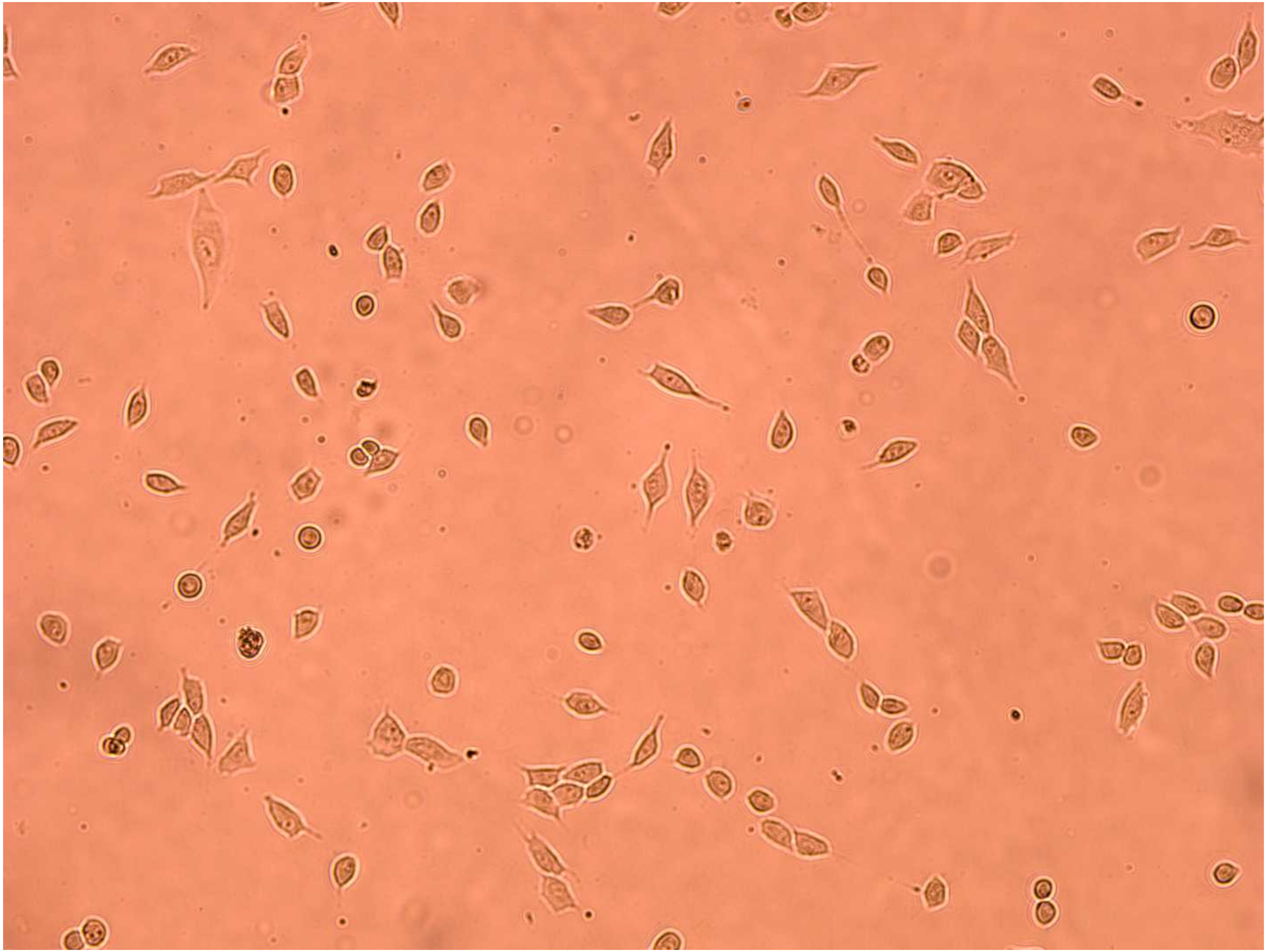

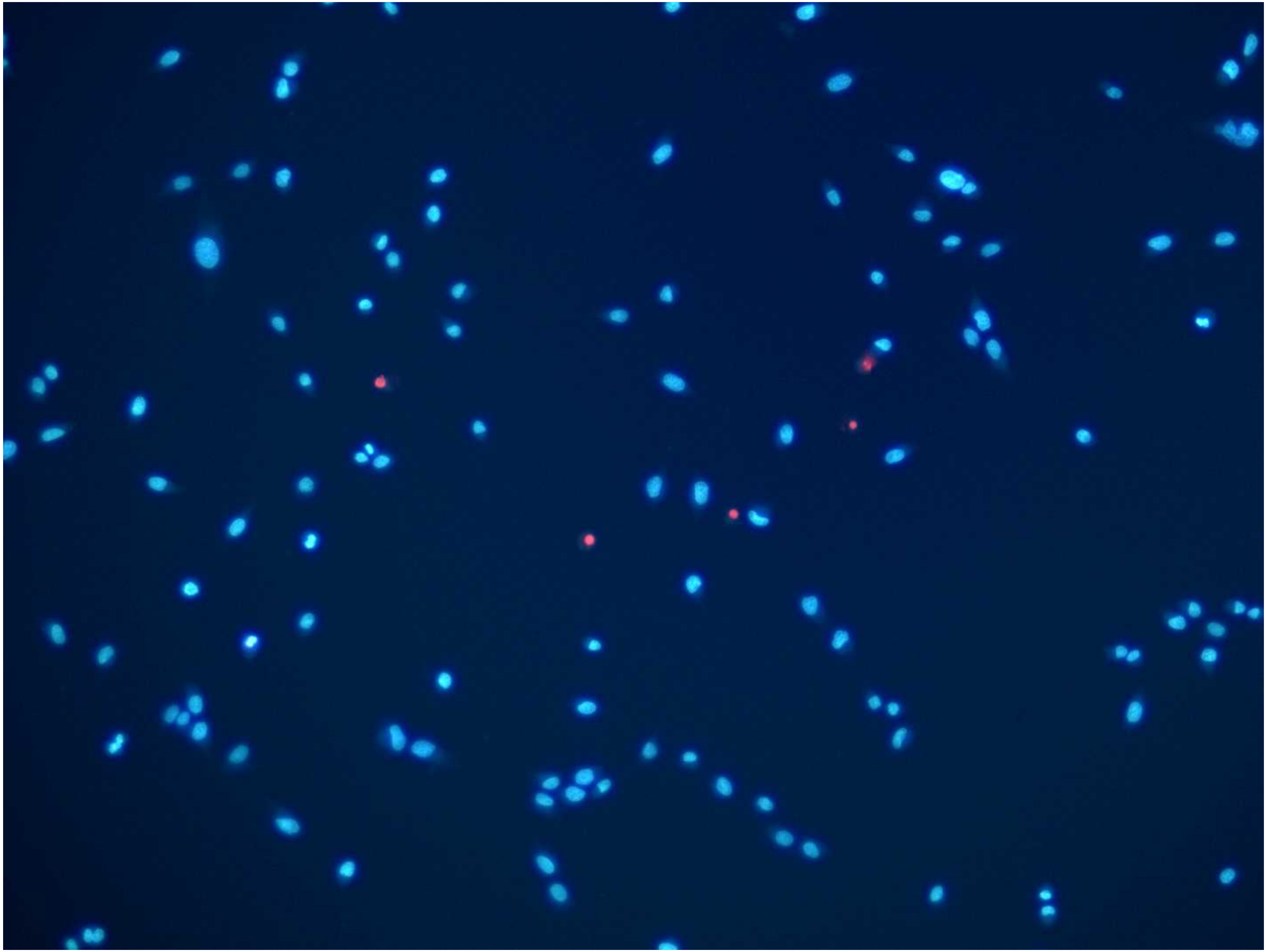

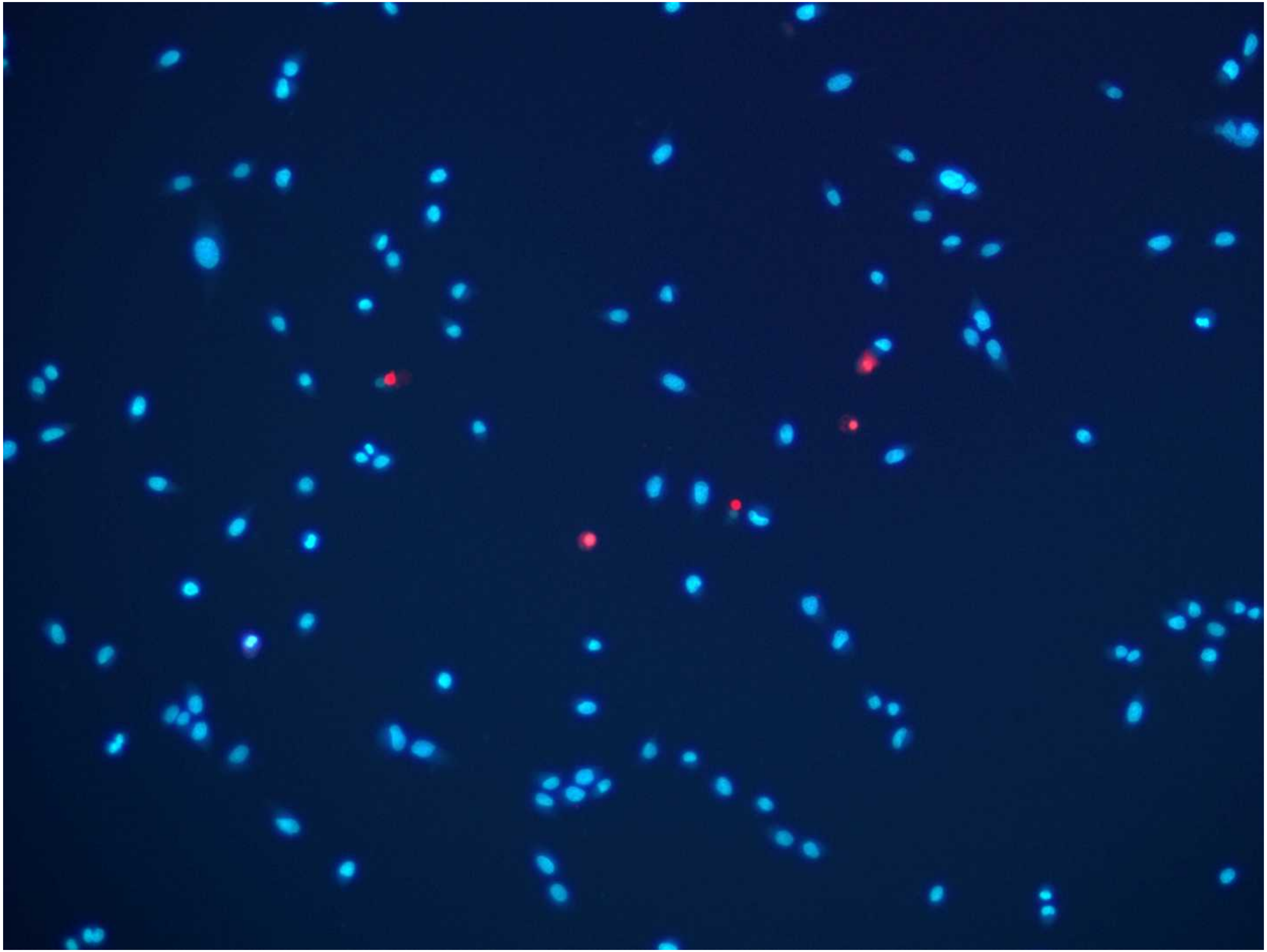

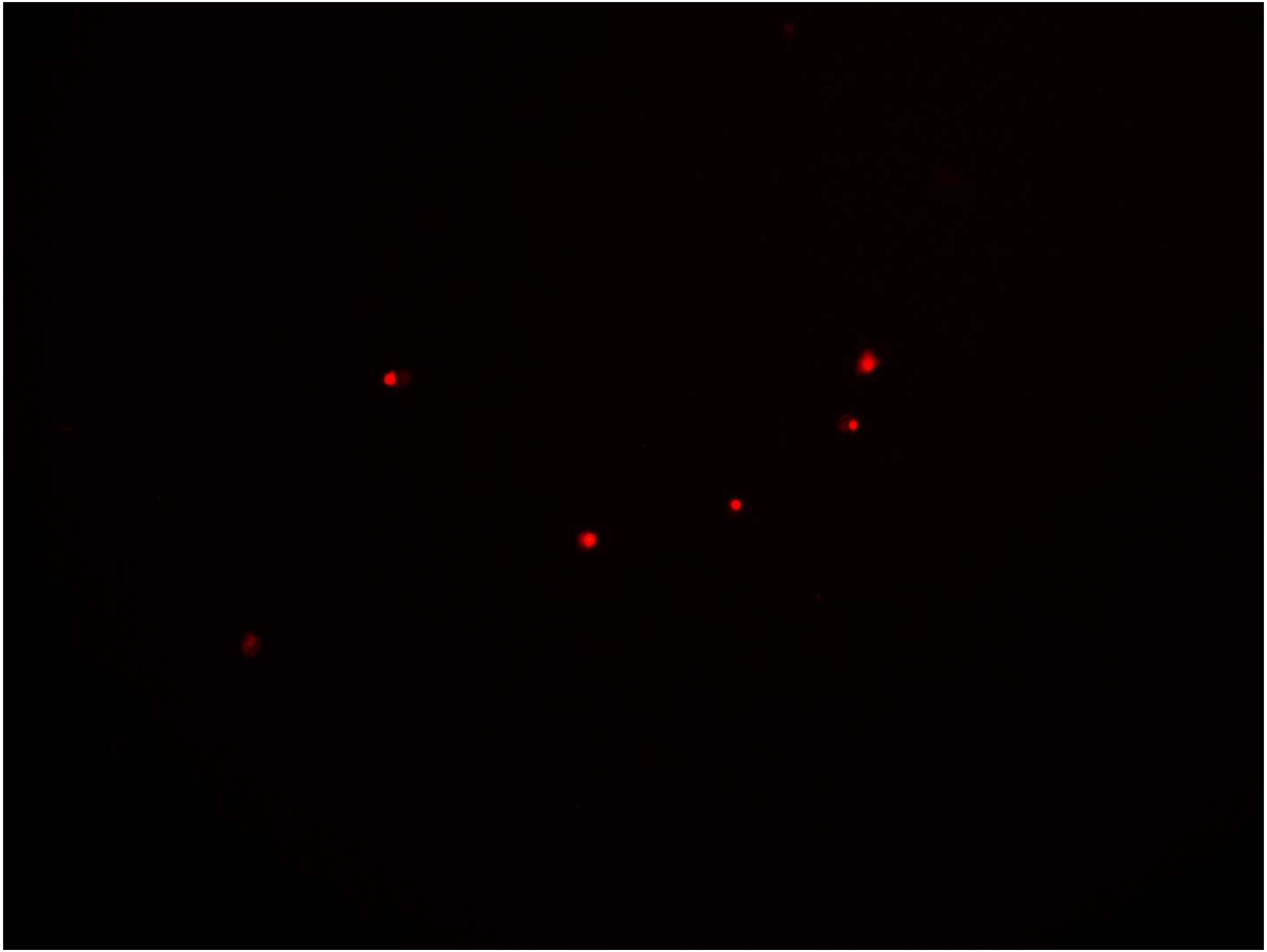

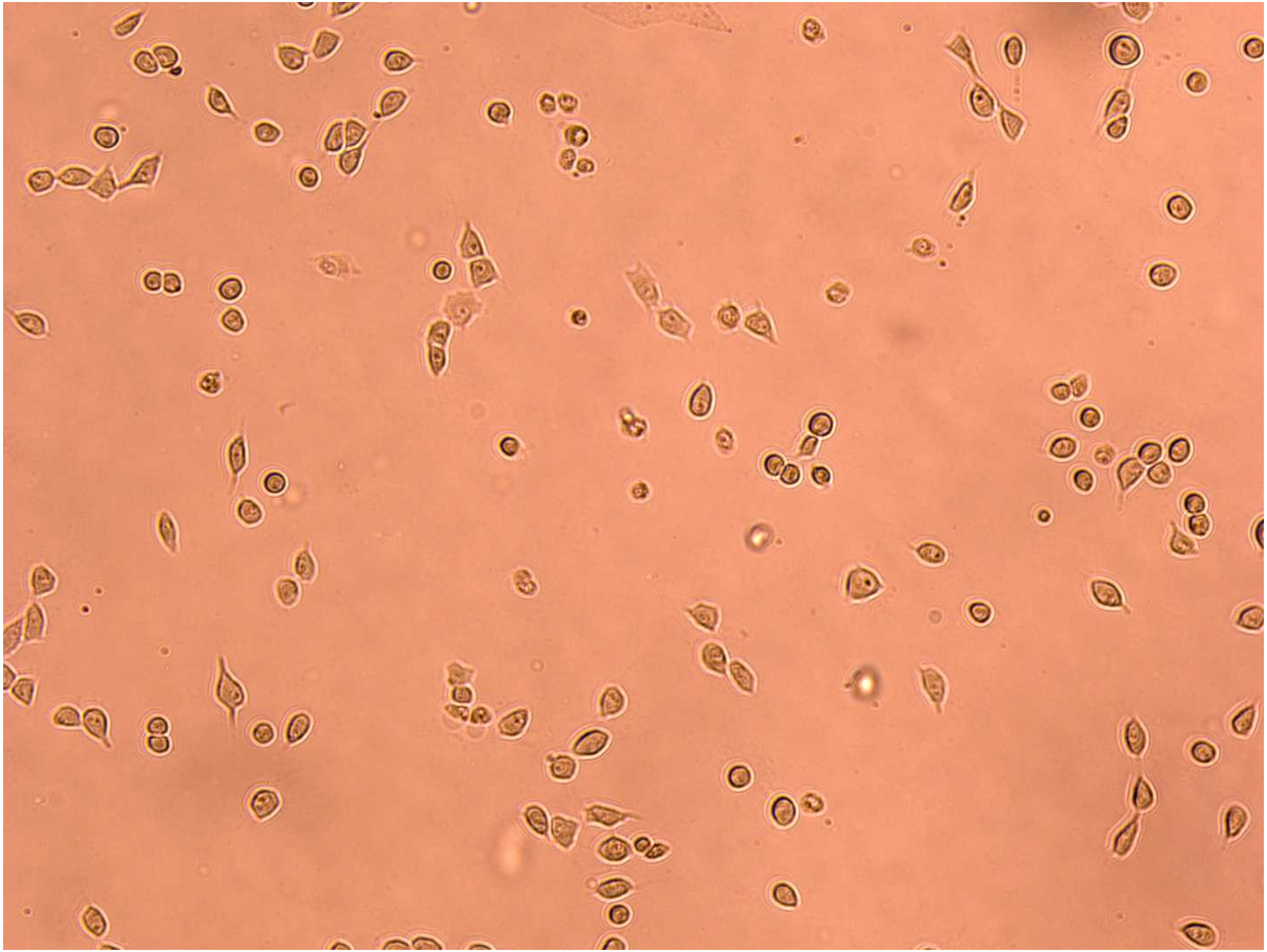

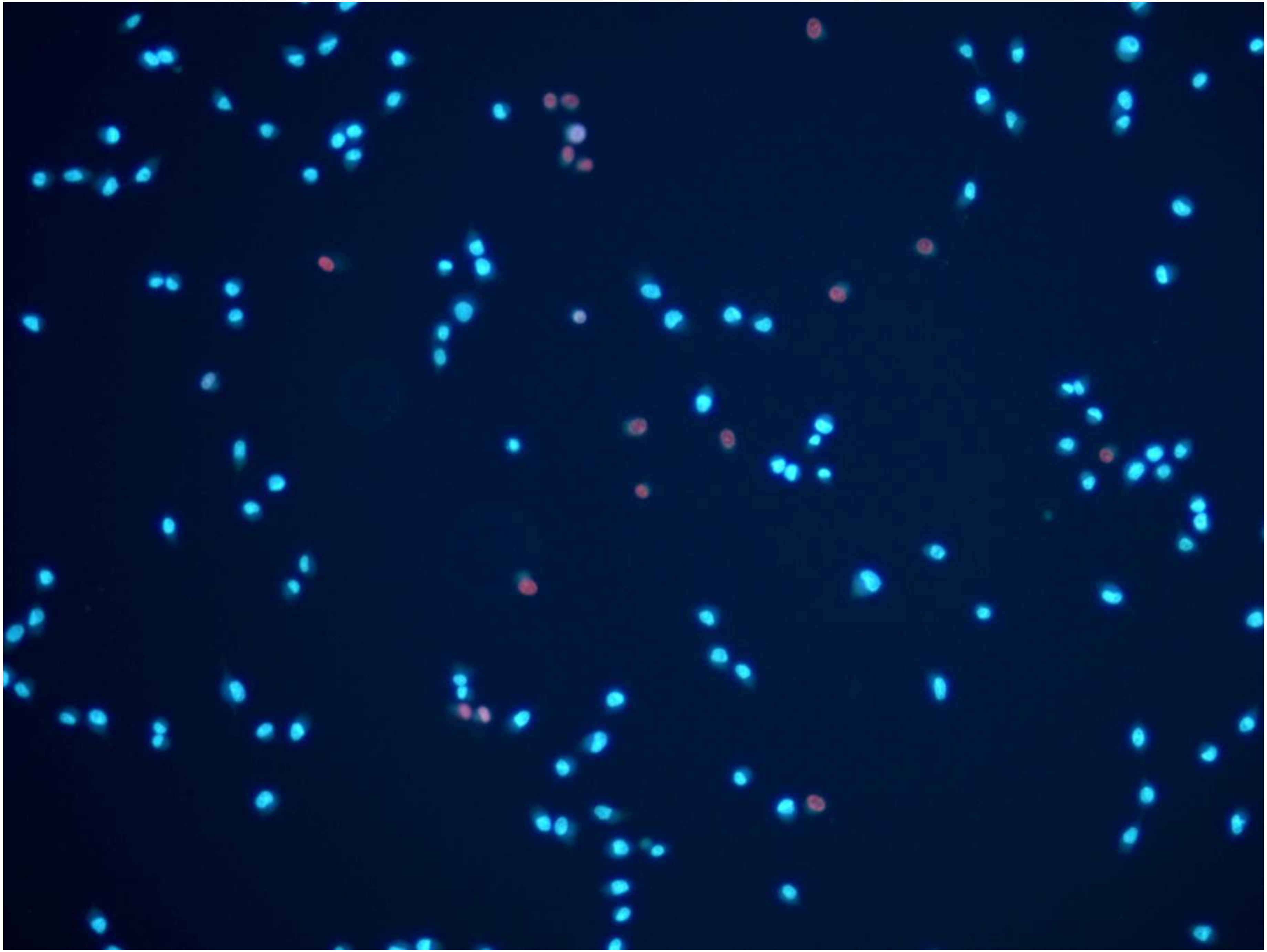

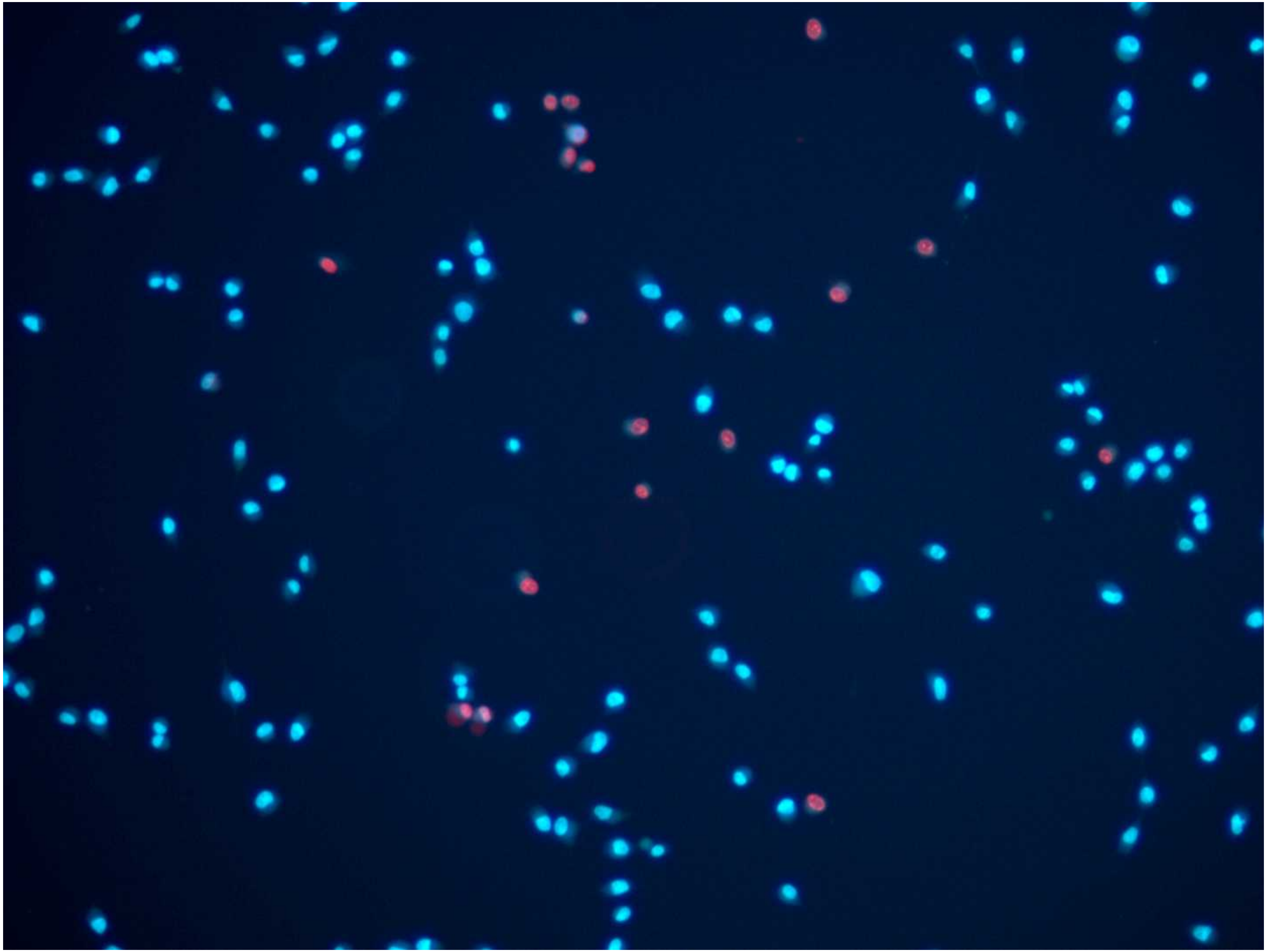

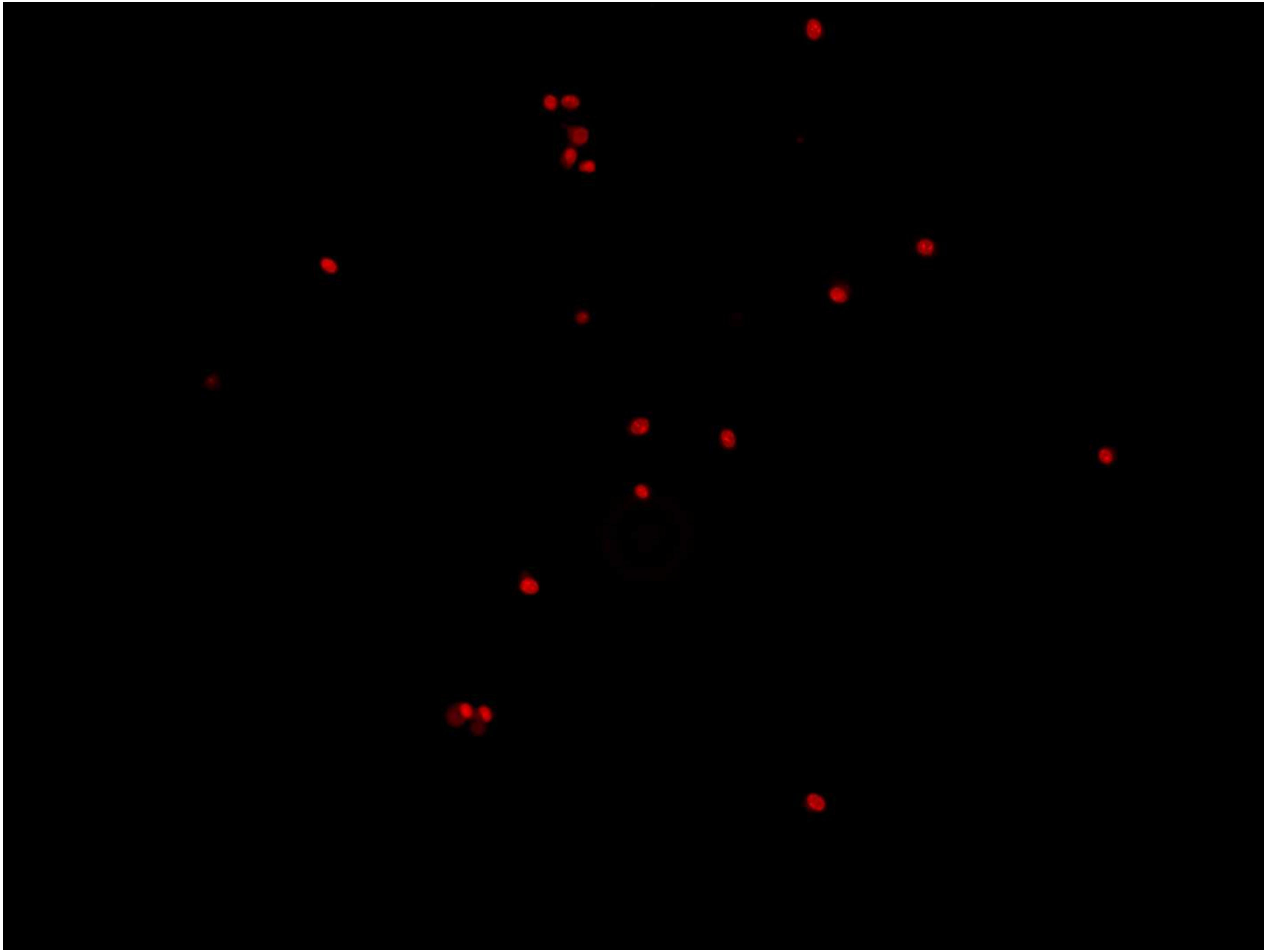

MG63 仙灵

1.NC 2.HG 3.HG+仙灵

凋亡蛋白

Bcl-2

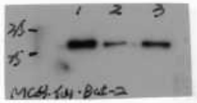

Bax

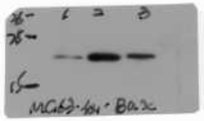

Active Caspase3 p17

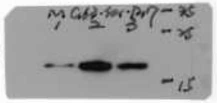

GAPDH

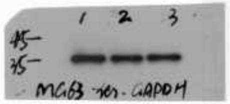

周期蛋白

CDK4

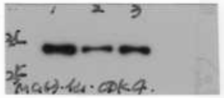

CDK6

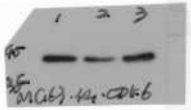

CyclinD1

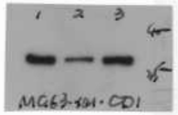

MKSAP1

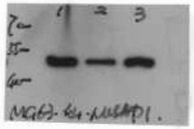

GAPDH

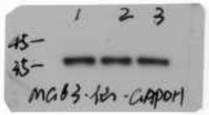

PI3K/AKT通路

AKT

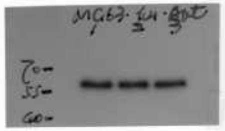

p-AKT

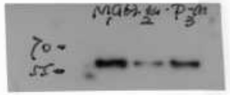

P70

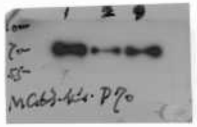

GAPDH

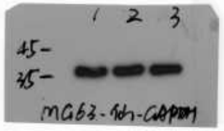

MG63-仙灵2

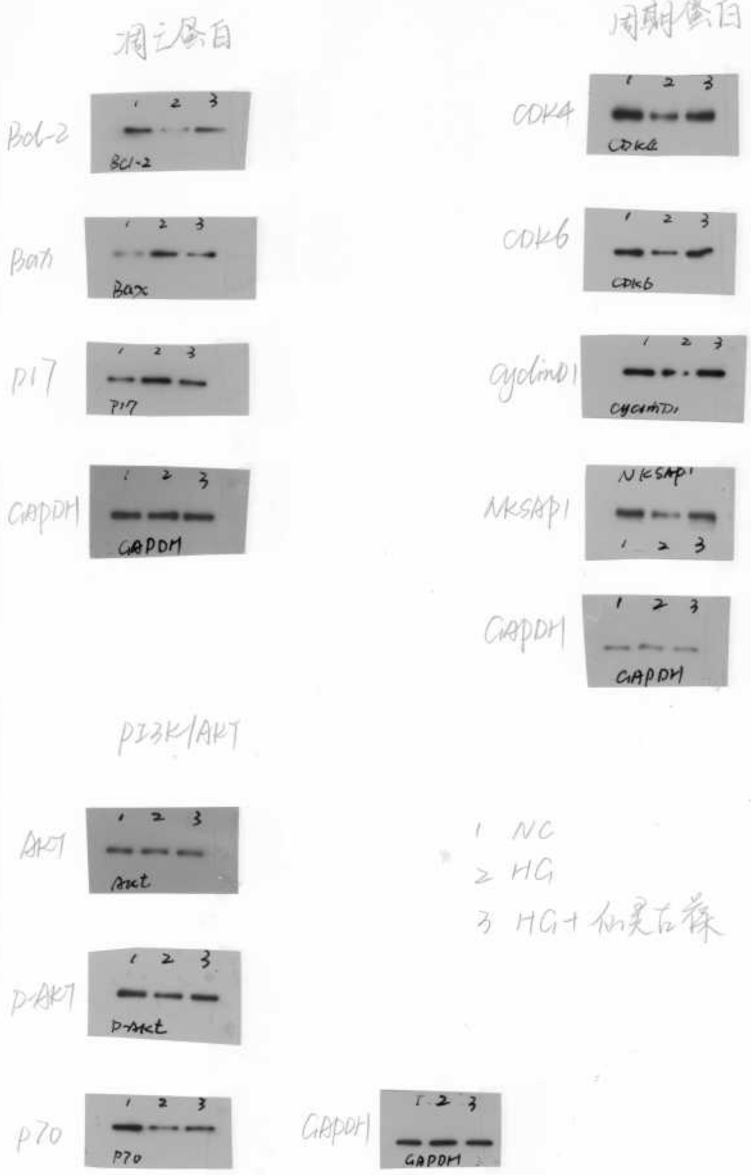

凋亡蛋白
周期蛋白
Bcl-2
Bax
P17
GAPDH
CDK4
CDK6
cyclinD1
NKSAP1
GAPDH
PI3K/AKT
AKT
P-AKT
P70
GAPDH
1 NC
2 HG
3 HG+仙灵骨葆

MG63-仙灵3

凋亡蛋白

Bcl-2
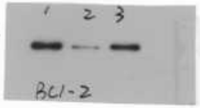

Bax
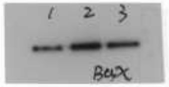

P17
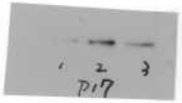

GAPDH
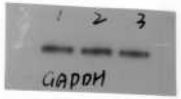

周期蛋白

CDK4
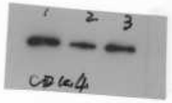

CDK6
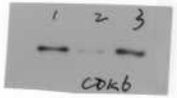

CyclinD1
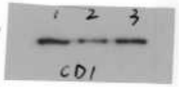

NKSAP1
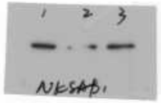

GAPDH
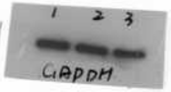

PI3K/AKT

AKT
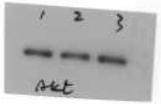

P-AKT
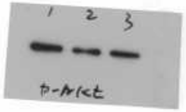

P70
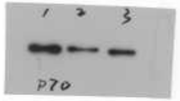

GAPDH
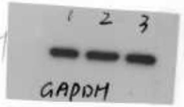

1 NC
2 HG
3 HG+仙灵古葆

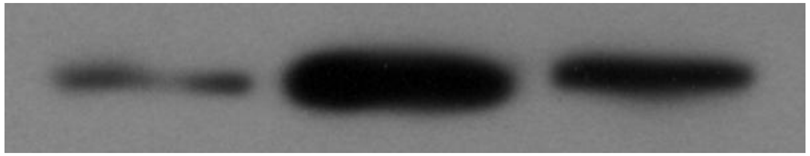

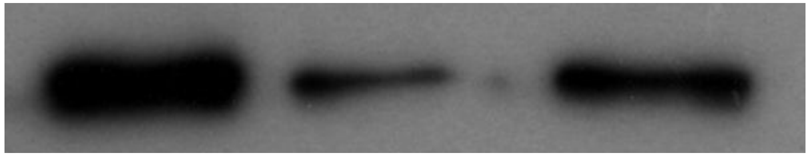

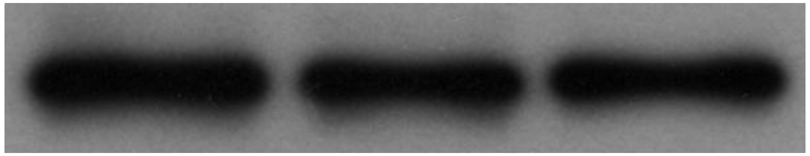

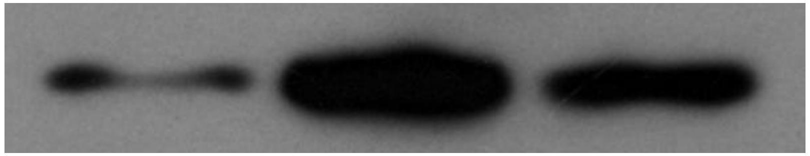

MG63 仙灵

1.NC 2.HG 3.HG+仙灵

凋亡蛋白

Bcl-2

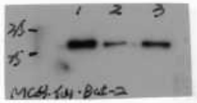

Bax

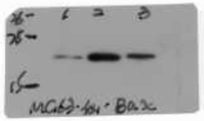

Active Caspase3 p17

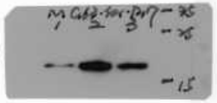

GAPDH

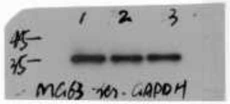

周期蛋白

CDK4

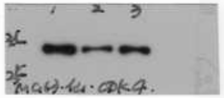

CDK6

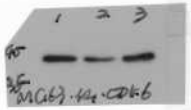

CyclinD1

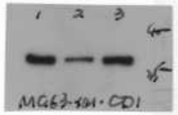

MKSAP1

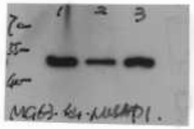

GAPDH

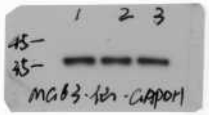

PI3K/AKT通路

AKT

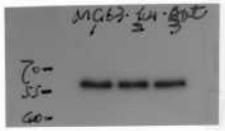

p-AKT

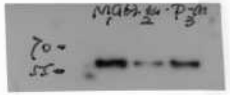

P70

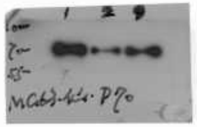

GAPDH

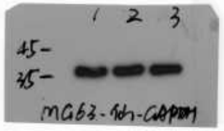

MG63-仙灵2

凋亡蛋白

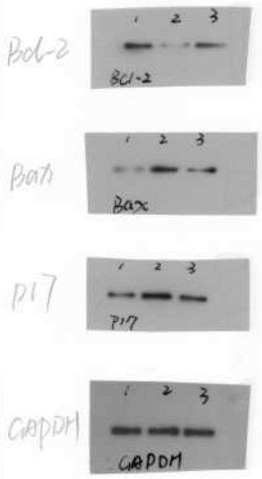

周期蛋白

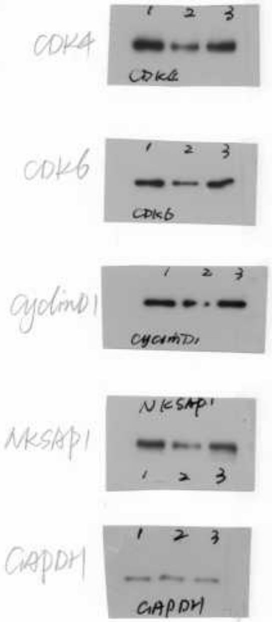

PI3K/AKT

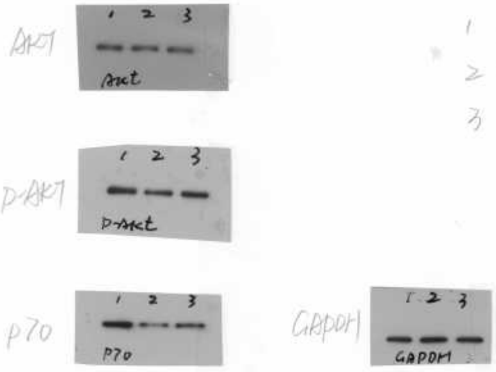

1 NC
2 HG
3 HG+仙灵石葆

MG63-仙灵3

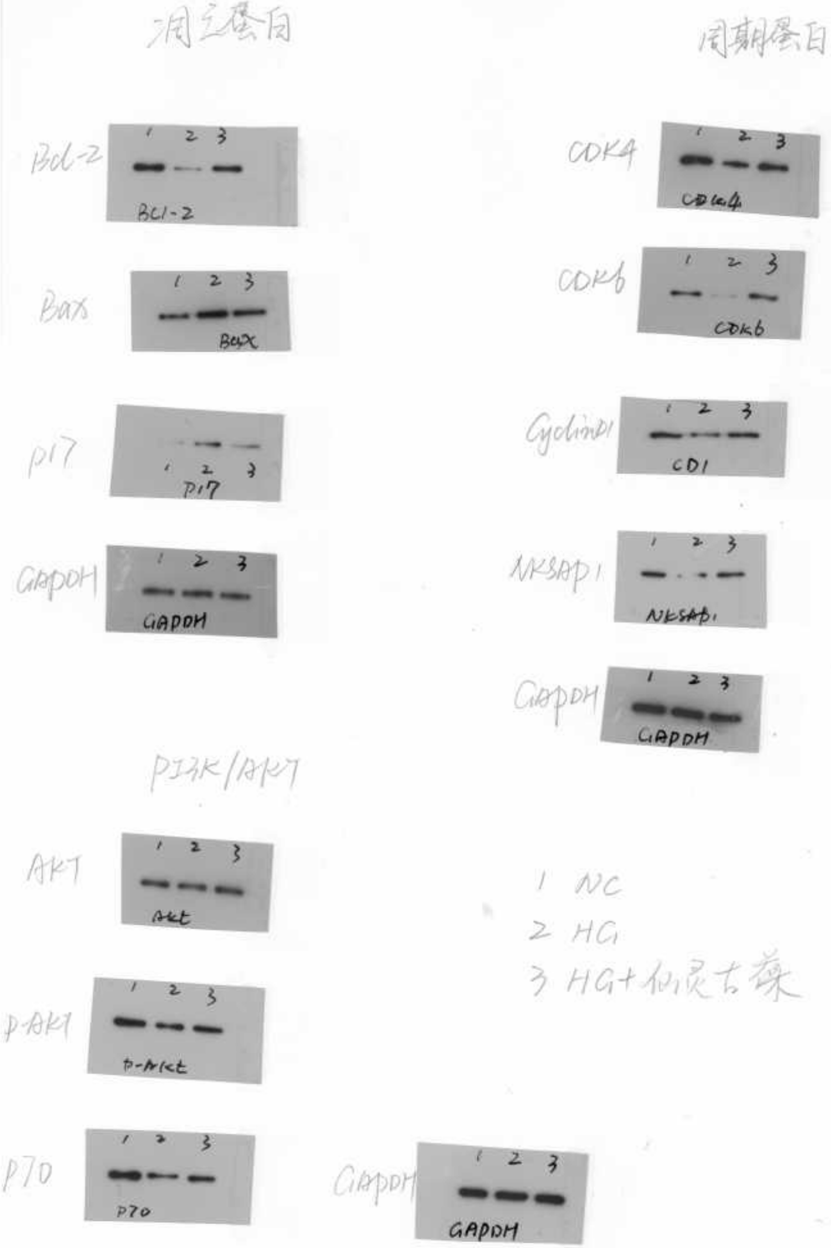

1 NC
2 HG
3 HG+仙灵古葆

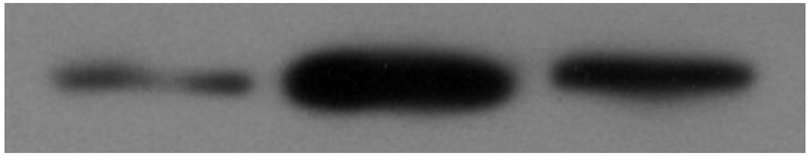

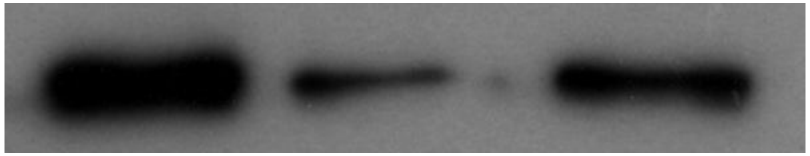

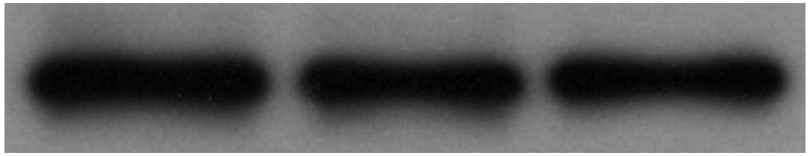

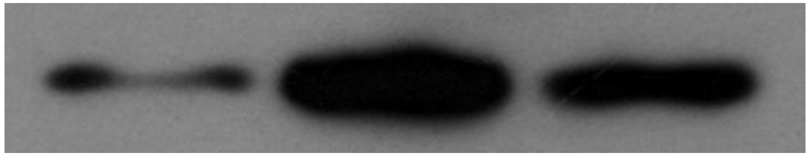

ALP

	Control					Glucose		
0.834	0.756	0.935			0.272	0.367	0.292	
	Control		**Mean**	**SD**		Glucose		**Mean**
1	0.90647	1.1211	**1.009193**	**0.107609**	0.326139	0.440048	0.35012	**0.372102**

OCN

	Control					Glucose		
0.568	0.571	0.62			0.248	0.202	0.265	
	Control		**Mean**	**SD**		Glucose		**Mean**
1	1.00528	1.09155	**1.032277**	**0.0513992**	0.43662	0.355634	0.466549	**0.419601**

OPN

	Control					Glucose		
0.793	0.856	0.733			0.24	0.311	0.283	
	Control		**Mean**	**SD**		Glucose		**Mean**
1	1.07945	0.92434	**1.001261**	**0.0775613**	0.302648	0.392182	0.356873	**0.350567**

RUNX2

	Control					Glucose		
0.675	0.767	0.605			0.263	0.329	0.356	
	Control		**Mean**	**SD**		Glucose		**Mean**
1	1.1363	0.8963	**1.010864**	**0.1203683**	0.38963	0.487407	0.527407	**0.468148**

OPG

	Control					Glucose		
0.825	0.791	0.855			0.23	0.237	0.181	
	Control		**Mean**	**SD**		Glucose		**Mean**
1	0.95879	1.03636	**0.998384**	**0.0388131**	0.278788	0.287273	0.219394	**0.261818**

OPGL

	Control					Glucose		
0.583	0.536	0.629			0.934	1.107	1.11	
	Control		**Mean**	**SD**		Glucose		**Mean**
1	0.91938	1.0789	**0.999428**	**0.0797614**	1.602058	1.898799	1.903945	**1.801601**

	Glucose+XLGB				
	0.764	0.685	0.657		
**SD**	Glucose+XLGB			**Mean**	**SD**
**0.06005**	0.9160671	0.82134293	0.7878	**0.8417**	**0.066533**
	Glucose+XLGB				
	0.488	0.543	0.566		
**SD**	Glucose+XLGB			**Mean**	**SD**
**0.05738**	0.8591549	0.95598592	0.9965	**0.9372**	**0.070562**
	Glucose+XLGB				
	0.648	0.68	0.587		
**SD**	Glucose+XLGB			**Mean**	**SD**
**0.0451**	0.8171501	0.85750315	0.7402	**0.805**	**0.059581**
	Glucose+XLGB				
	0.499	0.562	0.546		
**SD**	Glucose+XLGB			**Mean**	**SD**
**0.07088**	0.7392593	0.83259259	0.8089	**0.7936**	**0.048513**
	Glucose+XLGB				
	0.683	0.629	0.796		
**SD**	Glucose+XLGB			**Mean**	**SD**
**0.03698**	0.8278788	0.76242424	0.9648	**0.8517**	**0.103296**
	Glucose+XLGB				
	0.547	0.674	0.653		
**SD**	Glucose+XLGB			**Mean**	**SD**
**0.17283**	0.9382504	1.15608919	1.1201	**1.0715**	**0.116768**

	Control					Glucose	
0.568	0.571	0.62			0.248	0.202	0.265

	Control		**Mean**	**SD**		Glucose	
1	1.005282	1.091549	**1.03228**	**0.0514**	0.43662	0.355634	0.466549

	Glucose+XLGB	
0.488	0.543	0.566

Mean	SD		Glucose+XLGB		Mean	SD
**0.4196**	**0.05738**	0.859155	0.955986	0.996479	**0.93721**	**0.07056**

	Control					Glucose	
0.793	0.856	0.733			0.24	0.311	0.283

	Control		**Mean**	**SD**		Glucose	
1	1.079445	0.924338	**1.00126**	**0.07756**	0.302648	0.392182	0.356873

			Glucose+XLGB				
		0.648	0.68	0.587			
**Mean**	**SD**		Glucose+XLGB		**Mean**	**SD**
**0.35057**	**0.0451**	0.81715	0.857503	0.740227	**0.80496**	**0.05958**

	Control					Glucose	
0.675	0.767	0.605			0.263	0.329	0.356

	Control		**Mean**	**SD**		Glucose	
1	1.136296	0.896296	**1.01086**	**0.12037**	0.38963	0.487407	0.527407

	Glucose+XLGB	
0.499	0.562	0.546

Mean	SD		Glucose+XLGB		Mean	SD
**0.46815**	**0.07088**	0.739259	0.832593	0.808889	**0.79358**	**0.04851**

	Control					Glucose	
0.825	0.791	0.855			0.23	0.237	0.181
	Control		**Mean**	**SD**		Glucose	
1	0.958788	1.036364	**0.99838**	**0.03881**	0.278788	0.287273	0.219394

			Glucose+XLGB					
		0.683	0.629	0.796				
**Mean**	**SD**		Glucose+XLGB			**Mean**	**SD**	
**0.26182**	**0.03698**	0.827879	0.762424	0.964848	**0.85172**	**0.1033**		

	Control					Glucose	
0.583	0.536	0.629			0.934	1.107	1.11

	Control		**Mean**	**SD**		Glucose	
1	0.919383	1.078902	**0.99943**	**0.07976**	1.602058	1.898799	1.903945

			Glucose+XLGB				
		0.547	0.674	0.653			

**Mean**	**SD**		Glucose+XLGB		**Mean**	**SD**
**1.8016**	**0.17283**	0.93825	1.156089	1.120069	**1.07147**	**0.11677**

Experiment Information

Name	P_200615
Date	43997
Operator	admin
Location	F:\Funglyn\FTC-3000\experiments\admin
Template	F:\Funglyn\FTC-3000\templates\SYBR Green 两步法.tmt
Tips	

Protocol

Stage:Hold Cycles:1

Target	Hold(H:M:S)	Ramp Rate(C/s)	Touch Down	Multiple Temp	Singnal Acq.
95	0.416666667				

Stage:Cycles Cycles:40

Target	Hold(H:M:S)	Ramp Rate(C/s)	Touch Down	Multiple Temp	Singnal Acq.
95	0.010416667				
60	0.041666667				TRUE

Stage:Dissociation

Target	Hold(H:M:S)	Ramp Rate(C/s)	Touch Down	Multiple Temp	Singnal Acq.
94	0.0625				
60	0.125	0.1			
94	0.006944444				TRUE

Plate

Well	Reporter	Subset ID	Sample Name	Gene	Sample Type	Quantity
A2	SYBR GREEN	S I	CONTROL	OCN	Unknown Sample	
A3	SYBR GREEN	S I	CONTROL	OCN	Unknown Sample	
A4	SYBR GREEN	S I	CONTROL	OCN	Unknown Sample	
A5	SYBR GREEN	S I	GLUCOSE	OCN	Unknown Sample	
A6	SYBR GREEN	S I	GLUCOSE	OCN	Unknown Sample	
A7	SYBR GREEN	S I	GLUCOSE	OCN	Unknown Sample	
A8	SYBR GREEN	S I	GLUCOSE+XLGB	OCN	Unknown Sample	
A9	SYBR GREEN	S I	GLUCOSE+XLGB	OCN	Unknown Sample	
A10	SYBR GREEN	S I	GLUCOSE+XLGB	OCN	Unknown Sample	
B2	SYBR GREEN	S I	CONTROL	RUNX2	Unknown Sample	
B3	SYBR GREEN	S I	CONTROL	RUNX2	Unknown Sample	
B4	SYBR GREEN	S I	CONTROL	RUNX2	Unknown Sample	
B5	SYBR GREEN	S I	GLUCOSE	RUNX2	Unknown Sample	
B6	SYBR GREEN	S I	GLUCOSE	RUNX2	Unknown Sample	
B7	SYBR GREEN	S I	GLUCOSE	RUNX2	Unknown Sample	
B8	SYBR GREEN	S I	GLUCOSE+XLGB	RUNX2	Unknown Sample	
B9	SYBR GREEN	S I	GLUCOSE+XLGB	RUNX2	Unknown Sample	
B10	SYBR GREEN	S I	GLUCOSE+XLGB	RUNX2	Unknown Sample	
C2	SYBR GREEN	S I	CONTROL	actin	Unknown Sample	
C3	SYBR GREEN	S I	CONTROL	actin	Unknown Sample	
C4	SYBR GREEN	S I	CONTROL	actin	Unknown Sample	
C5	SYBR GREEN	S I	GLUCOSE	actin	Unknown Sample	
C6	SYBR GREEN	S I	GLUCOSE	actin	Unknown Sample	
C7	SYBR GREEN	S I	GLUCOSE	actin	Unknown Sample	
C8	SYBR GREEN	S I	GLUCOSE+XLGB	actin	Unknown Sample	
C9	SYBR GREEN	S I	GLUCOSE+XLGB	actin	Unknown Sample	
C10	SYBR GREEN	S I	GLUCOSE+XLGB	actin	Unknown Sample	
D2	SYBR GREEN	S I	CONTROL	ALP	Unknown Sample	

D3	SYBR GREEN	S I	CONTROL	ALP	Unknown Sample
D4	SYBR GREEN	S I	CONTROL	ALP	Unknown Sample
D5	SYBR GREEN	S I	GLUCOSE	ALP	Unknown Sample
D6	SYBR GREEN	S I	GLUCOSE	ALP	Unknown Sample
D7	SYBR GREEN	S I	GLUCOSE	ALP	Unknown Sample
D8	SYBR GREEN	S I	GLUCOSE+XLGB	ALP	Unknown Sample
D9	SYBR GREEN	S I	GLUCOSE+XLGB	ALP	Unknown Sample
D10	SYBR GREEN	S I	GLUCOSE+XLGB	ALP	Unknown Sample
E2	SYBR GREEN	S I	CONTROL	OPN	Unknown Sample
E3	SYBR GREEN	S I	CONTROL	OPN	Unknown Sample
E4	SYBR GREEN	S I	CONTROL	OPN	Unknown Sample
E5	SYBR GREEN	S I	GLUCOSE	OPN	Unknown Sample
E6	SYBR GREEN	S I	GLUCOSE	OPN	Unknown Sample
E7	SYBR GREEN	S I	GLUCOSE	OPN	Unknown Sample
E8	SYBR GREEN	S I	GLUCOSE+XLGB	OPN	Unknown Sample
E9	SYBR GREEN	S I	GLUCOSE+XLGB	OPN	Unknown Sample
E10	SYBR GREEN	S I	GLUCOSE+XLGB	OPN	Unknown Sample

Data

Well	Subset ID	Reporter	S_Name	S_Type	Ct	Mean Ct
A2	S I	SYBR GREEN	CONTROL	Unknown Sample	18.71	18.64
A3	S I	SYBR GREEN	CONTROL	Unknown Sample	18.64	18.64
A4	S I	SYBR GREEN	CONTROL	Unknown Sample	18.57	18.64
A5	S I	SYBR GREEN	GLUCOSE	Unknown Sample	19.11	19.03
A6	S I	SYBR GREEN	GLUCOSE	Unknown Sample	18.87	19.03
A7	S I	SYBR GREEN	GLUCOSE	Unknown Sample	19.12	19.03
A8	S I	SYBR GREEN	GLUCOSE+XLGB	Unknown Sample	18.4	18.32
A9	S I	SYBR GREEN	GLUCOSE+XLGB	Unknown Sample	18.36	18.32
A10	S I	SYBR GREEN	GLUCOSE+XLGB	Unknown Sample	18.21	18.32
B2	S I	SYBR GREEN	CONTROL	Unknown Sample	17.66	17.78
B3	S I	SYBR GREEN	CONTROL	Unknown Sample	17.73	17.78
B4	S I	SYBR GREEN	CONTROL	Unknown Sample	17.94	17.78
B5	S I	SYBR GREEN	GLUCOSE	Unknown Sample	17.82	17.82
B6	S I	SYBR GREEN	GLUCOSE	Unknown Sample	18.01	17.82
B7	S I	SYBR GREEN	GLUCOSE	Unknown Sample	17.64	17.82
B8	S I	SYBR GREEN	GLUCOSE+XLGB	Unknown Sample	17.41	17.45
B9	S I	SYBR GREEN	GLUCOSE+XLGB	Unknown Sample	17.37	17.45
B10	S I	SYBR GREEN	GLUCOSE+XLGB	Unknown Sample	17.56	17.45
C2	S I	SYBR GREEN	CONTROL	Unknown Sample	12.13	12.18
C3	S I	SYBR GREEN	CONTROL	Unknown Sample	12.12	12.18
C4	S I	SYBR GREEN	CONTROL	Unknown Sample	12.3	12.18
C5	S I	SYBR GREEN	GLUCOSE	Unknown Sample	11.11	11.27
C6	S I	SYBR GREEN	GLUCOSE	Unknown Sample	11.52	11.27
C7	S I	SYBR GREEN	GLUCOSE	Unknown Sample	11.17	11.27
C8	S I	SYBR GREEN	GLUCOSE+XLGB	Unknown Sample	11.98	11.75
C9	S I	SYBR GREEN	GLUCOSE+XLGB	Unknown Sample	11.8	11.75
C10	S I	SYBR GREEN	GLUCOSE+XLGB	Unknown Sample	11.46	11.75
D2	S I	SYBR GREEN	CONTROL	Unknown Sample	24.11	24.13
D3	S I	SYBR GREEN	CONTROL	Unknown Sample	24.03	24.13
D4	S I	SYBR GREEN	CONTROL	Unknown Sample	24.24	24.13
D5	S I	SYBR GREEN	GLUCOSE	Unknown Sample	24.61	24.53
D6	S I	SYBR GREEN	GLUCOSE	Unknown Sample	24.66	24.53
D7	S I	SYBR GREEN	GLUCOSE	Unknown Sample	24.32	24.53
D8	S I	SYBR GREEN	GLUCOSE+XLGB	Unknown Sample	23.84	23.77
D9	S I	SYBR GREEN	GLUCOSE+XLGB	Unknown Sample	23.82	23.77
D10	S I	SYBR GREEN	GLUCOSE+XLGB	Unknown Sample	23.64	23.77
E2	S I	SYBR GREEN	CONTROL	Unknown Sample	21.49	21.6
E3	S I	SYBR GREEN	CONTROL	Unknown Sample	21.69	21.6
E4	S I	SYBR GREEN	CONTROL	Unknown Sample	21.62	21.6
E5	S I	SYBR GREEN	GLUCOSE	Unknown Sample	22.14	22.14
E6	S I	SYBR GREEN	GLUCOSE	Unknown Sample	22.28	22.14
E7	S I	SYBR GREEN	GLUCOSE	Unknown Sample	22.01	22.14
E8	S I	SYBR GREEN	GLUCOSE+XLGB	Unknown Sample	20.98	20.94
E9	S I	SYBR GREEN	GLUCOSE+XLGB	Unknown Sample	20.87	20.94
E10	S I	SYBR GREEN	GLUCOSE+XLGB	Unknown Sample	20.95	20.94

Graph

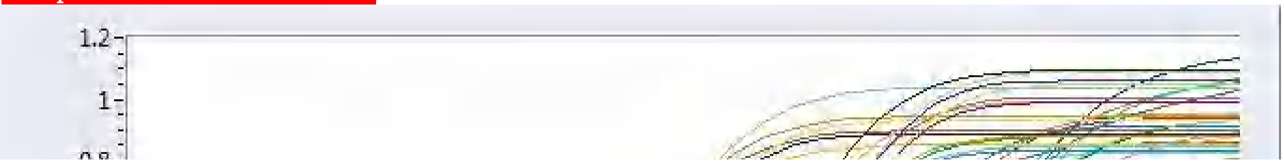

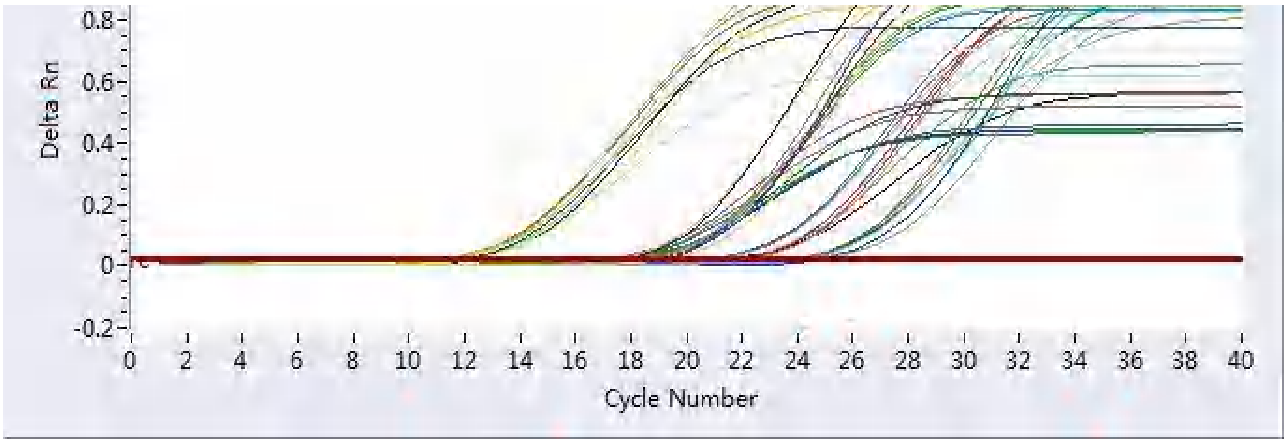

Delta Rn
0.8
0.6
0.4
0.2
0
-0.2
0 2 4 6 8 10 12 14 16 18 20 22 24 26 28 30 32 34 36 38 40
Cycle Number

StdDev Ct	Quantity	Gene
0.07	-	OCN
0.07	-	OCN
0.07	-	OCN
0.14	-	OCN
0.14	-	OCN
0.14	-	OCN
0.1	-	OCN
0.1	-	OCN
0.1	-	OCN
0.15	-	RUNX2
0.15	-	RUNX2
0.15	-	RUNX2
0.19	-	RUNX2
0.19	-	RUNX2
0.19	-	RUNX2
0.1	-	RUNX2
0.1	-	RUNX2
0.1	-	RUNX2
0.1	-	actin
0.1	-	actin
0.1	-	actin
0.22	-	actin
0.22	-	actin
0.22	-	actin
0.27	-	actin
0.27	-	actin
0.27	-	actin
0.1	-	ALP
0.1	-	ALP
0.1	-	ALP
0.18	-	ALP
0.18	-	ALP
0.18	-	ALP
0.11	-	ALP
0.11	-	ALP
0.11	-	ALP
0.1	-	OPN
0.1	-	OPN
0.1	-	OPN
0.14	-	OPN
0.14	-	OPN
0.14	-	OPN
0.06	-	OPN
0.06	-	OPN
0.06	-	OPN

Data

Well	Subset Id	Reporter	Sample Name	Gene	Tm1	Tm2
A2	S I	SYBR GREEN	CONTROL	OCN	80.38	-
A3	S I	SYBR GREEN	CONTROL	OCN	79.66	-
A4	S I	SYBR GREEN	CONTROL	OCN	79.86	-
A5	S I	SYBR GREEN	GLUCOSE	OCN	80.03	-
A6	S I	SYBR GREEN	GLUCOSE	OCN	80.06	-
A7	S I	SYBR GREEN	GLUCOSE	OCN	80.17	-
A8	S I	SYBR GREEN	GLUCOSE+XLGB	OCN	79.62	-
A9	S I	SYBR GREEN	GLUCOSE+XLGB	OCN	79.48	-
A10	S I	SYBR GREEN	GLUCOSE+XLGB	OCN	79.65	-
B2	S I	SYBR GREEN	CONTROL	RUNX2	79.67	-
B3	S I	SYBR GREEN	CONTROL	RUNX2	79.75	-
B4	S I	SYBR GREEN	CONTROL	RUNX2	79.79	-
B5	S I	SYBR GREEN	GLUCOSE	RUNX2	79.77	-
B6	S I	SYBR GREEN	GLUCOSE	RUNX2	78.96	-
B7	S I	SYBR GREEN	GLUCOSE	RUNX2	79.16	-
B8	S I	SYBR GREEN	GLUCOSE+XLGB	RUNX2	79.27	-
B9	S I	SYBR GREEN	GLUCOSE+XLGB	RUNX2	78.99	-
B10	S I	SYBR GREEN	GLUCOSE+XLGB	RUNX2	78.86	-
C2	S I	SYBR GREEN	CONTROL	actin	83.73	-
C3	S I	SYBR GREEN	CONTROL	actin	83.87	-
C4	S I	SYBR GREEN	CONTROL	actin	83.92	-
C5	S I	SYBR GREEN	GLUCOSE	actin	-	-
C6	S I	SYBR GREEN	GLUCOSE	actin	84.06	-
C7	S I	SYBR GREEN	GLUCOSE	actin	82.96	-
C8	S I	SYBR GREEN	GLUCOSE+XLGB	actin	83.25	-
C9	S I	SYBR GREEN	GLUCOSE+XLGB	actin	83.22	-
C10	S I	SYBR GREEN	GLUCOSE+XLGB	actin	83.23	-
D2	S I	SYBR GREEN	CONTROL	ALP	84.64	-
D3	S I	SYBR GREEN	CONTROL	ALP	83.82	-
D4	S I	SYBR GREEN	CONTROL	ALP	83.71	-
D5	S I	SYBR GREEN	GLUCOSE	ALP	84.85	-
D6	S I	SYBR GREEN	GLUCOSE	ALP	84.57	-
D7	S I	SYBR GREEN	GLUCOSE	ALP	84.62	-
D8	S I	SYBR GREEN	GLUCOSE+XLGB	ALP	84.71	-
D9	S I	SYBR GREEN	GLUCOSE+XLGB	ALP	83.98	-
D10	S I	SYBR GREEN	GLUCOSE+XLGB	ALP	83.68	-
E2	S I	SYBR GREEN	CONTROL	OPN	84.7	-
E3	S I	SYBR GREEN	CONTROL	OPN	84.56	-
E4	S I	SYBR GREEN	CONTROL	OPN	84.48	-
E5	S I	SYBR GREEN	GLUCOSE	OPN	85.47	-
E6	S I	SYBR GREEN	GLUCOSE	OPN	85.38	-
E7	S I	SYBR GREEN	GLUCOSE	OPN	85.59	-
E8	S I	SYBR GREEN	GLUCOSE+XLGB	OPN	85.54	-
E9	S I	SYBR GREEN	GLUCOSE+XLGB	OPN	85.55	-
E10	S I	SYBR GREEN	GLUCOSE+XLGB	OPN	84.32	-

Rn Curve

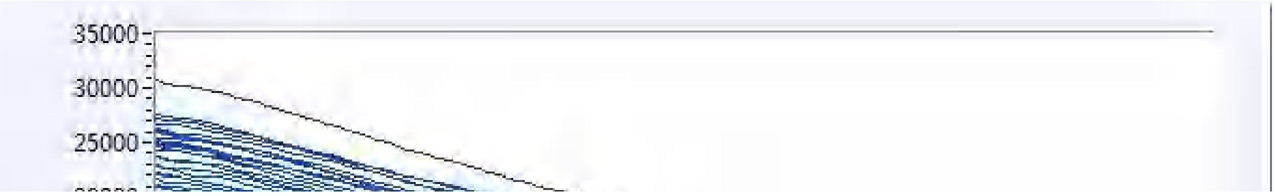

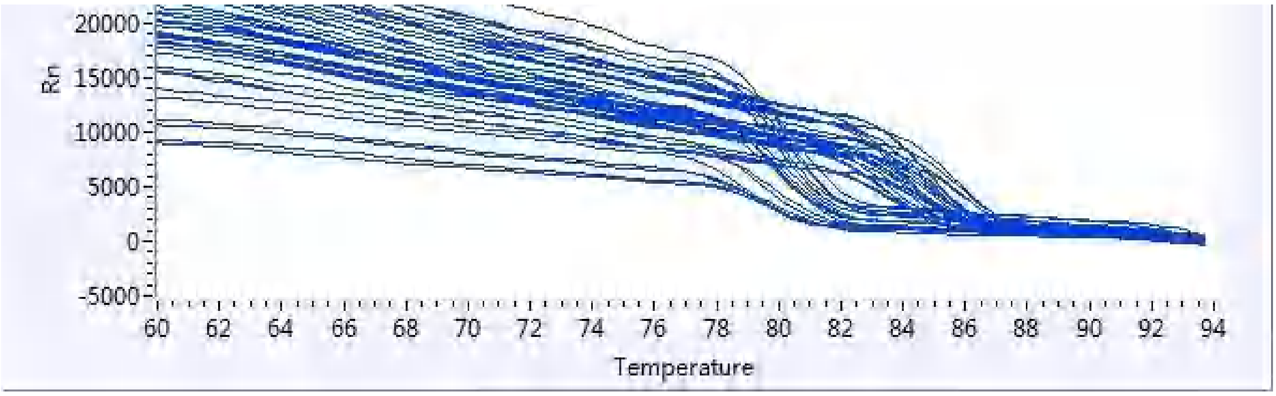

Rn
20000
15000
10000
5000
0
-5000
60
62
64
66
68
70
72
74
76
78
80
82
84
86
88
90
92
94
Temperature

-(di/dt) Curve

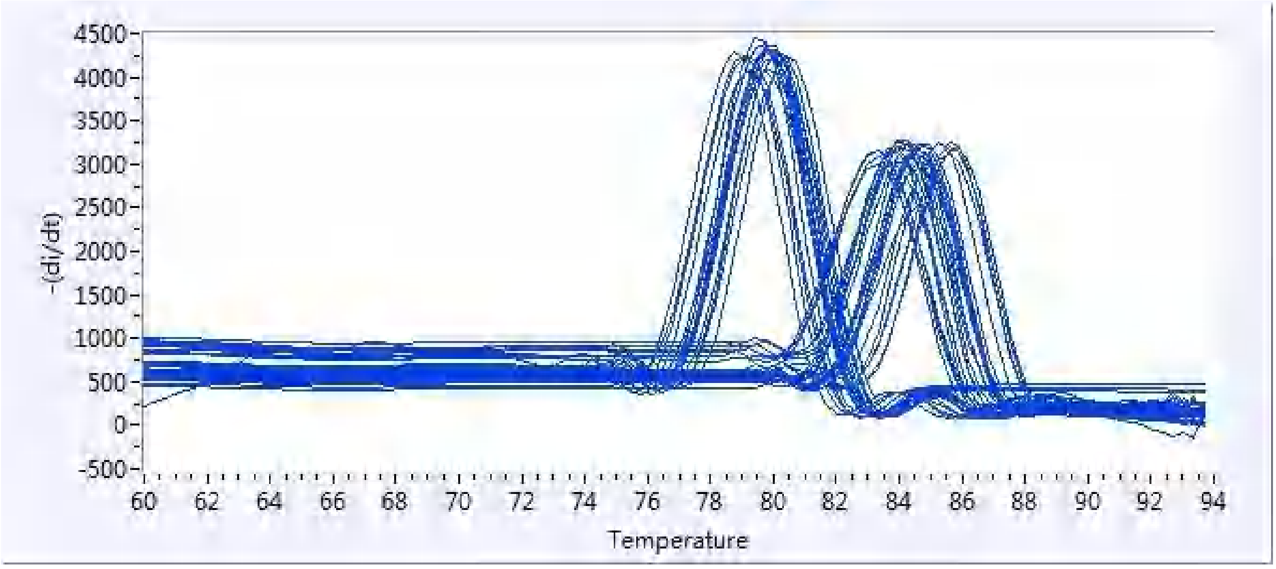

-(di/dt)
4500
4000
3500
3000
2500
2000
1500
1000
500
0
-500
60
62
64
66
68
70
72
74
76
78
80
82
84
86
88
90
92
94
Temperature

–
–
–
–
–
–
–
–
–
–
–
–
–
–
–
–
–
–
–
–
–
–
–
–
–
–
–
–
–
–
–
–
–
–
–
–
–
–
–
–
–
–
–
–
–

Subset Id	Control Group	Reference Gene

Table of Original Data

Well	Subset Id	Sample Name	Gene	Reporter	Ct	
	D2	S I	CONTROL	ALP	SYBR GREEN	24.1119
	D3	S I	CONTROL	ALP	SYBR GREEN	24.0336
	D4	S I	CONTROL	ALP	SYBR GREEN	24.2387
	A2	S I	CONTROL	OCN	SYBR GREEN	18.7099
	A3	S I	CONTROL	OCN	SYBR GREEN	18.645
	A4	S I	CONTROL	OCN	SYBR GREEN	18.5677
	E2	S I	CONTROL	OPN	SYBR GREEN	21.4938
	E3	S I	CONTROL	OPN	SYBR GREEN	21.6919
	E4	S I	CONTROL	OPN	SYBR GREEN	21.6231
	B2	S I	CONTROL	RUNX2	SYBR GREEN	17.6553
	B3	S I	CONTROL	RUNX2	SYBR GREEN	17.7305
	B4	S I	CONTROL	RUNX2	SYBR GREEN	17.9356
	C2	S I	CONTROL	actin	SYBR GREEN	12.1268
	C3	S I	CONTROL	actin	SYBR GREEN	12.1225
	C4	S I	CONTROL	actin	SYBR GREEN	12.2978
	D5	S I	GLUCOSE	ALP	SYBR GREEN	24.6053
	D6	S I	GLUCOSE	ALP	SYBR GREEN	24.6631
	D7	S I	GLUCOSE	ALP	SYBR GREEN	24.324
	A5	S I	GLUCOSE	OCN	SYBR GREEN	19.1102
	A6	S I	GLUCOSE	OCN	SYBR GREEN	18.8736
	A7	S I	GLUCOSE	OCN	SYBR GREEN	19.1175
	E5	S I	GLUCOSE	OPN	SYBR GREEN	22.1365
	E6	S I	GLUCOSE	OPN	SYBR GREEN	22.2826
	E7	S I	GLUCOSE	OPN	SYBR GREEN	22.0118
	B5	S I	GLUCOSE	RUNX2	SYBR GREEN	17.8151
	B6	S I	GLUCOSE	RUNX2	SYBR GREEN	18.0131
	B7	S I	GLUCOSE	RUNX2	SYBR GREEN	17.6387
	C5	S I	GLUCOSE	actin	SYBR GREEN	11.1088
	C6	S I	GLUCOSE	actin	SYBR GREEN	11.5179
	C7	S I	GLUCOSE	actin	SYBR GREEN	11.1717
	D8	S I	GLUCOSE+XLGB	ALP	SYBR GREEN	23.8423
	D9	S I	GLUCOSE+XLGB	ALP	SYBR GREEN	23.8155
	D10	S I	GLUCOSE+XLGB	ALP	SYBR GREEN	23.638
	A8	S I	GLUCOSE+XLGB	OCN	SYBR GREEN	18.3962
	A9	S I	GLUCOSE+XLGB	OCN	SYBR GREEN	18.3551
	A10	S I	GLUCOSE+XLGB	OCN	SYBR GREEN	18.2085
	E8	S I	GLUCOSE+XLGB	OPN	SYBR GREEN	20.9815
	E9	S I	GLUCOSE+XLGB	OPN	SYBR GREEN	20.8743
	E10	S I	GLUCOSE+XLGB	OPN	SYBR GREEN	20.9495
	B8	S I	GLUCOSE+XLGB	RUNX2	SYBR GREEN	17.4136
	B9	S I	GLUCOSE+XLGB	RUNX2	SYBR GREEN	17.3673
	B10	S I	GLUCOSE+XLGB	RUNX2	SYBR GREEN	17.5613
	C8	S I	GLUCOSE+XLGB	actin	SYBR GREEN	11.981
	C9	S I	GLUCOSE+XLGB	actin	SYBR GREEN	11.8032
	C10	S I	GLUCOSE+XLGB	actin	SYBR GREEN	11.4552

Table of RQ

Subset Id	Control	Delta Ct	Sample	Target Gene	Delta Ct	DD Ct

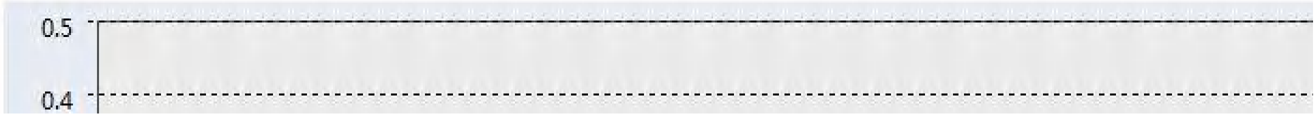

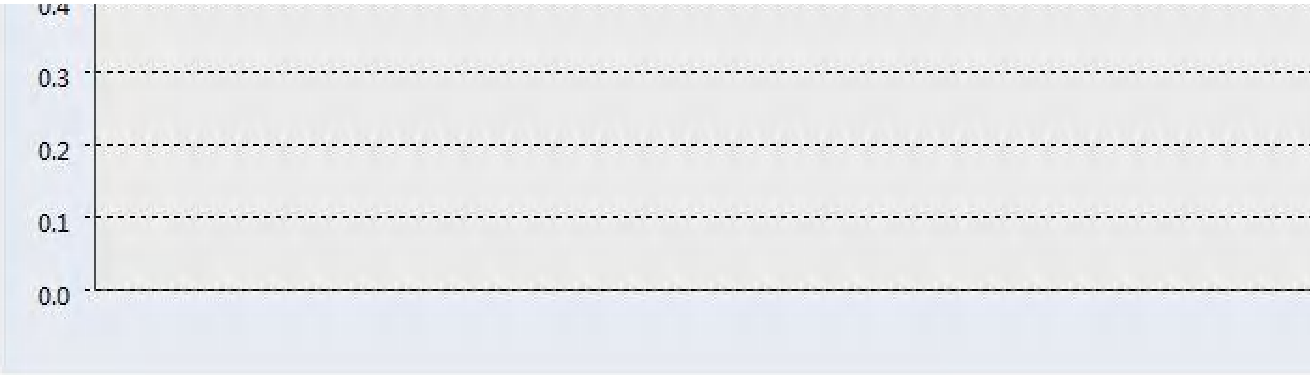
0.4
0.3
0.2
0.1
0.0

RQ	RQ max	RQ min

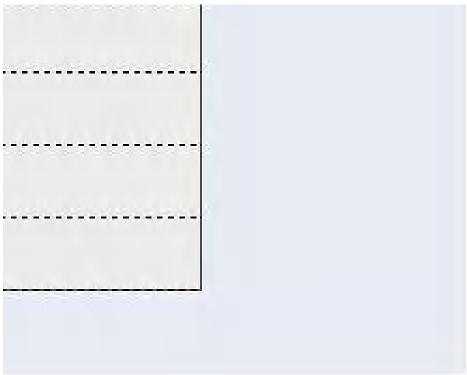

组织名称	编号	OCN	actin	内参平均值	C-E	F最大平均	F-G	函数POWER	I平均值
CONTROL	1	18.71	12.13	12.18333	6.526667	6.456667	0.07	0.952638	1.000785
CONTROL	2	18.64	12.12	12.18333	6.456667	6.456667	0	1	
CONTROL	3	18.57	12.3	12.18333	6.386667	6.456667	-0.07	1.049717	
GLUCOSE	1	19.11	11.11	11.26667	7.843333	6.456667	1.386667	0.382447	0.40464
GLUCOSE	2	18.87	11.52	11.26667	7.603333	6.456667	1.146667	0.451668	
GLUCOSE	3	19.12	11.17	11.26667	7.853333	6.456667	1.396667	0.379806	
GLUCOSE+XI	1	18.4	11.98	11.74667	6.653333	6.456667	0.196667	0.872564	0.921683
GLUCOSE+XI	2	18.36	11.8	11.74667	6.613333	6.456667	0.156667	0.897095	
GLUCOSE+XI	3	18.21	11.46	11.74667	6.463333	6.456667	0.006667	0.99539	

组织名称	编号	RUNX2	actin	内参平均值	C-E	F最大平均	F-G	函数POWER	I平均值
CONTROL	1	17.66	12.13	12.18333	5.476667	5.593333	-0.11667	1.084227	1.003354
CONTROL	2	17.73	12.12	12.18333	5.546667	5.593333	-0.04667	1.032876	
CONTROL	3	17.94	12.3	12.18333	5.756667	5.593333	0.163333	0.89296	
GLUCOSE	1	17.82	11.11	11.26667	6.553333	5.593333	0.96	0.514057	0.515683
GLUCOSE	2	18.01	11.52	11.26667	6.743333	5.593333	1.15	0.450625	
GLUCOSE	3	17.64	11.17	11.26667	6.373333	5.593333	0.78	0.582367	
GLUCOSE+XI	1	17.41	11.98	11.74667	5.663333	5.593333	0.07	0.952638	0.930208
GLUCOSE+XI	2	17.37	11.8	11.74667	5.623333	5.593333	0.03	0.97942	
GLUCOSE+XI	3	17.56	11.46	11.74667	5.813333	5.593333	0.22	0.858565	

组织名称	编号	ALP	actin	内参平均值	C-E	F最大平均	F-G	函数POWER	I平均值
CONTROL	1	24.11	12.13	12.18333	11.92667	11.94333	-0.01667	1.011619	1.00179
CONTROL	2	24.03	12.12	12.18333	11.84667	11.94333	-0.09667	1.0693	
CONTROL	3	24.24	12.3	12.18333	12.05667	11.94333	0.113333	0.92445	
GLUCOSE	1	24.61	11.11	11.26667	13.34333	11.94333	1.4	0.378929	0.402748
GLUCOSE	2	24.66	11.52	11.26667	13.39333	11.94333	1.45	0.366021	
GLUCOSE	3	24.32	11.17	11.26667	13.05333	11.94333	1.11	0.463294	
GLUCOSE+XI	1	23.84	11.98	11.74667	12.09333	11.94333	0.15	0.90125	0.950116
GLUCOSE+XI	2	23.82	11.8	11.74667	12.07333	11.94333	0.13	0.913831	
GLUCOSE+XI	3	23.64	11.46	11.74667	11.89333	11.94333	-0.05	1.035265	

组织名称	编号	OPN	actin	内参平均值	C-E	F最大平均	F-G	函数POWER	I平均值
CONTROL	1	21.49	12.13	12.18333	9.306667	9.416667	-0.11	1.079228	1.001661
CONTROL	2	21.69	12.12	12.18333	9.506667	9.416667	0.09	0.939523	
CONTROL	3	21.62	12.3	12.18333	9.436667	9.416667	0.02	0.986233	
GLUCOSE	1	22.14	11.11	11.26667	10.87333	9.416667	1.456667	0.364334	0.364554
GLUCOSE	2	22.28	11.52	11.26667	11.01333	9.416667	1.596667	0.33064	
GLUCOSE	3	22.01	11.17	11.26667	10.74333	9.416667	1.326667	0.398688	
GLUCOSE+XI	1	20.98	11.98	11.74667	9.233333	9.416667	-0.18333	1.135504	1.173446
GLUCOSE+XI	2	20.87	11.8	11.74667	9.123333	9.416667	-0.29333	1.225468	
GLUCOSE+XI	3	20.95	11.46	11.74667	9.203333	9.416667	-0.21333	1.159364	

ALP	1.00179	0.072924	3	0.402748	0.05283	3	0.950116	0.074009	3
OCN	1.000785	0.048544	3	0.40464	0.040748	3	0.921683	0.065	3
OPN	1.001661	0.071119	3	0.364554	0.034025	3	1.173446	0.046606	3

RUNX2	1.003354	0.098992	3	0.515683	0.065886	3	0.930208	0.063473	3

函数STDEV
0.048544

0.040748

0.065

函数STDEV
0.098992

0.065886

0.063473

函数STDEV
0.072924

0.05283

0.074009

函数STDEV
0.071119

0.034025

0.046606

Experiment Information

Name	P_200615
Date	43997
Operator	admin
Location	F:\Funglyn\FTC-3000\experiments\admin
Template	F:\Funglyn\FTC-3000\templates\SYBR Green 两步法.tmt
Tips	

Protocol

Stage:Hold Cycles:1

Target	Hold(H:M:S)	Ramp Rate(C/s)	Touch Down	Multiple Temp	Singnal Acq.
95	0.416666667				

Stage:Cycles Cycles:40

Target	Hold(H:M:S)	Ramp Rate(C/s)	Touch Down	Multiple Temp	Singnal Acq.
95	0.010416667				
60	0.041666667				TRUE

Stage:Dissociation

Target	Hold(H:M:S)	Ramp Rate(C/s)	Touch Down	Multiple Temp	Singnal Acq.
94	0.0625				
60	0.125	0.1			
94	0.006944444				TRUE

Plate

Well	Reporter	Subset ID	Sample Name	Gene	Sample Type	Quantity
A2	SYBR GREEN	S I	CONTROL	OCN	Unknown Sample	
A3	SYBR GREEN	S I	CONTROL	OCN	Unknown Sample	
A4	SYBR GREEN	S I	CONTROL	OCN	Unknown Sample	
A5	SYBR GREEN	S I	GLUCOSE	OCN	Unknown Sample	
A6	SYBR GREEN	S I	GLUCOSE	OCN	Unknown Sample	
A7	SYBR GREEN	S I	GLUCOSE	OCN	Unknown Sample	
A8	SYBR GREEN	S I	GLUCOSE+XLGB	OCN	Unknown Sample	
A9	SYBR GREEN	S I	GLUCOSE+XLGB	OCN	Unknown Sample	
A10	SYBR GREEN	S I	GLUCOSE+XLGB	OCN	Unknown Sample	
B2	SYBR GREEN	S I	CONTROL	RUNX2	Unknown Sample	
B3	SYBR GREEN	S I	CONTROL	RUNX2	Unknown Sample	
B4	SYBR GREEN	S I	CONTROL	RUNX2	Unknown Sample	
B5	SYBR GREEN	S I	GLUCOSE	RUNX2	Unknown Sample	
B6	SYBR GREEN	S I	GLUCOSE	RUNX2	Unknown Sample	
B7	SYBR GREEN	S I	GLUCOSE	RUNX2	Unknown Sample	
B8	SYBR GREEN	S I	GLUCOSE+XLGB	RUNX2	Unknown Sample	
B9	SYBR GREEN	S I	GLUCOSE+XLGB	RUNX2	Unknown Sample	
B10	SYBR GREEN	S I	GLUCOSE+XLGB	RUNX2	Unknown Sample	
C2	SYBR GREEN	S I	CONTROL	actin	Unknown Sample	
C3	SYBR GREEN	S I	CONTROL	actin	Unknown Sample	
C4	SYBR GREEN	S I	CONTROL	actin	Unknown Sample	
C5	SYBR GREEN	S I	GLUCOSE	actin	Unknown Sample	
C6	SYBR GREEN	S I	GLUCOSE	actin	Unknown Sample	
C7	SYBR GREEN	S I	GLUCOSE	actin	Unknown Sample	
C8	SYBR GREEN	S I	GLUCOSE+XLGB	actin	Unknown Sample	
C9	SYBR GREEN	S I	GLUCOSE+XLGB	actin	Unknown Sample	
C10	SYBR GREEN	S I	GLUCOSE+XLGB	actin	Unknown Sample	
D2	SYBR GREEN	S I	CONTROL	ALP	Unknown Sample	

D3	SYBR GREEN	S I	CONTROL	ALP	Unknown Sample
D4	SYBR GREEN	S I	CONTROL	ALP	Unknown Sample
D5	SYBR GREEN	S I	GLUCOSE	ALP	Unknown Sample
D6	SYBR GREEN	S I	GLUCOSE	ALP	Unknown Sample
D7	SYBR GREEN	S I	GLUCOSE	ALP	Unknown Sample
D8	SYBR GREEN	S I	GLUCOSE+XLGB	ALP	Unknown Sample
D9	SYBR GREEN	S I	GLUCOSE+XLGB	ALP	Unknown Sample
D10	SYBR GREEN	S I	GLUCOSE+XLGB	ALP	Unknown Sample
E2	SYBR GREEN	S I	CONTROL	OPN	Unknown Sample
E3	SYBR GREEN	S I	CONTROL	OPN	Unknown Sample
E4	SYBR GREEN	S I	CONTROL	OPN	Unknown Sample
E5	SYBR GREEN	S I	GLUCOSE	OPN	Unknown Sample
E6	SYBR GREEN	S I	GLUCOSE	OPN	Unknown Sample
E7	SYBR GREEN	S I	GLUCOSE	OPN	Unknown Sample
E8	SYBR GREEN	S I	GLUCOSE+XLGB	OPN	Unknown Sample
E9	SYBR GREEN	S I	GLUCOSE+XLGB	OPN	Unknown Sample
E10	SYBR GREEN	S I	GLUCOSE+XLGB	OPN	Unknown Sample

Data

Well	Subset ID	Reporter	S_Name	S_Type	Ct	Mean Ct
A2	S I	SYBR GREEN	CONTROL	Unknown Sample	18.71	18.64
A3	S I	SYBR GREEN	CONTROL	Unknown Sample	18.64	18.64
A4	S I	SYBR GREEN	CONTROL	Unknown Sample	18.57	18.64
A5	S I	SYBR GREEN	GLUCOSE	Unknown Sample	19.11	19.03
A6	S I	SYBR GREEN	GLUCOSE	Unknown Sample	18.87	19.03
A7	S I	SYBR GREEN	GLUCOSE	Unknown Sample	19.12	19.03
A8	S I	SYBR GREEN	GLUCOSE+XLGB	Unknown Sample	18.4	18.32
A9	S I	SYBR GREEN	GLUCOSE+XLGB	Unknown Sample	18.36	18.32
A10	S I	SYBR GREEN	GLUCOSE+XLGB	Unknown Sample	18.21	18.32
B2	S I	SYBR GREEN	CONTROL	Unknown Sample	17.66	17.78
B3	S I	SYBR GREEN	CONTROL	Unknown Sample	17.73	17.78
B4	S I	SYBR GREEN	CONTROL	Unknown Sample	17.94	17.78
B5	S I	SYBR GREEN	GLUCOSE	Unknown Sample	17.82	17.82
B6	S I	SYBR GREEN	GLUCOSE	Unknown Sample	18.01	17.82
B7	S I	SYBR GREEN	GLUCOSE	Unknown Sample	17.64	17.82
B8	S I	SYBR GREEN	GLUCOSE+XLGB	Unknown Sample	17.41	17.45
B9	S I	SYBR GREEN	GLUCOSE+XLGB	Unknown Sample	17.37	17.45
B10	S I	SYBR GREEN	GLUCOSE+XLGB	Unknown Sample	17.56	17.45
C2	S I	SYBR GREEN	CONTROL	Unknown Sample	12.13	12.18
C3	S I	SYBR GREEN	CONTROL	Unknown Sample	12.12	12.18
C4	S I	SYBR GREEN	CONTROL	Unknown Sample	12.3	12.18
C5	S I	SYBR GREEN	GLUCOSE	Unknown Sample	11.11	11.27
C6	S I	SYBR GREEN	GLUCOSE	Unknown Sample	11.52	11.27
C7	S I	SYBR GREEN	GLUCOSE	Unknown Sample	11.17	11.27
C8	S I	SYBR GREEN	GLUCOSE+XLGB	Unknown Sample	11.98	11.75
C9	S I	SYBR GREEN	GLUCOSE+XLGB	Unknown Sample	11.8	11.75
C10	S I	SYBR GREEN	GLUCOSE+XLGB	Unknown Sample	11.46	11.75
D2	S I	SYBR GREEN	CONTROL	Unknown Sample	24.11	24.13
D3	S I	SYBR GREEN	CONTROL	Unknown Sample	24.03	24.13
D4	S I	SYBR GREEN	CONTROL	Unknown Sample	24.24	24.13
D5	S I	SYBR GREEN	GLUCOSE	Unknown Sample	24.61	24.53
D6	S I	SYBR GREEN	GLUCOSE	Unknown Sample	24.66	24.53
D7	S I	SYBR GREEN	GLUCOSE	Unknown Sample	24.32	24.53
D8	S I	SYBR GREEN	GLUCOSE+XLGB	Unknown Sample	23.84	23.77
D9	S I	SYBR GREEN	GLUCOSE+XLGB	Unknown Sample	23.82	23.77
D10	S I	SYBR GREEN	GLUCOSE+XLGB	Unknown Sample	23.64	23.77
E2	S I	SYBR GREEN	CONTROL	Unknown Sample	21.49	21.6
E3	S I	SYBR GREEN	CONTROL	Unknown Sample	21.69	21.6
E4	S I	SYBR GREEN	CONTROL	Unknown Sample	21.62	21.6
E5	S I	SYBR GREEN	GLUCOSE	Unknown Sample	22.14	22.14
E6	S I	SYBR GREEN	GLUCOSE	Unknown Sample	22.28	22.14
E7	S I	SYBR GREEN	GLUCOSE	Unknown Sample	22.01	22.14
E8	S I	SYBR GREEN	GLUCOSE+XLGB	Unknown Sample	20.98	20.94
E9	S I	SYBR GREEN	GLUCOSE+XLGB	Unknown Sample	20.87	20.94
E10	S I	SYBR GREEN	GLUCOSE+XLGB	Unknown Sample	20.95	20.94

Graph

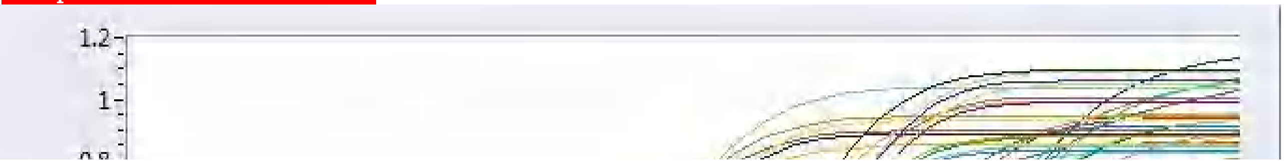

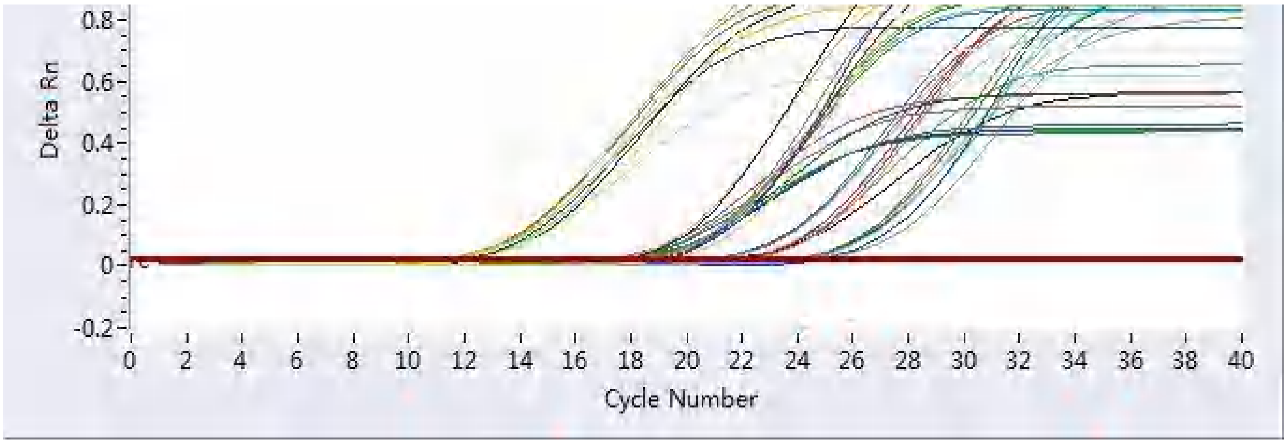

Delta Rn
0.8
0.6
0.4
0.2
0
-0.2
0 2 4 6 8 10 12 14 16 18 20 22 24 26 28 30 32 34 36 38 40
Cycle Number

StdDev Ct	Quantity	Gene
0.07	–	OCN
0.07	–	OCN
0.07	–	OCN
0.14	–	OCN
0.14	–	OCN
0.14	–	OCN
0.1	–	OCN
0.1	–	OCN
0.1	–	OCN
0.15	–	RUNX2
0.15	–	RUNX2
0.15	–	RUNX2
0.19	–	RUNX2
0.19	–	RUNX2
0.19	–	RUNX2
0.1	–	RUNX2
0.1	–	RUNX2
0.1	–	RUNX2
0.1	–	actin
0.1	–	actin
0.1	–	actin
0.22	–	actin
0.22	–	actin
0.22	–	actin
0.27	–	actin
0.27	–	actin
0.27	–	actin
0.1	–	ALP
0.1	–	ALP
0.1	–	ALP
0.18	–	ALP
0.18	–	ALP
0.18	–	ALP
0.11	–	ALP
0.11	–	ALP
0.11	–	ALP
0.1	–	OPN
0.1	–	OPN
0.1	–	OPN
0.14	–	OPN
0.14	–	OPN
0.14	–	OPN
0.06	–	OPN
0.06	–	OPN
0.06	–	OPN

Data

Well	Subset Id	Reporter	Sample Name	Gene	Tm1	Tm2
A2	S I	SYBR GREEN	CONTROL	OCN	80.38	-
A3	S I	SYBR GREEN	CONTROL	OCN	79.66	-
A4	S I	SYBR GREEN	CONTROL	OCN	79.86	-
A5	S I	SYBR GREEN	GLUCOSE	OCN	80.03	-
A6	S I	SYBR GREEN	GLUCOSE	OCN	80.06	-
A7	S I	SYBR GREEN	GLUCOSE	OCN	80.17	-
A8	S I	SYBR GREEN	GLUCOSE+XLGB	OCN	79.62	-
A9	S I	SYBR GREEN	GLUCOSE+XLGB	OCN	79.48	-
A10	S I	SYBR GREEN	GLUCOSE+XLGB	OCN	79.65	-
B2	S I	SYBR GREEN	CONTROL	RUNX2	79.67	-
B3	S I	SYBR GREEN	CONTROL	RUNX2	79.75	-
B4	S I	SYBR GREEN	CONTROL	RUNX2	79.79	-
B5	S I	SYBR GREEN	GLUCOSE	RUNX2	79.77	-
B6	S I	SYBR GREEN	GLUCOSE	RUNX2	78.96	-
B7	S I	SYBR GREEN	GLUCOSE	RUNX2	79.16	-
B8	S I	SYBR GREEN	GLUCOSE+XLGB	RUNX2	79.27	-
B9	S I	SYBR GREEN	GLUCOSE+XLGB	RUNX2	78.99	-
B10	S I	SYBR GREEN	GLUCOSE+XLGB	RUNX2	78.86	-
C2	S I	SYBR GREEN	CONTROL	actin	83.73	-
C3	S I	SYBR GREEN	CONTROL	actin	83.87	-
C4	S I	SYBR GREEN	CONTROL	actin	83.92	-
C5	S I	SYBR GREEN	GLUCOSE	actin	-	-
C6	S I	SYBR GREEN	GLUCOSE	actin	84.06	-
C7	S I	SYBR GREEN	GLUCOSE	actin	82.96	-
C8	S I	SYBR GREEN	GLUCOSE+XLGB	actin	83.25	-
C9	S I	SYBR GREEN	GLUCOSE+XLGB	actin	83.22	-
C10	S I	SYBR GREEN	GLUCOSE+XLGB	actin	83.23	-
D2	S I	SYBR GREEN	CONTROL	ALP	84.64	-
D3	S I	SYBR GREEN	CONTROL	ALP	83.82	-
D4	S I	SYBR GREEN	CONTROL	ALP	83.71	-
D5	S I	SYBR GREEN	GLUCOSE	ALP	84.85	-
D6	S I	SYBR GREEN	GLUCOSE	ALP	84.57	-
D7	S I	SYBR GREEN	GLUCOSE	ALP	84.62	-
D8	S I	SYBR GREEN	GLUCOSE+XLGB	ALP	84.71	-
D9	S I	SYBR GREEN	GLUCOSE+XLGB	ALP	83.98	-
D10	S I	SYBR GREEN	GLUCOSE+XLGB	ALP	83.68	-
E2	S I	SYBR GREEN	CONTROL	OPN	84.7	-
E3	S I	SYBR GREEN	CONTROL	OPN	84.56	-
E4	S I	SYBR GREEN	CONTROL	OPN	84.48	-
E5	S I	SYBR GREEN	GLUCOSE	OPN	85.47	-
E6	S I	SYBR GREEN	GLUCOSE	OPN	85.38	-
E7	S I	SYBR GREEN	GLUCOSE	OPN	85.59	-
E8	S I	SYBR GREEN	GLUCOSE+XLGB	OPN	85.54	-
E9	S I	SYBR GREEN	GLUCOSE+XLGB	OPN	85.55	-
E10	S I	SYBR GREEN	GLUCOSE+XLGB	OPN	84.32	-

Rn Curve

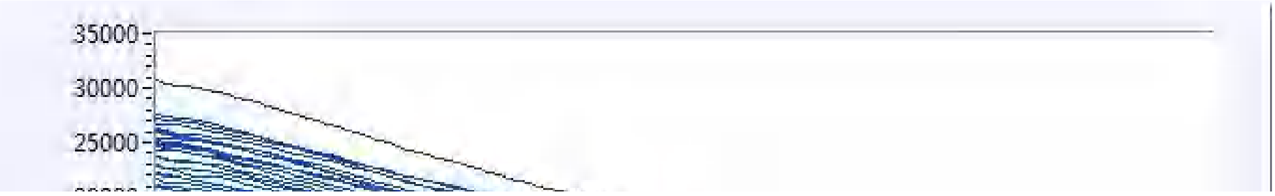

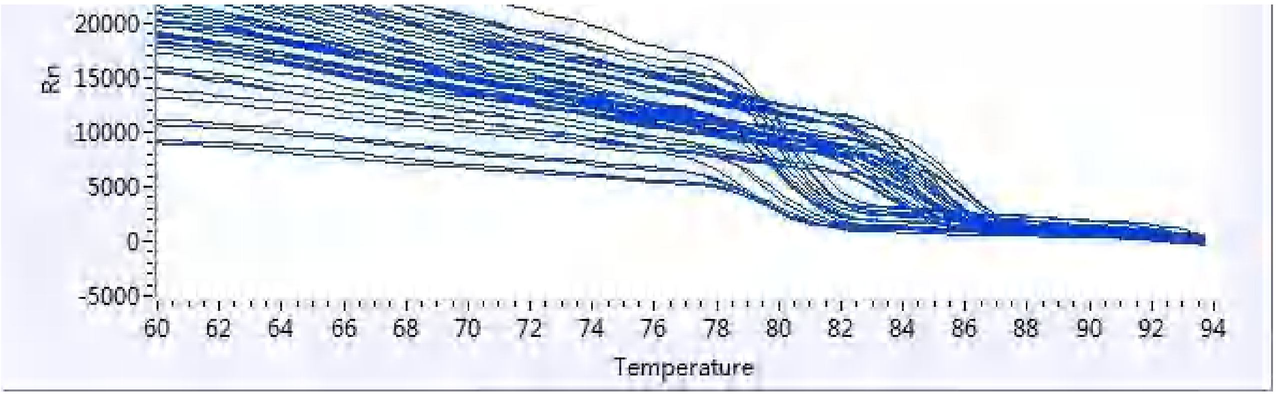

-(di/dt) Curve

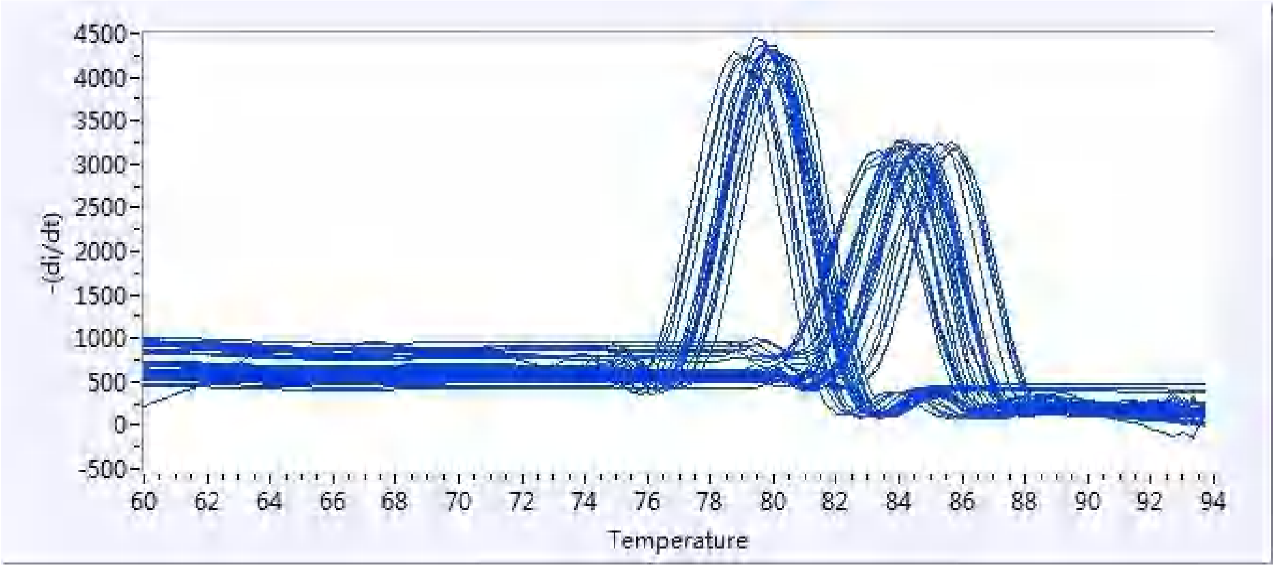

–
–
–
–
–
–
–
–
–
–
–
–
–
–
–
–
–
–
–
–
–
–
–
–
–
–
–
–
–
–
–
–
–
–
–
–
–
–
–
–
–
–
–
–
–

Subset Id	Control Group	Reference Gene

Table of Original Data

Well	Subset Id	Sample Name	Gene	Reporter	Ct	
	D2	S I	CONTROL	ALP	SYBR GREEN	24.1119
	D3	S I	CONTROL	ALP	SYBR GREEN	24.0336
	D4	S I	CONTROL	ALP	SYBR GREEN	24.2387
	A2	S I	CONTROL	OCN	SYBR GREEN	18.7099
	A3	S I	CONTROL	OCN	SYBR GREEN	18.645
	A4	S I	CONTROL	OCN	SYBR GREEN	18.5677
	E2	S I	CONTROL	OPN	SYBR GREEN	21.4938
	E3	S I	CONTROL	OPN	SYBR GREEN	21.6919
	E4	S I	CONTROL	OPN	SYBR GREEN	21.6231
	B2	S I	CONTROL	RUNX2	SYBR GREEN	17.6553
	B3	S I	CONTROL	RUNX2	SYBR GREEN	17.7305
	B4	S I	CONTROL	RUNX2	SYBR GREEN	17.9356
	C2	S I	CONTROL	actin	SYBR GREEN	12.1268
	C3	S I	CONTROL	actin	SYBR GREEN	12.1225
	C4	S I	CONTROL	actin	SYBR GREEN	12.2978
	D5	S I	GLUCOSE	ALP	SYBR GREEN	24.6053
	D6	S I	GLUCOSE	ALP	SYBR GREEN	24.6631
	D7	S I	GLUCOSE	ALP	SYBR GREEN	24.324
	A5	S I	GLUCOSE	OCN	SYBR GREEN	19.1102
	A6	S I	GLUCOSE	OCN	SYBR GREEN	18.8736
	A7	S I	GLUCOSE	OCN	SYBR GREEN	19.1175
	E5	S I	GLUCOSE	OPN	SYBR GREEN	22.1365
	E6	S I	GLUCOSE	OPN	SYBR GREEN	22.2826
	E7	S I	GLUCOSE	OPN	SYBR GREEN	22.0118
	B5	S I	GLUCOSE	RUNX2	SYBR GREEN	17.8151
	B6	S I	GLUCOSE	RUNX2	SYBR GREEN	18.0131
	B7	S I	GLUCOSE	RUNX2	SYBR GREEN	17.6387
	C5	S I	GLUCOSE	actin	SYBR GREEN	11.1088
	C6	S I	GLUCOSE	actin	SYBR GREEN	11.5179
	C7	S I	GLUCOSE	actin	SYBR GREEN	11.1717
	D8	S I	GLUCOSE+XLGB	ALP	SYBR GREEN	23.8423
	D9	S I	GLUCOSE+XLGB	ALP	SYBR GREEN	23.8155
	D10	S I	GLUCOSE+XLGB	ALP	SYBR GREEN	23.638
	A8	S I	GLUCOSE+XLGB	OCN	SYBR GREEN	18.3962
	A9	S I	GLUCOSE+XLGB	OCN	SYBR GREEN	18.3551
	A10	S I	GLUCOSE+XLGB	OCN	SYBR GREEN	18.2085
	E8	S I	GLUCOSE+XLGB	OPN	SYBR GREEN	20.9815
	E9	S I	GLUCOSE+XLGB	OPN	SYBR GREEN	20.8743
	E10	S I	GLUCOSE+XLGB	OPN	SYBR GREEN	20.9495
	B8	S I	GLUCOSE+XLGB	RUNX2	SYBR GREEN	17.4136
	B9	S I	GLUCOSE+XLGB	RUNX2	SYBR GREEN	17.3673
	B10	S I	GLUCOSE+XLGB	RUNX2	SYBR GREEN	17.5613
	C8	S I	GLUCOSE+XLGB	actin	SYBR GREEN	11.981
	C9	S I	GLUCOSE+XLGB	actin	SYBR GREEN	11.8032
	C10	S I	GLUCOSE+XLGB	actin	SYBR GREEN	11.4552

Table of RQ

Subset Id	Control	Delta Ct	Sample	Target Gene	Delta Ct	DD Ct

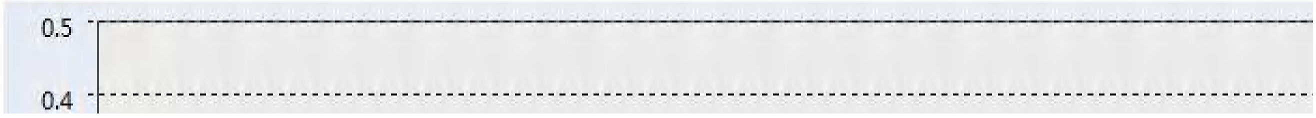

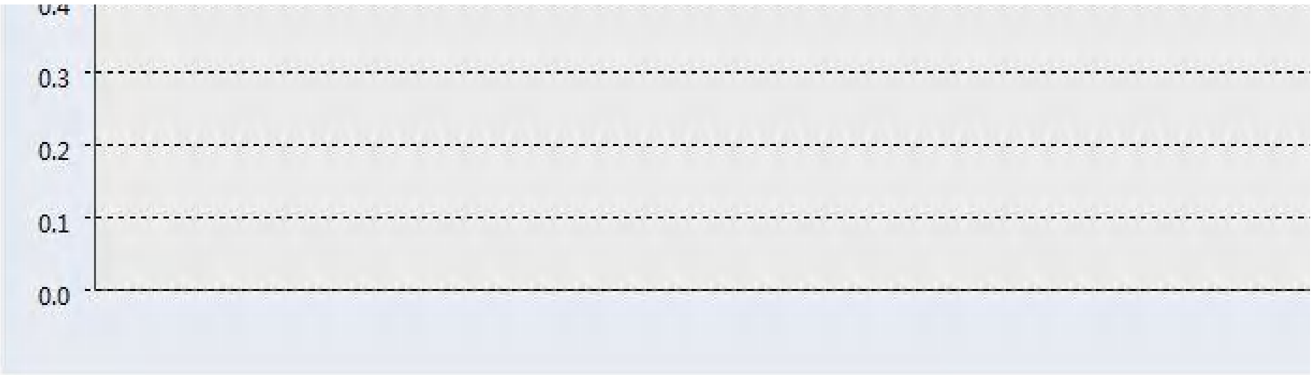
0.4
0.3
0.2
0.1
0.0

RQ	RQ max	RQ min

组织名称	编号	OCN	actin	内参平均值	C-E	F最大平均	F-G	函数POWER	I平均值
CONTROL	1	18.71	12.13	12.18333	6.526667	6.456667	0.07	0.952638	1.000785
CONTROL	2	18.64	12.12	12.18333	6.456667	6.456667	0	1	
CONTROL	3	18.57	12.3	12.18333	6.386667	6.456667	-0.07	1.049717	
GLUCOSE	1	19.11	11.11	11.26667	7.843333	6.456667	1.386667	0.382447	0.40464
GLUCOSE	2	18.87	11.52	11.26667	7.603333	6.456667	1.146667	0.451668	
GLUCOSE	3	19.12	11.17	11.26667	7.853333	6.456667	1.396667	0.379806	
GLUCOSE+XI	1	18.4	11.98	11.74667	6.653333	6.456667	0.196667	0.872564	0.921683
GLUCOSE+XI	2	18.36	11.8	11.74667	6.613333	6.456667	0.156667	0.897095	
GLUCOSE+XI	3	18.21	11.46	11.74667	6.463333	6.456667	0.006667	0.99539	

组织名称	编号	RUNX2	actin	内参平均值	C-E	F最大平均	F-G	函数POWER	I平均值
CONTROL	1	17.66	12.13	12.18333	5.476667	5.593333	-0.11667	1.084227	1.003354
CONTROL	2	17.73	12.12	12.18333	5.546667	5.593333	-0.04667	1.032876	
CONTROL	3	17.94	12.3	12.18333	5.756667	5.593333	0.163333	0.89296	
GLUCOSE	1	17.82	11.11	11.26667	6.553333	5.593333	0.96	0.514057	0.515683
GLUCOSE	2	18.01	11.52	11.26667	6.743333	5.593333	1.15	0.450625	
GLUCOSE	3	17.64	11.17	11.26667	6.373333	5.593333	0.78	0.582367	
GLUCOSE+XI	1	17.41	11.98	11.74667	5.663333	5.593333	0.07	0.952638	0.930208
GLUCOSE+XI	2	17.37	11.8	11.74667	5.623333	5.593333	0.03	0.97942	
GLUCOSE+XI	3	17.56	11.46	11.74667	5.813333	5.593333	0.22	0.858565	

组织名称	编号	ALP	actin	内参平均值	C-E	F最大平均	F-G	函数POWER	I平均值
CONTROL	1	24.11	12.13	12.18333	11.92667	11.94333	-0.01667	1.011619	1.00179
CONTROL	2	24.03	12.12	12.18333	11.84667	11.94333	-0.09667	1.0693	
CONTROL	3	24.24	12.3	12.18333	12.05667	11.94333	0.113333	0.92445	
GLUCOSE	1	24.61	11.11	11.26667	13.34333	11.94333	1.4	0.378929	0.402748
GLUCOSE	2	24.66	11.52	11.26667	13.39333	11.94333	1.45	0.366021	
GLUCOSE	3	24.32	11.17	11.26667	13.05333	11.94333	1.11	0.463294	
GLUCOSE+XI	1	23.84	11.98	11.74667	12.09333	11.94333	0.15	0.90125	0.950116
GLUCOSE+XI	2	23.82	11.8	11.74667	12.07333	11.94333	0.13	0.913831	
GLUCOSE+XI	3	23.64	11.46	11.74667	11.89333	11.94333	-0.05	1.035265	

组织名称	编号	OPN	actin	内参平均值	C-E	F最大平均	F-G	函数POWER	I平均值
CONTROL	1	21.49	12.13	12.18333	9.306667	9.416667	-0.11	1.079228	1.001661
CONTROL	2	21.69	12.12	12.18333	9.506667	9.416667	0.09	0.939523	
CONTROL	3	21.62	12.3	12.18333	9.436667	9.416667	0.02	0.986233	
GLUCOSE	1	22.14	11.11	11.26667	10.87333	9.416667	1.456667	0.364334	0.364554
GLUCOSE	2	22.28	11.52	11.26667	11.01333	9.416667	1.596667	0.33064	
GLUCOSE	3	22.01	11.17	11.26667	10.74333	9.416667	1.326667	0.398688	
GLUCOSE+XI	1	20.98	11.98	11.74667	9.233333	9.416667	-0.18333	1.135504	1.173446
GLUCOSE+XI	2	20.87	11.8	11.74667	9.123333	9.416667	-0.29333	1.225468	
GLUCOSE+XI	3	20.95	11.46	11.74667	9.203333	9.416667	-0.21333	1.159364	

ALP	1.00179	0.072924	3	0.402748	0.05283	3	0.950116	0.074009	3
OCN	1.000785	0.048544	3	0.40464	0.040748	3	0.921683	0.065	3
OPN	1.001661	0.071119	3	0.364554	0.034025	3	1.173446	0.046606	3

RUNX2	1.003354	0.098992	3	0.515683	0.065886	3	0.930208	0.063473	3

函数STDEV
0.048544

0.040748

0.065

函数STDEV
0.098992

0.065886

0.063473

函数STDEV
0.072924

0.05283

0.074009

函数STDEV
0.071119

0.034025

0.046606

Experiment Information

Name	P_200615
Date	43997
Operator	admin
Location	F:\Funglyn\FTC-3000\experiments\admin
Template	F:\Funglyn\FTC-3000\templates\SYBR Green 两步法.tmt
Tips	

Protocol

Stage:Hold Cycles:1

Target	Hold(H:M:S)	Ramp Rate(C/s)	Touch Down	Multiple Temp	Singnal Acq.
95	0.416666667				

Stage:Cycles Cycles:40

Target	Hold(H:M:S)	Ramp Rate(C/s)	Touch Down	Multiple Temp	Singnal Acq.
95	0.010416667				
60	0.041666667				TRUE

Stage:Dissociation

Target	Hold(H:M:S)	Ramp Rate(C/s)	Touch Down	Multiple Temp	Singnal Acq.
94	0.0625				
60	0.125	0.1			
94	0.006944444				TRUE

Plate

Well	Reporter	Subset ID	Sample Name	Gene	Sample Type	Quantity
A2	SYBR GREEN	S I	CONTROL	OCN	Unknown Sample	
A3	SYBR GREEN	S I	CONTROL	OCN	Unknown Sample	
A4	SYBR GREEN	S I	CONTROL	OCN	Unknown Sample	
A5	SYBR GREEN	S I	GLUCOSE	OCN	Unknown Sample	
A6	SYBR GREEN	S I	GLUCOSE	OCN	Unknown Sample	
A7	SYBR GREEN	S I	GLUCOSE	OCN	Unknown Sample	
A8	SYBR GREEN	S I	GLUCOSE+XLGB	OCN	Unknown Sample	
A9	SYBR GREEN	S I	GLUCOSE+XLGB	OCN	Unknown Sample	
A10	SYBR GREEN	S I	GLUCOSE+XLGB	OCN	Unknown Sample	
B2	SYBR GREEN	S I	CONTROL	RUNX2	Unknown Sample	
B3	SYBR GREEN	S I	CONTROL	RUNX2	Unknown Sample	
B4	SYBR GREEN	S I	CONTROL	RUNX2	Unknown Sample	
B5	SYBR GREEN	S I	GLUCOSE	RUNX2	Unknown Sample	
B6	SYBR GREEN	S I	GLUCOSE	RUNX2	Unknown Sample	
B7	SYBR GREEN	S I	GLUCOSE	RUNX2	Unknown Sample	
B8	SYBR GREEN	S I	GLUCOSE+XLGB	RUNX2	Unknown Sample	
B9	SYBR GREEN	S I	GLUCOSE+XLGB	RUNX2	Unknown Sample	
B10	SYBR GREEN	S I	GLUCOSE+XLGB	RUNX2	Unknown Sample	
C2	SYBR GREEN	S I	CONTROL	actin	Unknown Sample	
C3	SYBR GREEN	S I	CONTROL	actin	Unknown Sample	
C4	SYBR GREEN	S I	CONTROL	actin	Unknown Sample	
C5	SYBR GREEN	S I	GLUCOSE	actin	Unknown Sample	
C6	SYBR GREEN	S I	GLUCOSE	actin	Unknown Sample	
C7	SYBR GREEN	S I	GLUCOSE	actin	Unknown Sample	
C8	SYBR GREEN	S I	GLUCOSE+XLGB	actin	Unknown Sample	
C9	SYBR GREEN	S I	GLUCOSE+XLGB	actin	Unknown Sample	
C10	SYBR GREEN	S I	GLUCOSE+XLGB	actin	Unknown Sample	
D2	SYBR GREEN	S I	CONTROL	ALP	Unknown Sample	

D3	SYBR GREEN	S I	CONTROL	ALP	Unknown Sample
D4	SYBR GREEN	S I	CONTROL	ALP	Unknown Sample
D5	SYBR GREEN	S I	GLUCOSE	ALP	Unknown Sample
D6	SYBR GREEN	S I	GLUCOSE	ALP	Unknown Sample
D7	SYBR GREEN	S I	GLUCOSE	ALP	Unknown Sample
D8	SYBR GREEN	S I	GLUCOSE+XLGB	ALP	Unknown Sample
D9	SYBR GREEN	S I	GLUCOSE+XLGB	ALP	Unknown Sample
D10	SYBR GREEN	S I	GLUCOSE+XLGB	ALP	Unknown Sample
E2	SYBR GREEN	S I	CONTROL	OPN	Unknown Sample
E3	SYBR GREEN	S I	CONTROL	OPN	Unknown Sample
E4	SYBR GREEN	S I	CONTROL	OPN	Unknown Sample
E5	SYBR GREEN	S I	GLUCOSE	OPN	Unknown Sample
E6	SYBR GREEN	S I	GLUCOSE	OPN	Unknown Sample
E7	SYBR GREEN	S I	GLUCOSE	OPN	Unknown Sample
E8	SYBR GREEN	S I	GLUCOSE+XLGB	OPN	Unknown Sample
E9	SYBR GREEN	S I	GLUCOSE+XLGB	OPN	Unknown Sample
E10	SYBR GREEN	S I	GLUCOSE+XLGB	OPN	Unknown Sample

Data

Well	Subset ID	Reporter	S_Name	S_Type	Ct	Mean Ct
A2	S I	SYBR GREEN	CONTROL	Unknown Sample	18.71	18.64
A3	S I	SYBR GREEN	CONTROL	Unknown Sample	18.64	18.64
A4	S I	SYBR GREEN	CONTROL	Unknown Sample	18.57	18.64
A5	S I	SYBR GREEN	GLUCOSE	Unknown Sample	19.11	19.03
A6	S I	SYBR GREEN	GLUCOSE	Unknown Sample	18.87	19.03
A7	S I	SYBR GREEN	GLUCOSE	Unknown Sample	19.12	19.03
A8	S I	SYBR GREEN	GLUCOSE+XLGB	Unknown Sample	18.4	18.32
A9	S I	SYBR GREEN	GLUCOSE+XLGB	Unknown Sample	18.36	18.32
A10	S I	SYBR GREEN	GLUCOSE+XLGB	Unknown Sample	18.21	18.32
B2	S I	SYBR GREEN	CONTROL	Unknown Sample	17.66	17.78
B3	S I	SYBR GREEN	CONTROL	Unknown Sample	17.73	17.78
B4	S I	SYBR GREEN	CONTROL	Unknown Sample	17.94	17.78
B5	S I	SYBR GREEN	GLUCOSE	Unknown Sample	17.82	17.82
B6	S I	SYBR GREEN	GLUCOSE	Unknown Sample	18.01	17.82
B7	S I	SYBR GREEN	GLUCOSE	Unknown Sample	17.64	17.82
B8	S I	SYBR GREEN	GLUCOSE+XLGB	Unknown Sample	17.41	17.45
B9	S I	SYBR GREEN	GLUCOSE+XLGB	Unknown Sample	17.37	17.45
B10	S I	SYBR GREEN	GLUCOSE+XLGB	Unknown Sample	17.56	17.45
C2	S I	SYBR GREEN	CONTROL	Unknown Sample	12.13	12.18
C3	S I	SYBR GREEN	CONTROL	Unknown Sample	12.12	12.18
C4	S I	SYBR GREEN	CONTROL	Unknown Sample	12.3	12.18
C5	S I	SYBR GREEN	GLUCOSE	Unknown Sample	11.11	11.27
C6	S I	SYBR GREEN	GLUCOSE	Unknown Sample	11.52	11.27
C7	S I	SYBR GREEN	GLUCOSE	Unknown Sample	11.17	11.27
C8	S I	SYBR GREEN	GLUCOSE+XLGB	Unknown Sample	11.98	11.75
C9	S I	SYBR GREEN	GLUCOSE+XLGB	Unknown Sample	11.8	11.75
C10	S I	SYBR GREEN	GLUCOSE+XLGB	Unknown Sample	11.46	11.75
D2	S I	SYBR GREEN	CONTROL	Unknown Sample	24.11	24.13
D3	S I	SYBR GREEN	CONTROL	Unknown Sample	24.03	24.13
D4	S I	SYBR GREEN	CONTROL	Unknown Sample	24.24	24.13
D5	S I	SYBR GREEN	GLUCOSE	Unknown Sample	24.61	24.53
D6	S I	SYBR GREEN	GLUCOSE	Unknown Sample	24.66	24.53
D7	S I	SYBR GREEN	GLUCOSE	Unknown Sample	24.32	24.53
D8	S I	SYBR GREEN	GLUCOSE+XLGB	Unknown Sample	23.84	23.77
D9	S I	SYBR GREEN	GLUCOSE+XLGB	Unknown Sample	23.82	23.77
D10	S I	SYBR GREEN	GLUCOSE+XLGB	Unknown Sample	23.64	23.77
E2	S I	SYBR GREEN	CONTROL	Unknown Sample	21.49	21.6
E3	S I	SYBR GREEN	CONTROL	Unknown Sample	21.69	21.6
E4	S I	SYBR GREEN	CONTROL	Unknown Sample	21.62	21.6
E5	S I	SYBR GREEN	GLUCOSE	Unknown Sample	22.14	22.14
E6	S I	SYBR GREEN	GLUCOSE	Unknown Sample	22.28	22.14
E7	S I	SYBR GREEN	GLUCOSE	Unknown Sample	22.01	22.14
E8	S I	SYBR GREEN	GLUCOSE+XLGB	Unknown Sample	20.98	20.94
E9	S I	SYBR GREEN	GLUCOSE+XLGB	Unknown Sample	20.87	20.94
E10	S I	SYBR GREEN	GLUCOSE+XLGB	Unknown Sample	20.95	20.94

Graph

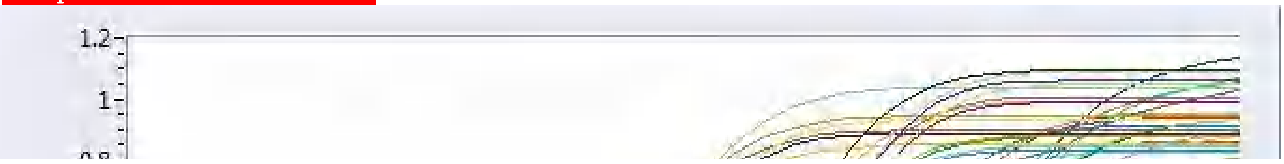

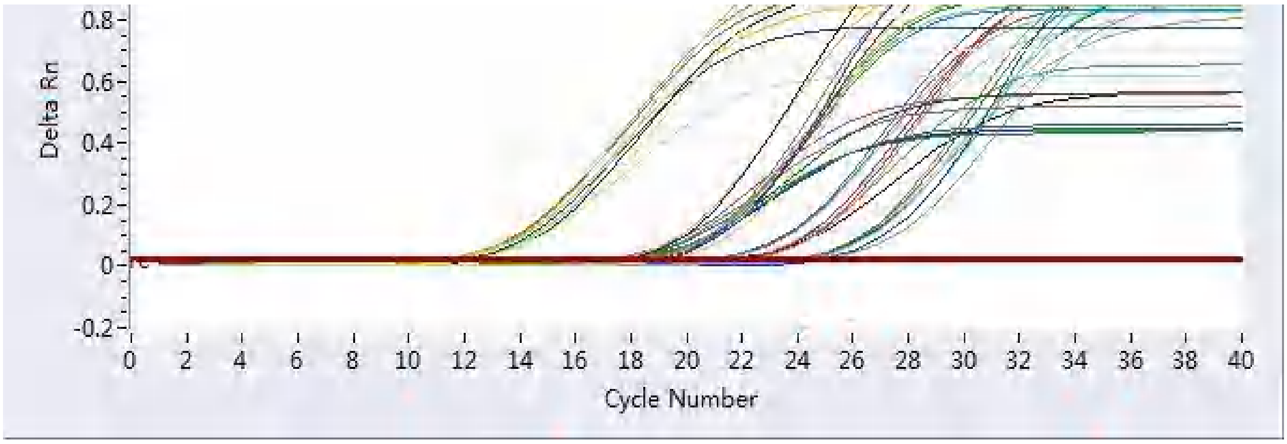
Delta Rn
0.8
0.6
0.4
0.2
0
-0.2
0 2 4 6 8 10 12 14 16 18 20 22 24 26 28 30 32 34 36 38 40
Cycle Number

StdDev Ct	Quantity	Gene
0.07	-	OCN
0.07	-	OCN
0.07	-	OCN
0.14	-	OCN
0.14	-	OCN
0.14	-	OCN
0.1	-	OCN
0.1	-	OCN
0.1	-	OCN
0.15	-	RUNX2
0.15	-	RUNX2
0.15	-	RUNX2
0.19	-	RUNX2
0.19	-	RUNX2
0.19	-	RUNX2
0.1	-	RUNX2
0.1	-	RUNX2
0.1	-	RUNX2
0.1	-	actin
0.1	-	actin
0.1	-	actin
0.22	-	actin
0.22	-	actin
0.22	-	actin
0.27	-	actin
0.27	-	actin
0.27	-	actin
0.1	-	ALP
0.1	-	ALP
0.1	-	ALP
0.18	-	ALP
0.18	-	ALP
0.18	-	ALP
0.11	-	ALP
0.11	-	ALP
0.11	-	ALP
0.1	-	OPN
0.1	-	OPN
0.1	-	OPN
0.14	-	OPN
0.14	-	OPN
0.14	-	OPN
0.06	-	OPN
0.06	-	OPN
0.06	-	OPN

Data

Well	Subset Id	Reporter	Sample Name	Gene	Tm1	Tm2
A2	S I	SYBR GREEN	CONTROL	OCN	80.38	-
A3	S I	SYBR GREEN	CONTROL	OCN	79.66	-
A4	S I	SYBR GREEN	CONTROL	OCN	79.86	-
A5	S I	SYBR GREEN	GLUCOSE	OCN	80.03	-
A6	S I	SYBR GREEN	GLUCOSE	OCN	80.06	-
A7	S I	SYBR GREEN	GLUCOSE	OCN	80.17	-
A8	S I	SYBR GREEN	GLUCOSE+XLGB	OCN	79.62	-
A9	S I	SYBR GREEN	GLUCOSE+XLGB	OCN	79.48	-
A10	S I	SYBR GREEN	GLUCOSE+XLGB	OCN	79.65	-
B2	S I	SYBR GREEN	CONTROL	RUNX2	79.67	-
B3	S I	SYBR GREEN	CONTROL	RUNX2	79.75	-
B4	S I	SYBR GREEN	CONTROL	RUNX2	79.79	-
B5	S I	SYBR GREEN	GLUCOSE	RUNX2	79.77	-
B6	S I	SYBR GREEN	GLUCOSE	RUNX2	78.96	-
B7	S I	SYBR GREEN	GLUCOSE	RUNX2	79.16	-
B8	S I	SYBR GREEN	GLUCOSE+XLGB	RUNX2	79.27	-
B9	S I	SYBR GREEN	GLUCOSE+XLGB	RUNX2	78.99	-
B10	S I	SYBR GREEN	GLUCOSE+XLGB	RUNX2	78.86	-
C2	S I	SYBR GREEN	CONTROL	actin	83.73	-
C3	S I	SYBR GREEN	CONTROL	actin	83.87	-
C4	S I	SYBR GREEN	CONTROL	actin	83.92	-
C5	S I	SYBR GREEN	GLUCOSE	actin	-	-
C6	S I	SYBR GREEN	GLUCOSE	actin	84.06	-
C7	S I	SYBR GREEN	GLUCOSE	actin	82.96	-
C8	S I	SYBR GREEN	GLUCOSE+XLGB	actin	83.25	-
C9	S I	SYBR GREEN	GLUCOSE+XLGB	actin	83.22	-
C10	S I	SYBR GREEN	GLUCOSE+XLGB	actin	83.23	-
D2	S I	SYBR GREEN	CONTROL	ALP	84.64	-
D3	S I	SYBR GREEN	CONTROL	ALP	83.82	-
D4	S I	SYBR GREEN	CONTROL	ALP	83.71	-
D5	S I	SYBR GREEN	GLUCOSE	ALP	84.85	-
D6	S I	SYBR GREEN	GLUCOSE	ALP	84.57	-
D7	S I	SYBR GREEN	GLUCOSE	ALP	84.62	-
D8	S I	SYBR GREEN	GLUCOSE+XLGB	ALP	84.71	-
D9	S I	SYBR GREEN	GLUCOSE+XLGB	ALP	83.98	-
D10	S I	SYBR GREEN	GLUCOSE+XLGB	ALP	83.68	-
E2	S I	SYBR GREEN	CONTROL	OPN	84.7	-
E3	S I	SYBR GREEN	CONTROL	OPN	84.56	-
E4	S I	SYBR GREEN	CONTROL	OPN	84.48	-
E5	S I	SYBR GREEN	GLUCOSE	OPN	85.47	-
E6	S I	SYBR GREEN	GLUCOSE	OPN	85.38	-
E7	S I	SYBR GREEN	GLUCOSE	OPN	85.59	-
E8	S I	SYBR GREEN	GLUCOSE+XLGB	OPN	85.54	-
E9	S I	SYBR GREEN	GLUCOSE+XLGB	OPN	85.55	-
E10	S I	SYBR GREEN	GLUCOSE+XLGB	OPN	84.32	-

Rn Curve

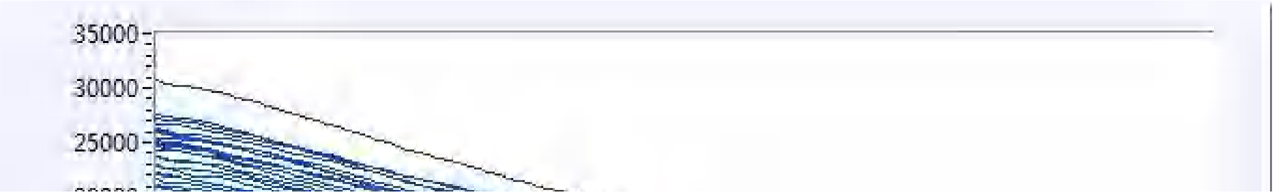

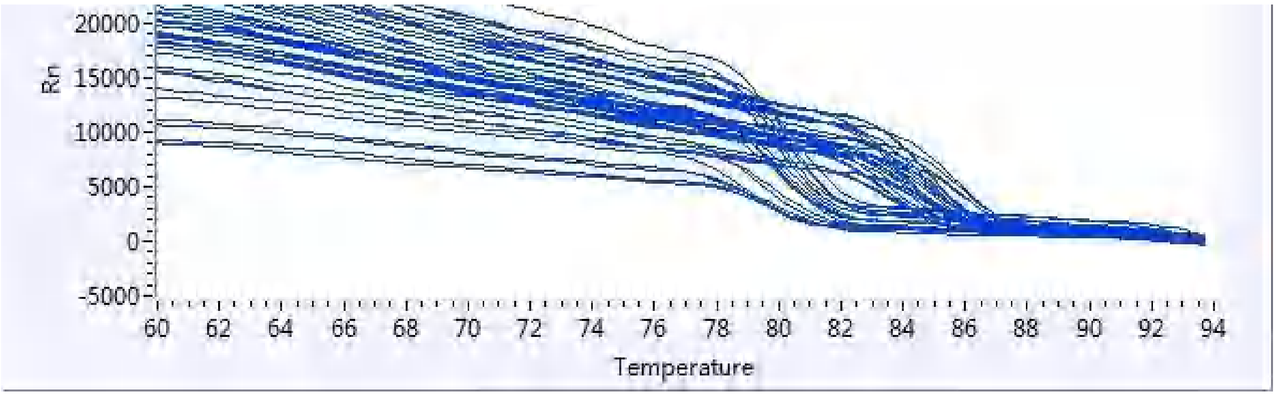
20000
15000
10000
5000
0
-5000
Rn
60
62
64
66
68
70
72
74
76
78
80
82
84
86
88
90
92
94
Temperature

-(di/dt)　Curve

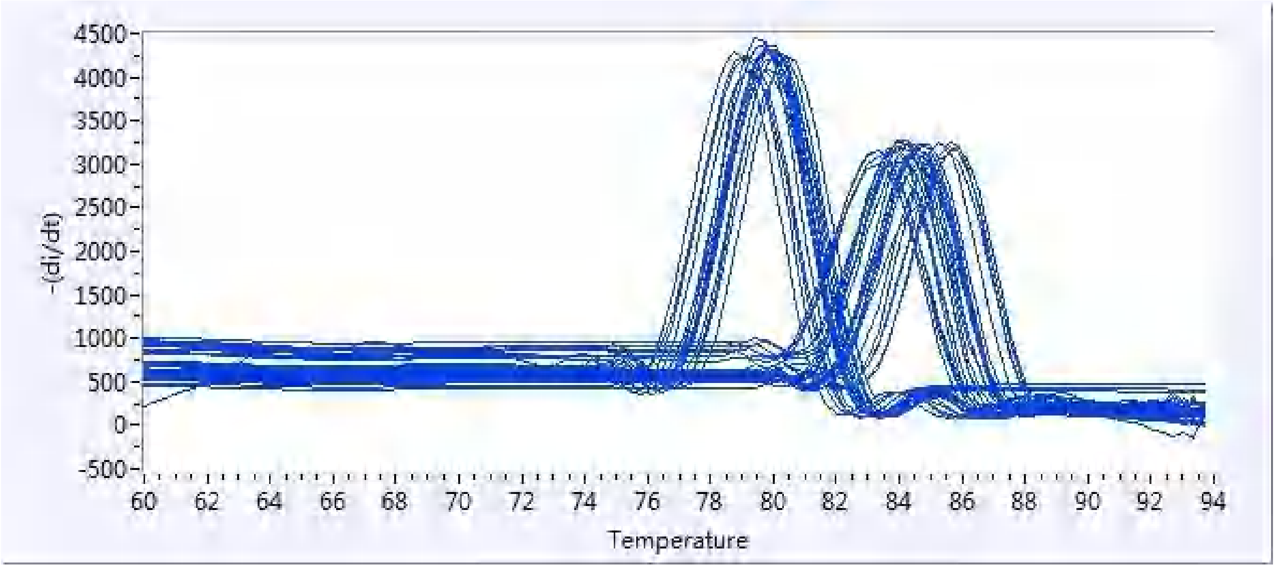
4500
4000
3500
3000
2500
2000
1500
1000
500
0
-500
-(di/dt)
60
62
64
66
68
70
72
74
76
78
80
82
84
86
88
90
92
94
Temperature

–
–
–
–
–
–
–
–
–
–
–
–
–
–
–
–
–
–
–
–
–
–
–
–
–
–
–
–
–
–
–
–
–
–
–
–
–
–
–
–
–
–
–
–
–

Subset Id	Control Group	Reference Gene

Table of Original Data

Well	Subset Id	Sample Name	Gene	Reporter	Ct	
	D2	S I	CONTROL	ALP	SYBR GREEN	24.1119
	D3	S I	CONTROL	ALP	SYBR GREEN	24.0336
	D4	S I	CONTROL	ALP	SYBR GREEN	24.2387
	A2	S I	CONTROL	OCN	SYBR GREEN	18.7099
	A3	S I	CONTROL	OCN	SYBR GREEN	18.645
	A4	S I	CONTROL	OCN	SYBR GREEN	18.5677
	E2	S I	CONTROL	OPN	SYBR GREEN	21.4938
	E3	S I	CONTROL	OPN	SYBR GREEN	21.6919
	E4	S I	CONTROL	OPN	SYBR GREEN	21.6231
	B2	S I	CONTROL	RUNX2	SYBR GREEN	17.6553
	B3	S I	CONTROL	RUNX2	SYBR GREEN	17.7305
	B4	S I	CONTROL	RUNX2	SYBR GREEN	17.9356
	C2	S I	CONTROL	actin	SYBR GREEN	12.1268
	C3	S I	CONTROL	actin	SYBR GREEN	12.1225
	C4	S I	CONTROL	actin	SYBR GREEN	12.2978
	D5	S I	GLUCOSE	ALP	SYBR GREEN	24.6053
	D6	S I	GLUCOSE	ALP	SYBR GREEN	24.6631
	D7	S I	GLUCOSE	ALP	SYBR GREEN	24.324
	A5	S I	GLUCOSE	OCN	SYBR GREEN	19.1102
	A6	S I	GLUCOSE	OCN	SYBR GREEN	18.8736
	A7	S I	GLUCOSE	OCN	SYBR GREEN	19.1175
	E5	S I	GLUCOSE	OPN	SYBR GREEN	22.1365
	E6	S I	GLUCOSE	OPN	SYBR GREEN	22.2826
	E7	S I	GLUCOSE	OPN	SYBR GREEN	22.0118
	B5	S I	GLUCOSE	RUNX2	SYBR GREEN	17.8151
	B6	S I	GLUCOSE	RUNX2	SYBR GREEN	18.0131
	B7	S I	GLUCOSE	RUNX2	SYBR GREEN	17.6387
	C5	S I	GLUCOSE	actin	SYBR GREEN	11.1088
	C6	S I	GLUCOSE	actin	SYBR GREEN	11.5179
	C7	S I	GLUCOSE	actin	SYBR GREEN	11.1717
	D8	S I	GLUCOSE+XLGB	ALP	SYBR GREEN	23.8423
	D9	S I	GLUCOSE+XLGB	ALP	SYBR GREEN	23.8155
	D10	S I	GLUCOSE+XLGB	ALP	SYBR GREEN	23.638
	A8	S I	GLUCOSE+XLGB	OCN	SYBR GREEN	18.3962
	A9	S I	GLUCOSE+XLGB	OCN	SYBR GREEN	18.3551
	A10	S I	GLUCOSE+XLGB	OCN	SYBR GREEN	18.2085
	E8	S I	GLUCOSE+XLGB	OPN	SYBR GREEN	20.9815
	E9	S I	GLUCOSE+XLGB	OPN	SYBR GREEN	20.8743
	E10	S I	GLUCOSE+XLGB	OPN	SYBR GREEN	20.9495
	B8	S I	GLUCOSE+XLGB	RUNX2	SYBR GREEN	17.4136
	B9	S I	GLUCOSE+XLGB	RUNX2	SYBR GREEN	17.3673
	B10	S I	GLUCOSE+XLGB	RUNX2	SYBR GREEN	17.5613
	C8	S I	GLUCOSE+XLGB	actin	SYBR GREEN	11.981
	C9	S I	GLUCOSE+XLGB	actin	SYBR GREEN	11.8032
	C10	S I	GLUCOSE+XLGB	actin	SYBR GREEN	11.4552

Table of RQ

Subset Id	Control	Delta Ct	Sample	Target Gene	Delta Ct	DD Ct

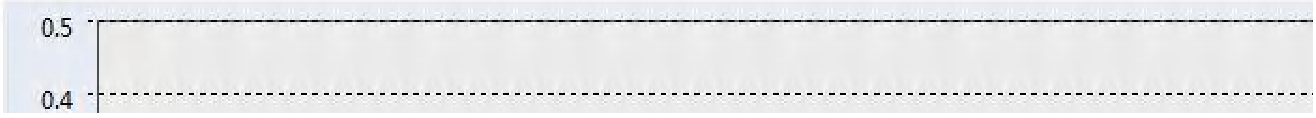

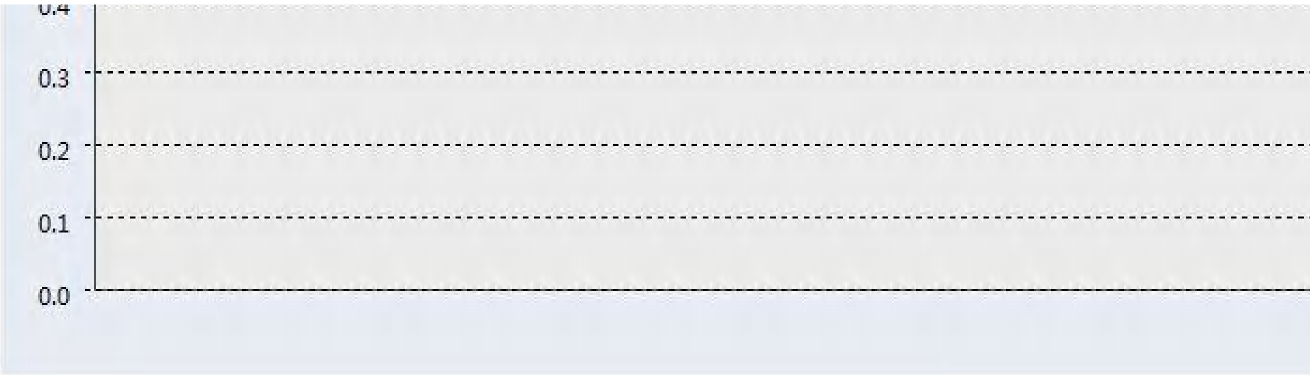
0.4
0.3
0.2
0.1
0.0

RQ	RQ max	RQ min

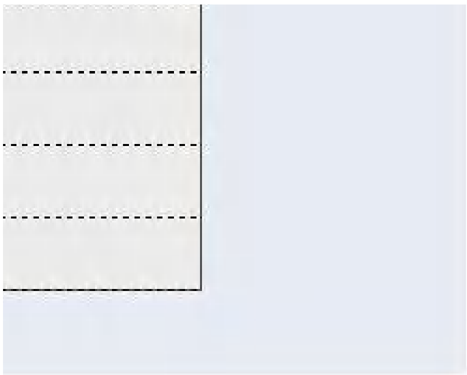

组织名称	编号	OCN	actin	内参平均值	C-E	F最大平均	F-G	函数POWER	I平均值
CONTROL	1	18.71	12.13	12.18333	6.526667	6.456667	0.07	0.952638	1.000785
CONTROL	2	18.64	12.12	12.18333	6.456667	6.456667	0	1	
CONTROL	3	18.57	12.3	12.18333	6.386667	6.456667	-0.07	1.049717	
GLUCOSE	1	19.11	11.11	11.26667	7.843333	6.456667	1.386667	0.382447	0.40464
GLUCOSE	2	18.87	11.52	11.26667	7.603333	6.456667	1.146667	0.451668	
GLUCOSE	3	19.12	11.17	11.26667	7.853333	6.456667	1.396667	0.379806	
GLUCOSE+XI	1	18.4	11.98	11.74667	6.653333	6.456667	0.196667	0.872564	0.921683
GLUCOSE+XI	2	18.36	11.8	11.74667	6.613333	6.456667	0.156667	0.897095	
GLUCOSE+XI	3	18.21	11.46	11.74667	6.463333	6.456667	0.006667	0.99539	

组织名称	编号	RUNX2	actin	内参平均值	C-E	F最大平均	F-G	函数POWER	I平均值
CONTROL	1	17.66	12.13	12.18333	5.476667	5.593333	-0.11667	1.084227	1.003354
CONTROL	2	17.73	12.12	12.18333	5.546667	5.593333	-0.04667	1.032876	
CONTROL	3	17.94	12.3	12.18333	5.756667	5.593333	0.163333	0.89296	
GLUCOSE	1	17.82	11.11	11.26667	6.553333	5.593333	0.96	0.514057	0.515683
GLUCOSE	2	18.01	11.52	11.26667	6.743333	5.593333	1.15	0.450625	
GLUCOSE	3	17.64	11.17	11.26667	6.373333	5.593333	0.78	0.582367	
GLUCOSE+XI	1	17.41	11.98	11.74667	5.663333	5.593333	0.07	0.952638	0.930208
GLUCOSE+XI	2	17.37	11.8	11.74667	5.623333	5.593333	0.03	0.97942	
GLUCOSE+XI	3	17.56	11.46	11.74667	5.813333	5.593333	0.22	0.858565	

组织名称	编号	ALP	actin	内参平均值	C-E	F最大平均	F-G	函数POWER	I平均值
CONTROL	1	24.11	12.13	12.18333	11.92667	11.94333	-0.01667	1.011619	1.00179
CONTROL	2	24.03	12.12	12.18333	11.84667	11.94333	-0.09667	1.0693	
CONTROL	3	24.24	12.3	12.18333	12.05667	11.94333	0.113333	0.92445	
GLUCOSE	1	24.61	11.11	11.26667	13.34333	11.94333	1.4	0.378929	0.402748
GLUCOSE	2	24.66	11.52	11.26667	13.39333	11.94333	1.45	0.366021	
GLUCOSE	3	24.32	11.17	11.26667	13.05333	11.94333	1.11	0.463294	
GLUCOSE+XI	1	23.84	11.98	11.74667	12.09333	11.94333	0.15	0.90125	0.950116
GLUCOSE+XI	2	23.82	11.8	11.74667	12.07333	11.94333	0.13	0.913831	
GLUCOSE+XI	3	23.64	11.46	11.74667	11.89333	11.94333	-0.05	1.035265	

组织名称	编号	OPN	actin	内参平均值	C-E	F最大平均	F-G	函数POWER	I平均值
CONTROL	1	21.49	12.13	12.18333	9.306667	9.416667	-0.11	1.079228	1.001661
CONTROL	2	21.69	12.12	12.18333	9.506667	9.416667	0.09	0.939523	
CONTROL	3	21.62	12.3	12.18333	9.436667	9.416667	0.02	0.986233	
GLUCOSE	1	22.14	11.11	11.26667	10.87333	9.416667	1.456667	0.364334	0.364554
GLUCOSE	2	22.28	11.52	11.26667	11.01333	9.416667	1.596667	0.33064	
GLUCOSE	3	22.01	11.17	11.26667	10.74333	9.416667	1.326667	0.398688	
GLUCOSE+XI	1	20.98	11.98	11.74667	9.233333	9.416667	-0.18333	1.135504	1.173446
GLUCOSE+XI	2	20.87	11.8	11.74667	9.123333	9.416667	-0.29333	1.225468	
GLUCOSE+XI	3	20.95	11.46	11.74667	9.203333	9.416667	-0.21333	1.159364	

ALP	1.00179	0.072924	3	0.402748	0.05283	3	0.950116	0.074009	3
OCN	1.000785	0.048544	3	0.40464	0.040748	3	0.921683	0.065	3
OPN	1.001661	0.071119	3	0.364554	0.034025	3	1.173446	0.046606	3

RUNX2	1.003354	0.098992	3	0.515683	0.065886	3	0.930208	0.063473	3

函数STDEV
0.048544

0.040748

0.065

函数STDEV
0.098992

0.065886

0.063473

函数STDEV
0.072924

0.05283

0.074009

函数STDEV
0.071119

0.034025

0.046606

Experiment Information

Name	P_200615
Date	43997
Operator	admin
Location	F:\Funglyn\FTC-3000\experiments\admin
Template	F:\Funglyn\FTC-3000\templates\SYBR Green 两步法.tmt
Tips	

Protocol

Stage:Hold Cycles:1

Target	Hold(H:M:S)	Ramp Rate(C/s)	Touch Down	Multiple Temp	Singnal Acq.
95	0.416666667				

Stage:Cycles Cycles:40

Target	Hold(H:M:S)	Ramp Rate(C/s)	Touch Down	Multiple Temp	Singnal Acq.
95	0.010416667				
60	0.041666667				TRUE

Stage:Dissociation

Target	Hold(H:M:S)	Ramp Rate(C/s)	Touch Down	Multiple Temp	Singnal Acq.
94	0.0625				
60	0.125	0.1			
94	0.006944444				TRUE

Plate

Well	Reporter	Subset ID	Sample Name	Gene	Sample Type	Quantity
A2	SYBR GREEN	S I	CONTROL	OCN	Unknown Sample	
A3	SYBR GREEN	S I	CONTROL	OCN	Unknown Sample	
A4	SYBR GREEN	S I	CONTROL	OCN	Unknown Sample	
A5	SYBR GREEN	S I	GLUCOSE	OCN	Unknown Sample	
A6	SYBR GREEN	S I	GLUCOSE	OCN	Unknown Sample	
A7	SYBR GREEN	S I	GLUCOSE	OCN	Unknown Sample	
A8	SYBR GREEN	S I	GLUCOSE+XLGB	OCN	Unknown Sample	
A9	SYBR GREEN	S I	GLUCOSE+XLGB	OCN	Unknown Sample	
A10	SYBR GREEN	S I	GLUCOSE+XLGB	OCN	Unknown Sample	
B2	SYBR GREEN	S I	CONTROL	RUNX2	Unknown Sample	
B3	SYBR GREEN	S I	CONTROL	RUNX2	Unknown Sample	
B4	SYBR GREEN	S I	CONTROL	RUNX2	Unknown Sample	
B5	SYBR GREEN	S I	GLUCOSE	RUNX2	Unknown Sample	
B6	SYBR GREEN	S I	GLUCOSE	RUNX2	Unknown Sample	
B7	SYBR GREEN	S I	GLUCOSE	RUNX2	Unknown Sample	
B8	SYBR GREEN	S I	GLUCOSE+XLGB	RUNX2	Unknown Sample	
B9	SYBR GREEN	S I	GLUCOSE+XLGB	RUNX2	Unknown Sample	
B10	SYBR GREEN	S I	GLUCOSE+XLGB	RUNX2	Unknown Sample	
C2	SYBR GREEN	S I	CONTROL	actin	Unknown Sample	
C3	SYBR GREEN	S I	CONTROL	actin	Unknown Sample	
C4	SYBR GREEN	S I	CONTROL	actin	Unknown Sample	
C5	SYBR GREEN	S I	GLUCOSE	actin	Unknown Sample	
C6	SYBR GREEN	S I	GLUCOSE	actin	Unknown Sample	
C7	SYBR GREEN	S I	GLUCOSE	actin	Unknown Sample	
C8	SYBR GREEN	S I	GLUCOSE+XLGB	actin	Unknown Sample	
C9	SYBR GREEN	S I	GLUCOSE+XLGB	actin	Unknown Sample	
C10	SYBR GREEN	S I	GLUCOSE+XLGB	actin	Unknown Sample	
D2	SYBR GREEN	S I	CONTROL	ALP	Unknown Sample	

D3	SYBR GREEN	S I	CONTROL	ALP	Unknown Sample
D4	SYBR GREEN	S I	CONTROL	ALP	Unknown Sample
D5	SYBR GREEN	S I	GLUCOSE	ALP	Unknown Sample
D6	SYBR GREEN	S I	GLUCOSE	ALP	Unknown Sample
D7	SYBR GREEN	S I	GLUCOSE	ALP	Unknown Sample
D8	SYBR GREEN	S I	GLUCOSE+XLGB	ALP	Unknown Sample
D9	SYBR GREEN	S I	GLUCOSE+XLGB	ALP	Unknown Sample
D10	SYBR GREEN	S I	GLUCOSE+XLGB	ALP	Unknown Sample
E2	SYBR GREEN	S I	CONTROL	OPN	Unknown Sample
E3	SYBR GREEN	S I	CONTROL	OPN	Unknown Sample
E4	SYBR GREEN	S I	CONTROL	OPN	Unknown Sample
E5	SYBR GREEN	S I	GLUCOSE	OPN	Unknown Sample
E6	SYBR GREEN	S I	GLUCOSE	OPN	Unknown Sample
E7	SYBR GREEN	S I	GLUCOSE	OPN	Unknown Sample
E8	SYBR GREEN	S I	GLUCOSE+XLGB	OPN	Unknown Sample
E9	SYBR GREEN	S I	GLUCOSE+XLGB	OPN	Unknown Sample
E10	SYBR GREEN	S I	GLUCOSE+XLGB	OPN	Unknown Sample

Data

Well	Subset ID	Reporter	S_Name	S_Type	Ct	Mean Ct
A2	S I	SYBR GREEN	CONTROL	Unknown Sample	18.71	18.64
A3	S I	SYBR GREEN	CONTROL	Unknown Sample	18.64	18.64
A4	S I	SYBR GREEN	CONTROL	Unknown Sample	18.57	18.64
A5	S I	SYBR GREEN	GLUCOSE	Unknown Sample	19.11	19.03
A6	S I	SYBR GREEN	GLUCOSE	Unknown Sample	18.87	19.03
A7	S I	SYBR GREEN	GLUCOSE	Unknown Sample	19.12	19.03
A8	S I	SYBR GREEN	GLUCOSE+XLGB	Unknown Sample	18.4	18.32
A9	S I	SYBR GREEN	GLUCOSE+XLGB	Unknown Sample	18.36	18.32
A10	S I	SYBR GREEN	GLUCOSE+XLGB	Unknown Sample	18.21	18.32
B2	S I	SYBR GREEN	CONTROL	Unknown Sample	17.66	17.78
B3	S I	SYBR GREEN	CONTROL	Unknown Sample	17.73	17.78
B4	S I	SYBR GREEN	CONTROL	Unknown Sample	17.94	17.78
B5	S I	SYBR GREEN	GLUCOSE	Unknown Sample	17.82	17.82
B6	S I	SYBR GREEN	GLUCOSE	Unknown Sample	18.01	17.82
B7	S I	SYBR GREEN	GLUCOSE	Unknown Sample	17.64	17.82
B8	S I	SYBR GREEN	GLUCOSE+XLGB	Unknown Sample	17.41	17.45
B9	S I	SYBR GREEN	GLUCOSE+XLGB	Unknown Sample	17.37	17.45
B10	S I	SYBR GREEN	GLUCOSE+XLGB	Unknown Sample	17.56	17.45
C2	S I	SYBR GREEN	CONTROL	Unknown Sample	12.13	12.18
C3	S I	SYBR GREEN	CONTROL	Unknown Sample	12.12	12.18
C4	S I	SYBR GREEN	CONTROL	Unknown Sample	12.3	12.18
C5	S I	SYBR GREEN	GLUCOSE	Unknown Sample	11.11	11.27
C6	S I	SYBR GREEN	GLUCOSE	Unknown Sample	11.52	11.27
C7	S I	SYBR GREEN	GLUCOSE	Unknown Sample	11.17	11.27
C8	S I	SYBR GREEN	GLUCOSE+XLGB	Unknown Sample	11.98	11.75
C9	S I	SYBR GREEN	GLUCOSE+XLGB	Unknown Sample	11.8	11.75
C10	S I	SYBR GREEN	GLUCOSE+XLGB	Unknown Sample	11.46	11.75
D2	S I	SYBR GREEN	CONTROL	Unknown Sample	24.11	24.13
D3	S I	SYBR GREEN	CONTROL	Unknown Sample	24.03	24.13
D4	S I	SYBR GREEN	CONTROL	Unknown Sample	24.24	24.13
D5	S I	SYBR GREEN	GLUCOSE	Unknown Sample	24.61	24.53
D6	S I	SYBR GREEN	GLUCOSE	Unknown Sample	24.66	24.53
D7	S I	SYBR GREEN	GLUCOSE	Unknown Sample	24.32	24.53
D8	S I	SYBR GREEN	GLUCOSE+XLGB	Unknown Sample	23.84	23.77
D9	S I	SYBR GREEN	GLUCOSE+XLGB	Unknown Sample	23.82	23.77
D10	S I	SYBR GREEN	GLUCOSE+XLGB	Unknown Sample	23.64	23.77
E2	S I	SYBR GREEN	CONTROL	Unknown Sample	21.49	21.6
E3	S I	SYBR GREEN	CONTROL	Unknown Sample	21.69	21.6
E4	S I	SYBR GREEN	CONTROL	Unknown Sample	21.62	21.6
E5	S I	SYBR GREEN	GLUCOSE	Unknown Sample	22.14	22.14
E6	S I	SYBR GREEN	GLUCOSE	Unknown Sample	22.28	22.14
E7	S I	SYBR GREEN	GLUCOSE	Unknown Sample	22.01	22.14
E8	S I	SYBR GREEN	GLUCOSE+XLGB	Unknown Sample	20.98	20.94
E9	S I	SYBR GREEN	GLUCOSE+XLGB	Unknown Sample	20.87	20.94
E10	S I	SYBR GREEN	GLUCOSE+XLGB	Unknown Sample	20.95	20.94

Graph

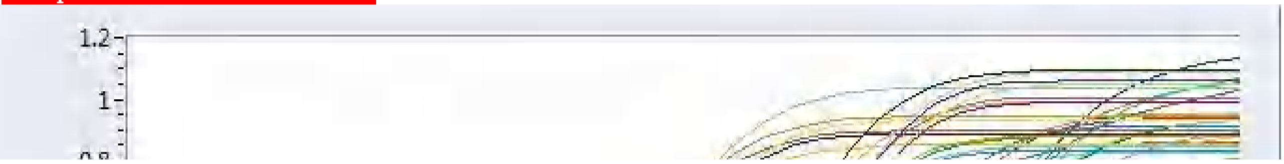

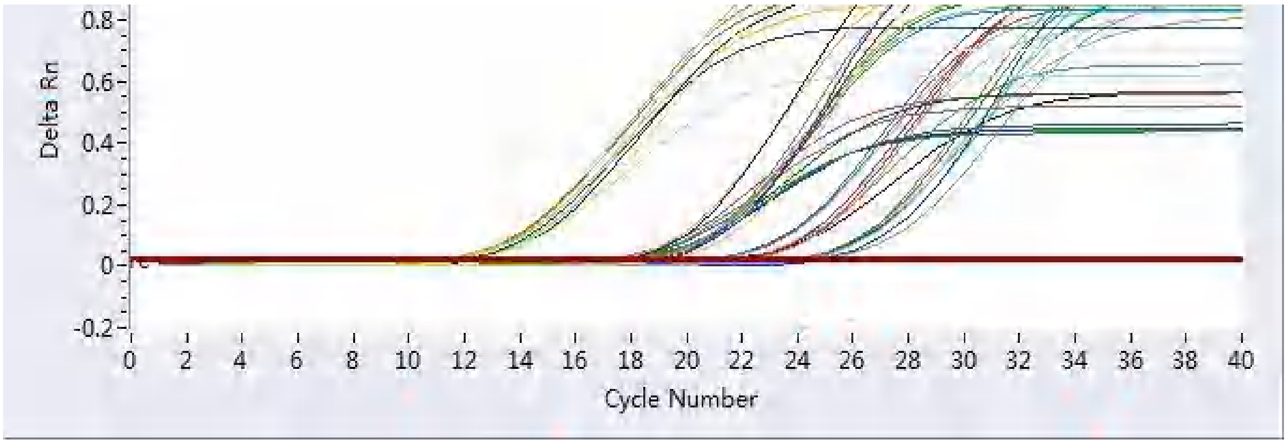
Delta Rn
0.8
0.6
0.4
0.2
0
-0.2
0 2 4 6 8 10 12 14 16 18 20 22 24 26 28 30 32 34 36 38 40
Cycle Number

StdDev Ct	Quantity	Gene
0.07	-	OCN
0.07	-	OCN
0.07	-	OCN
0.14	-	OCN
0.14	-	OCN
0.14	-	OCN
0.1	-	OCN
0.1	-	OCN
0.1	-	OCN
0.15	-	RUNX2
0.15	-	RUNX2
0.15	-	RUNX2
0.19	-	RUNX2
0.19	-	RUNX2
0.19	-	RUNX2
0.1	-	RUNX2
0.1	-	RUNX2
0.1	-	RUNX2
0.1	-	actin
0.1	-	actin
0.1	-	actin
0.22	-	actin
0.22	-	actin
0.22	-	actin
0.27	-	actin
0.27	-	actin
0.27	-	actin
0.1	-	ALP
0.1	-	ALP
0.1	-	ALP
0.18	-	ALP
0.18	-	ALP
0.18	-	ALP
0.11	-	ALP
0.11	-	ALP
0.11	-	ALP
0.1	-	OPN
0.1	-	OPN
0.1	-	OPN
0.14	-	OPN
0.14	-	OPN
0.14	-	OPN
0.06	-	OPN
0.06	-	OPN
0.06	-	OPN

Data

Well	Subset Id	Reporter	Sample Name	Gene	Tm1	Tm2
A2	S I	SYBR GREEN	CONTROL	OCN	80.38	-
A3	S I	SYBR GREEN	CONTROL	OCN	79.66	-
A4	S I	SYBR GREEN	CONTROL	OCN	79.86	-
A5	S I	SYBR GREEN	GLUCOSE	OCN	80.03	-
A6	S I	SYBR GREEN	GLUCOSE	OCN	80.06	-
A7	S I	SYBR GREEN	GLUCOSE	OCN	80.17	-
A8	S I	SYBR GREEN	GLUCOSE+XLGB	OCN	79.62	-
A9	S I	SYBR GREEN	GLUCOSE+XLGB	OCN	79.48	-
A10	S I	SYBR GREEN	GLUCOSE+XLGB	OCN	79.65	-
B2	S I	SYBR GREEN	CONTROL	RUNX2	79.67	-
B3	S I	SYBR GREEN	CONTROL	RUNX2	79.75	-
B4	S I	SYBR GREEN	CONTROL	RUNX2	79.79	-
B5	S I	SYBR GREEN	GLUCOSE	RUNX2	79.77	-
B6	S I	SYBR GREEN	GLUCOSE	RUNX2	78.96	-
B7	S I	SYBR GREEN	GLUCOSE	RUNX2	79.16	-
B8	S I	SYBR GREEN	GLUCOSE+XLGB	RUNX2	79.27	-
B9	S I	SYBR GREEN	GLUCOSE+XLGB	RUNX2	78.99	-
B10	S I	SYBR GREEN	GLUCOSE+XLGB	RUNX2	78.86	-
C2	S I	SYBR GREEN	CONTROL	actin	83.73	-
C3	S I	SYBR GREEN	CONTROL	actin	83.87	-
C4	S I	SYBR GREEN	CONTROL	actin	83.92	-
C5	S I	SYBR GREEN	GLUCOSE	actin	-	-
C6	S I	SYBR GREEN	GLUCOSE	actin	84.06	-
C7	S I	SYBR GREEN	GLUCOSE	actin	82.96	-
C8	S I	SYBR GREEN	GLUCOSE+XLGB	actin	83.25	-
C9	S I	SYBR GREEN	GLUCOSE+XLGB	actin	83.22	-
C10	S I	SYBR GREEN	GLUCOSE+XLGB	actin	83.23	-
D2	S I	SYBR GREEN	CONTROL	ALP	84.64	-
D3	S I	SYBR GREEN	CONTROL	ALP	83.82	-
D4	S I	SYBR GREEN	CONTROL	ALP	83.71	-
D5	S I	SYBR GREEN	GLUCOSE	ALP	84.85	-
D6	S I	SYBR GREEN	GLUCOSE	ALP	84.57	-
D7	S I	SYBR GREEN	GLUCOSE	ALP	84.62	-
D8	S I	SYBR GREEN	GLUCOSE+XLGB	ALP	84.71	-
D9	S I	SYBR GREEN	GLUCOSE+XLGB	ALP	83.98	-
D10	S I	SYBR GREEN	GLUCOSE+XLGB	ALP	83.68	-
E2	S I	SYBR GREEN	CONTROL	OPN	84.7	-
E3	S I	SYBR GREEN	CONTROL	OPN	84.56	-
E4	S I	SYBR GREEN	CONTROL	OPN	84.48	-
E5	S I	SYBR GREEN	GLUCOSE	OPN	85.47	-
E6	S I	SYBR GREEN	GLUCOSE	OPN	85.38	-
E7	S I	SYBR GREEN	GLUCOSE	OPN	85.59	-
E8	S I	SYBR GREEN	GLUCOSE+XLGB	OPN	85.54	-
E9	S I	SYBR GREEN	GLUCOSE+XLGB	OPN	85.55	-
E10	S I	SYBR GREEN	GLUCOSE+XLGB	OPN	84.32	-

Rn Curve

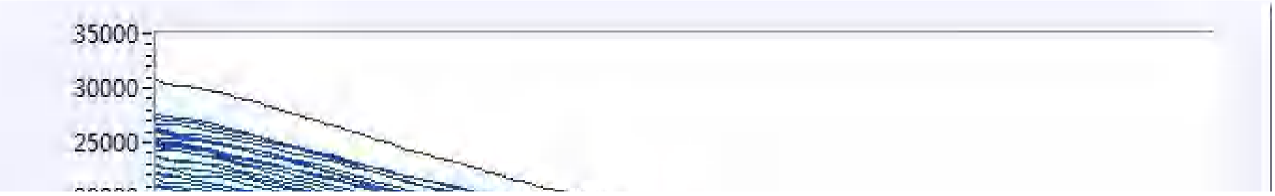

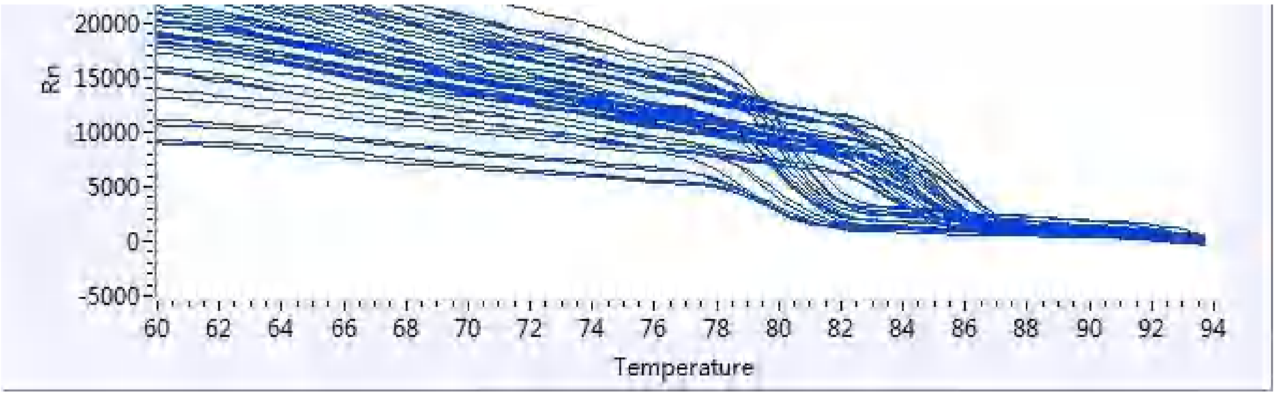

20000
Rn 15000
10000
5000
0
-5000
60 62 64 66 68 70 72 74 76 78 80 82 84 86 88 90 92 94
Temperature

-(di/dt) Curve

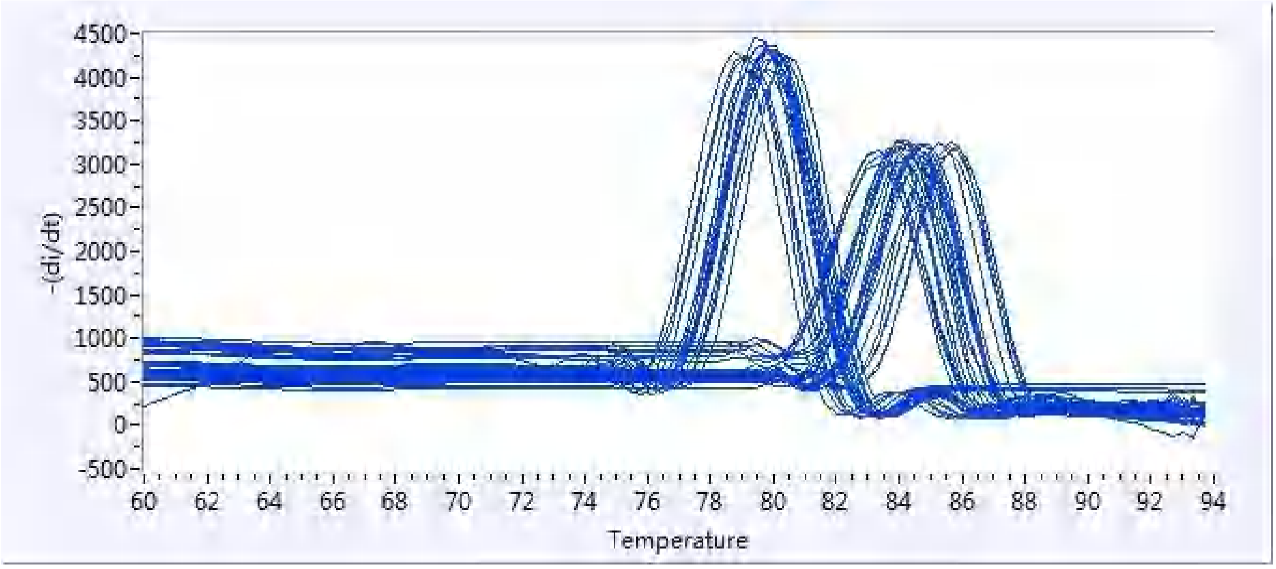

4500
4000
3500
3000
2500
2000
1500
1000
500
0
-500
-(di/dt)
60 62 64 66 68 70 72 74 76 78 80 82 84 86 88 90 92 94
Temperature

–
–
–
–
–
–
–
–
–
–
–
–
–
–
–
–
–
–
–
–
–
–
–
–
–
–
–
–
–
–
–
–
–
–
–
–
–
–
–
–
–
–
–
–
–

Subset Id	Control Group	Reference Gene

Table of Original Data

Well	Subset Id	Sample Name	Gene	Reporter	Ct	
	D2	S I	CONTROL	ALP	SYBR GREEN	24.1119
	D3	S I	CONTROL	ALP	SYBR GREEN	24.0336
	D4	S I	CONTROL	ALP	SYBR GREEN	24.2387
	A2	S I	CONTROL	OCN	SYBR GREEN	18.7099
	A3	S I	CONTROL	OCN	SYBR GREEN	18.645
	A4	S I	CONTROL	OCN	SYBR GREEN	18.5677
	E2	S I	CONTROL	OPN	SYBR GREEN	21.4938
	E3	S I	CONTROL	OPN	SYBR GREEN	21.6919
	E4	S I	CONTROL	OPN	SYBR GREEN	21.6231
	B2	S I	CONTROL	RUNX2	SYBR GREEN	17.6553
	B3	S I	CONTROL	RUNX2	SYBR GREEN	17.7305
	B4	S I	CONTROL	RUNX2	SYBR GREEN	17.9356
	C2	S I	CONTROL	actin	SYBR GREEN	12.1268
	C3	S I	CONTROL	actin	SYBR GREEN	12.1225
	C4	S I	CONTROL	actin	SYBR GREEN	12.2978
	D5	S I	GLUCOSE	ALP	SYBR GREEN	24.6053
	D6	S I	GLUCOSE	ALP	SYBR GREEN	24.6631
	D7	S I	GLUCOSE	ALP	SYBR GREEN	24.324
	A5	S I	GLUCOSE	OCN	SYBR GREEN	19.1102
	A6	S I	GLUCOSE	OCN	SYBR GREEN	18.8736
	A7	S I	GLUCOSE	OCN	SYBR GREEN	19.1175
	E5	S I	GLUCOSE	OPN	SYBR GREEN	22.1365
	E6	S I	GLUCOSE	OPN	SYBR GREEN	22.2826
	E7	S I	GLUCOSE	OPN	SYBR GREEN	22.0118
	B5	S I	GLUCOSE	RUNX2	SYBR GREEN	17.8151
	B6	S I	GLUCOSE	RUNX2	SYBR GREEN	18.0131
	B7	S I	GLUCOSE	RUNX2	SYBR GREEN	17.6387
	C5	S I	GLUCOSE	actin	SYBR GREEN	11.1088
	C6	S I	GLUCOSE	actin	SYBR GREEN	11.5179
	C7	S I	GLUCOSE	actin	SYBR GREEN	11.1717
	D8	S I	GLUCOSE+XLGB	ALP	SYBR GREEN	23.8423
	D9	S I	GLUCOSE+XLGB	ALP	SYBR GREEN	23.8155
	D10	S I	GLUCOSE+XLGB	ALP	SYBR GREEN	23.638
	A8	S I	GLUCOSE+XLGB	OCN	SYBR GREEN	18.3962
	A9	S I	GLUCOSE+XLGB	OCN	SYBR GREEN	18.3551
	A10	S I	GLUCOSE+XLGB	OCN	SYBR GREEN	18.2085
	E8	S I	GLUCOSE+XLGB	OPN	SYBR GREEN	20.9815
	E9	S I	GLUCOSE+XLGB	OPN	SYBR GREEN	20.8743
	E10	S I	GLUCOSE+XLGB	OPN	SYBR GREEN	20.9495
	B8	S I	GLUCOSE+XLGB	RUNX2	SYBR GREEN	17.4136
	B9	S I	GLUCOSE+XLGB	RUNX2	SYBR GREEN	17.3673
	B10	S I	GLUCOSE+XLGB	RUNX2	SYBR GREEN	17.5613
	C8	S I	GLUCOSE+XLGB	actin	SYBR GREEN	11.981
	C9	S I	GLUCOSE+XLGB	actin	SYBR GREEN	11.8032
	C10	S I	GLUCOSE+XLGB	actin	SYBR GREEN	11.4552

Table of RQ

Subset Id	Control	Delta Ct	Sample	Target Gene	Delta Ct	DD Ct

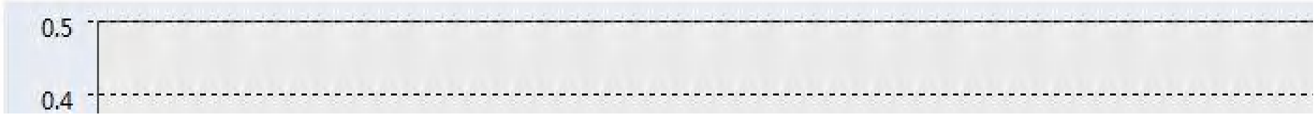

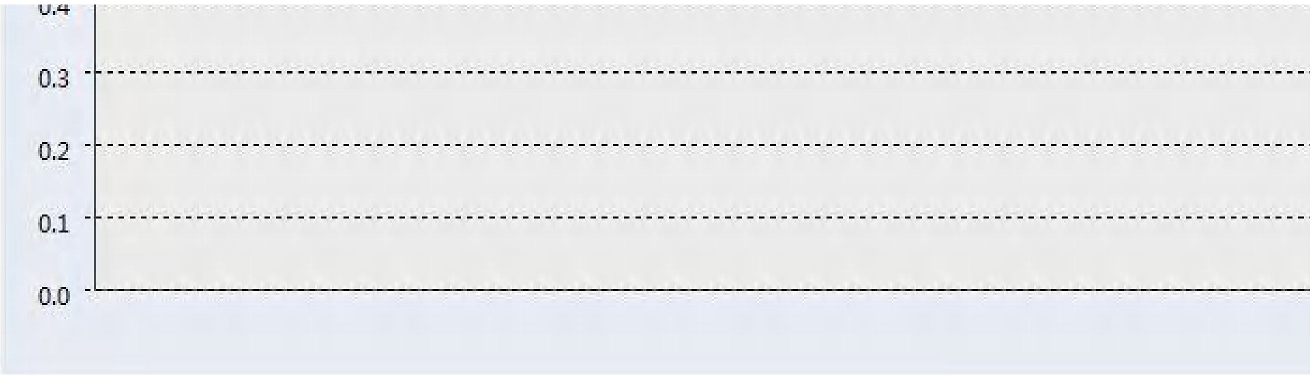
0.4
0.3
0.2
0.1
0.0

RQ	RQ max	RQ min

组织名称	编号	OCN	actin	内参平均值	C-E	F最大平均	F-G	函数POWER	I平均值
CONTROL	1	18.71	12.13	12.18333	6.526667	6.456667	0.07	0.952638	1.000785
CONTROL	2	18.64	12.12	12.18333	6.456667	6.456667	0	1	
CONTROL	3	18.57	12.3	12.18333	6.386667	6.456667	-0.07	1.049717	
GLUCOSE	1	19.11	11.11	11.26667	7.843333	6.456667	1.386667	0.382447	0.40464
GLUCOSE	2	18.87	11.52	11.26667	7.603333	6.456667	1.146667	0.451668	
GLUCOSE	3	19.12	11.17	11.26667	7.853333	6.456667	1.396667	0.379806	
GLUCOSE+XI	1	18.4	11.98	11.74667	6.653333	6.456667	0.196667	0.872564	0.921683
GLUCOSE+XI	2	18.36	11.8	11.74667	6.613333	6.456667	0.156667	0.897095	
GLUCOSE+XI	3	18.21	11.46	11.74667	6.463333	6.456667	0.006667	0.99539	

组织名称	编号	RUNX2	actin	内参平均值	C-E	F最大平均	F-G	函数POWER	I平均值
CONTROL	1	17.66	12.13	12.18333	5.476667	5.593333	-0.11667	1.084227	1.003354
CONTROL	2	17.73	12.12	12.18333	5.546667	5.593333	-0.04667	1.032876	
CONTROL	3	17.94	12.3	12.18333	5.756667	5.593333	0.163333	0.89296	
GLUCOSE	1	17.82	11.11	11.26667	6.553333	5.593333	0.96	0.514057	0.515683
GLUCOSE	2	18.01	11.52	11.26667	6.743333	5.593333	1.15	0.450625	
GLUCOSE	3	17.64	11.17	11.26667	6.373333	5.593333	0.78	0.582367	
GLUCOSE+XI	1	17.41	11.98	11.74667	5.663333	5.593333	0.07	0.952638	0.930208
GLUCOSE+XI	2	17.37	11.8	11.74667	5.623333	5.593333	0.03	0.97942	
GLUCOSE+XI	3	17.56	11.46	11.74667	5.813333	5.593333	0.22	0.858565	

组织名称	编号	ALP	actin	内参平均值	C-E	F最大平均	F-G	函数POWER	I平均值
CONTROL	1	24.11	12.13	12.18333	11.92667	11.94333	-0.01667	1.011619	1.00179
CONTROL	2	24.03	12.12	12.18333	11.84667	11.94333	-0.09667	1.0693	
CONTROL	3	24.24	12.3	12.18333	12.05667	11.94333	0.113333	0.92445	
GLUCOSE	1	24.61	11.11	11.26667	13.34333	11.94333	1.4	0.378929	0.402748
GLUCOSE	2	24.66	11.52	11.26667	13.39333	11.94333	1.45	0.366021	
GLUCOSE	3	24.32	11.17	11.26667	13.05333	11.94333	1.11	0.463294	
GLUCOSE+XI	1	23.84	11.98	11.74667	12.09333	11.94333	0.15	0.90125	0.950116
GLUCOSE+XI	2	23.82	11.8	11.74667	12.07333	11.94333	0.13	0.913831	
GLUCOSE+XI	3	23.64	11.46	11.74667	11.89333	11.94333	-0.05	1.035265	

组织名称	编号	OPN	actin	内参平均值	C-E	F最大平均	F-G	函数POWER	I平均值
CONTROL	1	21.49	12.13	12.18333	9.306667	9.416667	-0.11	1.079228	1.001661
CONTROL	2	21.69	12.12	12.18333	9.506667	9.416667	0.09	0.939523	
CONTROL	3	21.62	12.3	12.18333	9.436667	9.416667	0.02	0.986233	
GLUCOSE	1	22.14	11.11	11.26667	10.87333	9.416667	1.456667	0.364334	0.364554
GLUCOSE	2	22.28	11.52	11.26667	11.01333	9.416667	1.596667	0.33064	
GLUCOSE	3	22.01	11.17	11.26667	10.74333	9.416667	1.326667	0.398688	
GLUCOSE+XI	1	20.98	11.98	11.74667	9.233333	9.416667	-0.18333	1.135504	1.173446
GLUCOSE+XI	2	20.87	11.8	11.74667	9.123333	9.416667	-0.29333	1.225468	
GLUCOSE+XI	3	20.95	11.46	11.74667	9.203333	9.416667	-0.21333	1.159364	

ALP	1.00179	0.072924	3	0.402748	0.05283	3	0.950116	0.074009	3
OCN	1.000785	0.048544	3	0.40464	0.040748	3	0.921683	0.065	3
OPN	1.001661	0.071119	3	0.364554	0.034025	3	1.173446	0.046606	3

RUNX2	1.003354	0.098992	3	0.515683	0.065886	3	0.930208	0.063473	3

函数STDEV
0.048544

0.040748

0.065

函数STDEV
0.098992

0.065886

0.063473

函数STDEV
0.072924

0.05283

0.074009

函数STDEV
0.071119

0.034025

0.046606

Experiment Information

Name	P_210910
Date	44449
Operator	admin
Location	F:\Funglyn\FTC-3000\experiments\admin
Template	F:\Funglyn\FTC-3000\templates\SYBR Green 两步法.tmt
Tips	

Protocol

Stage:Hold Cycles:1

Target	Hold(H:M:S)	Ramp Rate(C/s)	Touch Down	Multiple Temp	Singnal Acq.
95	0.416666667				

Stage:Cycles Cycles:40

Target	Hold(H:M:S)	Ramp Rate(C/s)	Touch Down	Multiple Temp	Singnal Acq.
95	0.010416667				
60	0.041666667				TRUE

Stage:Dissociation

Target	Hold(H:M:S)	Ramp Rate(C/s)	Touch Down	Multiple Temp	Singnal Acq.
94	0.0625				
60	0.125	0.1			
94	0.006944444				TRUE

Plate

Well	Reporter	Subset ID	Sample Name	Gene	Sample Type	Quantity
B2	SYBR GREEN	S I	CONTROL	OPG	Unknown Sample	
B3	SYBR GREEN	S I	CONTROL	OPG	Unknown Sample	
B4	SYBR GREEN	S I	CONTROL	OPG	Unknown Sample	
B5	SYBR GREEN	S I	GLUCOSE	OPG	Unknown Sample	
B6	SYBR GREEN	S I	GLUCOSE	OPG	Unknown Sample	
B7	SYBR GREEN	S I	GLUCOSE	OPG	Unknown Sample	
B8	SYBR GREEN	S I	GLUCOSE+XLGB	OPG	Unknown Sample	
B9	SYBR GREEN	S I	GLUCOSE+XLGB	OPG	Unknown Sample	
B10	SYBR GREEN	S I	GLUCOSE+XLGB	OPG	Unknown Sample	
C2	SYBR GREEN	S I	CONTROL	OPGL	Unknown Sample	
C3	SYBR GREEN	S I	CONTROL	OPGL	Unknown Sample	
C4	SYBR GREEN	S I	CONTROL	OPGL	Unknown Sample	
C5	SYBR GREEN	S I	GLUCOSE	OPGL	Unknown Sample	
C6	SYBR GREEN	S I	GLUCOSE	OPGL	Unknown Sample	
C7	SYBR GREEN	S I	GLUCOSE	OPGL	Unknown Sample	
C8	SYBR GREEN	S I	GLUCOSE+XLGB	OPGL	Unknown Sample	
C9	SYBR GREEN	S I	GLUCOSE+XLGB	OPGL	Unknown Sample	
C10	SYBR GREEN	S I	GLUCOSE+XLGB	OPGL	Unknown Sample	
D2	SYBR GREEN	S I	CONTROL	ACTIN	Unknown Sample	
D3	SYBR GREEN	S I	CONTROL	ACTIN	Unknown Sample	
D4	SYBR GREEN	S I	CONTROL	ACTIN	Unknown Sample	
D5	SYBR GREEN	S I	GLUCOSE	ACTIN	Unknown Sample	
D6	SYBR GREEN	S I	GLUCOSE	ACTIN	Unknown Sample	
D7	SYBR GREEN	S I	GLUCOSE	ACTIN	Unknown Sample	
D8	SYBR GREEN	S I	GLUCOSE+XLGB	ACTIN	Unknown Sample	
D9	SYBR GREEN	S I	GLUCOSE+XLGB	ACTIN	Unknown Sample	
D10	SYBR GREEN	S I	GLUCOSE+XLGB	ACTIN	Unknown Sample	

Data

Well	Subset ID	Reporter	S_Name	S_Type	Ct	Mean Ct
B2	S I	SYBR GREEN	CONTROL	Unknown Sample	27.04	27.02
B3	S I	SYBR GREEN	CONTROL	Unknown Sample	27.08	27.02
B4	S I	SYBR GREEN	CONTROL	Unknown Sample	26.93	27.02
B5	S I	SYBR GREEN	GLUCOSE	Unknown Sample	28.37	28.55
B6	S I	SYBR GREEN	GLUCOSE	Unknown Sample	28.85	28.55
B7	S I	SYBR GREEN	GLUCOSE	Unknown Sample	28.43	28.55
B8	S I	SYBR GREEN	GLUCOSE+XLGB	Unknown Sample	26.59	26.47
B9	S I	SYBR GREEN	GLUCOSE+XLGB	Unknown Sample	26.39	26.47
B10	S I	SYBR GREEN	GLUCOSE+XLGB	Unknown Sample	26.44	26.47
C2	S I	SYBR GREEN	CONTROL	Unknown Sample	28.28	28.39
C3	S I	SYBR GREEN	CONTROL	Unknown Sample	28.4	28.39
C4	S I	SYBR GREEN	CONTROL	Unknown Sample	28.49	28.39
C5	S I	SYBR GREEN	GLUCOSE	Unknown Sample	27.16	27.26
C6	S I	SYBR GREEN	GLUCOSE	Unknown Sample	27.4	27.26
C7	S I	SYBR GREEN	GLUCOSE	Unknown Sample	27.23	27.26
C8	S I	SYBR GREEN	GLUCOSE+XLGB	Unknown Sample	27.65	27.59
C9	S I	SYBR GREEN	GLUCOSE+XLGB	Unknown Sample	27.4	27.59
C10	S I	SYBR GREEN	GLUCOSE+XLGB	Unknown Sample	27.72	27.59
D2	S I	SYBR GREEN	CONTROL	Unknown Sample	13.31	13.56
D3	S I	SYBR GREEN	CONTROL	Unknown Sample	13.68	13.56
D4	S I	SYBR GREEN	CONTROL	Unknown Sample	13.7	13.56
D5	S I	SYBR GREEN	GLUCOSE	Unknown Sample	13.5	13.36
D6	S I	SYBR GREEN	GLUCOSE	Unknown Sample	13.18	13.36
D7	S I	SYBR GREEN	GLUCOSE	Unknown Sample	13.42	13.36
D8	S I	SYBR GREEN	GLUCOSE+XLGB	Unknown Sample	13.14	13.12
D9	S I	SYBR GREEN	GLUCOSE+XLGB	Unknown Sample	13.13	13.12
D10	S I	SYBR GREEN	GLUCOSE+XLGB	Unknown Sample	13.09	13.12

Graph

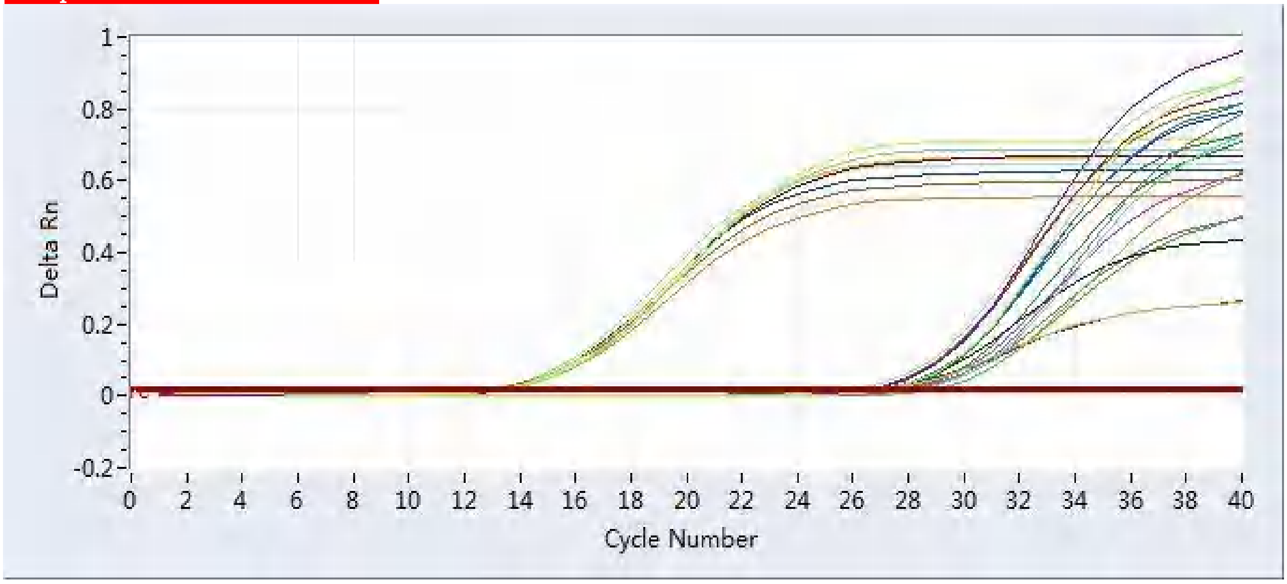

StdDev Ct	Quantity	Gene
0.08	-	OPG
0.08	-	OPG
0.08	-	OPG
0.26	-	OPG
0.26	-	OPG
0.26	-	OPG
0.1	-	OPG
0.1	-	OPG
0.1	-	OPG
0.11	-	OPGL
0.11	-	OPGL
0.11	-	OPGL
0.12	-	OPGL
0.12	-	OPGL
0.12	-	OPGL
0.17	-	OPGL
0.17	-	OPGL
0.17	-	OPGL
0.22	-	ACTIN
0.22	-	ACTIN
0.22	-	ACTIN
0.17	-	ACTIN
0.17	-	ACTIN
0.17	-	ACTIN
0.03	-	ACTIN
0.03	-	ACTIN
0.03	-	ACTIN

Data

Well	Subset Id	Reporter	Sample Name	Gene	Tm1	Tm2
B2	S I	SYBR GREEN	CONTROL	OPG	81.12	-
B3	S I	SYBR GREEN	CONTROL	OPG	81.34	-
B4	S I	SYBR GREEN	CONTROL	OPG	81.08	-
B5	S I	SYBR GREEN	GLUCOSE	OPG	81.4	-
B6	S I	SYBR GREEN	GLUCOSE	OPG	80.76	-
B7	S I	SYBR GREEN	GLUCOSE	OPG	80.72	-
B8	S I	SYBR GREEN	GLUCOSE+XLGB	OPG	81.15	-
B9	S I	SYBR GREEN	GLUCOSE+XLGB	OPG	81.27	-
B10	S I	SYBR GREEN	GLUCOSE+XLGB	OPG	81.48	-
C2	S I	SYBR GREEN	CONTROL	OPGL	81.06	-
C3	S I	SYBR GREEN	CONTROL	OPGL	81.07	-
C4	S I	SYBR GREEN	CONTROL	OPGL	81.08	-
C5	S I	SYBR GREEN	GLUCOSE	OPGL	81.24	-
C6	S I	SYBR GREEN	GLUCOSE	OPGL	81.55	-
C7	S I	SYBR GREEN	GLUCOSE	OPGL	81.58	-
C8	S I	SYBR GREEN	GLUCOSE+XLGB	OPGL	81.59	-
C9	S I	SYBR GREEN	GLUCOSE+XLGB	OPGL	80.82	-
C10	S I	SYBR GREEN	GLUCOSE+XLGB	OPGL	80.79	-
D2	S I	SYBR GREEN	CONTROL	ACTIN	83.38	-
D3	S I	SYBR GREEN	CONTROL	ACTIN	83.74	-
D4	S I	SYBR GREEN	CONTROL	ACTIN	83.43	-
D5	S I	SYBR GREEN	GLUCOSE	ACTIN	83.77	-
D6	S I	SYBR GREEN	GLUCOSE	ACTIN	82.52	-
D7	S I	SYBR GREEN	GLUCOSE	ACTIN	82.53	-
D8	S I	SYBR GREEN	GLUCOSE+XLGB	ACTIN	83.52	-
D9	S I	SYBR GREEN	GLUCOSE+XLGB	ACTIN	82.95	-
D10	S I	SYBR GREEN	GLUCOSE+XLGB	ACTIN	82.84	-

Rn Curve

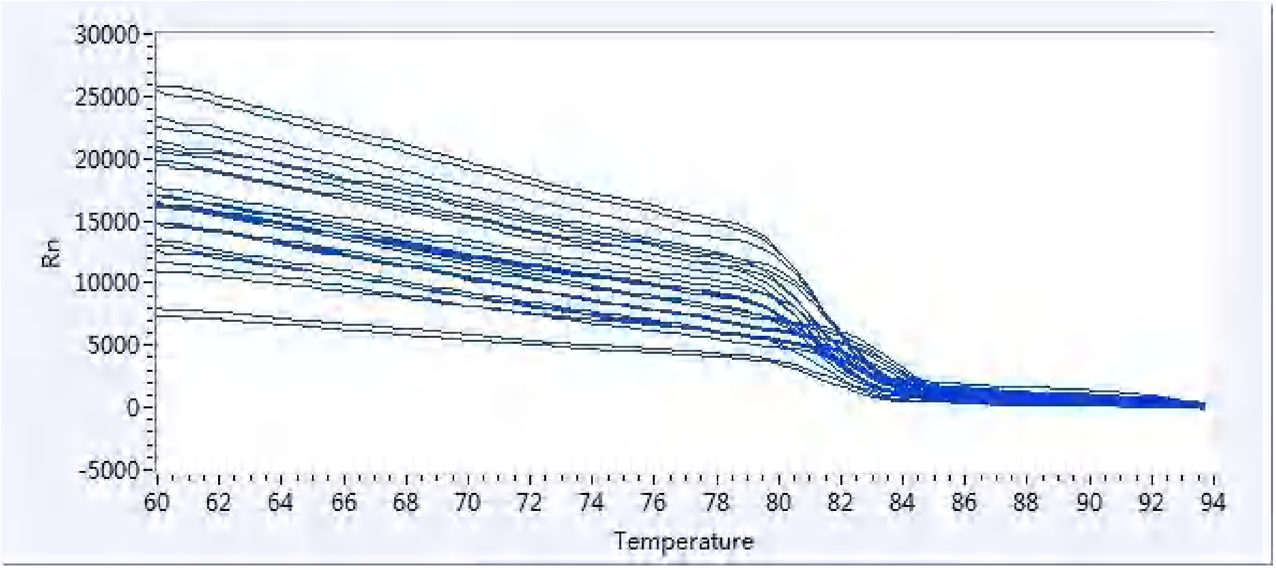

-(di/dt) Curve

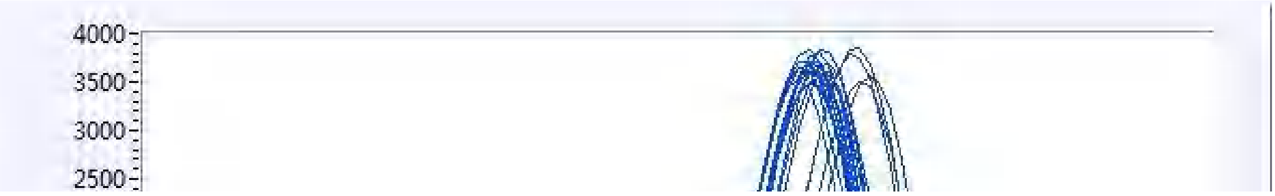

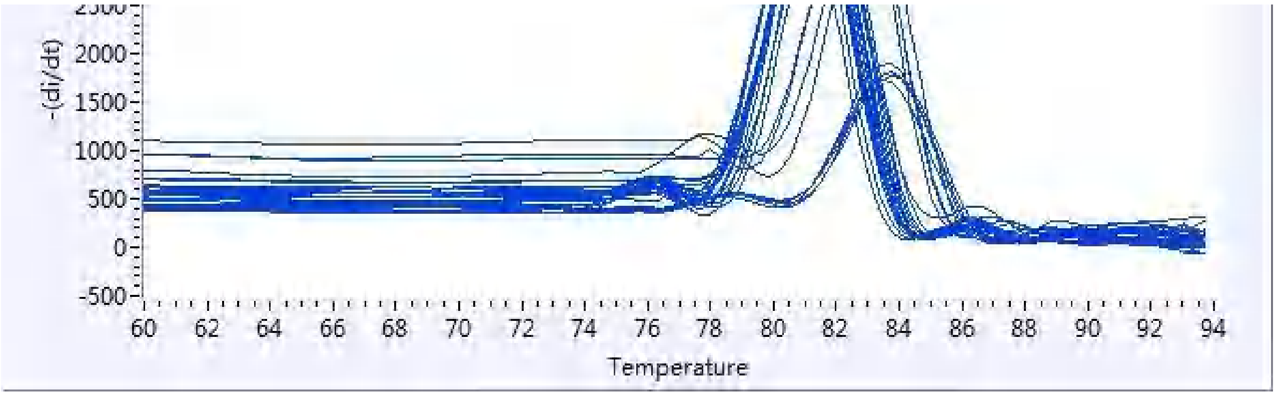
-(dI/dt)
2500
2000
1500
1000
500
0
-500
60 62 64 66 68 70 72 74 76 78 80 82 84 86 88 90 92 94
Temperature

–
–
–
–
–
–
–
–
–
–
–
–
–
–
–
–
–
–
–
–
–
–
–
–
–
–
–

Subset Id	Control Group	Reference Gene

Table of Original Data

Well	Subset Id	Sample Name	Gene	Reporter	Ct	
	D2	S I	CONTROL	ACTIN	SYBR GREEN	13.3094
	D3	S I	CONTROL	ACTIN	SYBR GREEN	13.6752
	D4	S I	CONTROL	ACTIN	SYBR GREEN	13.6995
	B2	S I	CONTROL	OPG	SYBR GREEN	27.0439
	B3	S I	CONTROL	OPG	SYBR GREEN	27.0778
	B4	S I	CONTROL	OPG	SYBR GREEN	26.9265
	C2	S I	CONTROL	OPGL	SYBR GREEN	28.2776
	C3	S I	CONTROL	OPGL	SYBR GREEN	28.3954
	C4	S I	CONTROL	OPGL	SYBR GREEN	28.4947
	D5	S I	GLUCOSE	ACTIN	SYBR GREEN	13.4971
	D6	S I	GLUCOSE	ACTIN	SYBR GREEN	13.1771
	D7	S I	GLUCOSE	ACTIN	SYBR GREEN	13.4159
	B5	S I	GLUCOSE	OPG	SYBR GREEN	28.3728
	B6	S I	GLUCOSE	OPG	SYBR GREEN	28.8521
	B7	S I	GLUCOSE	OPG	SYBR GREEN	28.4328
	C5	S I	GLUCOSE	OPGL	SYBR GREEN	27.1565
	C6	S I	GLUCOSE	OPGL	SYBR GREEN	27.4002
	C7	S I	GLUCOSE	OPGL	SYBR GREEN	27.2304
	D8	S I	GLUCOSE+XLGB	ACTIN	SYBR GREEN	13.1369
	D9	S I	GLUCOSE+XLGB	ACTIN	SYBR GREEN	13.1258
	D10	S I	GLUCOSE+XLGB	ACTIN	SYBR GREEN	13.0887
	B8	S I	GLUCOSE+XLGB	OPG	SYBR GREEN	26.5913
	B9	S I	GLUCOSE+XLGB	OPG	SYBR GREEN	26.3853
	B10	S I	GLUCOSE+XLGB	OPG	SYBR GREEN	26.4361
	C8	S I	GLUCOSE+XLGB	OPGL	SYBR GREEN	27.6486
	C9	S I	GLUCOSE+XLGB	OPGL	SYBR GREEN	27.3989
	C10	S I	GLUCOSE+XLGB	OPGL	SYBR GREEN	27.7184

Table of RQ

Subset Id	Control	Delta Ct	Sample	Target Gene	Delta Ct	DD Ct

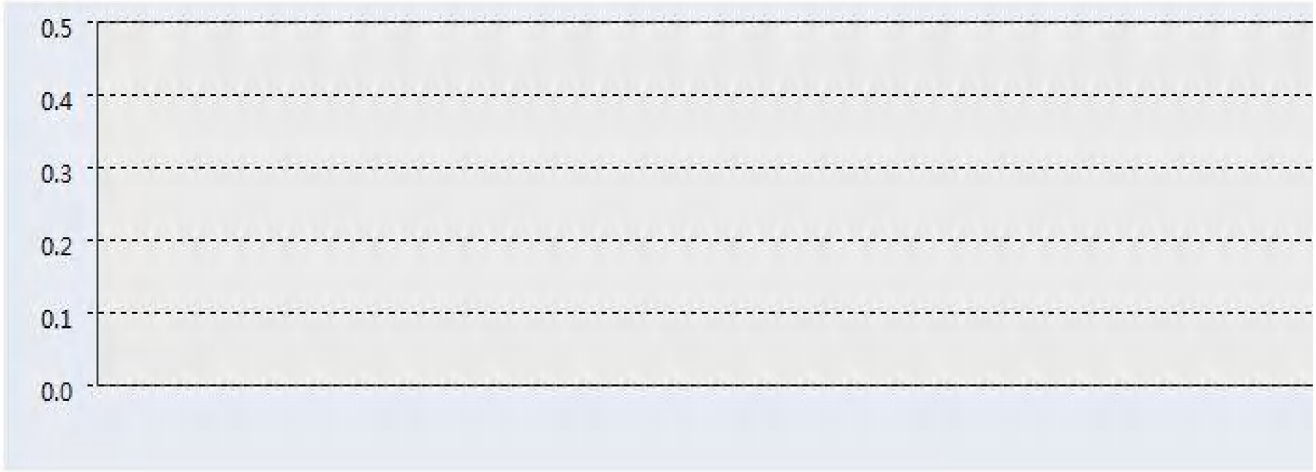

RQ

组织名称	编号	OPG	内参CT值	内参平均值	C-E	F最大平均	F-G	函数POWER	I平均值
CONTROL	1	27.04	13.31	13.56333	13.47667	13.45333	0.023333	0.983957	1.000974
CONTROL	2	27.08	13.68	13.56333	13.51667	13.45333	0.063333	0.95705	
CONTROL	3	26.93	13.7	13.56333	13.36667	13.45333	-0.08667	1.061914	
GLUCOSE	1	28.37	13.5	13.36667	15.00333	13.45333	1.55	0.34151	0.304654
GLUCOSE	2	28.85	13.18	13.36667	15.48333	13.45333	2.03	0.244855	
GLUCOSE	3	28.43	13.42	13.36667	15.06333	13.45333	1.61	0.327598	
GLUCOSE+XI	1	26.59	13.14	13.12	13.47	13.45333	0.016667	0.988514	1.073614
GLUCOSE+XI	2	26.39	13.13	13.12	13.27	13.45333	-0.18333	1.135504	
GLUCOSE+XI	3	26.44	13.09	13.12	13.32	13.45333	-0.13333	1.096825	

组织名称	编号	OPGL	内参CT值	内参平均值	C-E	F最大平均	F-G	函数POWER	I平均值
CONTROL	1	28.28	13.31	13.56333	14.71667	14.82667	-0.11	1.079228	1.001785
CONTROL	2	28.4	13.68	13.56333	14.83667	14.82667	0.01	0.993092	
CONTROL	3	28.49	13.7	13.56333	14.92667	14.82667	0.1	0.933033	
GLUCOSE	1	27.16	13.5	13.36667	13.79333	14.82667	-1.03333	2.046748	1.909877
GLUCOSE	2	27.4	13.18	13.36667	14.03333	14.82667	-0.79333	1.733074	
GLUCOSE	3	27.23	13.42	13.36667	13.86333	14.82667	-0.96333	1.94981	
GLUCOSE+XI	1	27.65	13.14	13.12	14.53	14.82667	-0.29667	1.228303	1.286379
GLUCOSE+XI	2	27.4	13.13	13.12	14.28	14.82667	-0.54667	1.460707	
GLUCOSE+XI	3	27.72	13.09	13.12	14.6	14.82667	-0.22667	1.170128	

OPG	1.000974	0.054463	3	0.304654	0.052253	3	1.073614	0.076194	3
OPGL	1.001785	0.073484	3	1.909877	0.160604	3	1.286379	0.153749	3

函数STDEV
0.054463

0.052253

0.076194

函数STDEV
0.073484

0.160604

0.153749

Experiment Information

Name	P_210910
Date	44449
Operator	admin
Location	F:\Funglyn\FTC-3000\experiments\admin
Template	F:\Funglyn\FTC-3000\templates\SYBR Green 两步法.tmt
Tips	

Protocol

Stage:Hold Cycles:1

Target	Hold(H:M:S)	Ramp Rate(C/s)	Touch Down	Multiple Temp	Singnal Acq.
95	0.416666667				

Stage:Cycles Cycles:40

Target	Hold(H:M:S)	Ramp Rate(C/s)	Touch Down	Multiple Temp	Singnal Acq.
95	0.010416667				
60	0.041666667				TRUE

Stage:Dissociation

Target	Hold(H:M:S)	Ramp Rate(C/s)	Touch Down	Multiple Temp	Singnal Acq.
94	0.0625				
60	0.125	0.1			
94	0.006944444				TRUE

Plate

Well	Reporter	Subset ID	Sample Name	Gene	Sample Type	Quantity
B2	SYBR GREEN	S I	CONTROL	OPG	Unknown Sample	
B3	SYBR GREEN	S I	CONTROL	OPG	Unknown Sample	
B4	SYBR GREEN	S I	CONTROL	OPG	Unknown Sample	
B5	SYBR GREEN	S I	GLUCOSE	OPG	Unknown Sample	
B6	SYBR GREEN	S I	GLUCOSE	OPG	Unknown Sample	
B7	SYBR GREEN	S I	GLUCOSE	OPG	Unknown Sample	
B8	SYBR GREEN	S I	GLUCOSE+XLGB	OPG	Unknown Sample	
B9	SYBR GREEN	S I	GLUCOSE+XLGB	OPG	Unknown Sample	
B10	SYBR GREEN	S I	GLUCOSE+XLGB	OPG	Unknown Sample	
C2	SYBR GREEN	S I	CONTROL	OPGL	Unknown Sample	
C3	SYBR GREEN	S I	CONTROL	OPGL	Unknown Sample	
C4	SYBR GREEN	S I	CONTROL	OPGL	Unknown Sample	
C5	SYBR GREEN	S I	GLUCOSE	OPGL	Unknown Sample	
C6	SYBR GREEN	S I	GLUCOSE	OPGL	Unknown Sample	
C7	SYBR GREEN	S I	GLUCOSE	OPGL	Unknown Sample	
C8	SYBR GREEN	S I	GLUCOSE+XLGB	OPGL	Unknown Sample	
C9	SYBR GREEN	S I	GLUCOSE+XLGB	OPGL	Unknown Sample	
C10	SYBR GREEN	S I	GLUCOSE+XLGB	OPGL	Unknown Sample	
D2	SYBR GREEN	S I	CONTROL	ACTIN	Unknown Sample	
D3	SYBR GREEN	S I	CONTROL	ACTIN	Unknown Sample	
D4	SYBR GREEN	S I	CONTROL	ACTIN	Unknown Sample	
D5	SYBR GREEN	S I	GLUCOSE	ACTIN	Unknown Sample	
D6	SYBR GREEN	S I	GLUCOSE	ACTIN	Unknown Sample	
D7	SYBR GREEN	S I	GLUCOSE	ACTIN	Unknown Sample	
D8	SYBR GREEN	S I	GLUCOSE+XLGB	ACTIN	Unknown Sample	
D9	SYBR GREEN	S I	GLUCOSE+XLGB	ACTIN	Unknown Sample	
D10	SYBR GREEN	S I	GLUCOSE+XLGB	ACTIN	Unknown Sample	

Data

Well	Subset ID	Reporter	S_Name	S_Type	Ct	Mean Ct
B2	S I	SYBR GREEN	CONTROL	Unknown Sample	27.04	27.02
B3	S I	SYBR GREEN	CONTROL	Unknown Sample	27.08	27.02
B4	S I	SYBR GREEN	CONTROL	Unknown Sample	26.93	27.02
B5	S I	SYBR GREEN	GLUCOSE	Unknown Sample	28.37	28.55
B6	S I	SYBR GREEN	GLUCOSE	Unknown Sample	28.85	28.55
B7	S I	SYBR GREEN	GLUCOSE	Unknown Sample	28.43	28.55
B8	S I	SYBR GREEN	GLUCOSE+XLGB	Unknown Sample	26.59	26.47
B9	S I	SYBR GREEN	GLUCOSE+XLGB	Unknown Sample	26.39	26.47
B10	S I	SYBR GREEN	GLUCOSE+XLGB	Unknown Sample	26.44	26.47
C2	S I	SYBR GREEN	CONTROL	Unknown Sample	28.28	28.39
C3	S I	SYBR GREEN	CONTROL	Unknown Sample	28.4	28.39
C4	S I	SYBR GREEN	CONTROL	Unknown Sample	28.49	28.39
C5	S I	SYBR GREEN	GLUCOSE	Unknown Sample	27.16	27.26
C6	S I	SYBR GREEN	GLUCOSE	Unknown Sample	27.4	27.26
C7	S I	SYBR GREEN	GLUCOSE	Unknown Sample	27.23	27.26
C8	S I	SYBR GREEN	GLUCOSE+XLGB	Unknown Sample	27.65	27.59
C9	S I	SYBR GREEN	GLUCOSE+XLGB	Unknown Sample	27.4	27.59
C10	S I	SYBR GREEN	GLUCOSE+XLGB	Unknown Sample	27.72	27.59
D2	S I	SYBR GREEN	CONTROL	Unknown Sample	13.31	13.56
D3	S I	SYBR GREEN	CONTROL	Unknown Sample	13.68	13.56
D4	S I	SYBR GREEN	CONTROL	Unknown Sample	13.7	13.56
D5	S I	SYBR GREEN	GLUCOSE	Unknown Sample	13.5	13.36
D6	S I	SYBR GREEN	GLUCOSE	Unknown Sample	13.18	13.36
D7	S I	SYBR GREEN	GLUCOSE	Unknown Sample	13.42	13.36
D8	S I	SYBR GREEN	GLUCOSE+XLGB	Unknown Sample	13.14	13.12
D9	S I	SYBR GREEN	GLUCOSE+XLGB	Unknown Sample	13.13	13.12
D10	S I	SYBR GREEN	GLUCOSE+XLGB	Unknown Sample	13.09	13.12

Graph

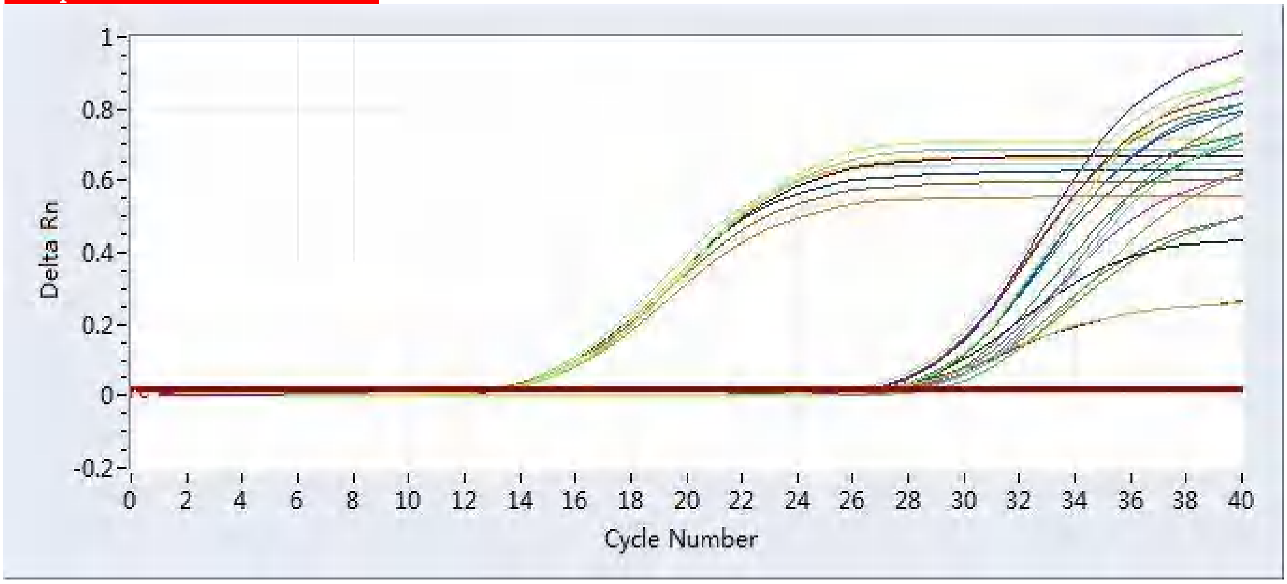

StdDev Ct	Quantity	Gene
0.08	-	OPG
0.08	-	OPG
0.08	-	OPG
0.26	-	OPG
0.26	-	OPG
0.26	-	OPG
0.1	-	OPG
0.1	-	OPG
0.1	-	OPG
0.11	-	OPGL
0.11	-	OPGL
0.11	-	OPGL
0.12	-	OPGL
0.12	-	OPGL
0.12	-	OPGL
0.17	-	OPGL
0.17	-	OPGL
0.17	-	OPGL
0.22	-	ACTIN
0.22	-	ACTIN
0.22	-	ACTIN
0.17	-	ACTIN
0.17	-	ACTIN
0.17	-	ACTIN
0.03	-	ACTIN
0.03	-	ACTIN
0.03	-	ACTIN

Data

Well	Subset Id	Reporter	Sample Name	Gene	Tm1	Tm2
B2	S I	SYBR GREEN	CONTROL	OPG	81.12	-
B3	S I	SYBR GREEN	CONTROL	OPG	81.34	-
B4	S I	SYBR GREEN	CONTROL	OPG	81.08	-
B5	S I	SYBR GREEN	GLUCOSE	OPG	81.4	-
B6	S I	SYBR GREEN	GLUCOSE	OPG	80.76	-
B7	S I	SYBR GREEN	GLUCOSE	OPG	80.72	-
B8	S I	SYBR GREEN	GLUCOSE+XLGB	OPG	81.15	-
B9	S I	SYBR GREEN	GLUCOSE+XLGB	OPG	81.27	-
B10	S I	SYBR GREEN	GLUCOSE+XLGB	OPG	81.48	-
C2	S I	SYBR GREEN	CONTROL	OPGL	81.06	-
C3	S I	SYBR GREEN	CONTROL	OPGL	81.07	-
C4	S I	SYBR GREEN	CONTROL	OPGL	81.08	-
C5	S I	SYBR GREEN	GLUCOSE	OPGL	81.24	-
C6	S I	SYBR GREEN	GLUCOSE	OPGL	81.55	-
C7	S I	SYBR GREEN	GLUCOSE	OPGL	81.58	-
C8	S I	SYBR GREEN	GLUCOSE+XLGB	OPGL	81.59	-
C9	S I	SYBR GREEN	GLUCOSE+XLGB	OPGL	80.82	-
C10	S I	SYBR GREEN	GLUCOSE+XLGB	OPGL	80.79	-
D2	S I	SYBR GREEN	CONTROL	ACTIN	83.38	-
D3	S I	SYBR GREEN	CONTROL	ACTIN	83.74	-
D4	S I	SYBR GREEN	CONTROL	ACTIN	83.43	-
D5	S I	SYBR GREEN	GLUCOSE	ACTIN	83.77	-
D6	S I	SYBR GREEN	GLUCOSE	ACTIN	82.52	-
D7	S I	SYBR GREEN	GLUCOSE	ACTIN	82.53	-
D8	S I	SYBR GREEN	GLUCOSE+XLGB	ACTIN	83.52	-
D9	S I	SYBR GREEN	GLUCOSE+XLGB	ACTIN	82.95	-
D10	S I	SYBR GREEN	GLUCOSE+XLGB	ACTIN	82.84	-

Rn Curve

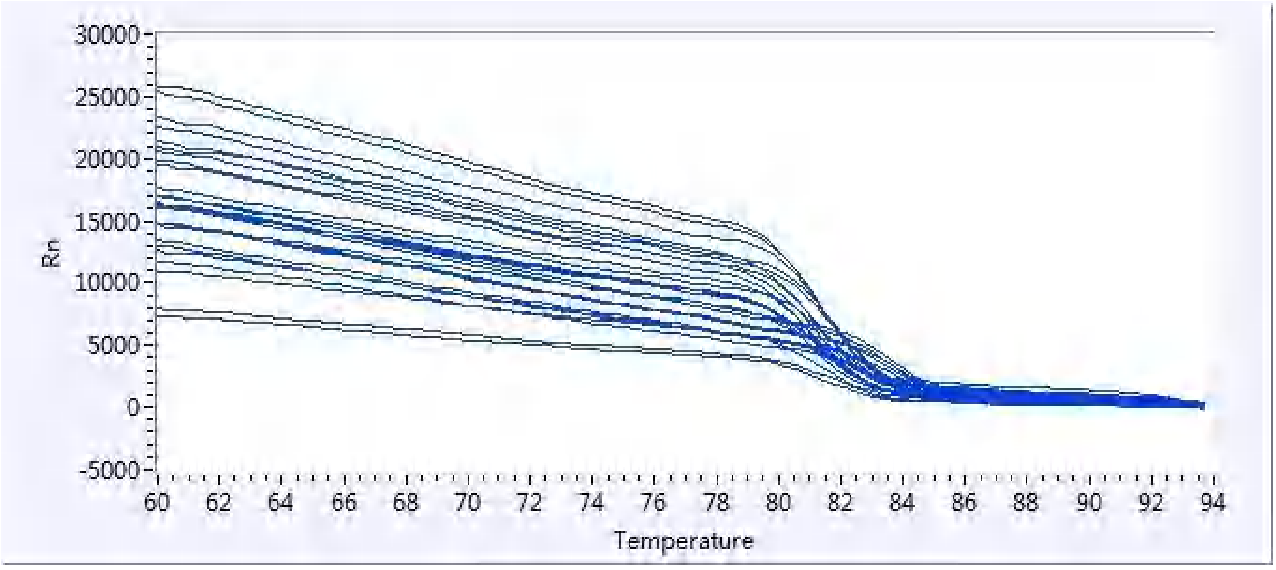

-(di/dt) Curve

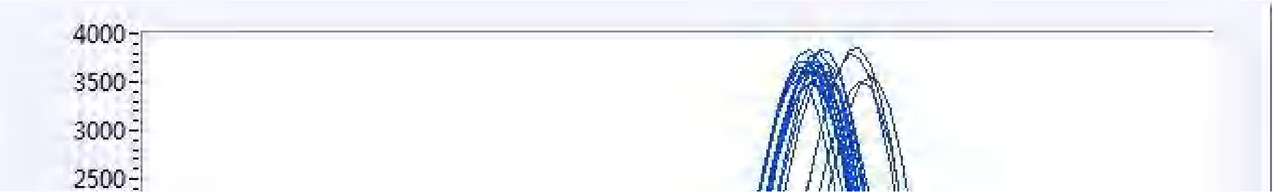

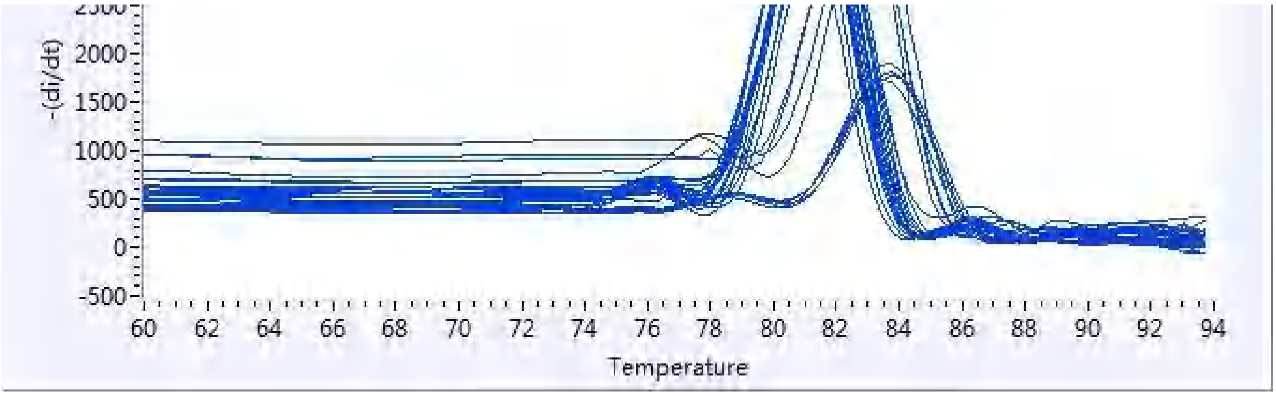
-(dI/dt)
Temperature
2500
2000
1500
1000
500
0
-500
60
62
64
66
68
70
72
74
76
78
80
82
84
86
88
90
92
94

–
–
–
–
–
–
–
–
–
–
–
–
–
–
–
–
–
–
–
–
–
–
–
–
–
–
–

Subset Id	Control Group	Reference Gene

Table of Original Data

Well	Subset Id	Sample Name	Gene	Reporter	Ct	
	D2	S I	CONTROL	ACTIN	SYBR GREEN	13.3094
	D3	S I	CONTROL	ACTIN	SYBR GREEN	13.6752
	D4	S I	CONTROL	ACTIN	SYBR GREEN	13.6995
	B2	S I	CONTROL	OPG	SYBR GREEN	27.0439
	B3	S I	CONTROL	OPG	SYBR GREEN	27.0778
	B4	S I	CONTROL	OPG	SYBR GREEN	26.9265
	C2	S I	CONTROL	OPGL	SYBR GREEN	28.2776
	C3	S I	CONTROL	OPGL	SYBR GREEN	28.3954
	C4	S I	CONTROL	OPGL	SYBR GREEN	28.4947
	D5	S I	GLUCOSE	ACTIN	SYBR GREEN	13.4971
	D6	S I	GLUCOSE	ACTIN	SYBR GREEN	13.1771
	D7	S I	GLUCOSE	ACTIN	SYBR GREEN	13.4159
	B5	S I	GLUCOSE	OPG	SYBR GREEN	28.3728
	B6	S I	GLUCOSE	OPG	SYBR GREEN	28.8521
	B7	S I	GLUCOSE	OPG	SYBR GREEN	28.4328
	C5	S I	GLUCOSE	OPGL	SYBR GREEN	27.1565
	C6	S I	GLUCOSE	OPGL	SYBR GREEN	27.4002
	C7	S I	GLUCOSE	OPGL	SYBR GREEN	27.2304
	D8	S I	GLUCOSE+XLGB	ACTIN	SYBR GREEN	13.1369
	D9	S I	GLUCOSE+XLGB	ACTIN	SYBR GREEN	13.1258
	D10	S I	GLUCOSE+XLGB	ACTIN	SYBR GREEN	13.0887
	B8	S I	GLUCOSE+XLGB	OPG	SYBR GREEN	26.5913
	B9	S I	GLUCOSE+XLGB	OPG	SYBR GREEN	26.3853
	B10	S I	GLUCOSE+XLGB	OPG	SYBR GREEN	26.4361
	C8	S I	GLUCOSE+XLGB	OPGL	SYBR GREEN	27.6486
	C9	S I	GLUCOSE+XLGB	OPGL	SYBR GREEN	27.3989
	C10	S I	GLUCOSE+XLGB	OPGL	SYBR GREEN	27.7184

Table of RQ

Subset Id	Control	Delta Ct	Sample	Target Gene	Delta Ct	DD Ct

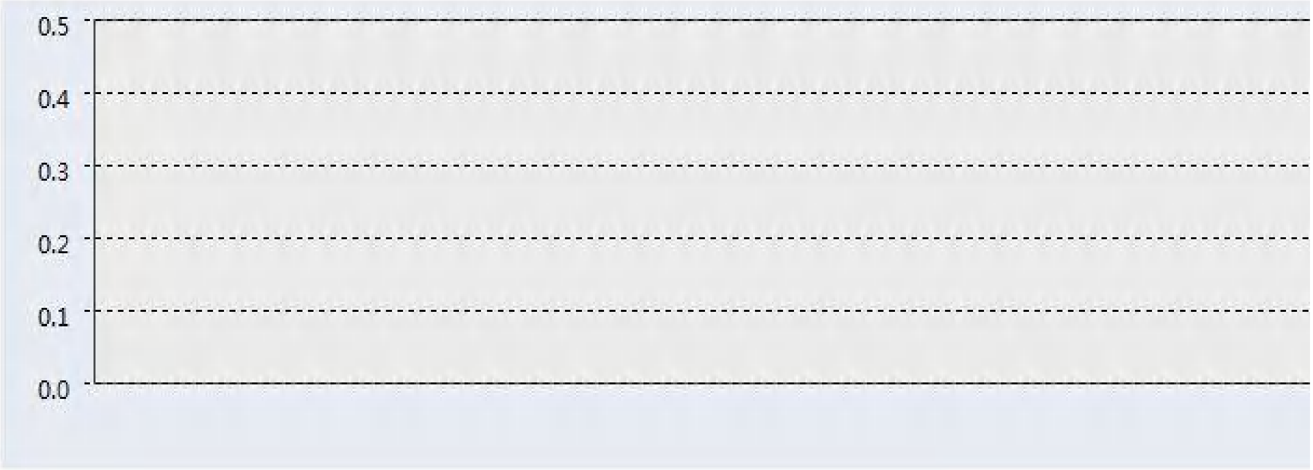

RQ

组织名称	编号	OPG	内参CT值	内参平均值	C-E	F最大平均	F-G	函数POWER	I平均值
CONTROL	1	27.04	13.31	13.56333	13.47667	13.45333	0.023333	0.983957	1.000974
CONTROL	2	27.08	13.68	13.56333	13.51667	13.45333	0.063333	0.95705	
CONTROL	3	26.93	13.7	13.56333	13.36667	13.45333	-0.08667	1.061914	
GLUCOSE	1	28.37	13.5	13.36667	15.00333	13.45333	1.55	0.34151	0.304654
GLUCOSE	2	28.85	13.18	13.36667	15.48333	13.45333	2.03	0.244855	
GLUCOSE	3	28.43	13.42	13.36667	15.06333	13.45333	1.61	0.327598	
GLUCOSE+XI	1	26.59	13.14	13.12	13.47	13.45333	0.016667	0.988514	1.073614
GLUCOSE+XI	2	26.39	13.13	13.12	13.27	13.45333	-0.18333	1.135504	
GLUCOSE+XI	3	26.44	13.09	13.12	13.32	13.45333	-0.13333	1.096825	

组织名称	编号	OPGL	内参CT值	内参平均值	C-E	F最大平均	F-G	函数POWER	I平均值
CONTROL	1	28.28	13.31	13.56333	14.71667	14.82667	-0.11	1.079228	1.001785
CONTROL	2	28.4	13.68	13.56333	14.83667	14.82667	0.01	0.993092	
CONTROL	3	28.49	13.7	13.56333	14.92667	14.82667	0.1	0.933033	
GLUCOSE	1	27.16	13.5	13.36667	13.79333	14.82667	-1.03333	2.046748	1.909877
GLUCOSE	2	27.4	13.18	13.36667	14.03333	14.82667	-0.79333	1.733074	
GLUCOSE	3	27.23	13.42	13.36667	13.86333	14.82667	-0.96333	1.94981	
GLUCOSE+XI	1	27.65	13.14	13.12	14.53	14.82667	-0.29667	1.228303	1.286379
GLUCOSE+XI	2	27.4	13.13	13.12	14.28	14.82667	-0.54667	1.460707	
GLUCOSE+XI	3	27.72	13.09	13.12	14.6	14.82667	-0.22667	1.170128	

OPG	1.000974	0.054463	3	0.304654	0.052253	3	1.073614	0.076194	3
OPGL	1.001785	0.073484	3	1.909877	0.160604	3	1.286379	0.153749	3

函数STDEV
0.054463

0.052253

0.076194

函数STDEV
0.073484

0.160604

0.153749

Experiment Information

Name	P_210910
Date	44449
Operator	admin
Location	F:\Funglyn\FTC-3000\experiments\admin
Template	F:\Funglyn\FTC-3000\templates\SYBR Green 两步法.tmt
Tips	

Protocol

Stage:Hold Cycles:1

Target	Hold(H:M:S)	Ramp Rate(C/s)	Touch Down	Multiple Temp	Singnal Acq.
95	0.416666667				

Stage:Cycles Cycles:40

Target	Hold(H:M:S)	Ramp Rate(C/s)	Touch Down	Multiple Temp	Singnal Acq.
95	0.010416667				
60	0.041666667				TRUE

Stage:Dissociation

Target	Hold(H:M:S)	Ramp Rate(C/s)	Touch Down	Multiple Temp	Singnal Acq.
94	0.0625				
60	0.125	0.1			
94	0.006944444				TRUE

Plate

Well	Reporter	Subset ID	Sample Name	Gene	Sample Type	Quantity
B2	SYBR GREEN	S I	CONTROL	OPG	Unknown Sample	
B3	SYBR GREEN	S I	CONTROL	OPG	Unknown Sample	
B4	SYBR GREEN	S I	CONTROL	OPG	Unknown Sample	
B5	SYBR GREEN	S I	GLUCOSE	OPG	Unknown Sample	
B6	SYBR GREEN	S I	GLUCOSE	OPG	Unknown Sample	
B7	SYBR GREEN	S I	GLUCOSE	OPG	Unknown Sample	
B8	SYBR GREEN	S I	GLUCOSE+XLGB	OPG	Unknown Sample	
B9	SYBR GREEN	S I	GLUCOSE+XLGB	OPG	Unknown Sample	
B10	SYBR GREEN	S I	GLUCOSE+XLGB	OPG	Unknown Sample	
C2	SYBR GREEN	S I	CONTROL	OPGL	Unknown Sample	
C3	SYBR GREEN	S I	CONTROL	OPGL	Unknown Sample	
C4	SYBR GREEN	S I	CONTROL	OPGL	Unknown Sample	
C5	SYBR GREEN	S I	GLUCOSE	OPGL	Unknown Sample	
C6	SYBR GREEN	S I	GLUCOSE	OPGL	Unknown Sample	
C7	SYBR GREEN	S I	GLUCOSE	OPGL	Unknown Sample	
C8	SYBR GREEN	S I	GLUCOSE+XLGB	OPGL	Unknown Sample	
C9	SYBR GREEN	S I	GLUCOSE+XLGB	OPGL	Unknown Sample	
C10	SYBR GREEN	S I	GLUCOSE+XLGB	OPGL	Unknown Sample	
D2	SYBR GREEN	S I	CONTROL	ACTIN	Unknown Sample	
D3	SYBR GREEN	S I	CONTROL	ACTIN	Unknown Sample	
D4	SYBR GREEN	S I	CONTROL	ACTIN	Unknown Sample	
D5	SYBR GREEN	S I	GLUCOSE	ACTIN	Unknown Sample	
D6	SYBR GREEN	S I	GLUCOSE	ACTIN	Unknown Sample	
D7	SYBR GREEN	S I	GLUCOSE	ACTIN	Unknown Sample	
D8	SYBR GREEN	S I	GLUCOSE+XLGB	ACTIN	Unknown Sample	
D9	SYBR GREEN	S I	GLUCOSE+XLGB	ACTIN	Unknown Sample	
D10	SYBR GREEN	S I	GLUCOSE+XLGB	ACTIN	Unknown Sample	

Data

Well	Subset ID	Reporter	S_Name	S_Type	Ct	Mean Ct
B2	S I	SYBR GREEN	CONTROL	Unknown Sample	27.04	27.02
B3	S I	SYBR GREEN	CONTROL	Unknown Sample	27.08	27.02
B4	S I	SYBR GREEN	CONTROL	Unknown Sample	26.93	27.02
B5	S I	SYBR GREEN	GLUCOSE	Unknown Sample	28.37	28.55
B6	S I	SYBR GREEN	GLUCOSE	Unknown Sample	28.85	28.55
B7	S I	SYBR GREEN	GLUCOSE	Unknown Sample	28.43	28.55
B8	S I	SYBR GREEN	GLUCOSE+XLGB	Unknown Sample	26.59	26.47
B9	S I	SYBR GREEN	GLUCOSE+XLGB	Unknown Sample	26.39	26.47
B10	S I	SYBR GREEN	GLUCOSE+XLGB	Unknown Sample	26.44	26.47
C2	S I	SYBR GREEN	CONTROL	Unknown Sample	28.28	28.39
C3	S I	SYBR GREEN	CONTROL	Unknown Sample	28.4	28.39
C4	S I	SYBR GREEN	CONTROL	Unknown Sample	28.49	28.39
C5	S I	SYBR GREEN	GLUCOSE	Unknown Sample	27.16	27.26
C6	S I	SYBR GREEN	GLUCOSE	Unknown Sample	27.4	27.26
C7	S I	SYBR GREEN	GLUCOSE	Unknown Sample	27.23	27.26
C8	S I	SYBR GREEN	GLUCOSE+XLGB	Unknown Sample	27.65	27.59
C9	S I	SYBR GREEN	GLUCOSE+XLGB	Unknown Sample	27.4	27.59
C10	S I	SYBR GREEN	GLUCOSE+XLGB	Unknown Sample	27.72	27.59
D2	S I	SYBR GREEN	CONTROL	Unknown Sample	13.31	13.56
D3	S I	SYBR GREEN	CONTROL	Unknown Sample	13.68	13.56
D4	S I	SYBR GREEN	CONTROL	Unknown Sample	13.7	13.56
D5	S I	SYBR GREEN	GLUCOSE	Unknown Sample	13.5	13.36
D6	S I	SYBR GREEN	GLUCOSE	Unknown Sample	13.18	13.36
D7	S I	SYBR GREEN	GLUCOSE	Unknown Sample	13.42	13.36
D8	S I	SYBR GREEN	GLUCOSE+XLGB	Unknown Sample	13.14	13.12
D9	S I	SYBR GREEN	GLUCOSE+XLGB	Unknown Sample	13.13	13.12
D10	S I	SYBR GREEN	GLUCOSE+XLGB	Unknown Sample	13.09	13.12

Graph

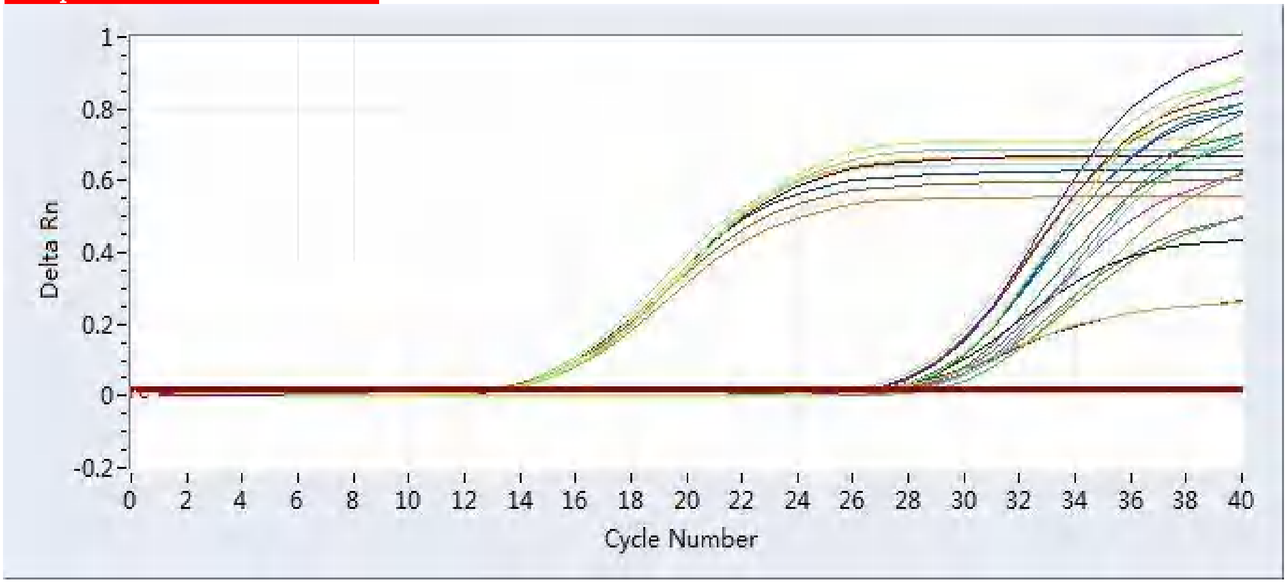

StdDev Ct	Quantity	Gene
0.08	-	OPG
0.08	-	OPG
0.08	-	OPG
0.26	-	OPG
0.26	-	OPG
0.26	-	OPG
0.1	-	OPG
0.1	-	OPG
0.1	-	OPG
0.11	-	OPGL
0.11	-	OPGL
0.11	-	OPGL
0.12	-	OPGL
0.12	-	OPGL
0.12	-	OPGL
0.17	-	OPGL
0.17	-	OPGL
0.17	-	OPGL
0.22	-	ACTIN
0.22	-	ACTIN
0.22	-	ACTIN
0.17	-	ACTIN
0.17	-	ACTIN
0.17	-	ACTIN
0.03	-	ACTIN
0.03	-	ACTIN
0.03	-	ACTIN

Data

Well	Subset Id	Reporter	Sample Name	Gene	Tm1	Tm2
B2	S I	SYBR GREEN	CONTROL	OPG	81.12	-
B3	S I	SYBR GREEN	CONTROL	OPG	81.34	-
B4	S I	SYBR GREEN	CONTROL	OPG	81.08	-
B5	S I	SYBR GREEN	GLUCOSE	OPG	81.4	-
B6	S I	SYBR GREEN	GLUCOSE	OPG	80.76	-
B7	S I	SYBR GREEN	GLUCOSE	OPG	80.72	-
B8	S I	SYBR GREEN	GLUCOSE+XLGB	OPG	81.15	-
B9	S I	SYBR GREEN	GLUCOSE+XLGB	OPG	81.27	-
B10	S I	SYBR GREEN	GLUCOSE+XLGB	OPG	81.48	-
C2	S I	SYBR GREEN	CONTROL	OPGL	81.06	-
C3	S I	SYBR GREEN	CONTROL	OPGL	81.07	-
C4	S I	SYBR GREEN	CONTROL	OPGL	81.08	-
C5	S I	SYBR GREEN	GLUCOSE	OPGL	81.24	-
C6	S I	SYBR GREEN	GLUCOSE	OPGL	81.55	-
C7	S I	SYBR GREEN	GLUCOSE	OPGL	81.58	-
C8	S I	SYBR GREEN	GLUCOSE+XLGB	OPGL	81.59	-
C9	S I	SYBR GREEN	GLUCOSE+XLGB	OPGL	80.82	-
C10	S I	SYBR GREEN	GLUCOSE+XLGB	OPGL	80.79	-
D2	S I	SYBR GREEN	CONTROL	ACTIN	83.38	-
D3	S I	SYBR GREEN	CONTROL	ACTIN	83.74	-
D4	S I	SYBR GREEN	CONTROL	ACTIN	83.43	-
D5	S I	SYBR GREEN	GLUCOSE	ACTIN	83.77	-
D6	S I	SYBR GREEN	GLUCOSE	ACTIN	82.52	-
D7	S I	SYBR GREEN	GLUCOSE	ACTIN	82.53	-
D8	S I	SYBR GREEN	GLUCOSE+XLGB	ACTIN	83.52	-
D9	S I	SYBR GREEN	GLUCOSE+XLGB	ACTIN	82.95	-
D10	S I	SYBR GREEN	GLUCOSE+XLGB	ACTIN	82.84	-

Rn Curve

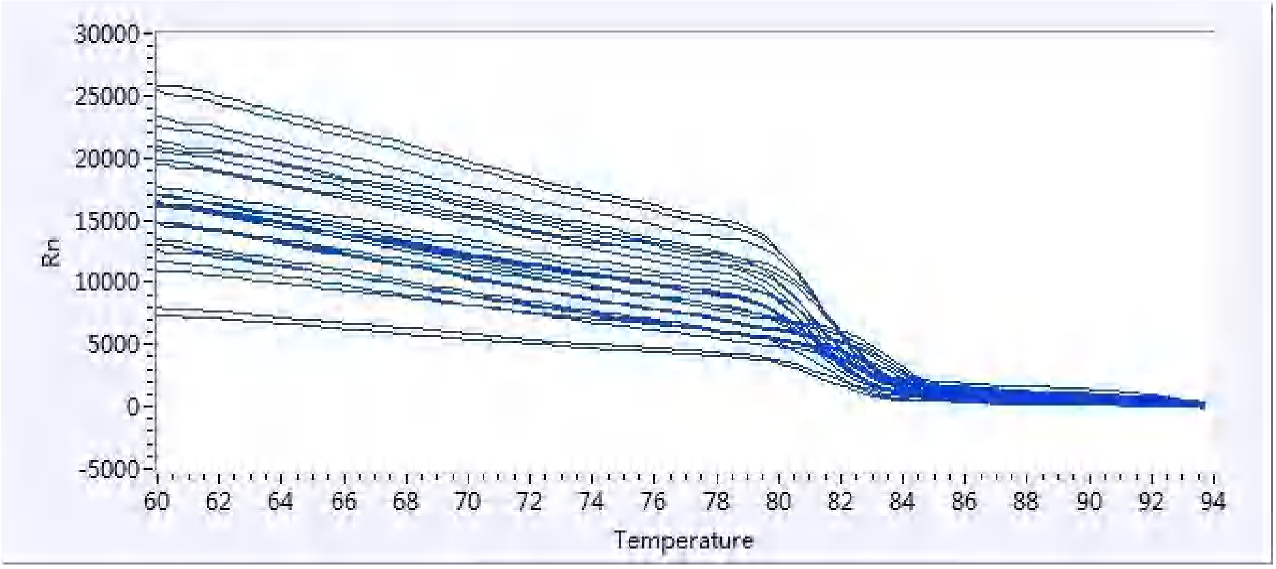

-(di/dt) Curve

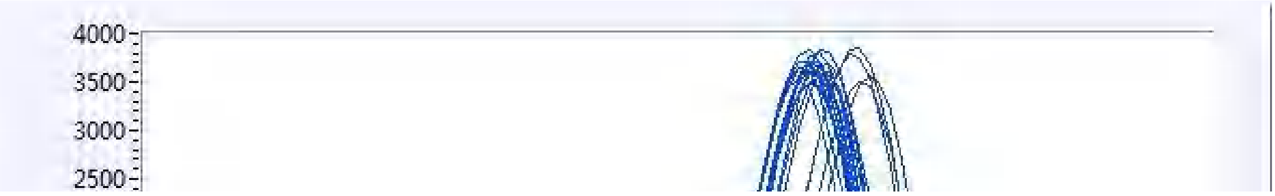

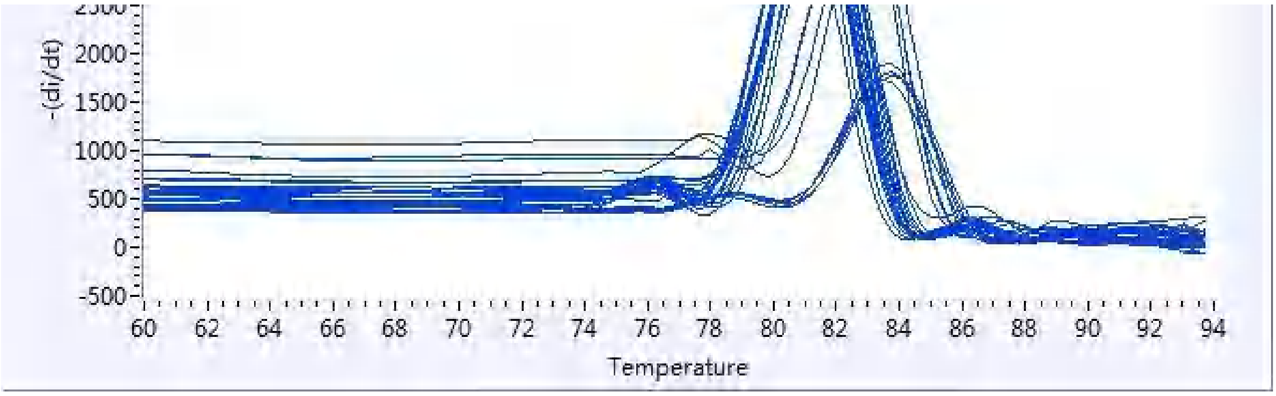

-(dI/dT)
2500
2000
1500
1000
500
0
-500
60
62
64
66
68
70
72
74
76
78
80
82
84
86
88
90
92
94
Temperature

–
–
–
–
–
–
–
–
–
–
–
–
–
–
–
–
–
–
–
–
–
–
–
–
–
–
–

Subset Id	Control Group	Reference Gene

Table of Original Data

Well	Subset Id	Sample Name	Gene	Reporter	Ct	
	D2	S I	CONTROL	ACTIN	SYBR GREEN	13.3094
	D3	S I	CONTROL	ACTIN	SYBR GREEN	13.6752
	D4	S I	CONTROL	ACTIN	SYBR GREEN	13.6995
	B2	S I	CONTROL	OPG	SYBR GREEN	27.0439
	B3	S I	CONTROL	OPG	SYBR GREEN	27.0778
	B4	S I	CONTROL	OPG	SYBR GREEN	26.9265
	C2	S I	CONTROL	OPGL	SYBR GREEN	28.2776
	C3	S I	CONTROL	OPGL	SYBR GREEN	28.3954
	C4	S I	CONTROL	OPGL	SYBR GREEN	28.4947
	D5	S I	GLUCOSE	ACTIN	SYBR GREEN	13.4971
	D6	S I	GLUCOSE	ACTIN	SYBR GREEN	13.1771
	D7	S I	GLUCOSE	ACTIN	SYBR GREEN	13.4159
	B5	S I	GLUCOSE	OPG	SYBR GREEN	28.3728
	B6	S I	GLUCOSE	OPG	SYBR GREEN	28.8521
	B7	S I	GLUCOSE	OPG	SYBR GREEN	28.4328
	C5	S I	GLUCOSE	OPGL	SYBR GREEN	27.1565
	C6	S I	GLUCOSE	OPGL	SYBR GREEN	27.4002
	C7	S I	GLUCOSE	OPGL	SYBR GREEN	27.2304
	D8	S I	GLUCOSE+XLGB	ACTIN	SYBR GREEN	13.1369
	D9	S I	GLUCOSE+XLGB	ACTIN	SYBR GREEN	13.1258
	D10	S I	GLUCOSE+XLGB	ACTIN	SYBR GREEN	13.0887
	B8	S I	GLUCOSE+XLGB	OPG	SYBR GREEN	26.5913
	B9	S I	GLUCOSE+XLGB	OPG	SYBR GREEN	26.3853
	B10	S I	GLUCOSE+XLGB	OPG	SYBR GREEN	26.4361
	C8	S I	GLUCOSE+XLGB	OPGL	SYBR GREEN	27.6486
	C9	S I	GLUCOSE+XLGB	OPGL	SYBR GREEN	27.3989
	C10	S I	GLUCOSE+XLGB	OPGL	SYBR GREEN	27.7184

Table of RQ

Subset Id	Control	Delta Ct	Sample	Target Gene	Delta Ct	DD Ct

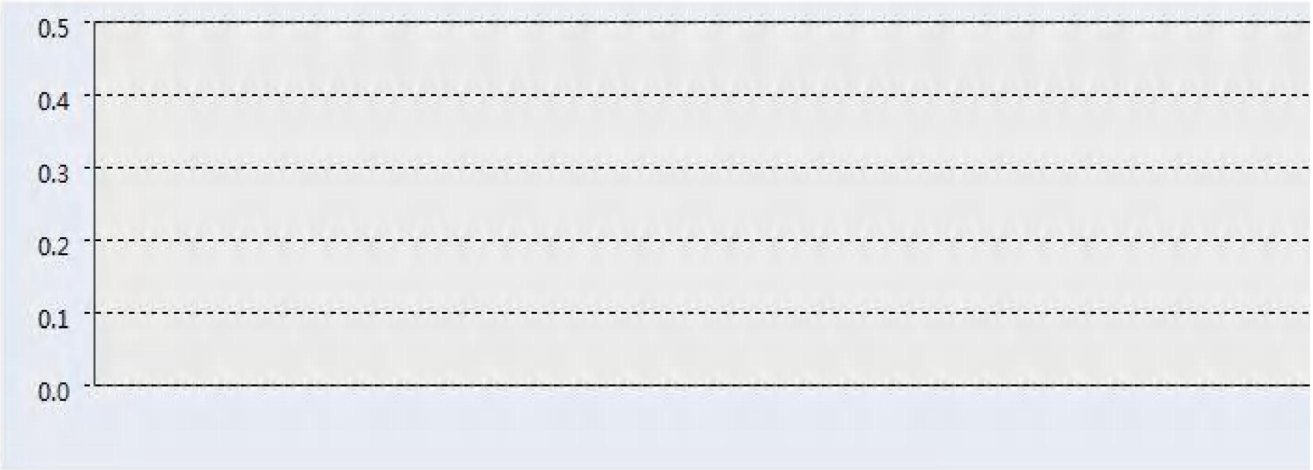

RQ

组织名称	编号	OPG	内参CT值	内参平均值	C-E	F最大平均	F-G	函数POWER	I平均值
CONTROL	1	27.04	13.31	13.56333	13.47667	13.45333	0.023333	0.983957	1.000974
CONTROL	2	27.08	13.68	13.56333	13.51667	13.45333	0.063333	0.95705	
CONTROL	3	26.93	13.7	13.56333	13.36667	13.45333	-0.08667	1.061914	
GLUCOSE	1	28.37	13.5	13.36667	15.00333	13.45333	1.55	0.34151	0.304654
GLUCOSE	2	28.85	13.18	13.36667	15.48333	13.45333	2.03	0.244855	
GLUCOSE	3	28.43	13.42	13.36667	15.06333	13.45333	1.61	0.327598	
GLUCOSE+XI	1	26.59	13.14	13.12	13.47	13.45333	0.016667	0.988514	1.073614
GLUCOSE+XI	2	26.39	13.13	13.12	13.27	13.45333	-0.18333	1.135504	
GLUCOSE+XI	3	26.44	13.09	13.12	13.32	13.45333	-0.13333	1.096825	

组织名称	编号	OPGL	内参CT值	内参平均值	C-E	F最大平均	F-G	函数POWER	I平均值
CONTROL	1	28.28	13.31	13.56333	14.71667	14.82667	-0.11	1.079228	1.001785
CONTROL	2	28.4	13.68	13.56333	14.83667	14.82667	0.01	0.993092	
CONTROL	3	28.49	13.7	13.56333	14.92667	14.82667	0.1	0.933033	
GLUCOSE	1	27.16	13.5	13.36667	13.79333	14.82667	-1.03333	2.046748	1.909877
GLUCOSE	2	27.4	13.18	13.36667	14.03333	14.82667	-0.79333	1.733074	
GLUCOSE	3	27.23	13.42	13.36667	13.86333	14.82667	-0.96333	1.94981	
GLUCOSE+XI	1	27.65	13.14	13.12	14.53	14.82667	-0.29667	1.228303	1.286379
GLUCOSE+XI	2	27.4	13.13	13.12	14.28	14.82667	-0.54667	1.460707	
GLUCOSE+XI	3	27.72	13.09	13.12	14.6	14.82667	-0.22667	1.170128	

OPG	1.000974	0.054463	3	0.304654	0.052253	3	1.073614	0.076194	3
OPGL	1.001785	0.073484	3	1.909877	0.160604	3	1.286379	0.153749	3

函数STDEV
0.054463

0.052253

0.076194

函数STDEV
0.073484

0.160604

0.153749

Experiment Information

Name	P_210910
Date	44449
Operator	admin
Location	F:\Funglyn\FTC-3000\experiments\admin
Template	F:\Funglyn\FTC-3000\templates\SYBR Green 两步法.tmt
Tips	

Protocol

Stage:Hold Cycles:1

Target	Hold(H:M:S)	Ramp Rate(C/s)	Touch Down	Multiple Temp	Singnal Acq.
95	0.416666667				

Stage:Cycles Cycles:40

Target	Hold(H:M:S)	Ramp Rate(C/s)	Touch Down	Multiple Temp	Singnal Acq.
95	0.010416667				
60	0.041666667				TRUE

Stage:Dissociation

Target	Hold(H:M:S)	Ramp Rate(C/s)	Touch Down	Multiple Temp	Singnal Acq.
94	0.0625				
60	0.125	0.1			
94	0.006944444				TRUE

Plate

Well	Reporter	Subset ID	Sample Name	Gene	Sample Type	Quantity
B2	SYBR GREEN	S I	CONTROL	OPG	Unknown Sample	
B3	SYBR GREEN	S I	CONTROL	OPG	Unknown Sample	
B4	SYBR GREEN	S I	CONTROL	OPG	Unknown Sample	
B5	SYBR GREEN	S I	GLUCOSE	OPG	Unknown Sample	
B6	SYBR GREEN	S I	GLUCOSE	OPG	Unknown Sample	
B7	SYBR GREEN	S I	GLUCOSE	OPG	Unknown Sample	
B8	SYBR GREEN	S I	GLUCOSE+XLGB	OPG	Unknown Sample	
B9	SYBR GREEN	S I	GLUCOSE+XLGB	OPG	Unknown Sample	
B10	SYBR GREEN	S I	GLUCOSE+XLGB	OPG	Unknown Sample	
C2	SYBR GREEN	S I	CONTROL	OPGL	Unknown Sample	
C3	SYBR GREEN	S I	CONTROL	OPGL	Unknown Sample	
C4	SYBR GREEN	S I	CONTROL	OPGL	Unknown Sample	
C5	SYBR GREEN	S I	GLUCOSE	OPGL	Unknown Sample	
C6	SYBR GREEN	S I	GLUCOSE	OPGL	Unknown Sample	
C7	SYBR GREEN	S I	GLUCOSE	OPGL	Unknown Sample	
C8	SYBR GREEN	S I	GLUCOSE+XLGB	OPGL	Unknown Sample	
C9	SYBR GREEN	S I	GLUCOSE+XLGB	OPGL	Unknown Sample	
C10	SYBR GREEN	S I	GLUCOSE+XLGB	OPGL	Unknown Sample	
D2	SYBR GREEN	S I	CONTROL	ACTIN	Unknown Sample	
D3	SYBR GREEN	S I	CONTROL	ACTIN	Unknown Sample	
D4	SYBR GREEN	S I	CONTROL	ACTIN	Unknown Sample	
D5	SYBR GREEN	S I	GLUCOSE	ACTIN	Unknown Sample	
D6	SYBR GREEN	S I	GLUCOSE	ACTIN	Unknown Sample	
D7	SYBR GREEN	S I	GLUCOSE	ACTIN	Unknown Sample	
D8	SYBR GREEN	S I	GLUCOSE+XLGB	ACTIN	Unknown Sample	
D9	SYBR GREEN	S I	GLUCOSE+XLGB	ACTIN	Unknown Sample	
D10	SYBR GREEN	S I	GLUCOSE+XLGB	ACTIN	Unknown Sample	

Data

Well	Subset ID	Reporter	S_Name	S_Type	Ct	Mean Ct
B2	S I	SYBR GREEN	CONTROL	Unknown Sample	27.04	27.02
B3	S I	SYBR GREEN	CONTROL	Unknown Sample	27.08	27.02
B4	S I	SYBR GREEN	CONTROL	Unknown Sample	26.93	27.02
B5	S I	SYBR GREEN	GLUCOSE	Unknown Sample	28.37	28.55
B6	S I	SYBR GREEN	GLUCOSE	Unknown Sample	28.85	28.55
B7	S I	SYBR GREEN	GLUCOSE	Unknown Sample	28.43	28.55
B8	S I	SYBR GREEN	GLUCOSE+XLGB	Unknown Sample	26.59	26.47
B9	S I	SYBR GREEN	GLUCOSE+XLGB	Unknown Sample	26.39	26.47
B10	S I	SYBR GREEN	GLUCOSE+XLGB	Unknown Sample	26.44	26.47
C2	S I	SYBR GREEN	CONTROL	Unknown Sample	28.28	28.39
C3	S I	SYBR GREEN	CONTROL	Unknown Sample	28.4	28.39
C4	S I	SYBR GREEN	CONTROL	Unknown Sample	28.49	28.39
C5	S I	SYBR GREEN	GLUCOSE	Unknown Sample	27.16	27.26
C6	S I	SYBR GREEN	GLUCOSE	Unknown Sample	27.4	27.26
C7	S I	SYBR GREEN	GLUCOSE	Unknown Sample	27.23	27.26
C8	S I	SYBR GREEN	GLUCOSE+XLGB	Unknown Sample	27.65	27.59
C9	S I	SYBR GREEN	GLUCOSE+XLGB	Unknown Sample	27.4	27.59
C10	S I	SYBR GREEN	GLUCOSE+XLGB	Unknown Sample	27.72	27.59
D2	S I	SYBR GREEN	CONTROL	Unknown Sample	13.31	13.56
D3	S I	SYBR GREEN	CONTROL	Unknown Sample	13.68	13.56
D4	S I	SYBR GREEN	CONTROL	Unknown Sample	13.7	13.56
D5	S I	SYBR GREEN	GLUCOSE	Unknown Sample	13.5	13.36
D6	S I	SYBR GREEN	GLUCOSE	Unknown Sample	13.18	13.36
D7	S I	SYBR GREEN	GLUCOSE	Unknown Sample	13.42	13.36
D8	S I	SYBR GREEN	GLUCOSE+XLGB	Unknown Sample	13.14	13.12
D9	S I	SYBR GREEN	GLUCOSE+XLGB	Unknown Sample	13.13	13.12
D10	S I	SYBR GREEN	GLUCOSE+XLGB	Unknown Sample	13.09	13.12

Graph

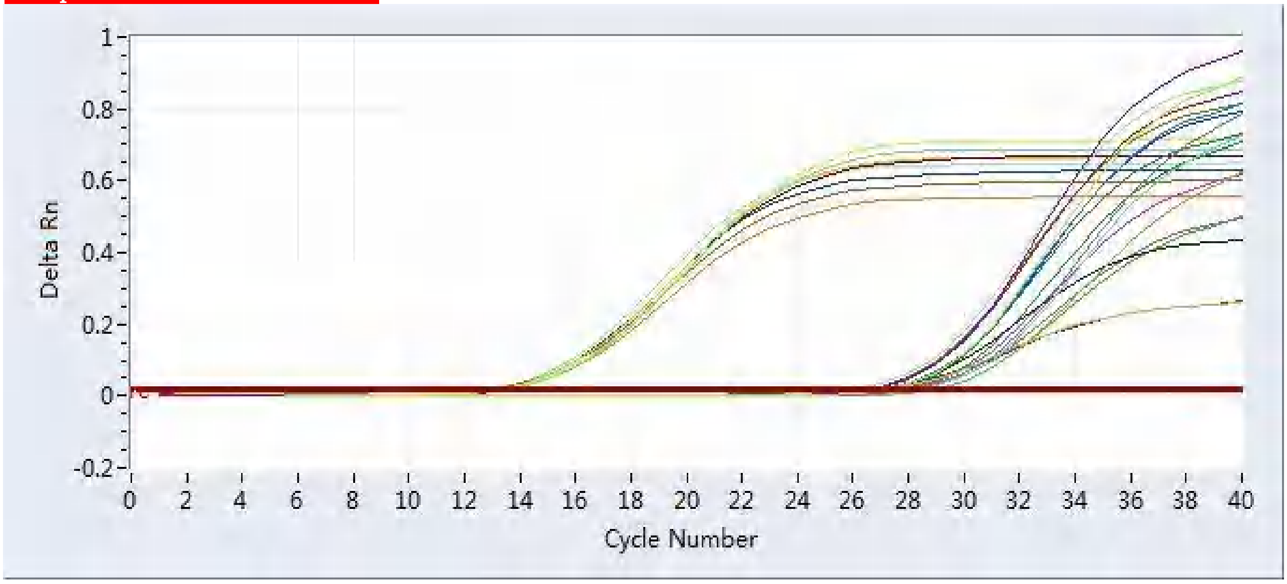

StdDev Ct	Quantity	Gene
0.08	-	OPG
0.08	-	OPG
0.08	-	OPG
0.26	-	OPG
0.26	-	OPG
0.26	-	OPG
0.1	-	OPG
0.1	-	OPG
0.1	-	OPG
0.11	-	OPGL
0.11	-	OPGL
0.11	-	OPGL
0.12	-	OPGL
0.12	-	OPGL
0.12	-	OPGL
0.17	-	OPGL
0.17	-	OPGL
0.17	-	OPGL
0.22	-	ACTIN
0.22	-	ACTIN
0.22	-	ACTIN
0.17	-	ACTIN
0.17	-	ACTIN
0.17	-	ACTIN
0.03	-	ACTIN
0.03	-	ACTIN
0.03	-	ACTIN

Data

Well	Subset Id	Reporter	Sample Name	Gene	Tm1	Tm2
B2	S I	SYBR GREEN	CONTROL	OPG	81.12	-
B3	S I	SYBR GREEN	CONTROL	OPG	81.34	-
B4	S I	SYBR GREEN	CONTROL	OPG	81.08	-
B5	S I	SYBR GREEN	GLUCOSE	OPG	81.4	-
B6	S I	SYBR GREEN	GLUCOSE	OPG	80.76	-
B7	S I	SYBR GREEN	GLUCOSE	OPG	80.72	-
B8	S I	SYBR GREEN	GLUCOSE+XLGB	OPG	81.15	-
B9	S I	SYBR GREEN	GLUCOSE+XLGB	OPG	81.27	-
B10	S I	SYBR GREEN	GLUCOSE+XLGB	OPG	81.48	-
C2	S I	SYBR GREEN	CONTROL	OPGL	81.06	-
C3	S I	SYBR GREEN	CONTROL	OPGL	81.07	-
C4	S I	SYBR GREEN	CONTROL	OPGL	81.08	-
C5	S I	SYBR GREEN	GLUCOSE	OPGL	81.24	-
C6	S I	SYBR GREEN	GLUCOSE	OPGL	81.55	-
C7	S I	SYBR GREEN	GLUCOSE	OPGL	81.58	-
C8	S I	SYBR GREEN	GLUCOSE+XLGB	OPGL	81.59	-
C9	S I	SYBR GREEN	GLUCOSE+XLGB	OPGL	80.82	-
C10	S I	SYBR GREEN	GLUCOSE+XLGB	OPGL	80.79	-
D2	S I	SYBR GREEN	CONTROL	ACTIN	83.38	-
D3	S I	SYBR GREEN	CONTROL	ACTIN	83.74	-
D4	S I	SYBR GREEN	CONTROL	ACTIN	83.43	-
D5	S I	SYBR GREEN	GLUCOSE	ACTIN	83.77	-
D6	S I	SYBR GREEN	GLUCOSE	ACTIN	82.52	-
D7	S I	SYBR GREEN	GLUCOSE	ACTIN	82.53	-
D8	S I	SYBR GREEN	GLUCOSE+XLGB	ACTIN	83.52	-
D9	S I	SYBR GREEN	GLUCOSE+XLGB	ACTIN	82.95	-
D10	S I	SYBR GREEN	GLUCOSE+XLGB	ACTIN	82.84	-

Rn Curve

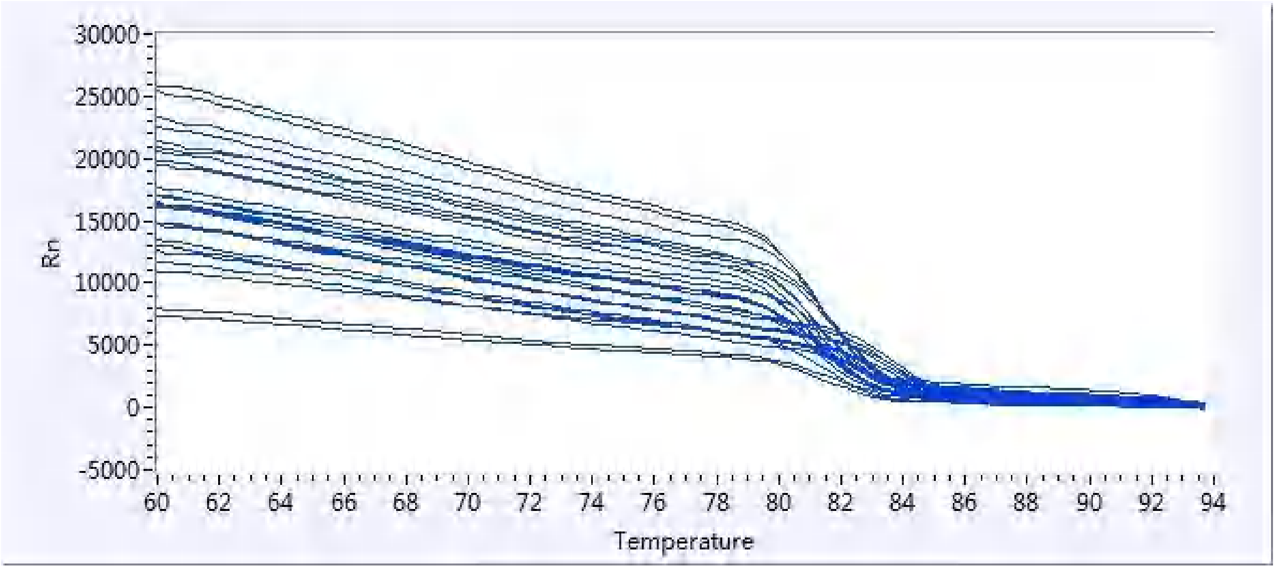

-(di/dt) Curve

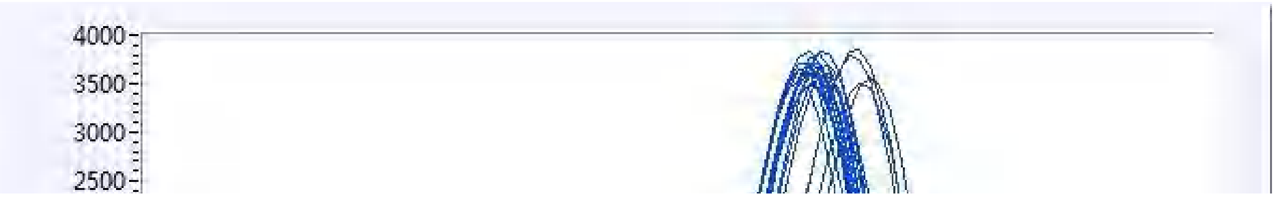

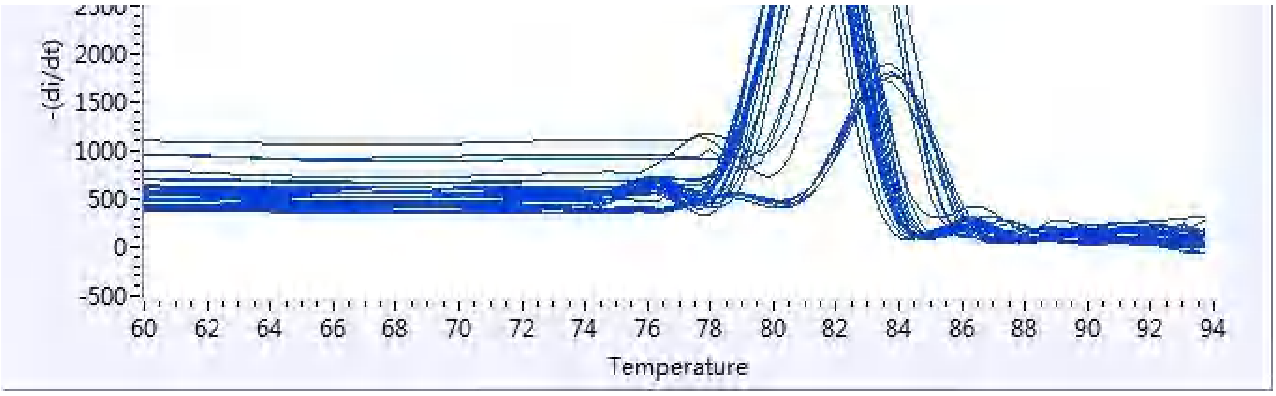

-(dI/dt)
2500
2000
1500
1000
500
0
-500
60
62
64
66
68
70
72
74
76
78
80
82
84
86
88
90
92
94
Temperature

–
–
–
–
–
–
–
–
–
–
–
–
–
–
–
–
–
–
–
–
–
–
–
–
–
–
–

Subset Id	Control Group	Reference Gene

Table of Original Data

Well	Subset Id	Sample Name	Gene	Reporter	Ct	
	D2	S I	CONTROL	ACTIN	SYBR GREEN	13.3094
	D3	S I	CONTROL	ACTIN	SYBR GREEN	13.6752
	D4	S I	CONTROL	ACTIN	SYBR GREEN	13.6995
	B2	S I	CONTROL	OPG	SYBR GREEN	27.0439
	B3	S I	CONTROL	OPG	SYBR GREEN	27.0778
	B4	S I	CONTROL	OPG	SYBR GREEN	26.9265
	C2	S I	CONTROL	OPGL	SYBR GREEN	28.2776
	C3	S I	CONTROL	OPGL	SYBR GREEN	28.3954
	C4	S I	CONTROL	OPGL	SYBR GREEN	28.4947
	D5	S I	GLUCOSE	ACTIN	SYBR GREEN	13.4971
	D6	S I	GLUCOSE	ACTIN	SYBR GREEN	13.1771
	D7	S I	GLUCOSE	ACTIN	SYBR GREEN	13.4159
	B5	S I	GLUCOSE	OPG	SYBR GREEN	28.3728
	B6	S I	GLUCOSE	OPG	SYBR GREEN	28.8521
	B7	S I	GLUCOSE	OPG	SYBR GREEN	28.4328
	C5	S I	GLUCOSE	OPGL	SYBR GREEN	27.1565
	C6	S I	GLUCOSE	OPGL	SYBR GREEN	27.4002
	C7	S I	GLUCOSE	OPGL	SYBR GREEN	27.2304
	D8	S I	GLUCOSE+XLGB	ACTIN	SYBR GREEN	13.1369
	D9	S I	GLUCOSE+XLGB	ACTIN	SYBR GREEN	13.1258
	D10	S I	GLUCOSE+XLGB	ACTIN	SYBR GREEN	13.0887
	B8	S I	GLUCOSE+XLGB	OPG	SYBR GREEN	26.5913
	B9	S I	GLUCOSE+XLGB	OPG	SYBR GREEN	26.3853
	B10	S I	GLUCOSE+XLGB	OPG	SYBR GREEN	26.4361
	C8	S I	GLUCOSE+XLGB	OPGL	SYBR GREEN	27.6486
	C9	S I	GLUCOSE+XLGB	OPGL	SYBR GREEN	27.3989
	C10	S I	GLUCOSE+XLGB	OPGL	SYBR GREEN	27.7184

Table of RQ

Subset Id	Control	Delta Ct	Sample	Target Gene	Delta Ct	DD Ct

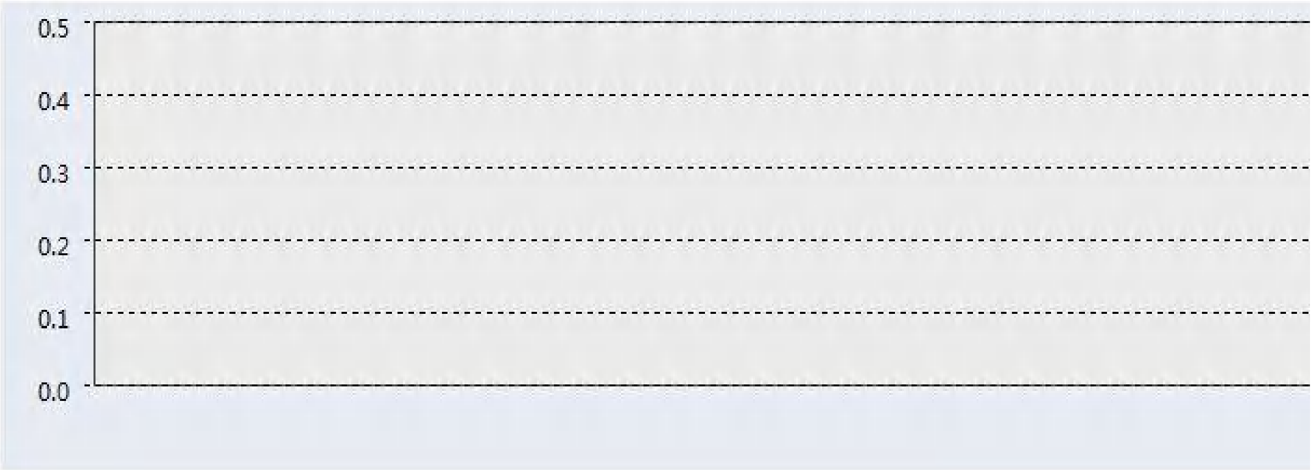

RQ

组织名称	编号	OPG	内参CT值	内参平均值	C-E	F最大平均	F-G	函数POWER	I平均值
CONTROL	1	27.04	13.31	13.56333	13.47667	13.45333	0.023333	0.983957	1.000974
CONTROL	2	27.08	13.68	13.56333	13.51667	13.45333	0.063333	0.95705	
CONTROL	3	26.93	13.7	13.56333	13.36667	13.45333	-0.08667	1.061914	
GLUCOSE	1	28.37	13.5	13.36667	15.00333	13.45333	1.55	0.34151	0.304654
GLUCOSE	2	28.85	13.18	13.36667	15.48333	13.45333	2.03	0.244855	
GLUCOSE	3	28.43	13.42	13.36667	15.06333	13.45333	1.61	0.327598	
GLUCOSE+XI	1	26.59	13.14	13.12	13.47	13.45333	0.016667	0.988514	1.073614
GLUCOSE+XI	2	26.39	13.13	13.12	13.27	13.45333	-0.18333	1.135504	
GLUCOSE+XI	3	26.44	13.09	13.12	13.32	13.45333	-0.13333	1.096825	

组织名称	编号	OPGL	内参CT值	内参平均值	C-E	F最大平均	F-G	函数POWER	I平均值
CONTROL	1	28.28	13.31	13.56333	14.71667	14.82667	-0.11	1.079228	1.001785
CONTROL	2	28.4	13.68	13.56333	14.83667	14.82667	0.01	0.993092	
CONTROL	3	28.49	13.7	13.56333	14.92667	14.82667	0.1	0.933033	
GLUCOSE	1	27.16	13.5	13.36667	13.79333	14.82667	-1.03333	2.046748	1.909877
GLUCOSE	2	27.4	13.18	13.36667	14.03333	14.82667	-0.79333	1.733074	
GLUCOSE	3	27.23	13.42	13.36667	13.86333	14.82667	-0.96333	1.94981	
GLUCOSE+XI	1	27.65	13.14	13.12	14.53	14.82667	-0.29667	1.228303	1.286379
GLUCOSE+XI	2	27.4	13.13	13.12	14.28	14.82667	-0.54667	1.460707	
GLUCOSE+XI	3	27.72	13.09	13.12	14.6	14.82667	-0.22667	1.170128	

OPG	1.000974	0.054463	3	0.304654	0.052253	3	1.073614	0.076194	3
OPGL	1.001785	0.073484	3	1.909877	0.160604	3	1.286379	0.153749	3

函数STDEV
0.054463

0.052253

0.076194

函数STDEV
0.073484

0.160604

0.153749

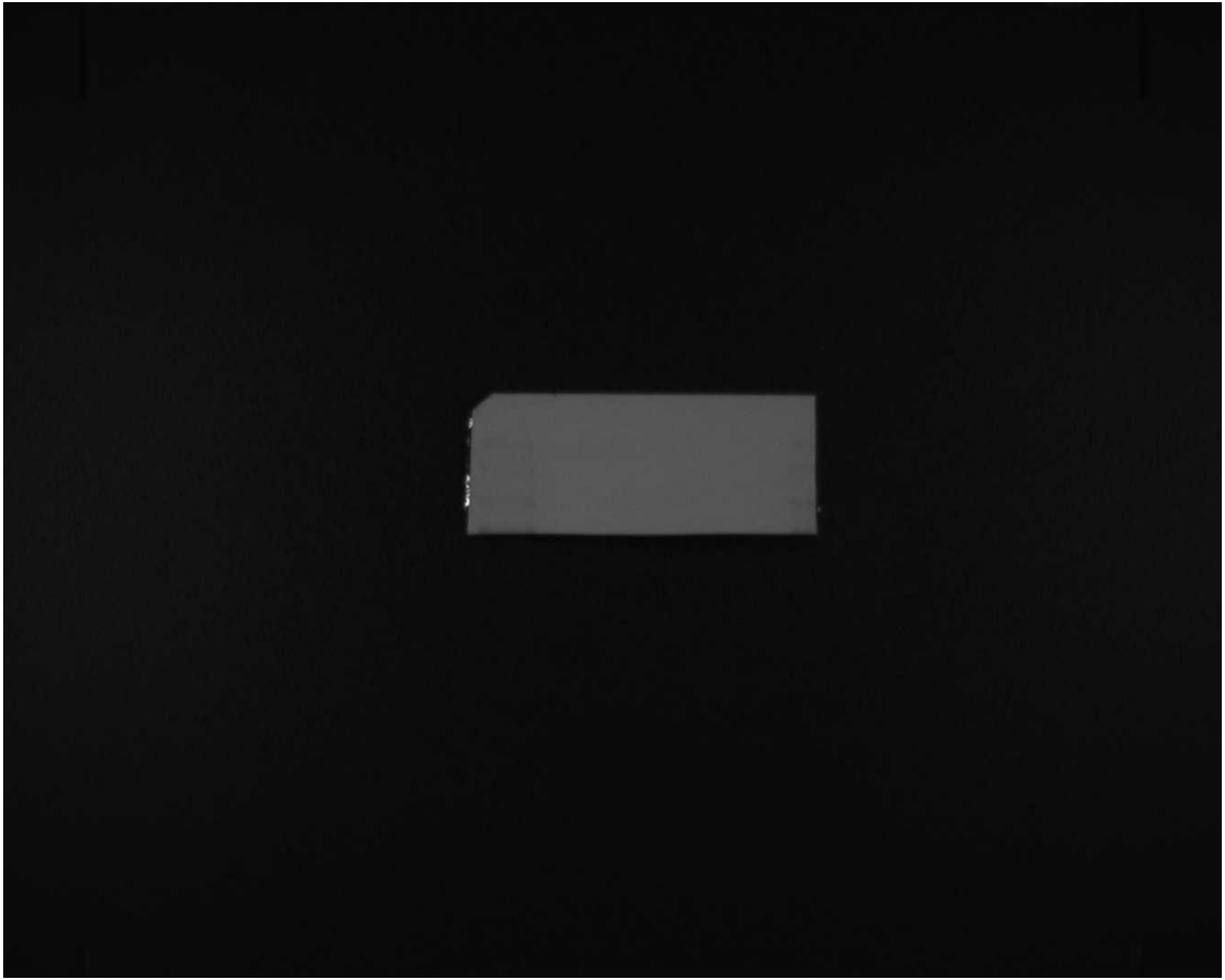

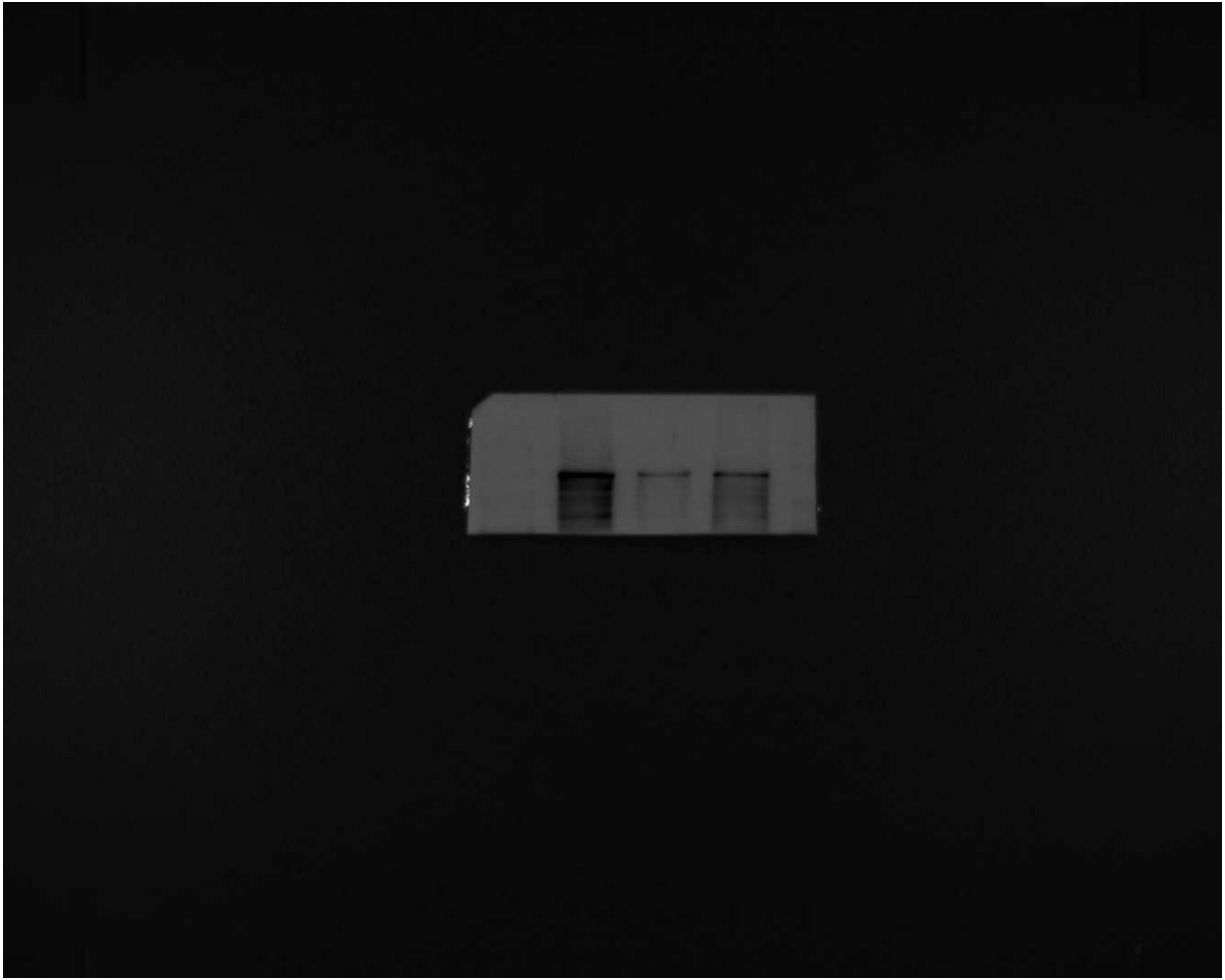

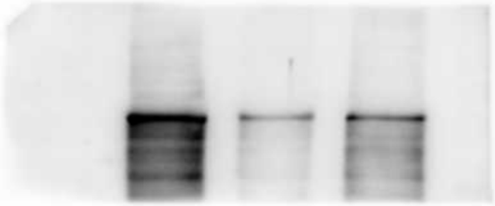

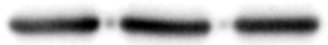

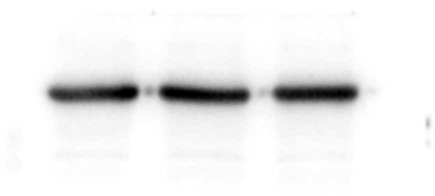

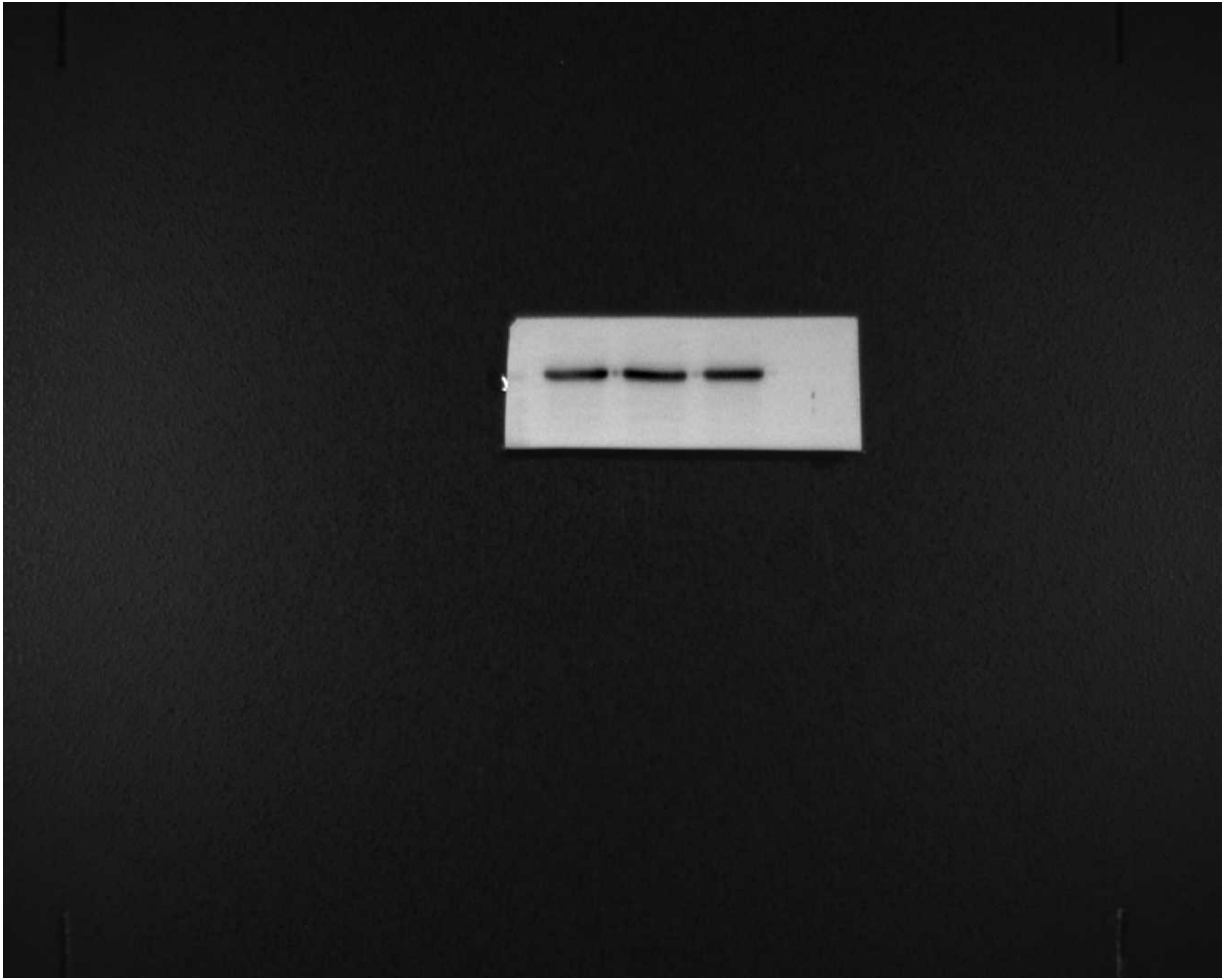

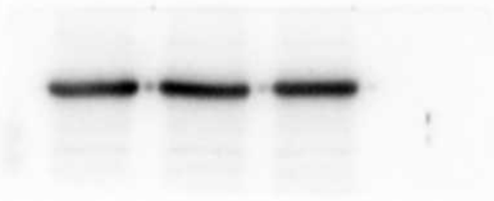

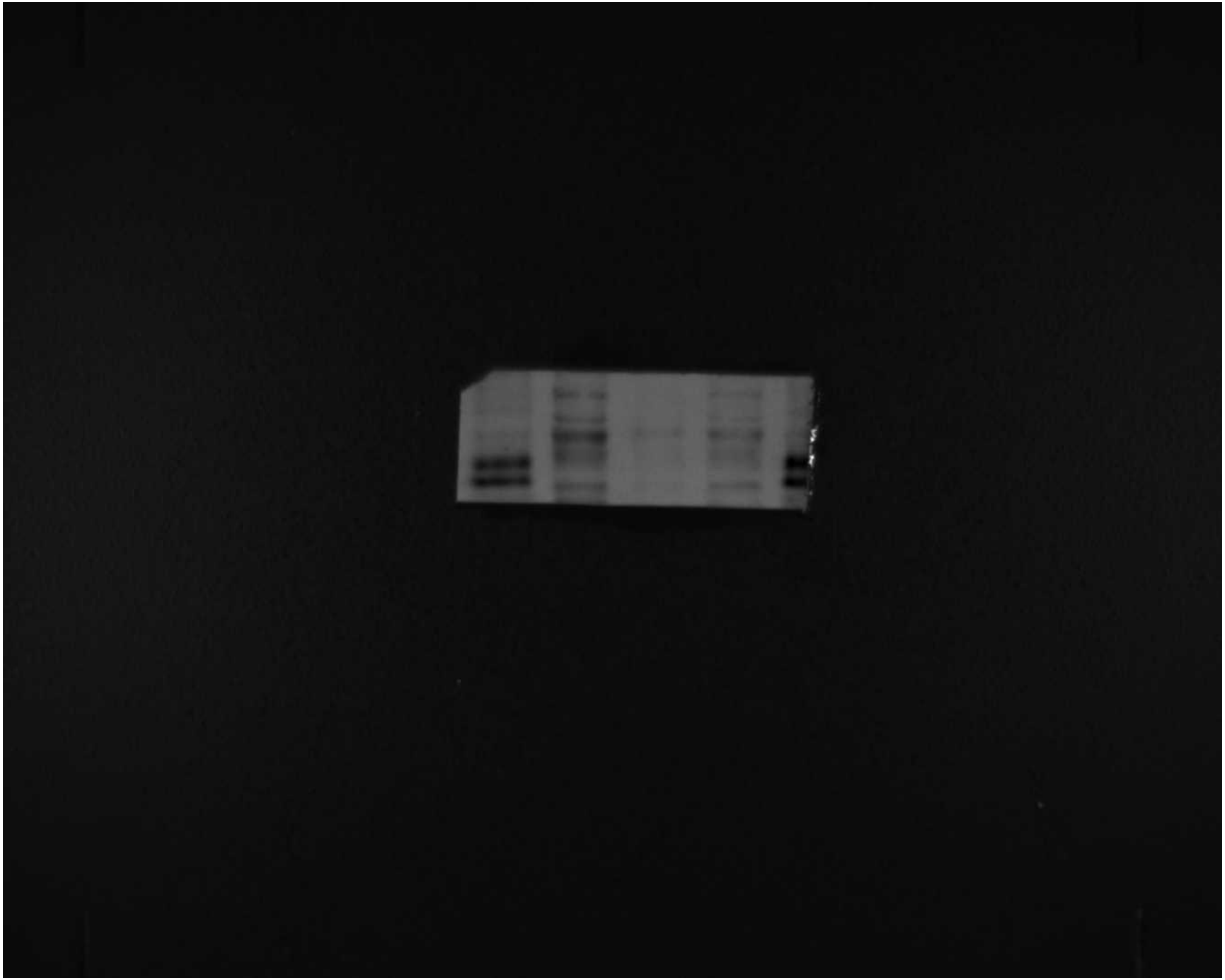

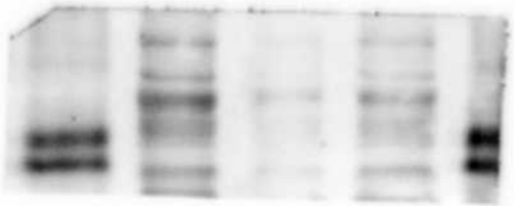

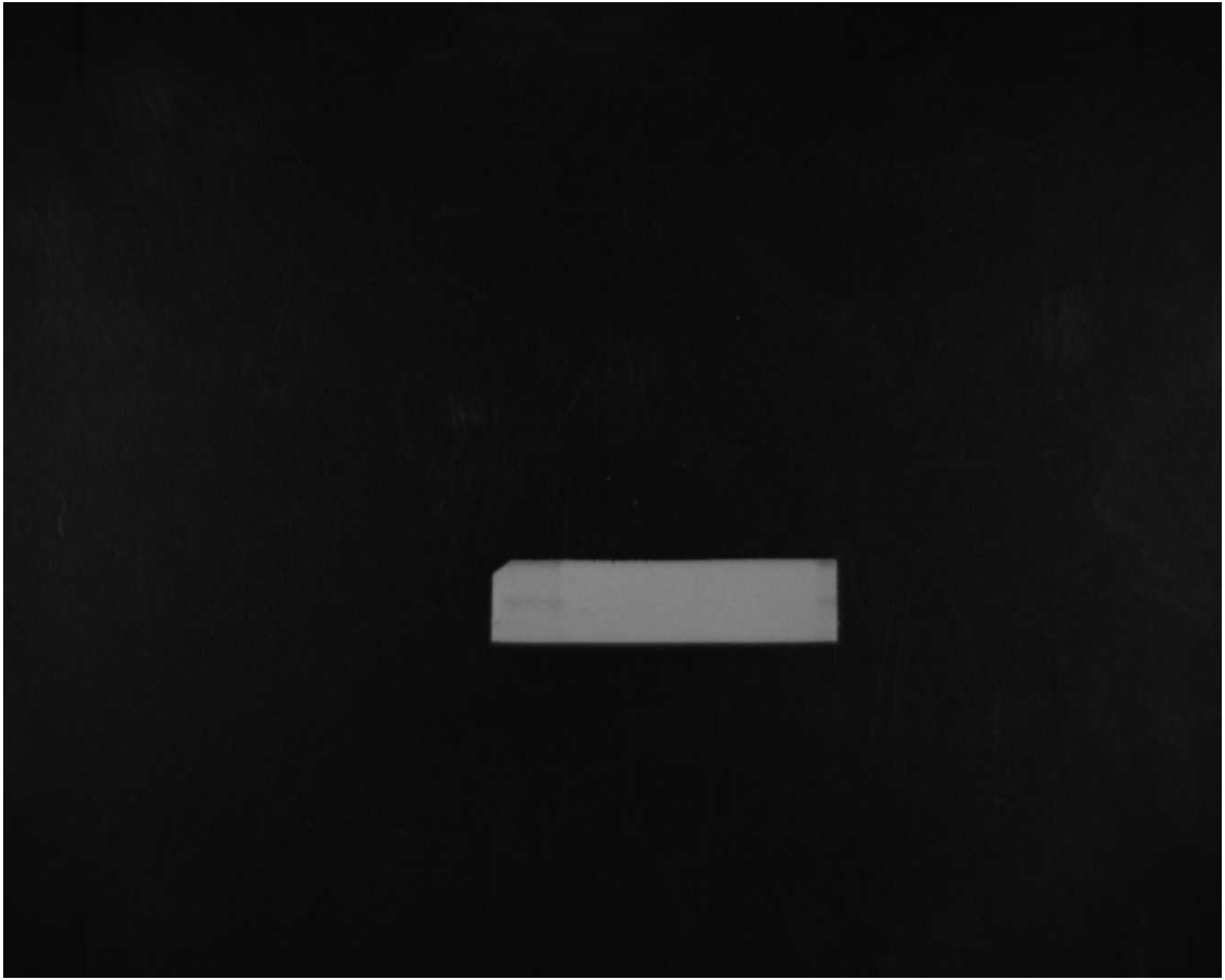

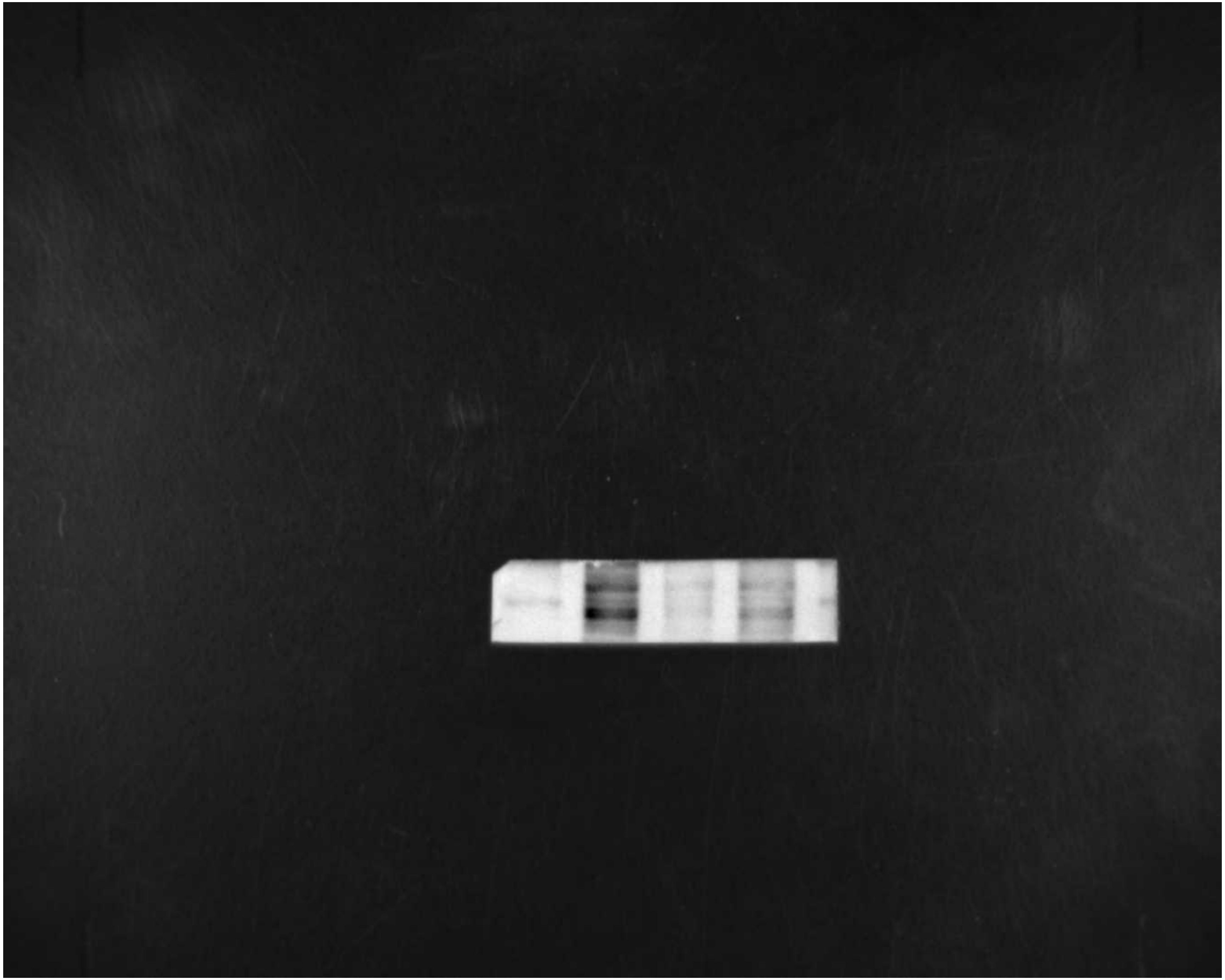

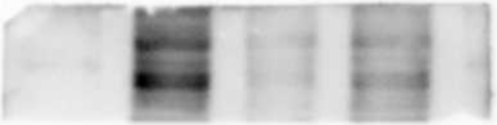

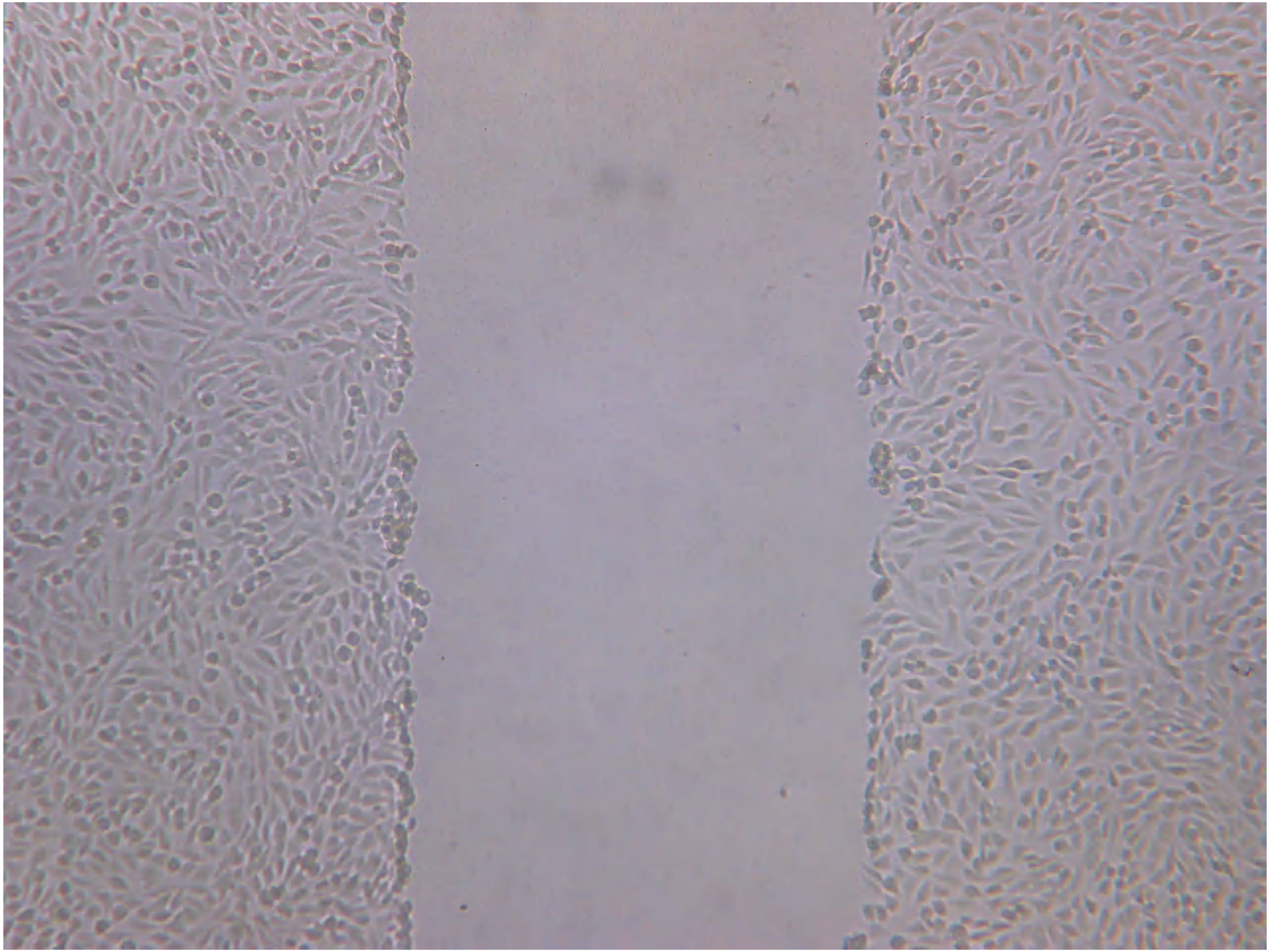

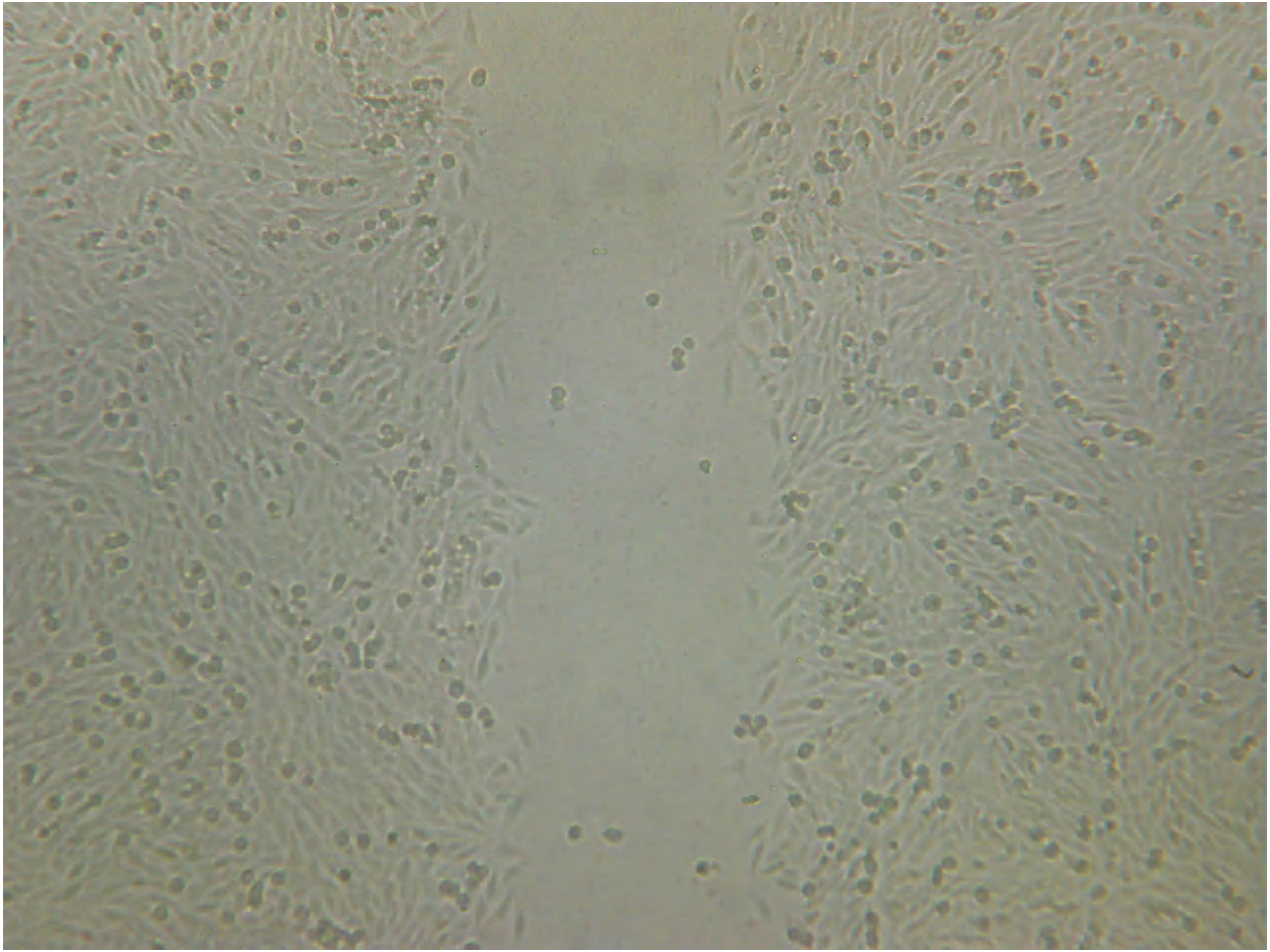

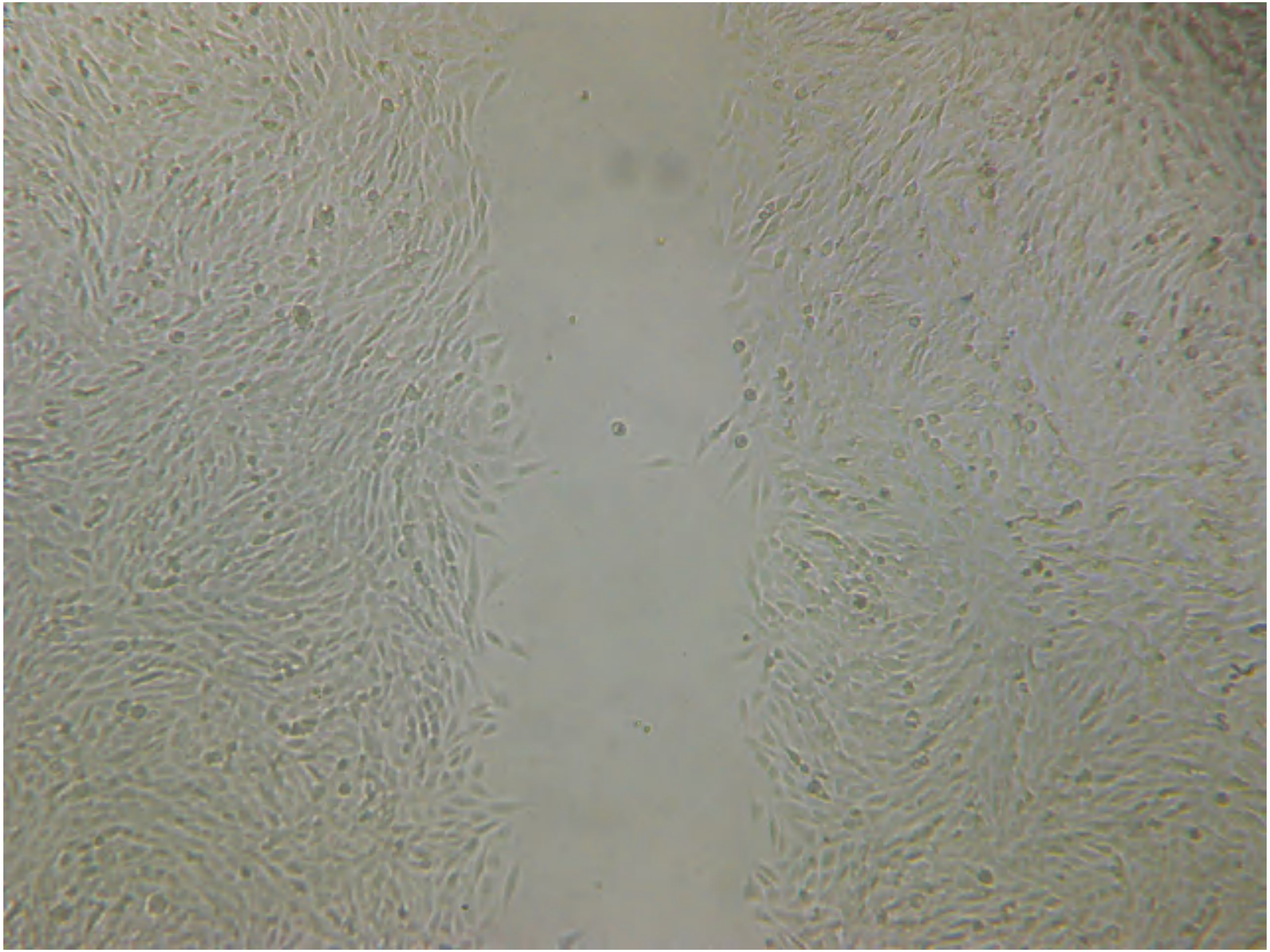

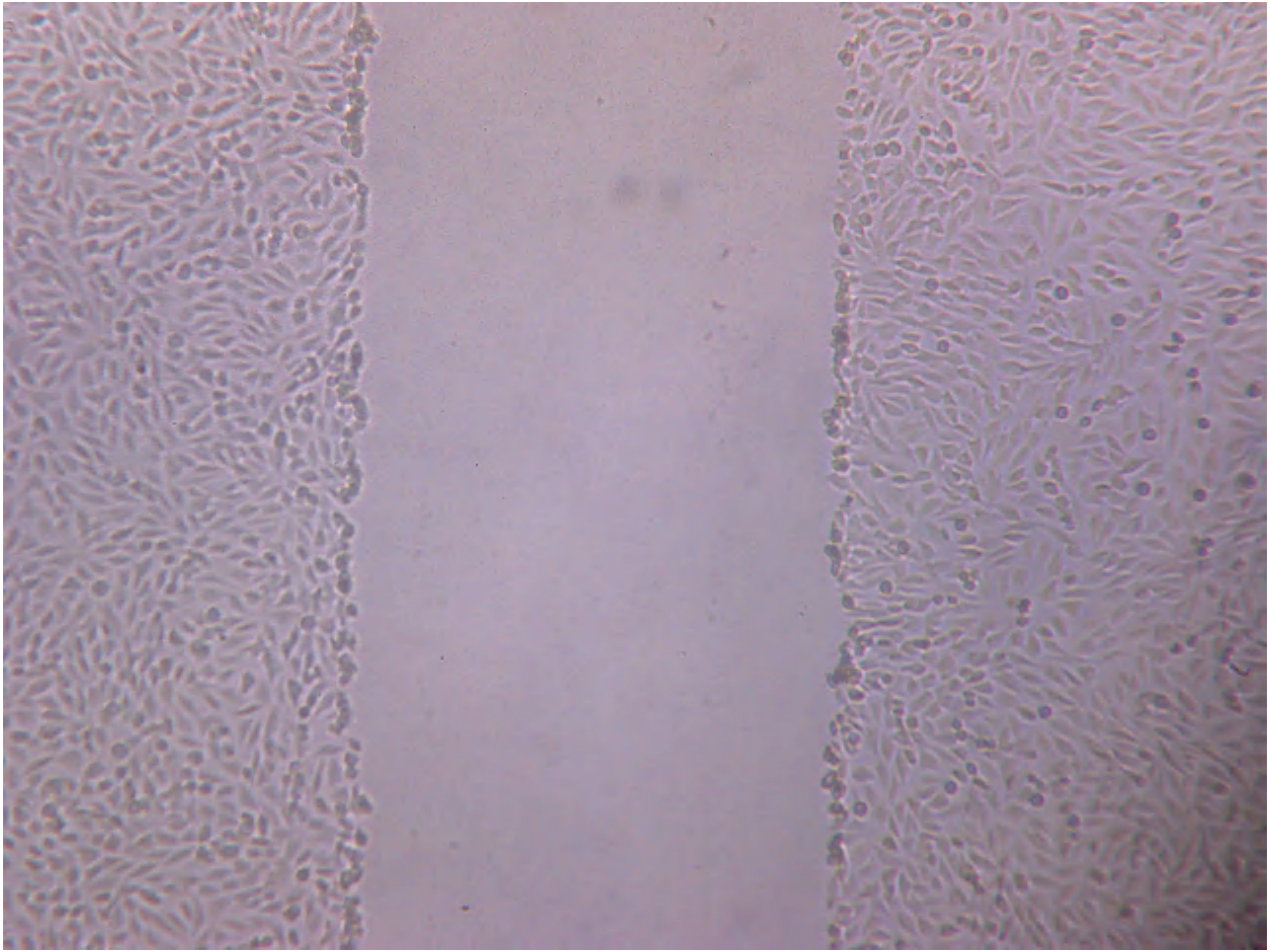

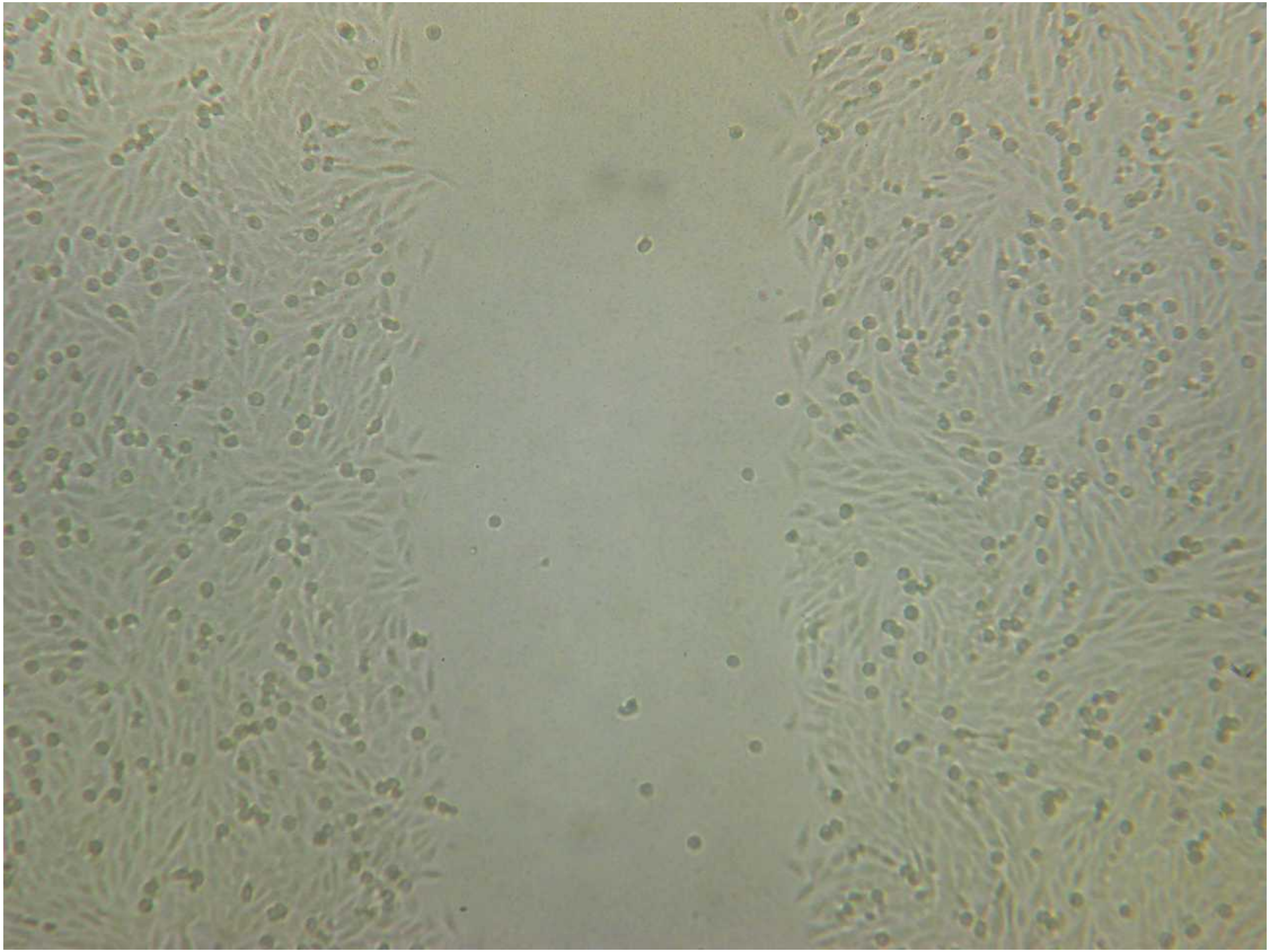

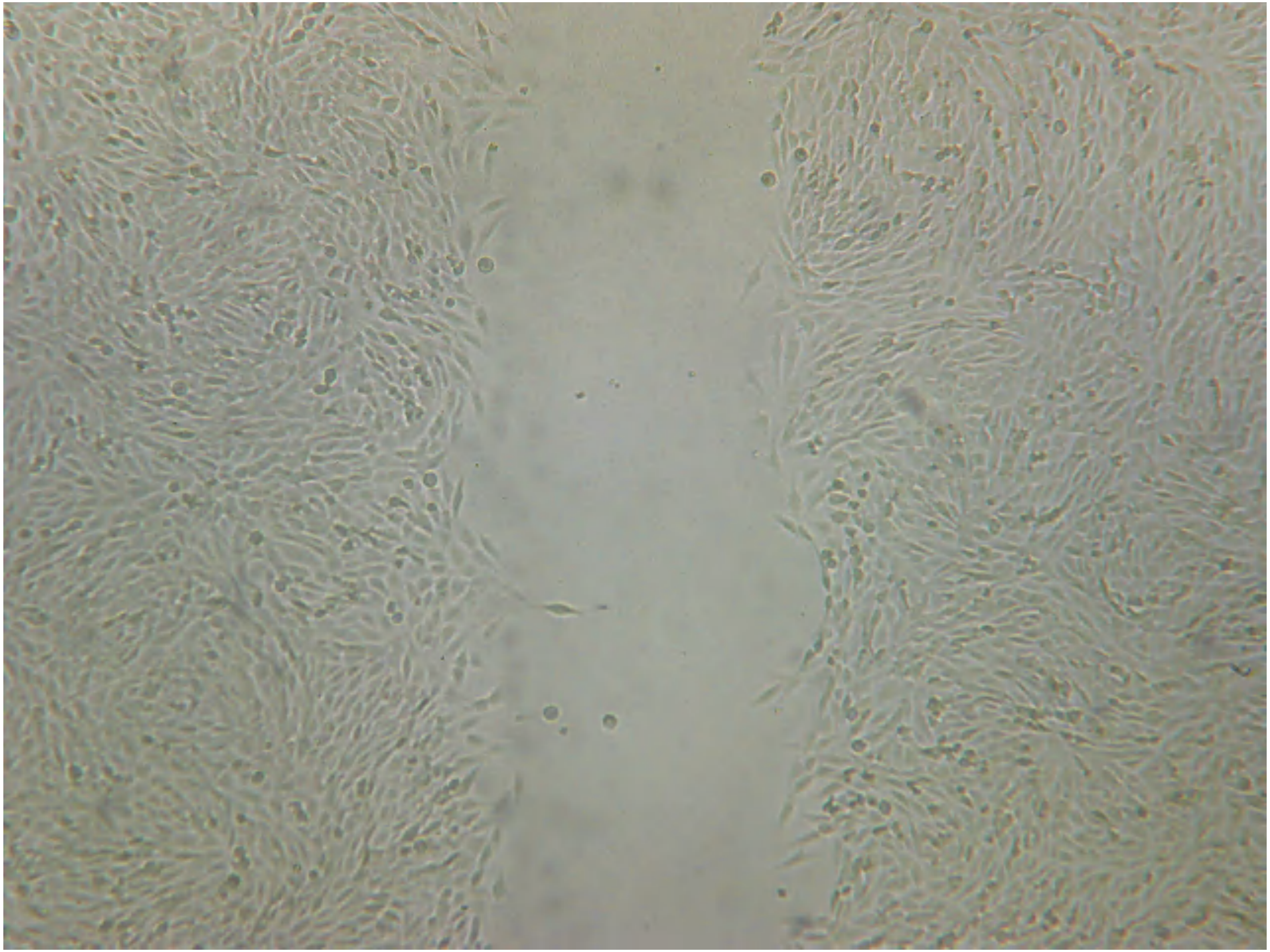

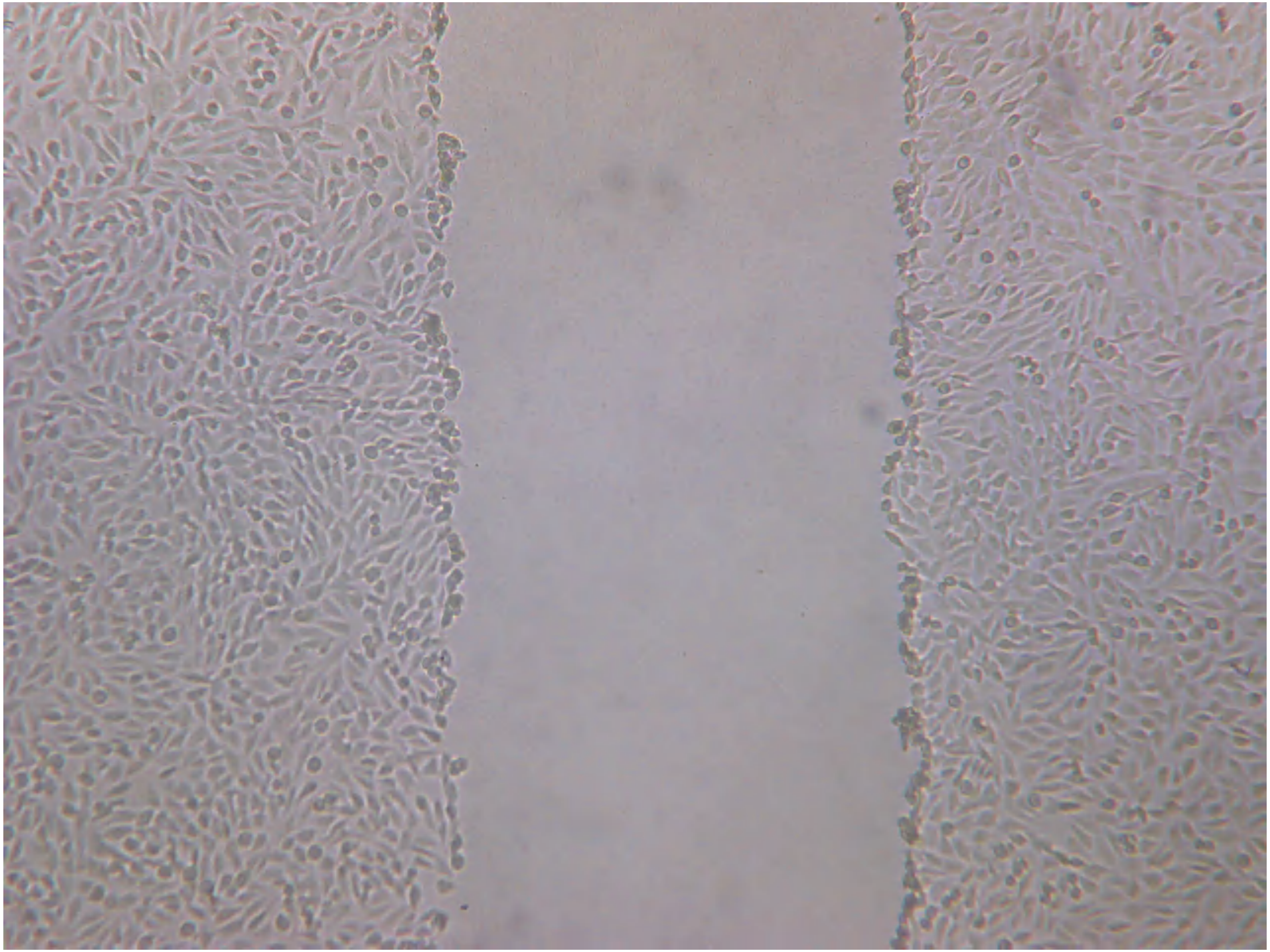

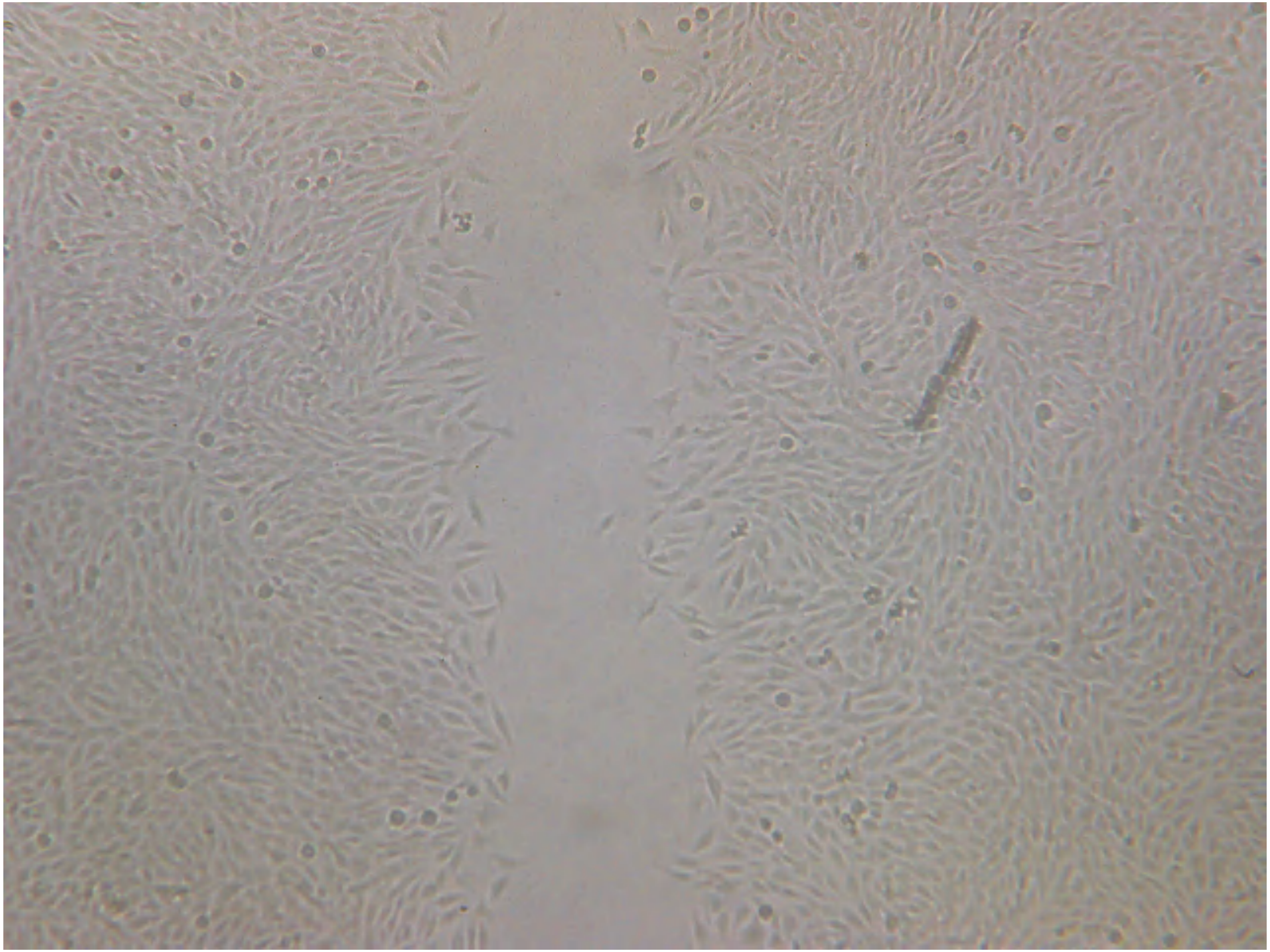

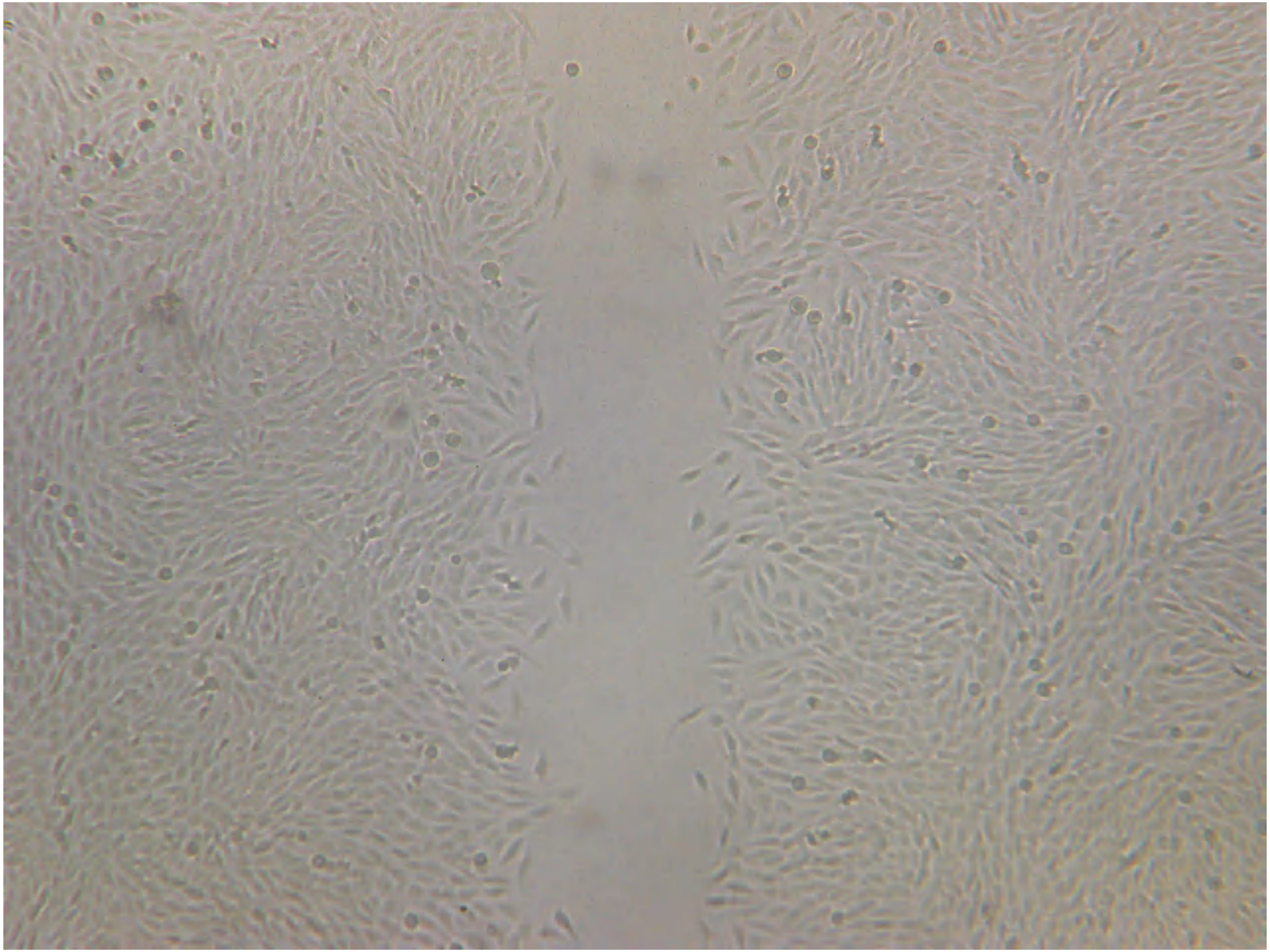

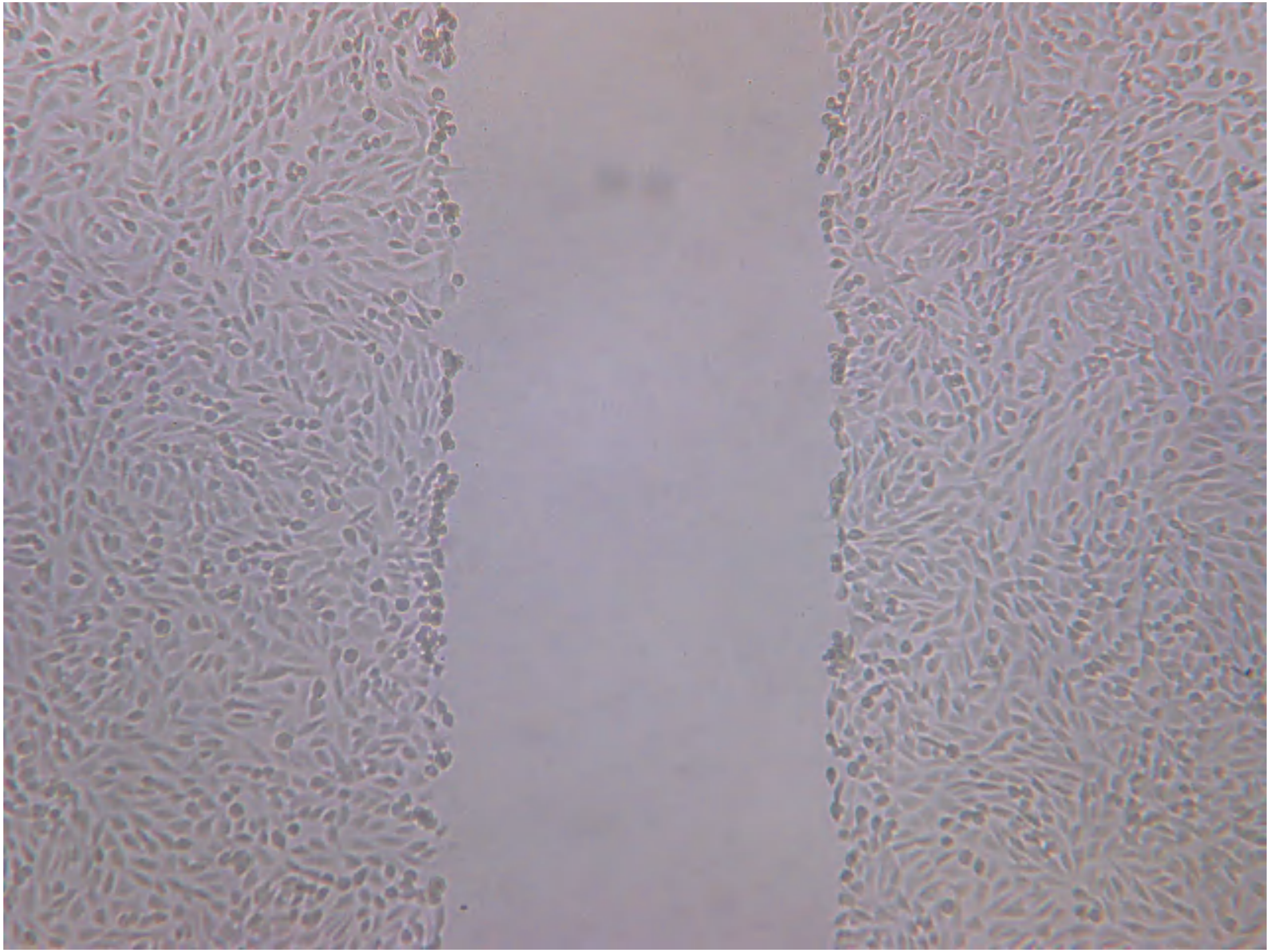

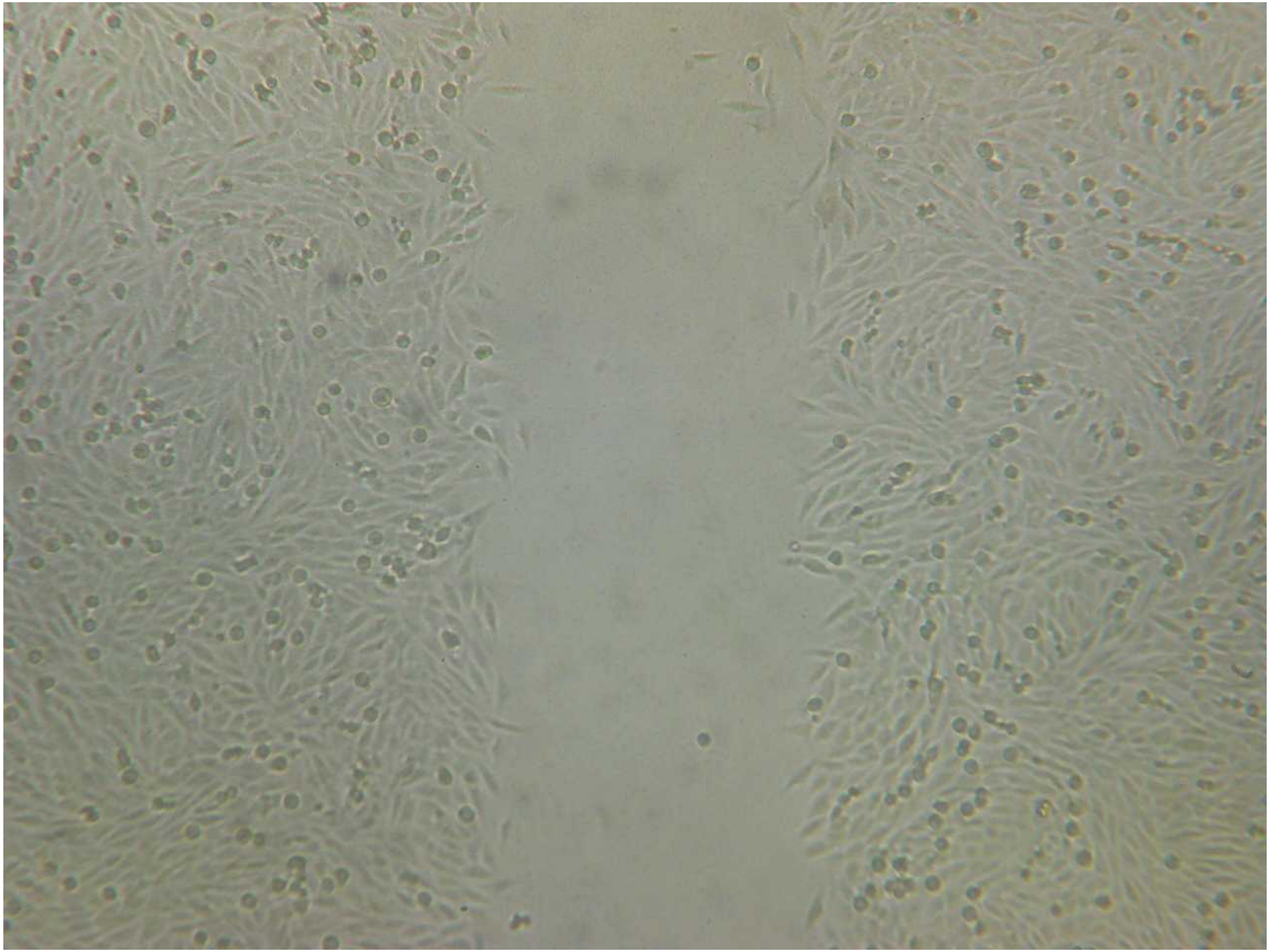

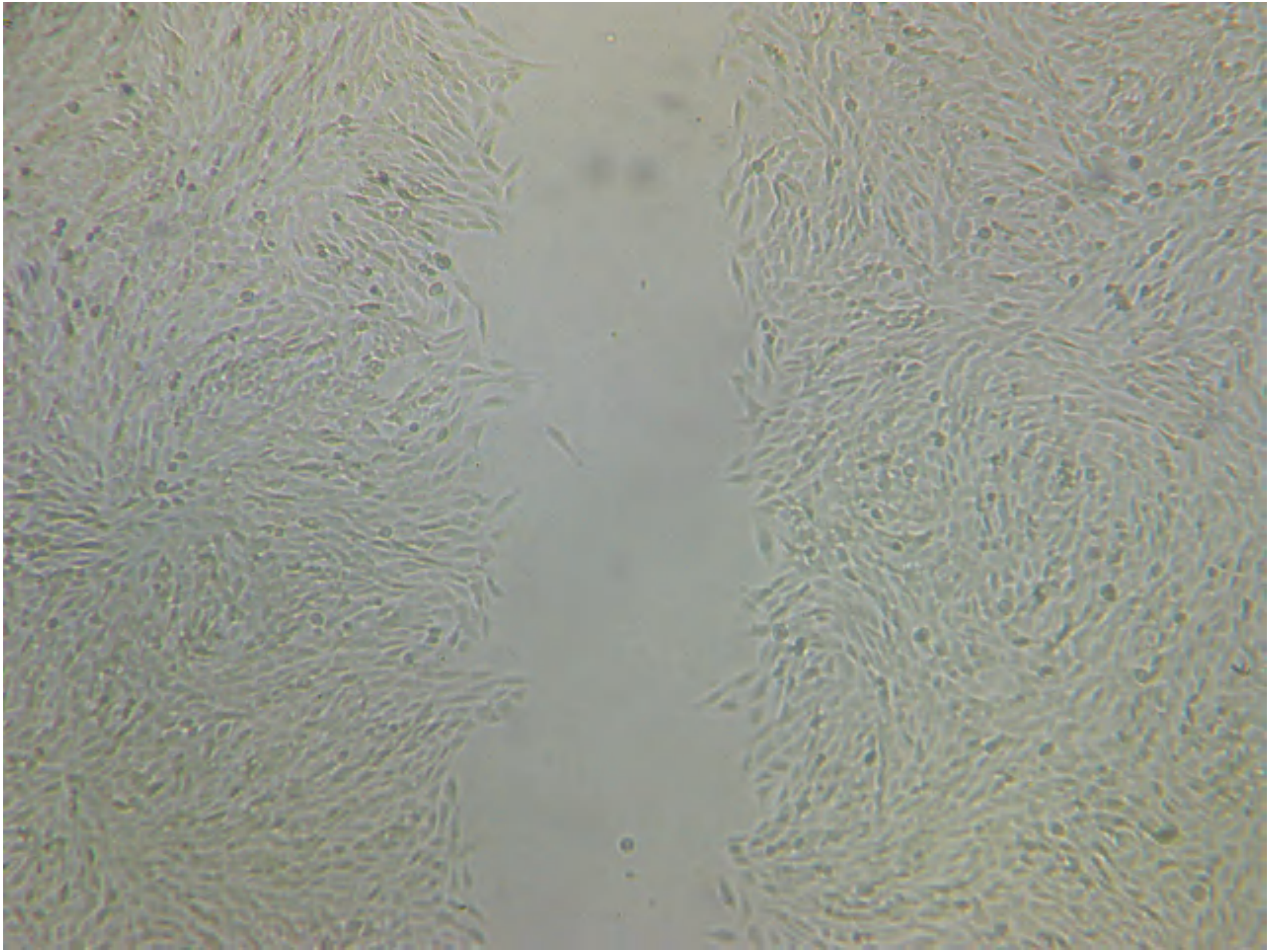

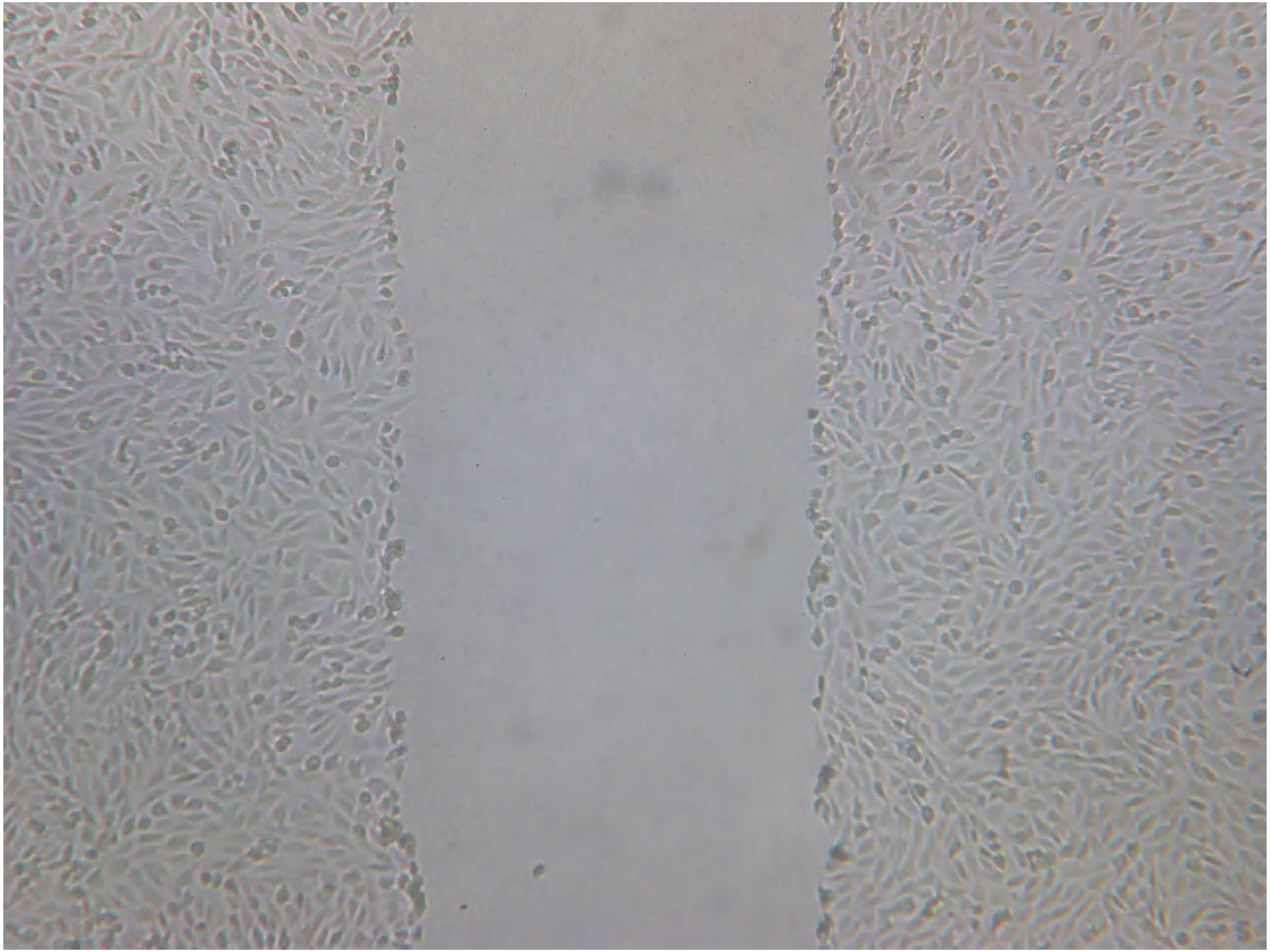

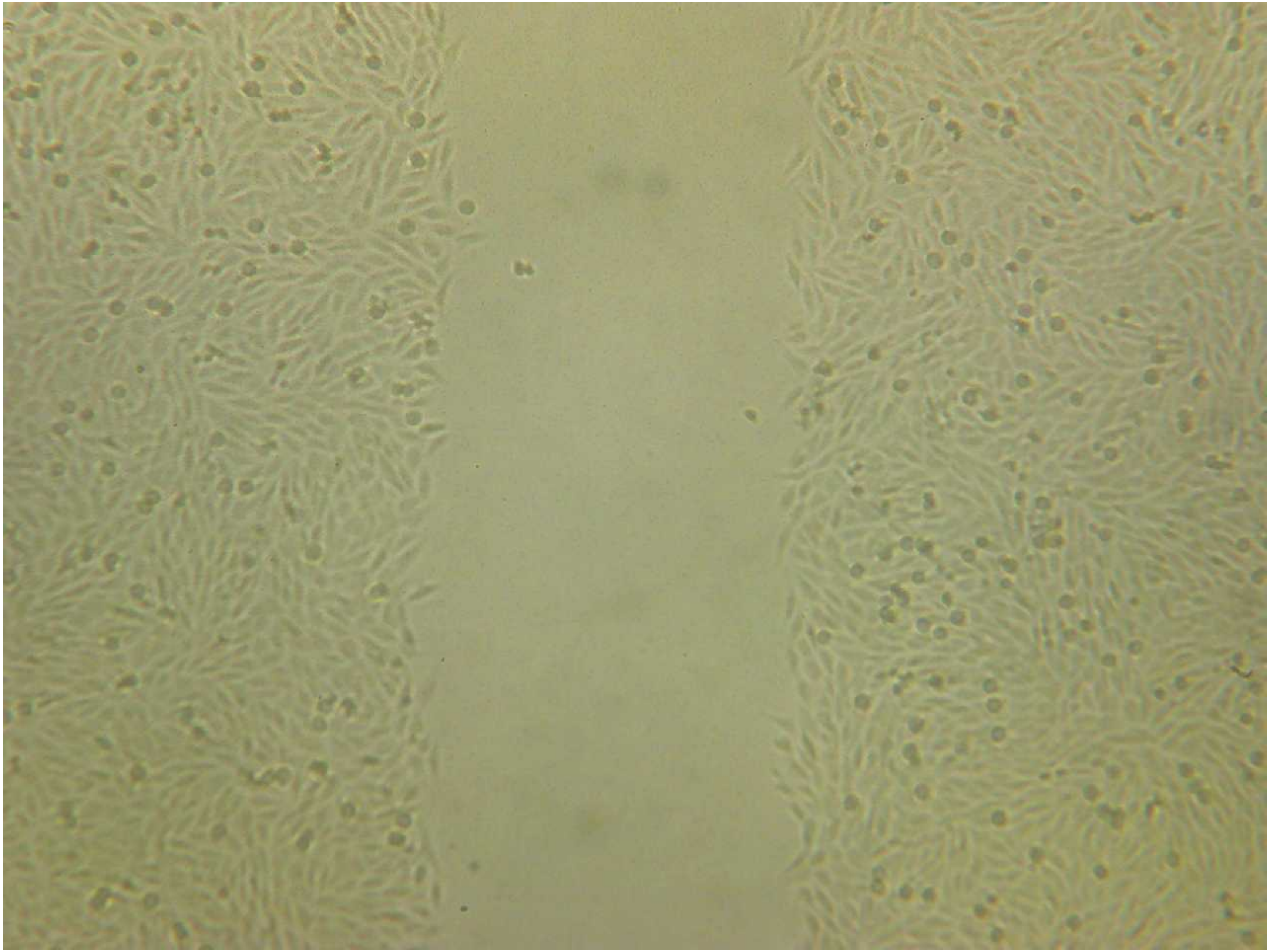

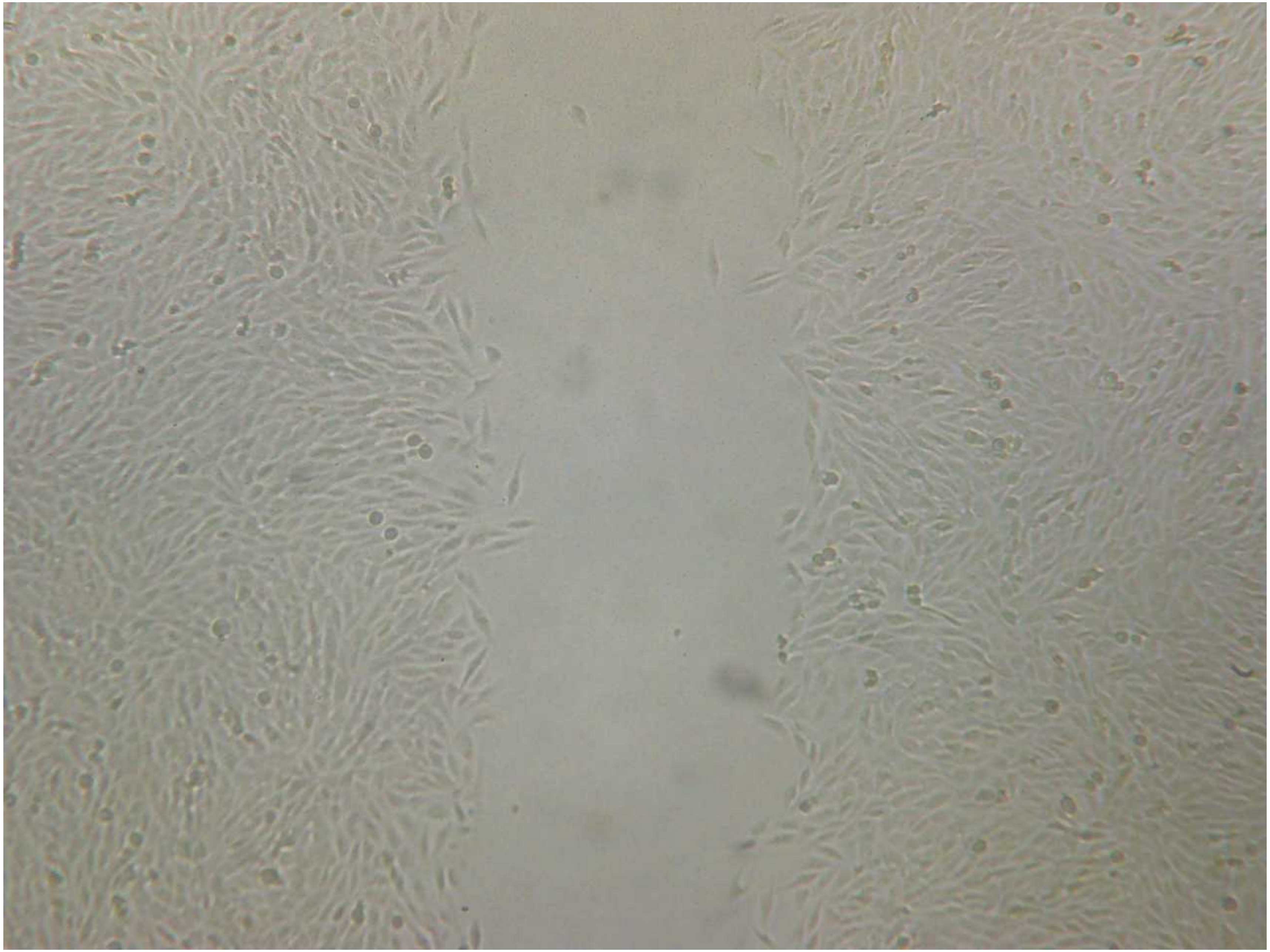

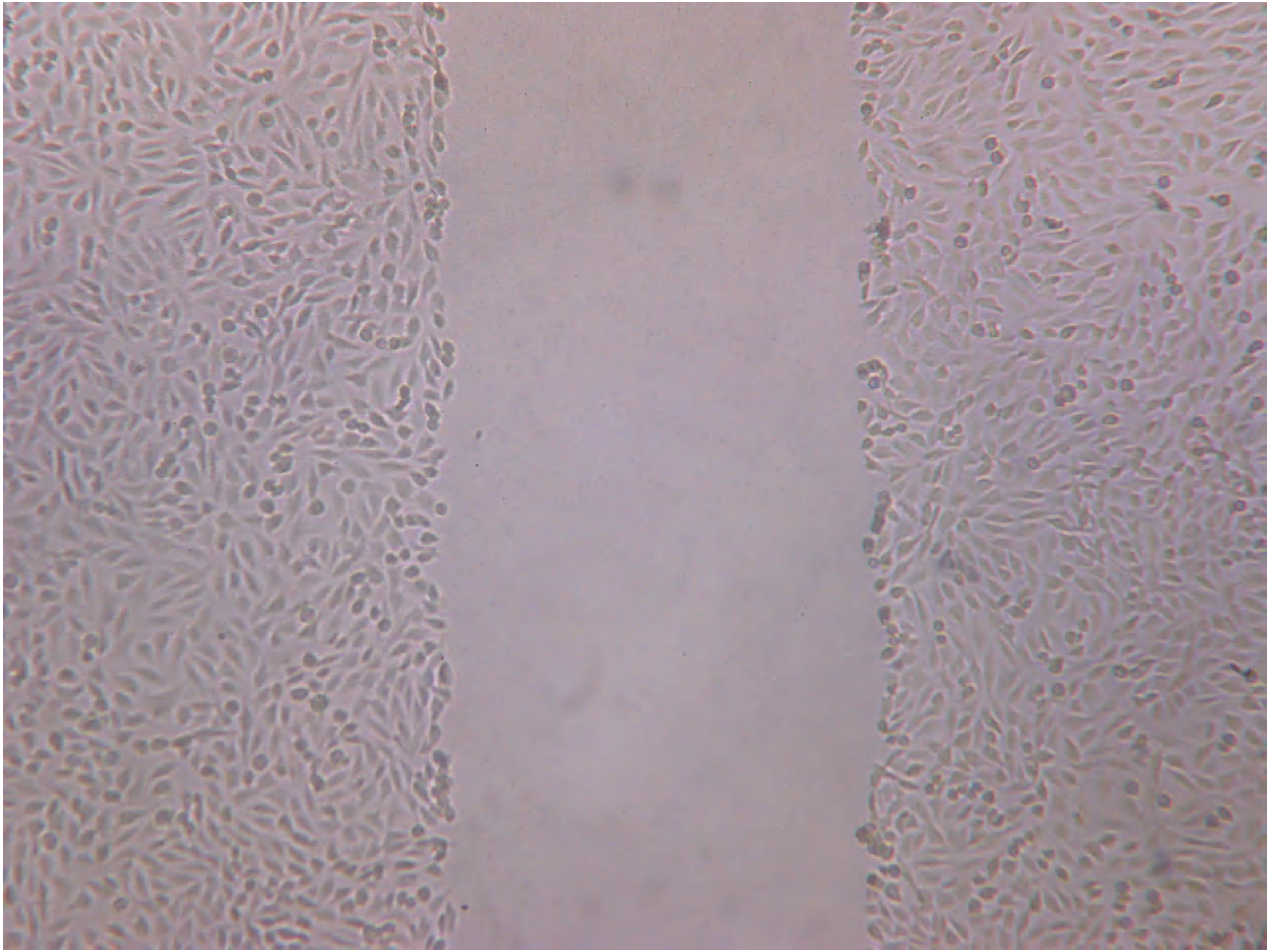

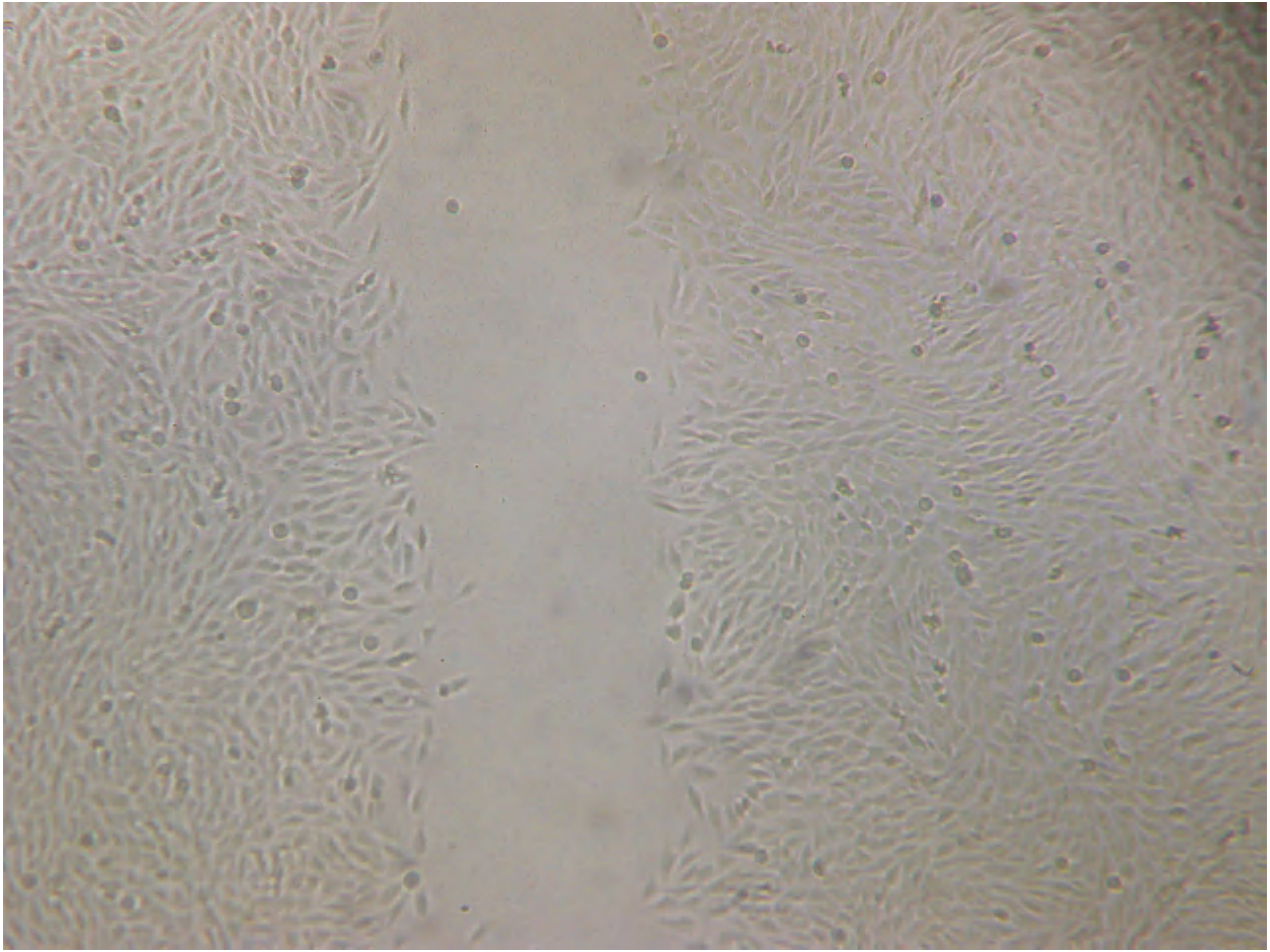

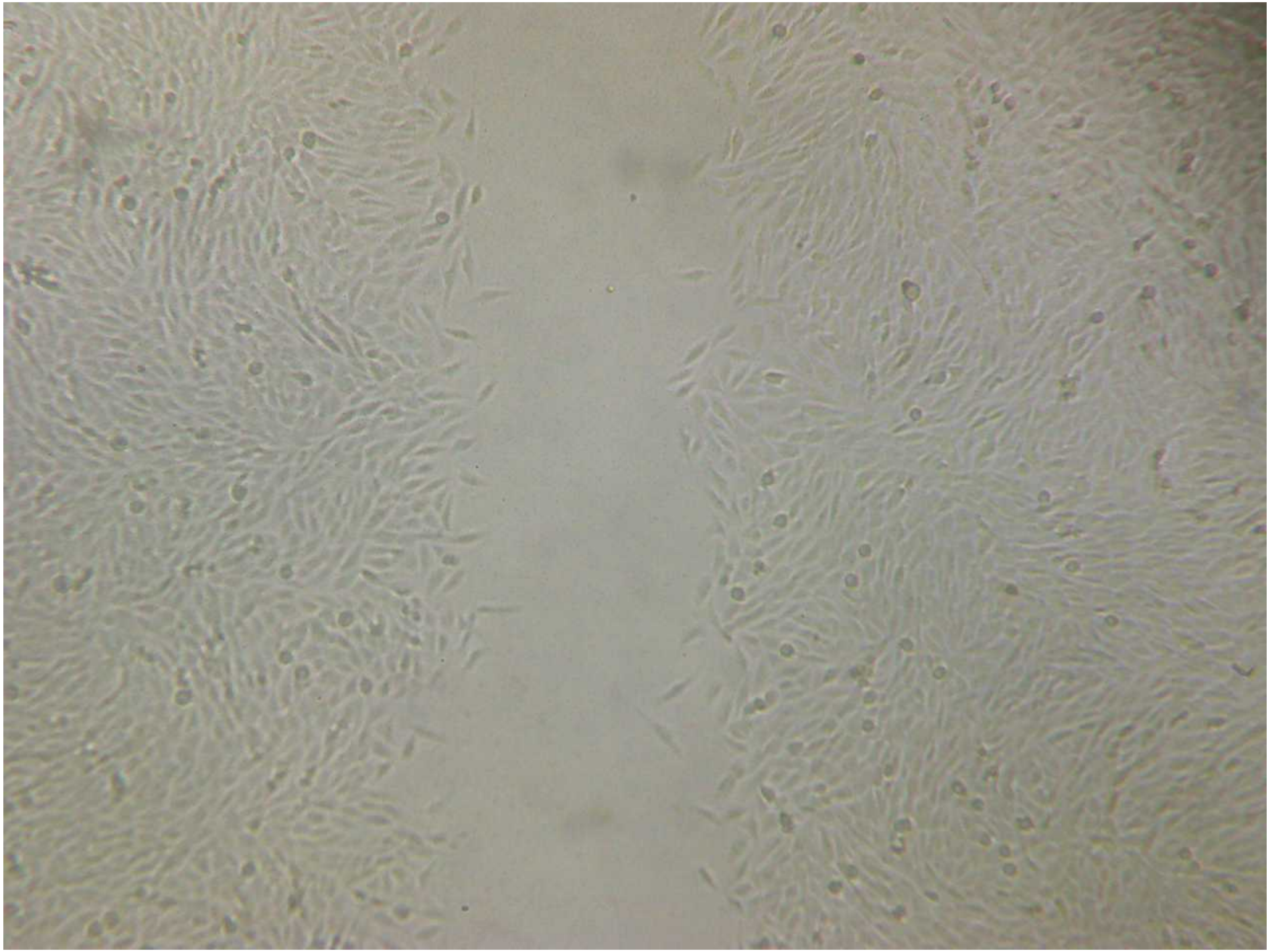

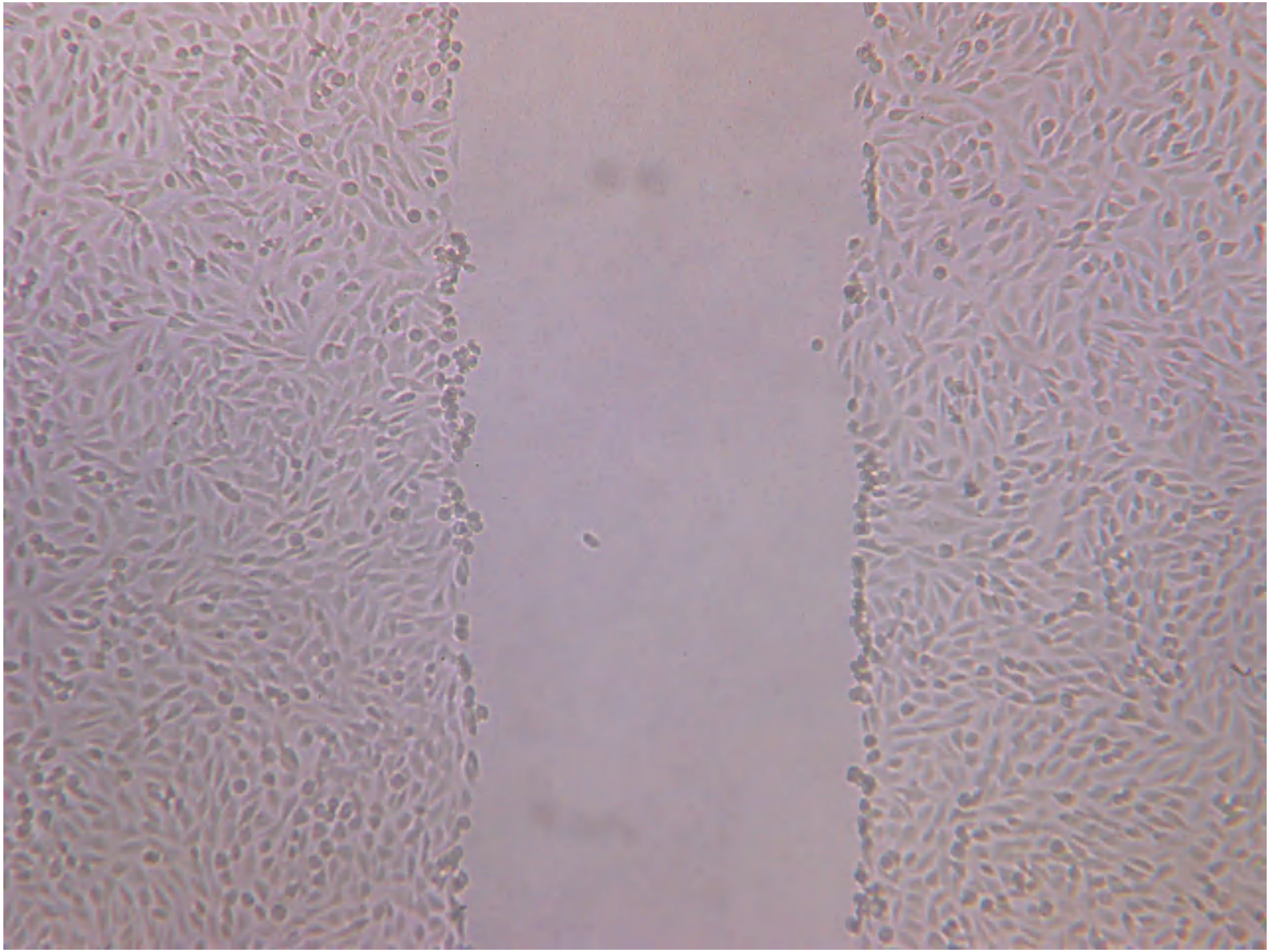

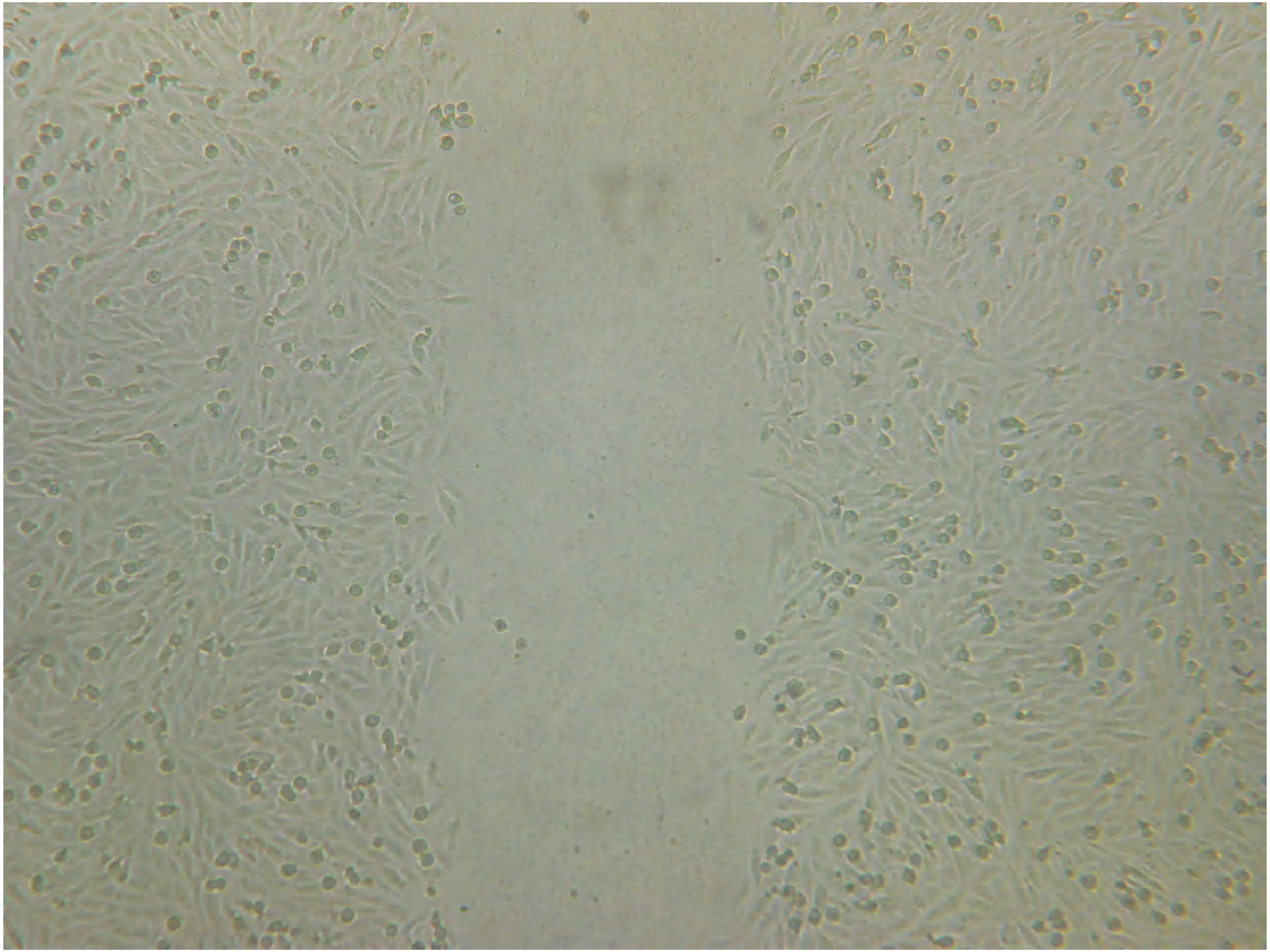

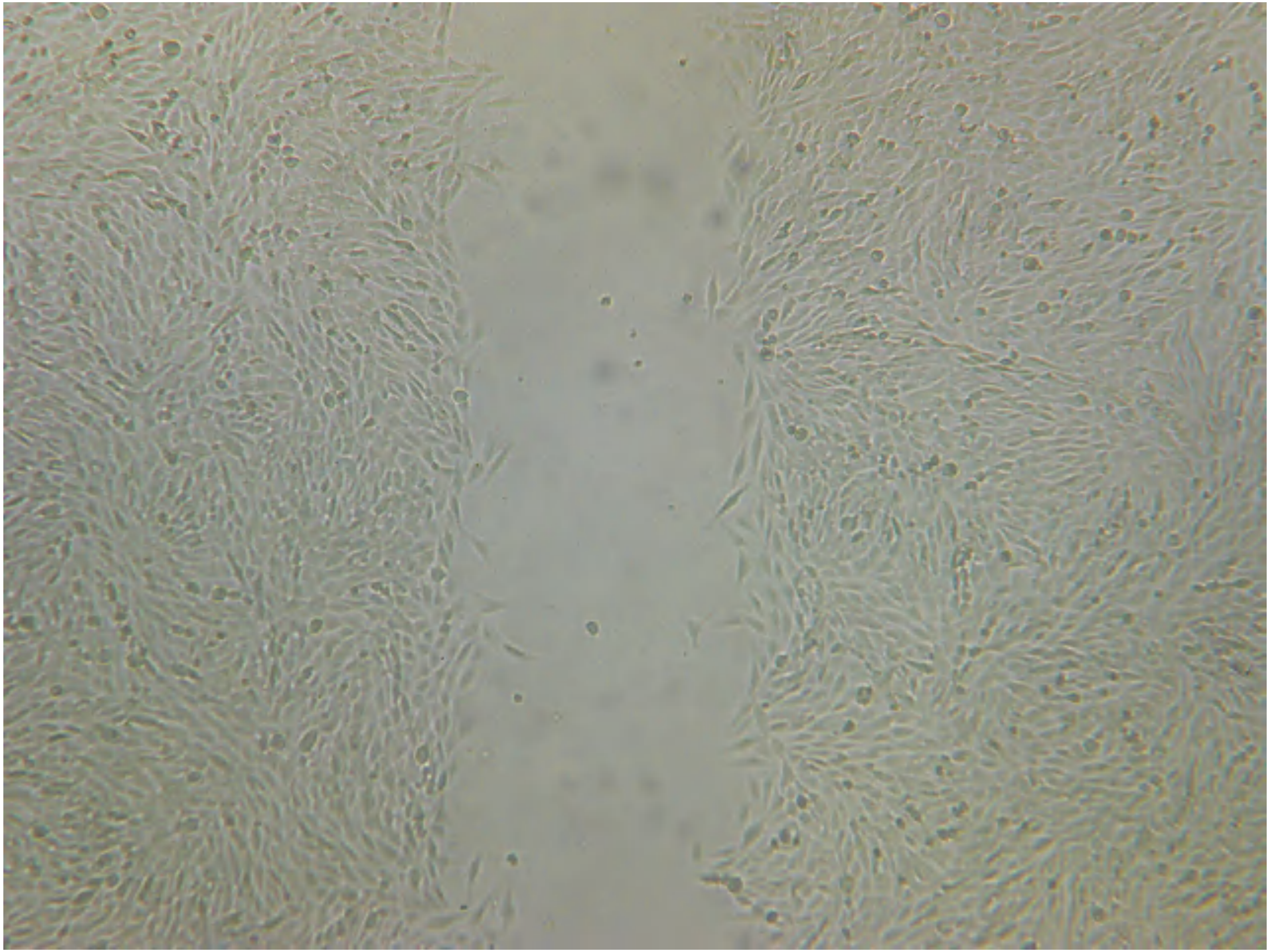

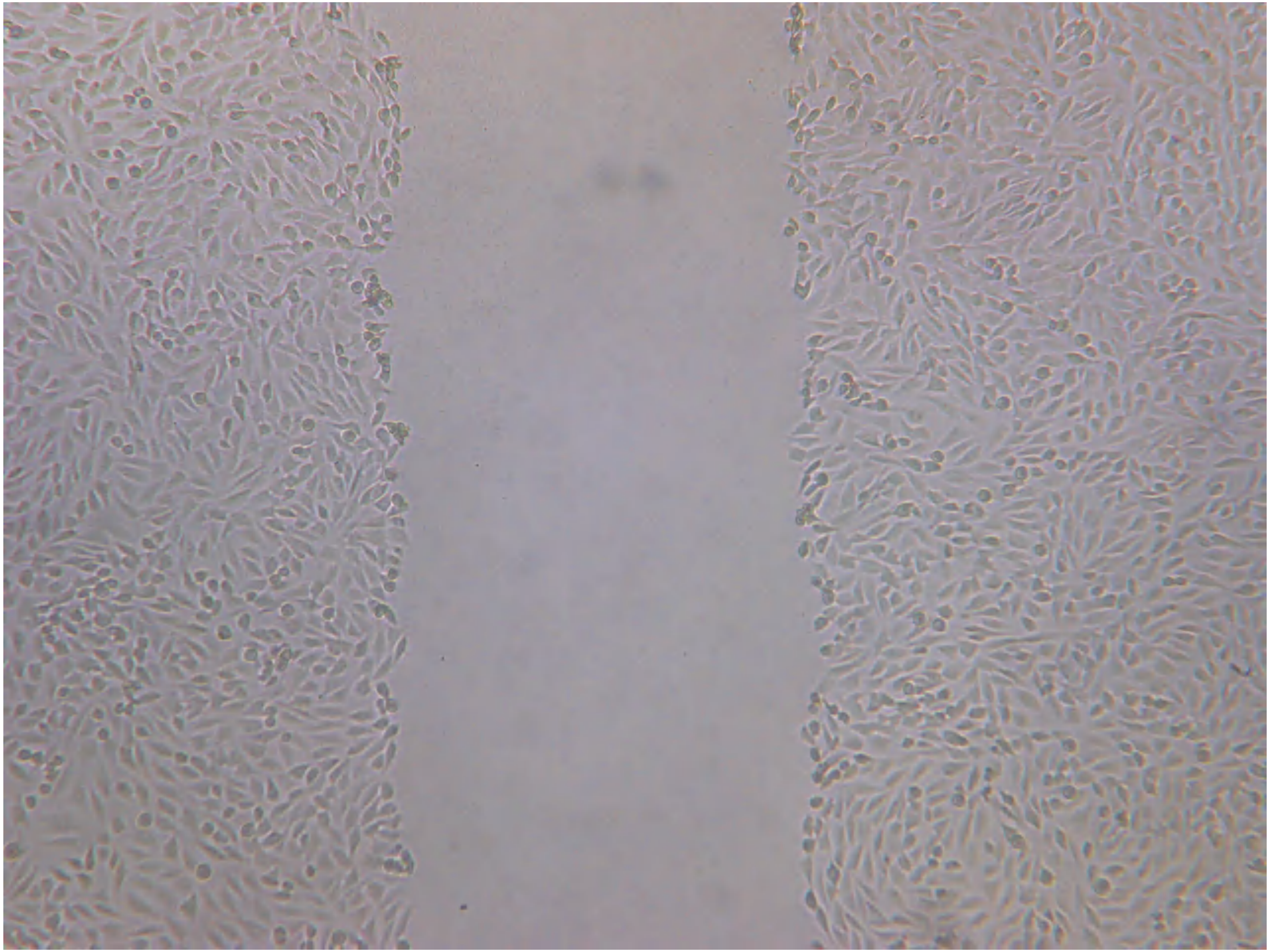

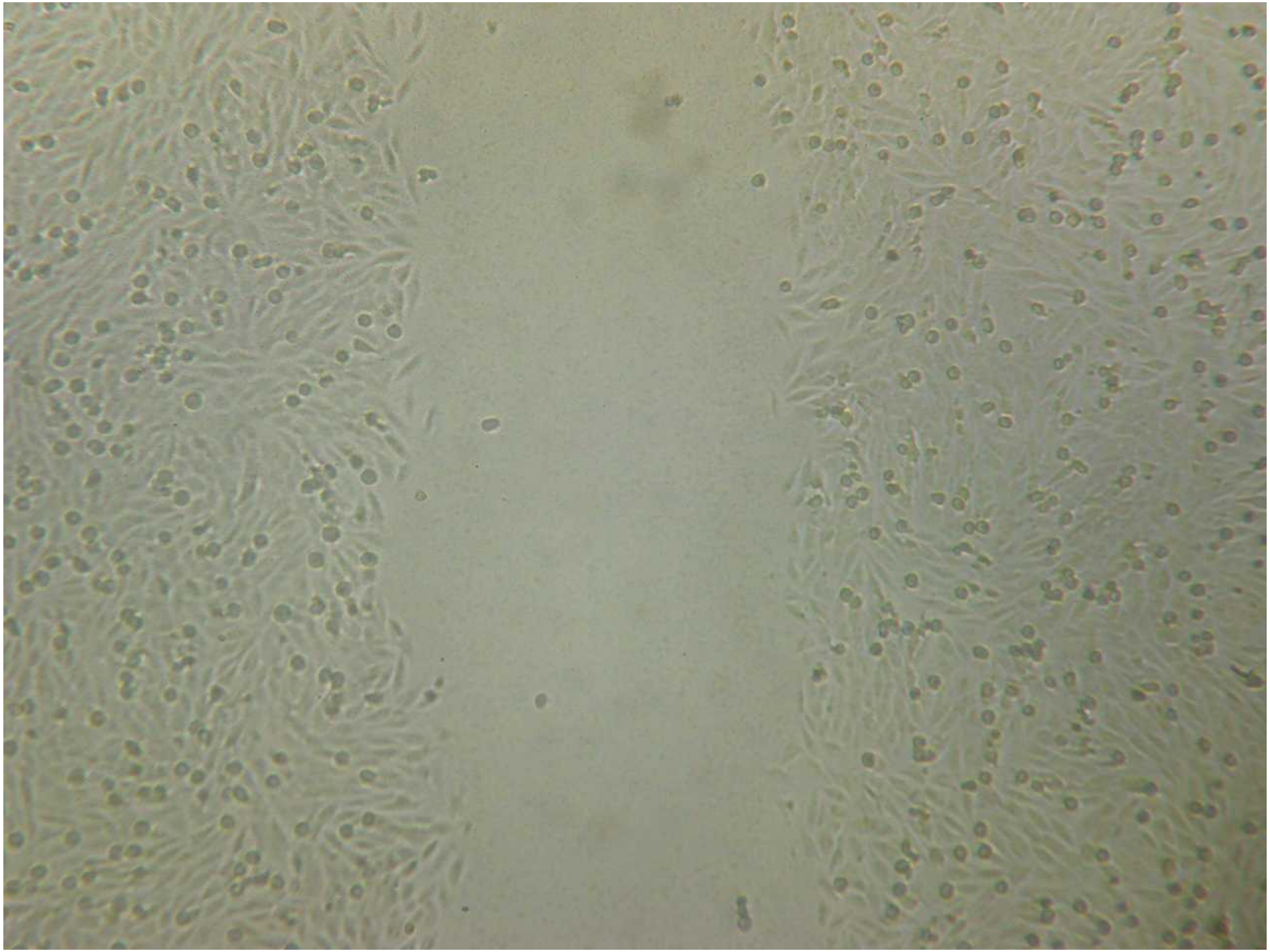

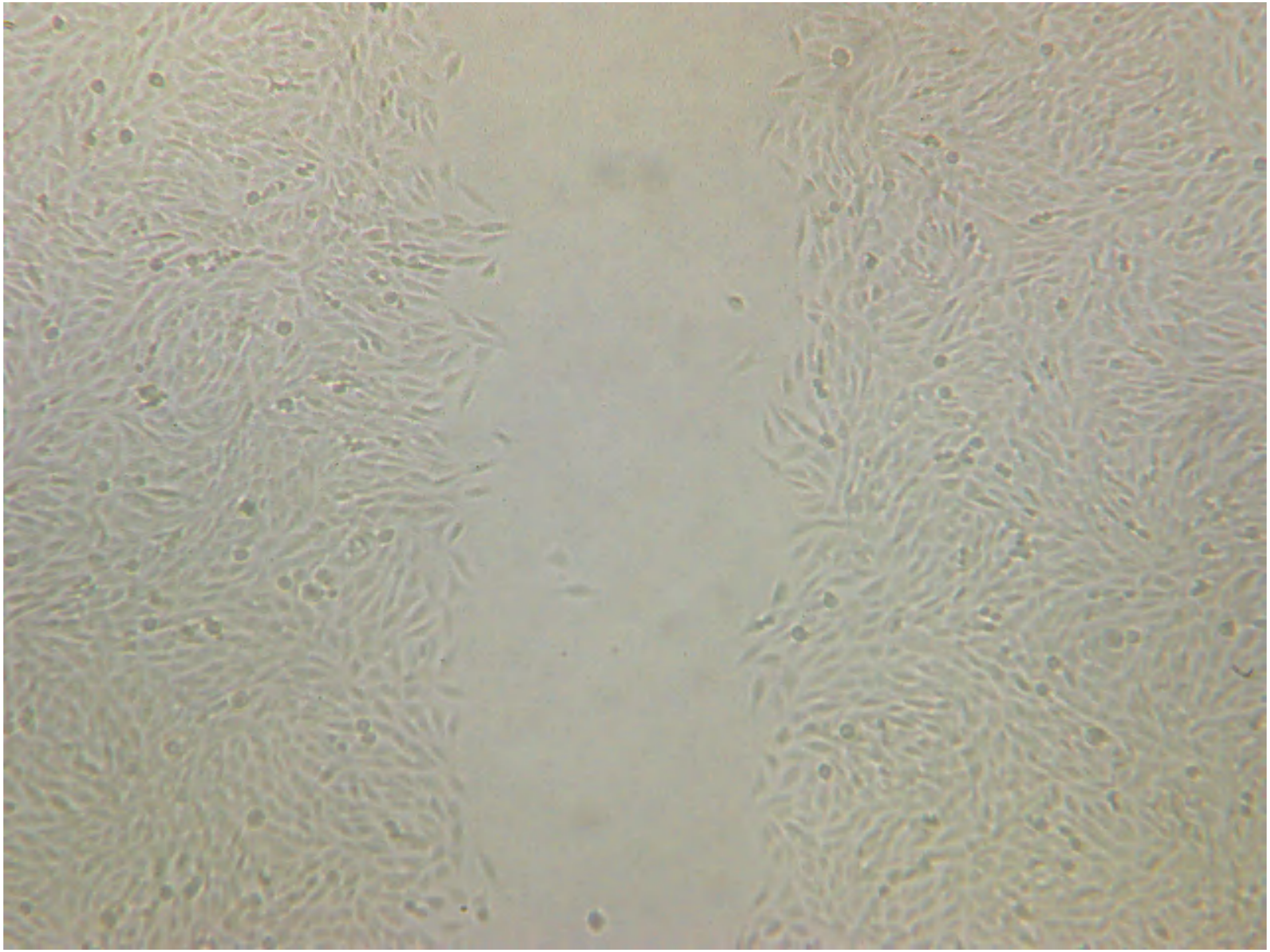

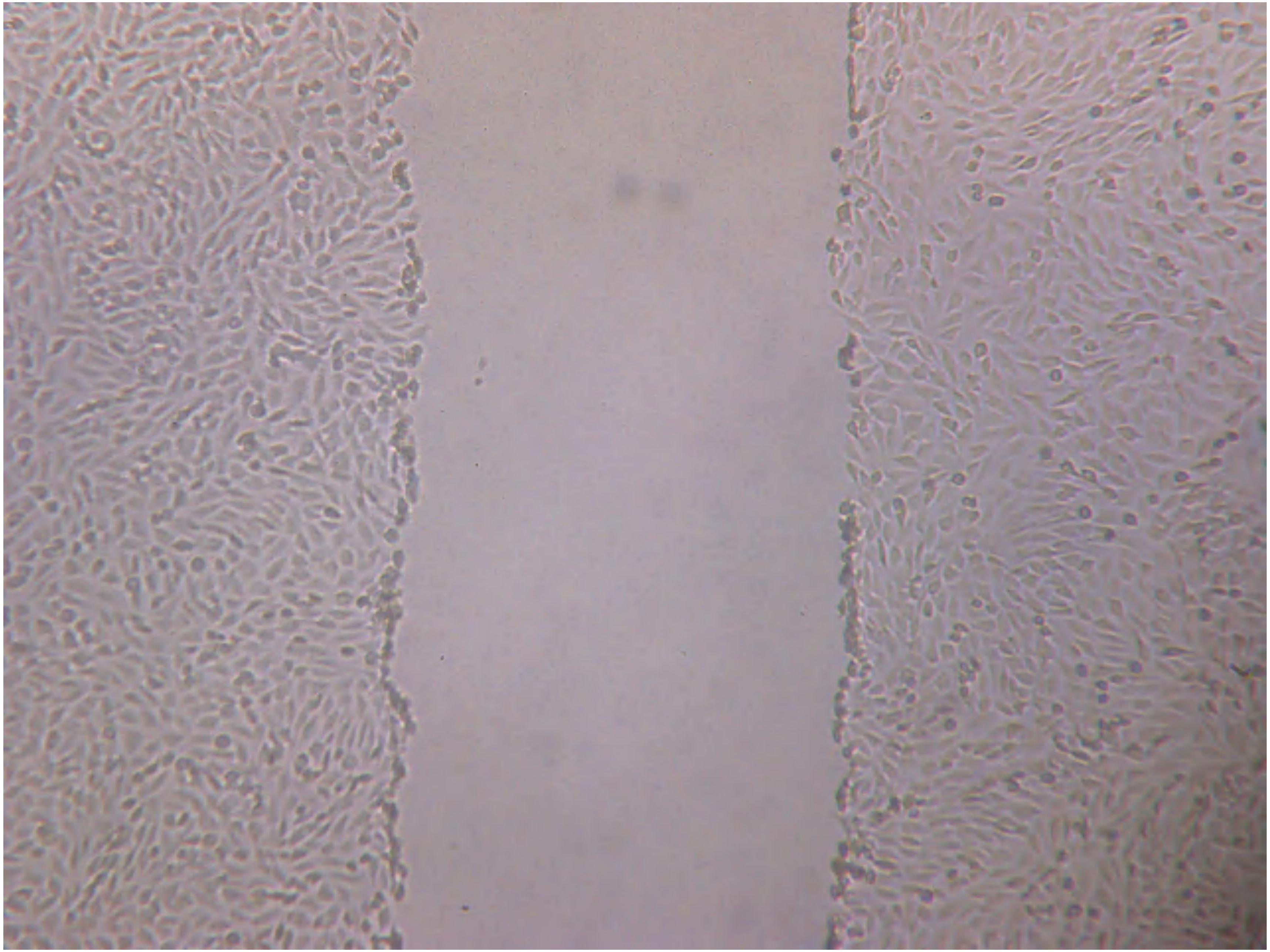

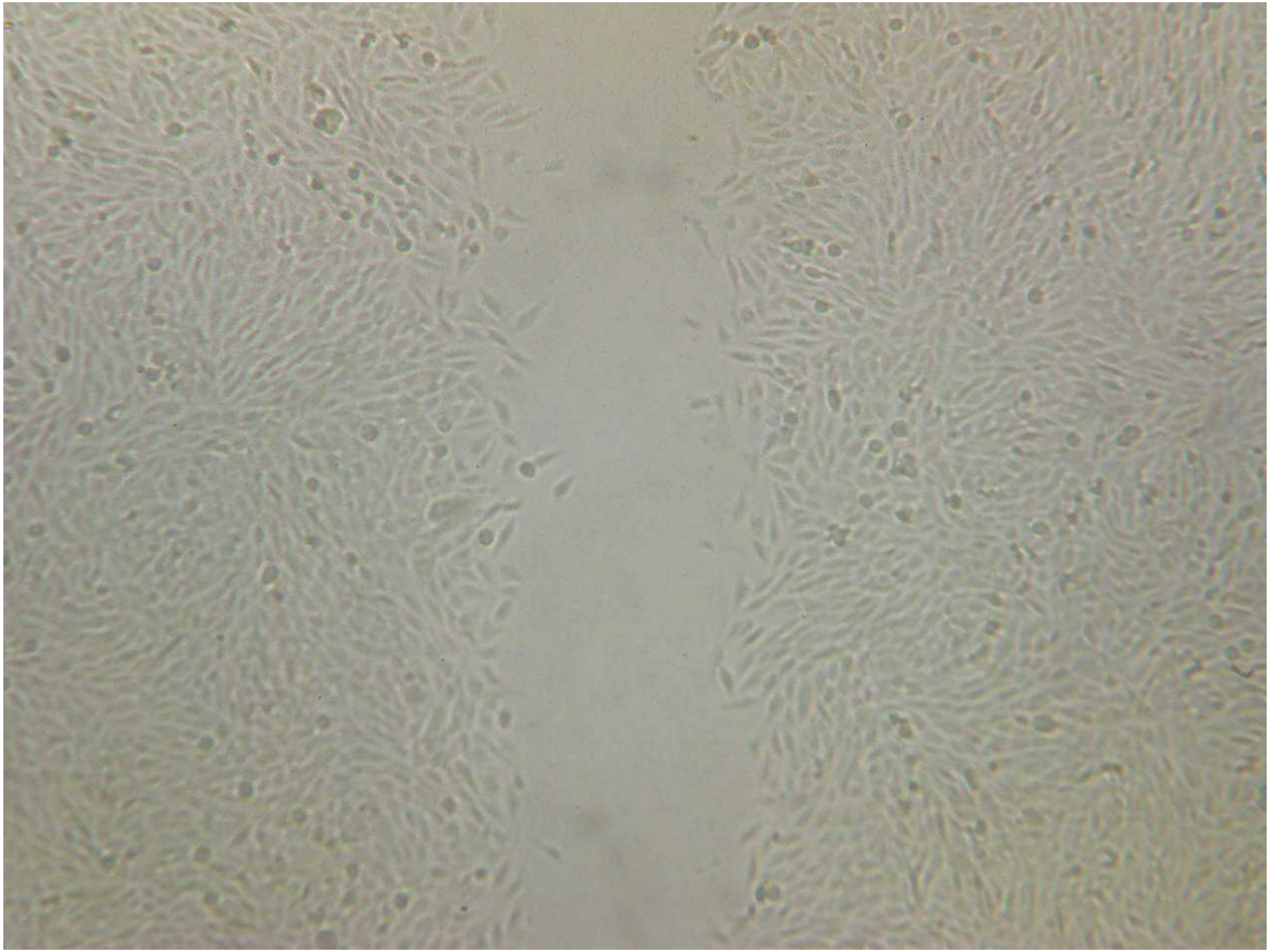

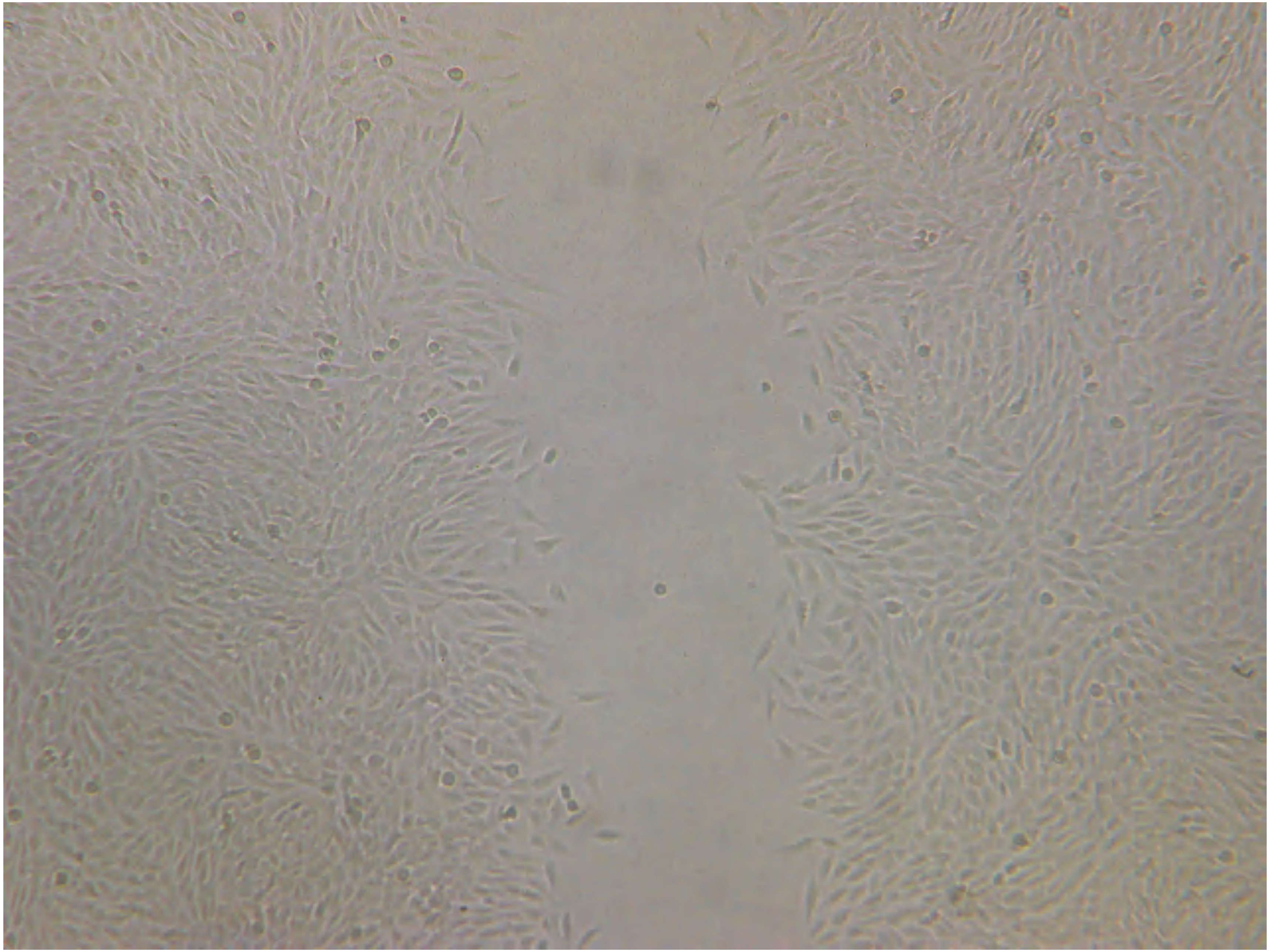

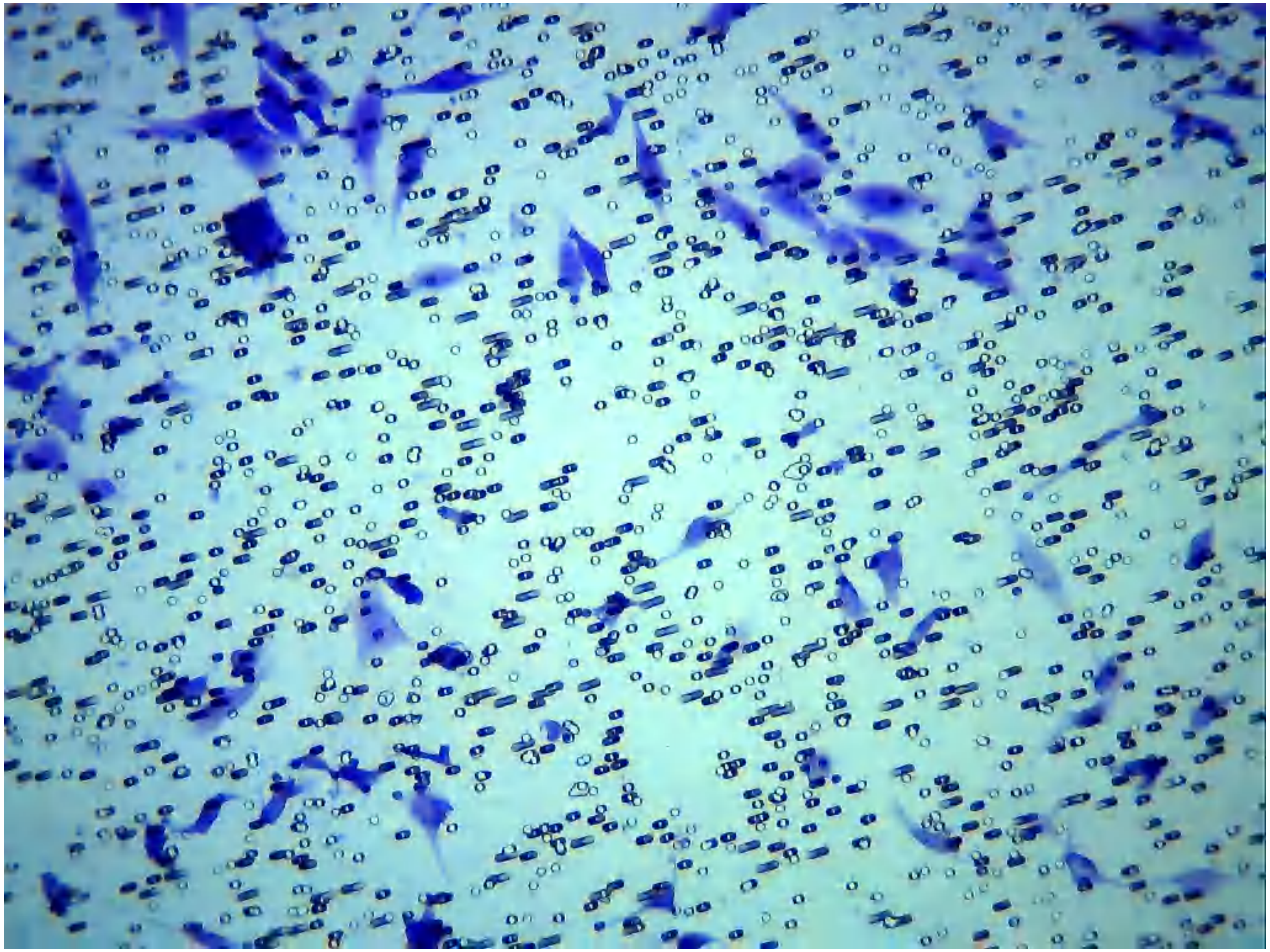

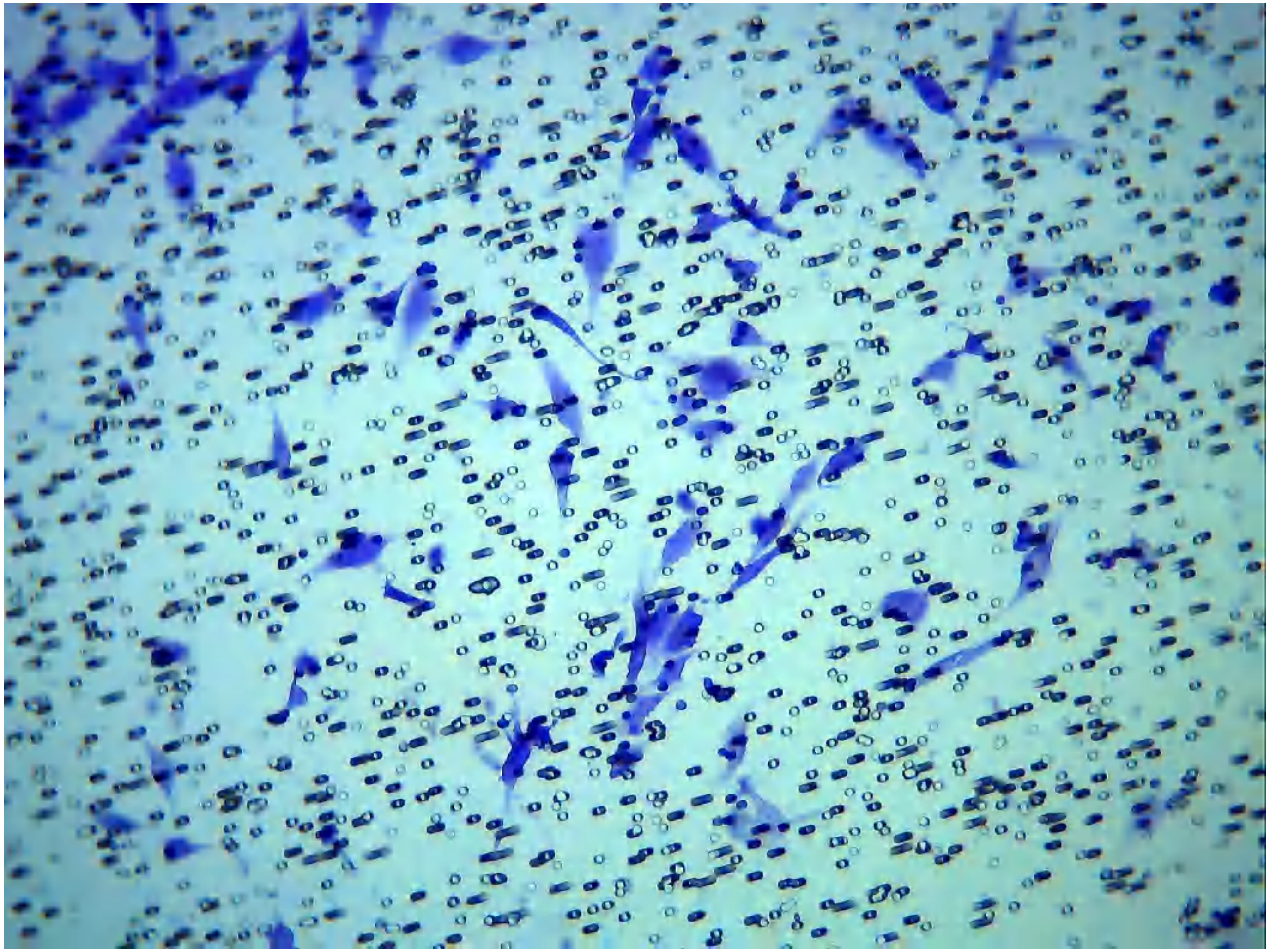

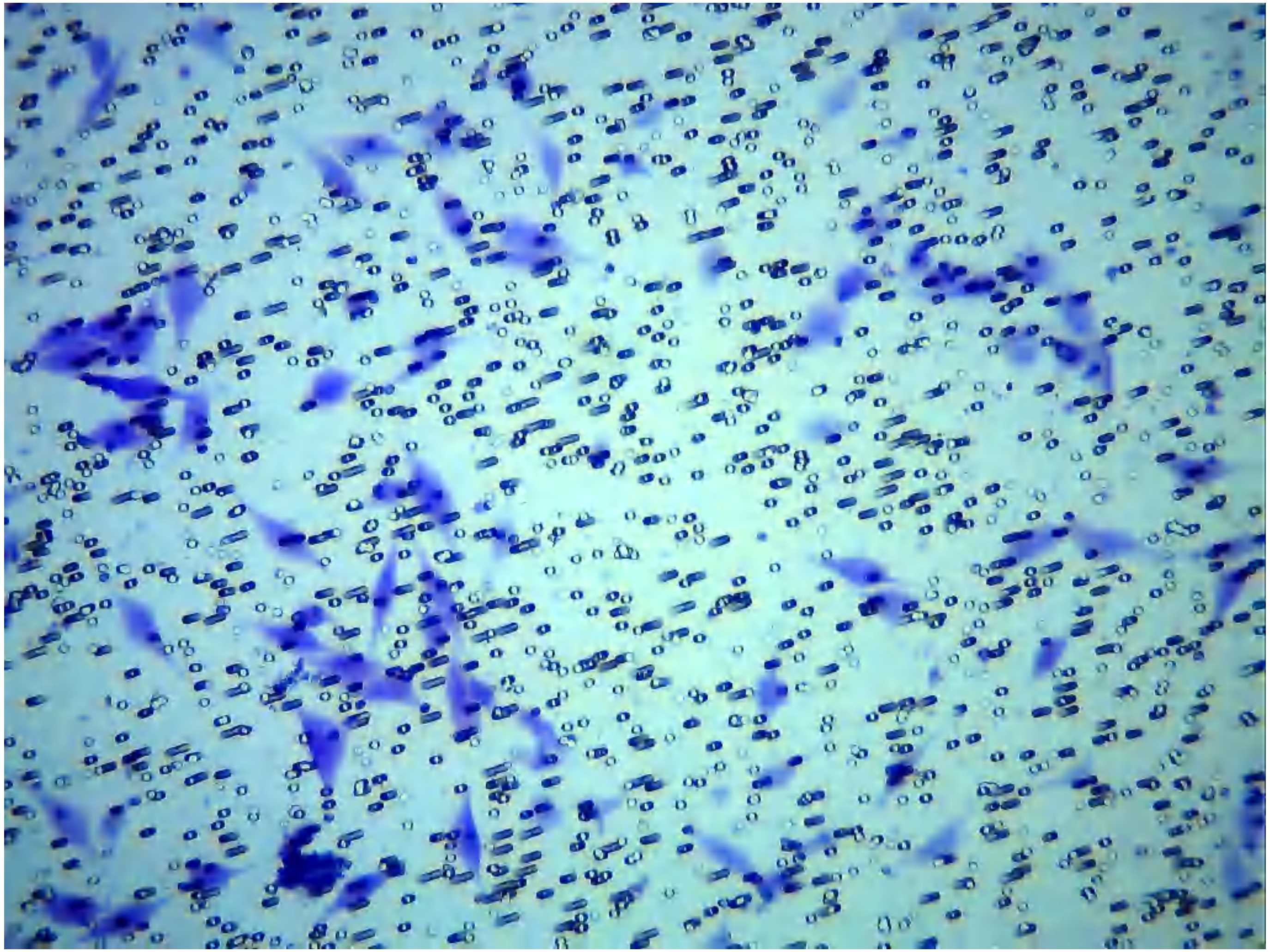

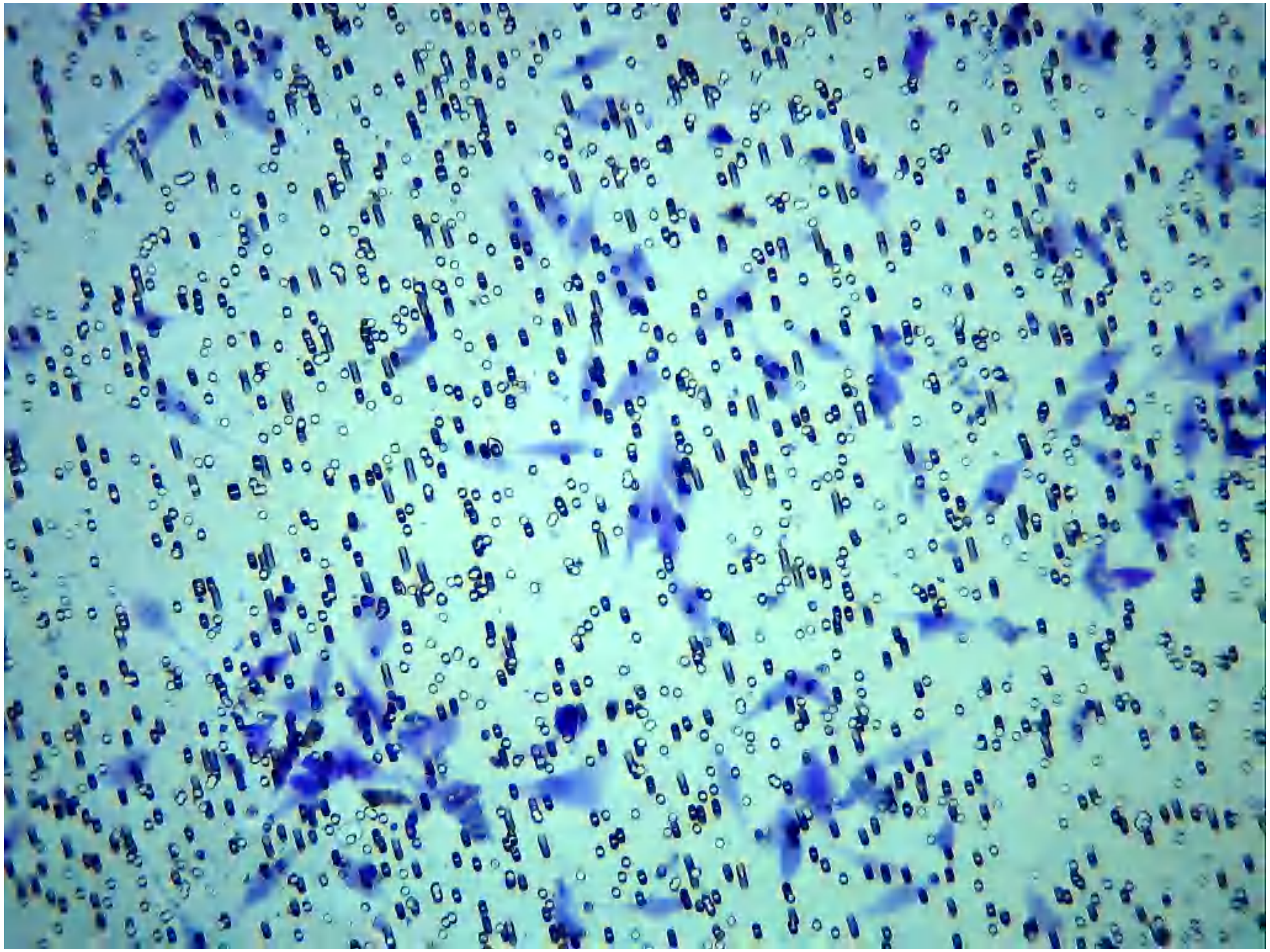

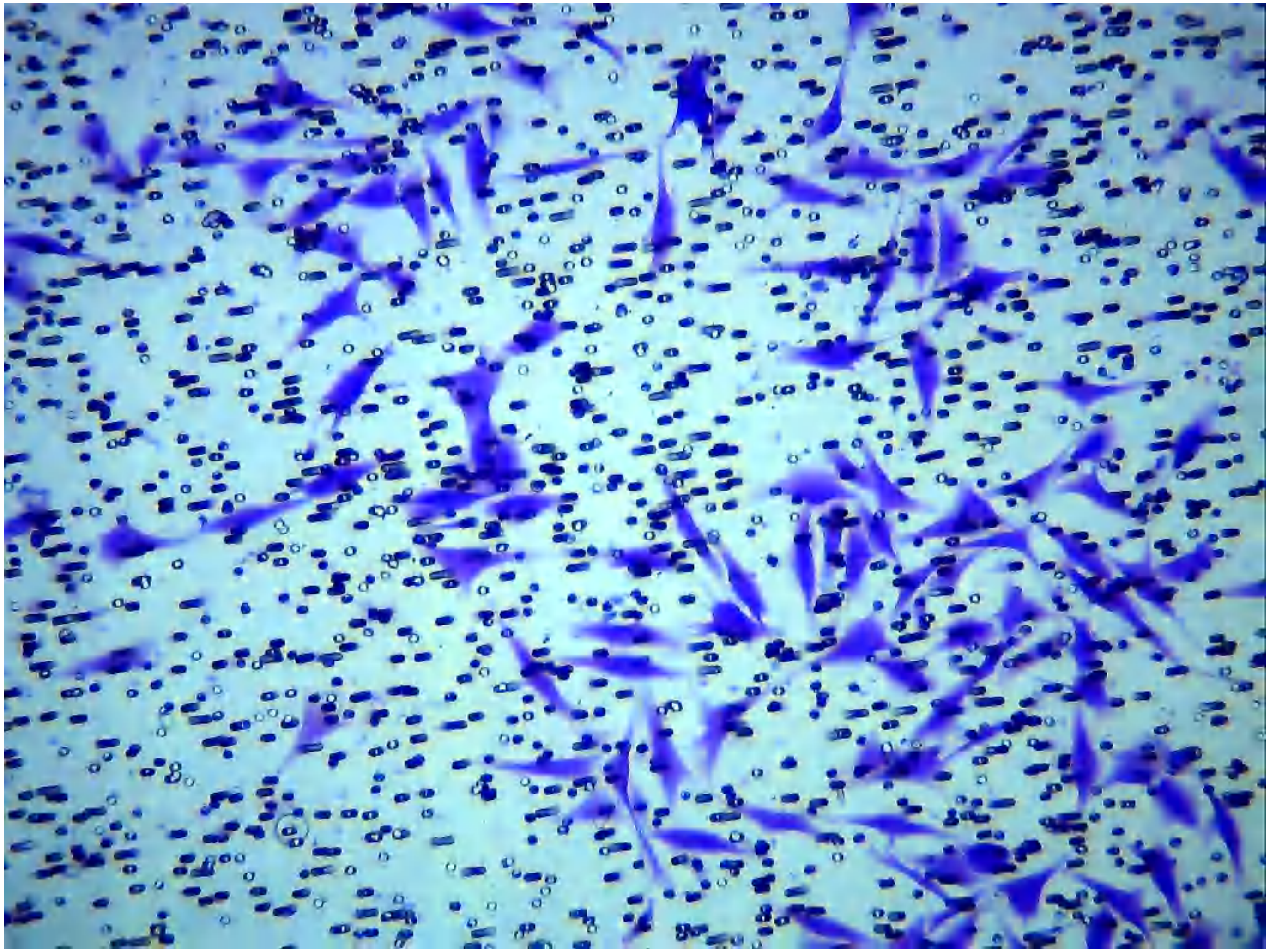

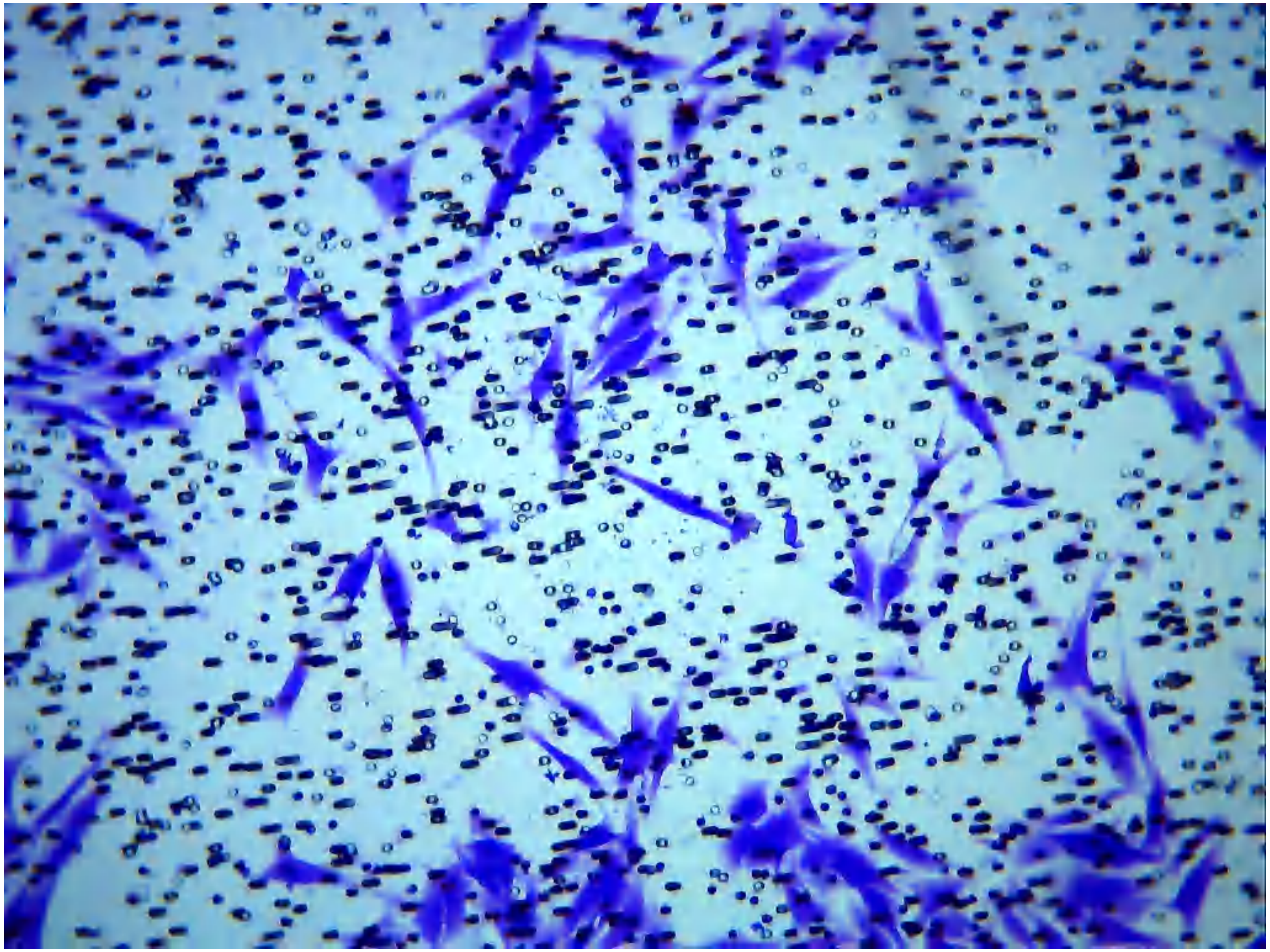

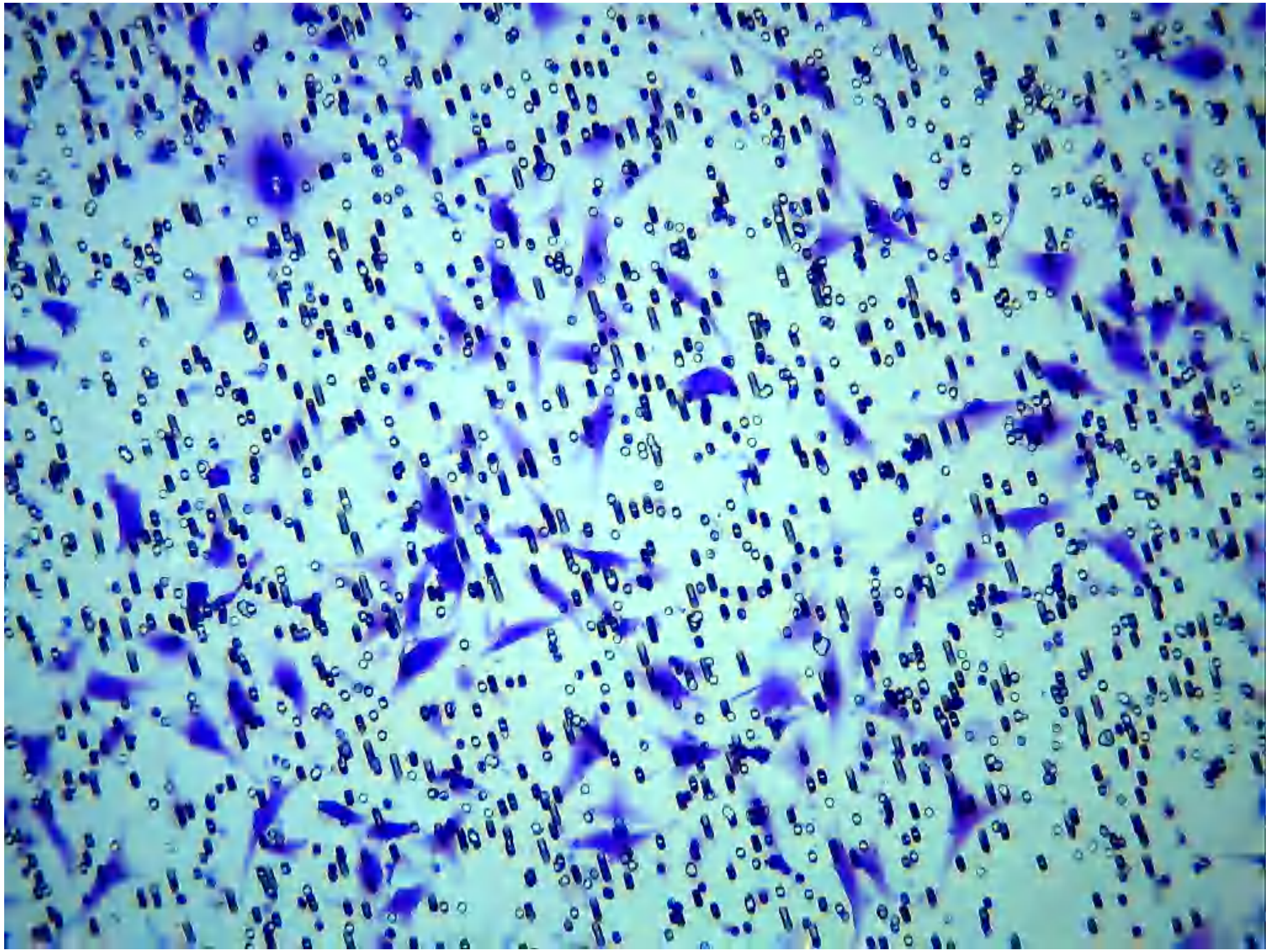

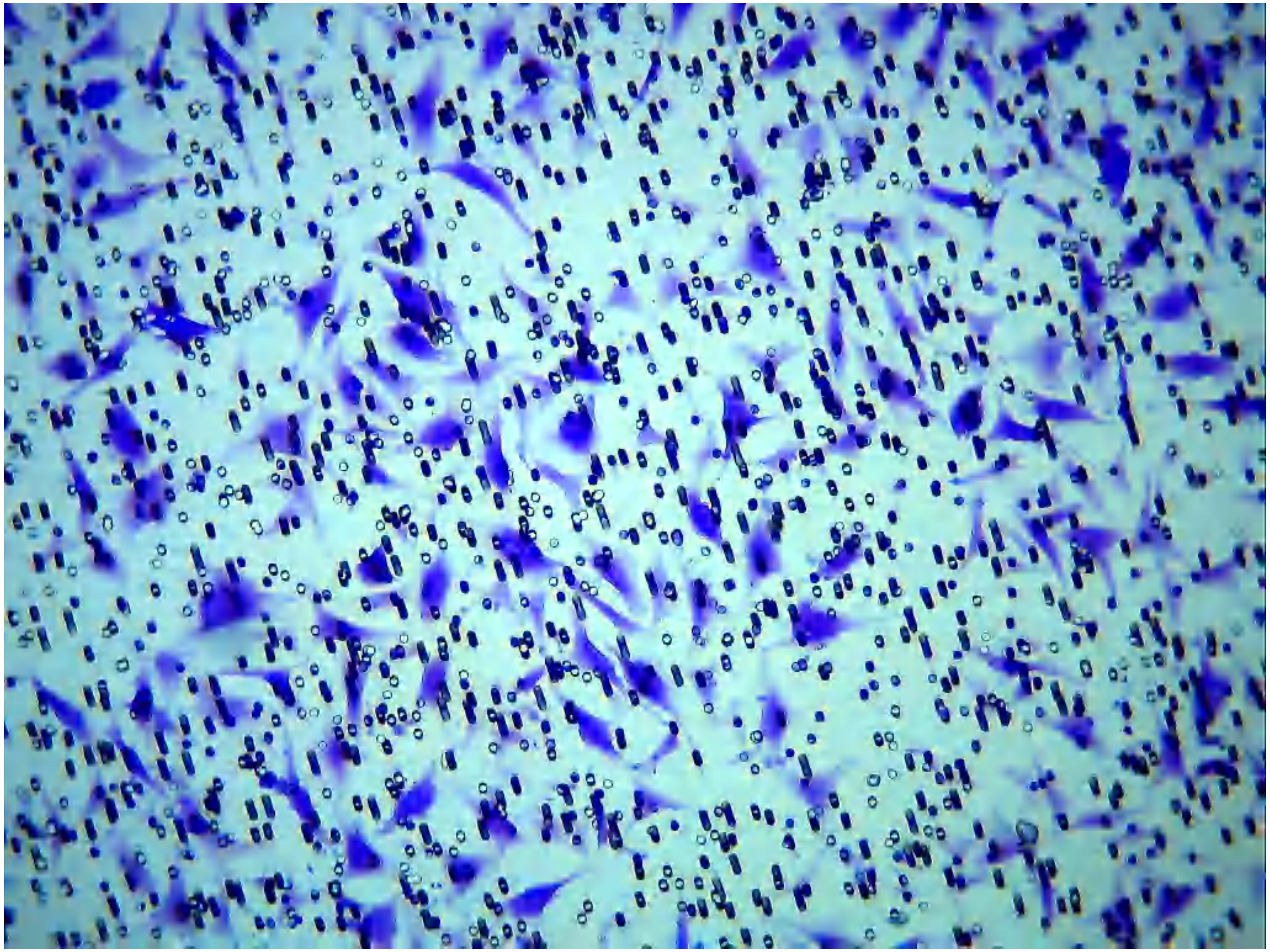

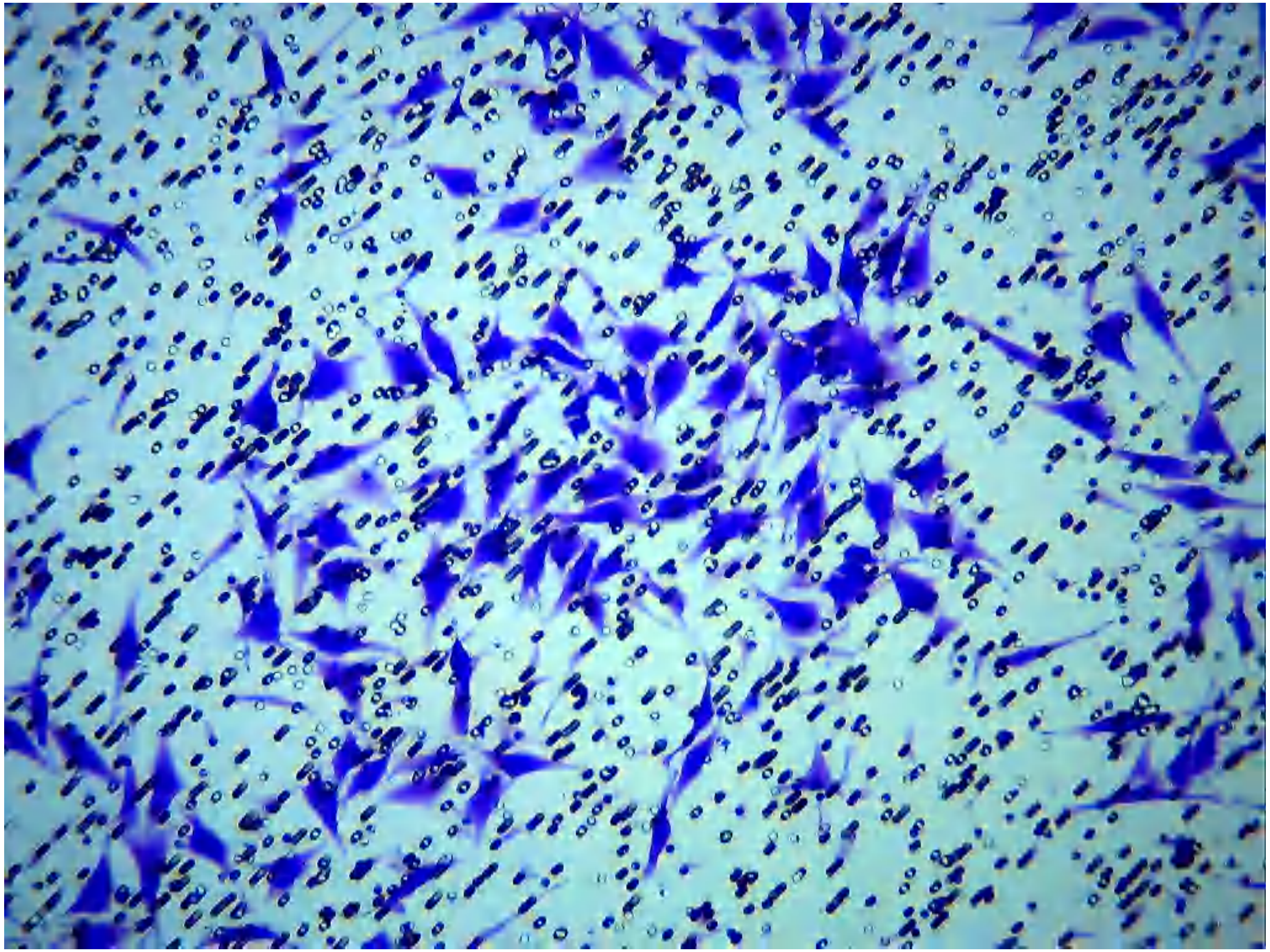

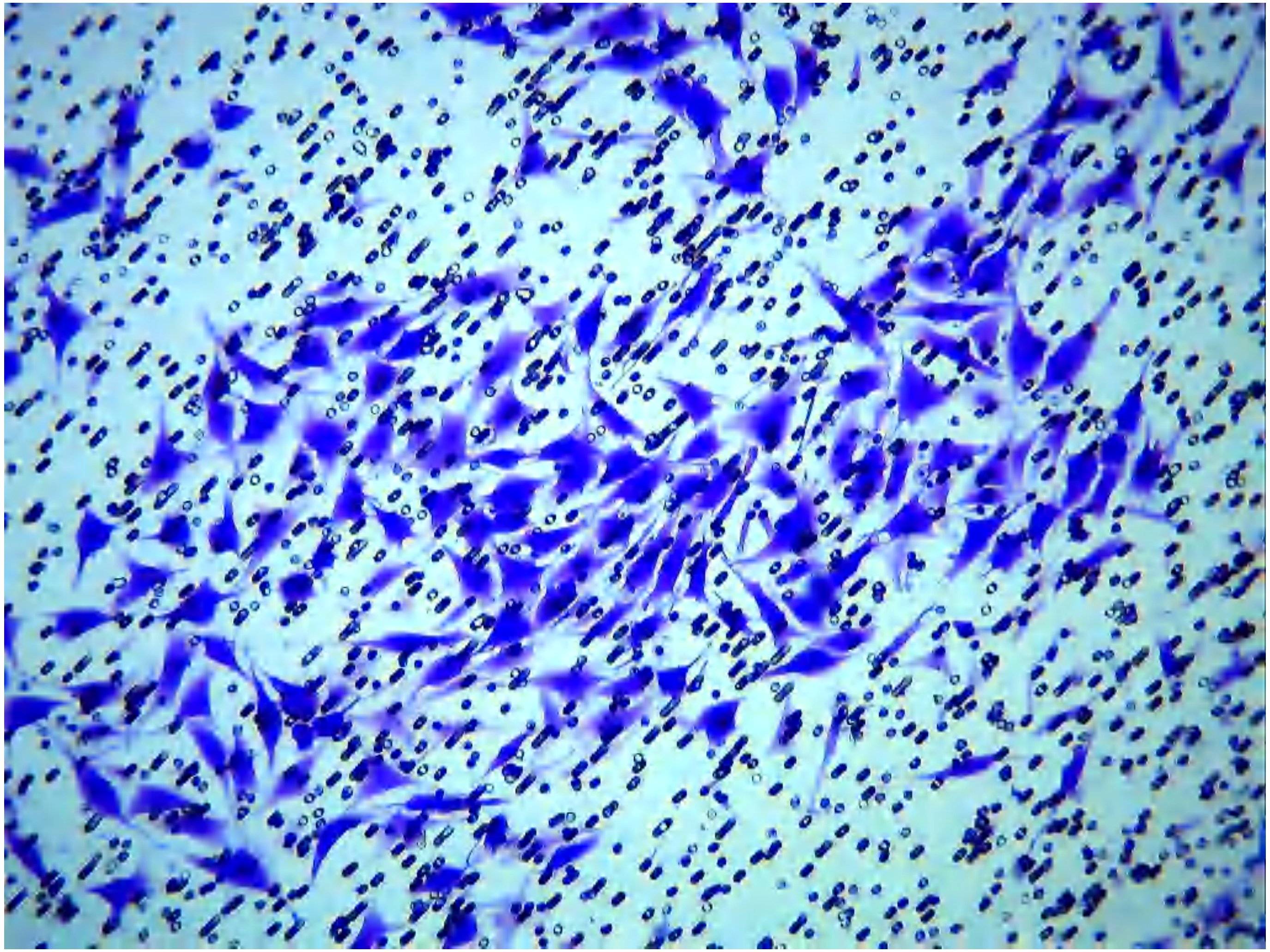

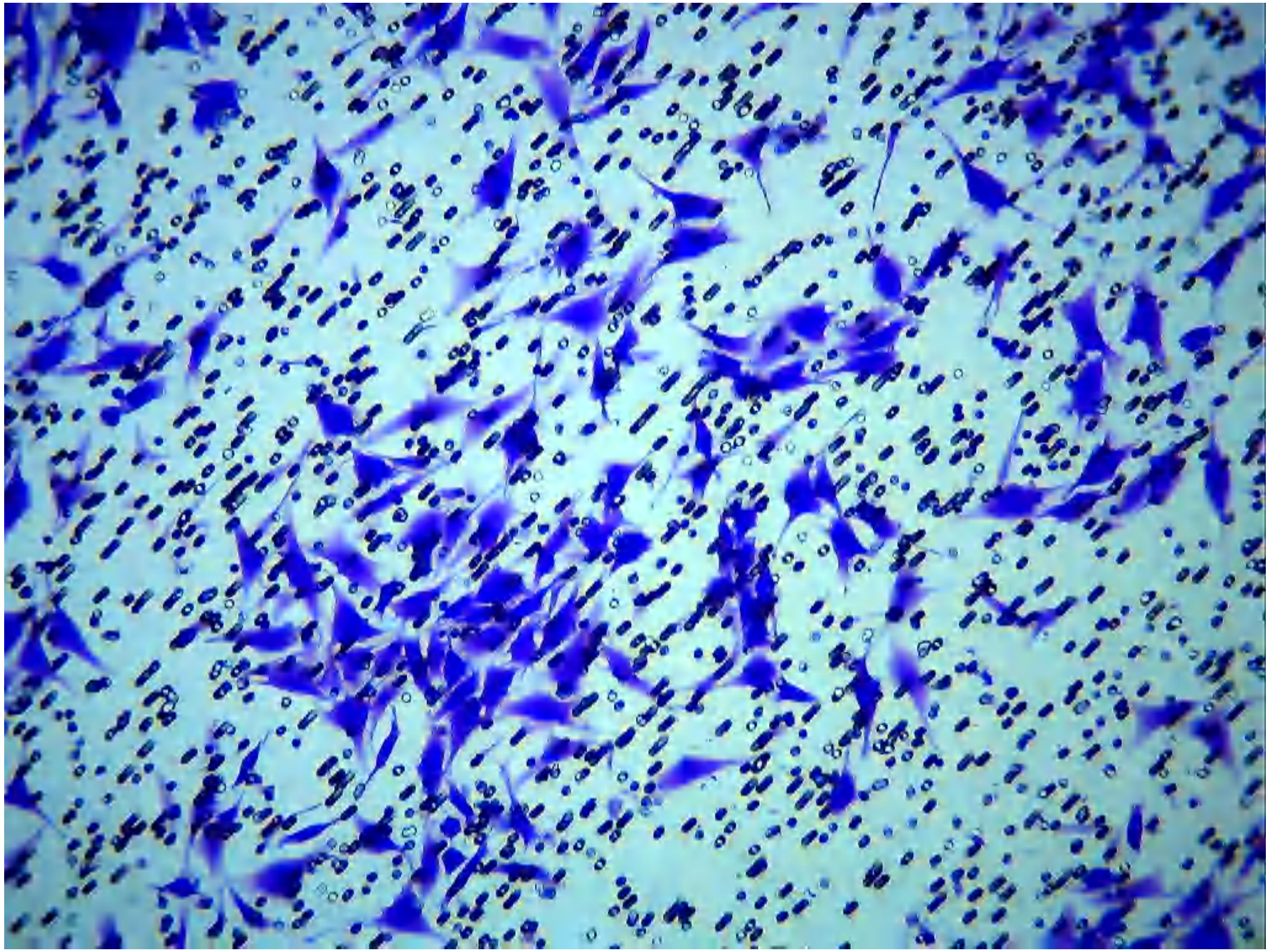

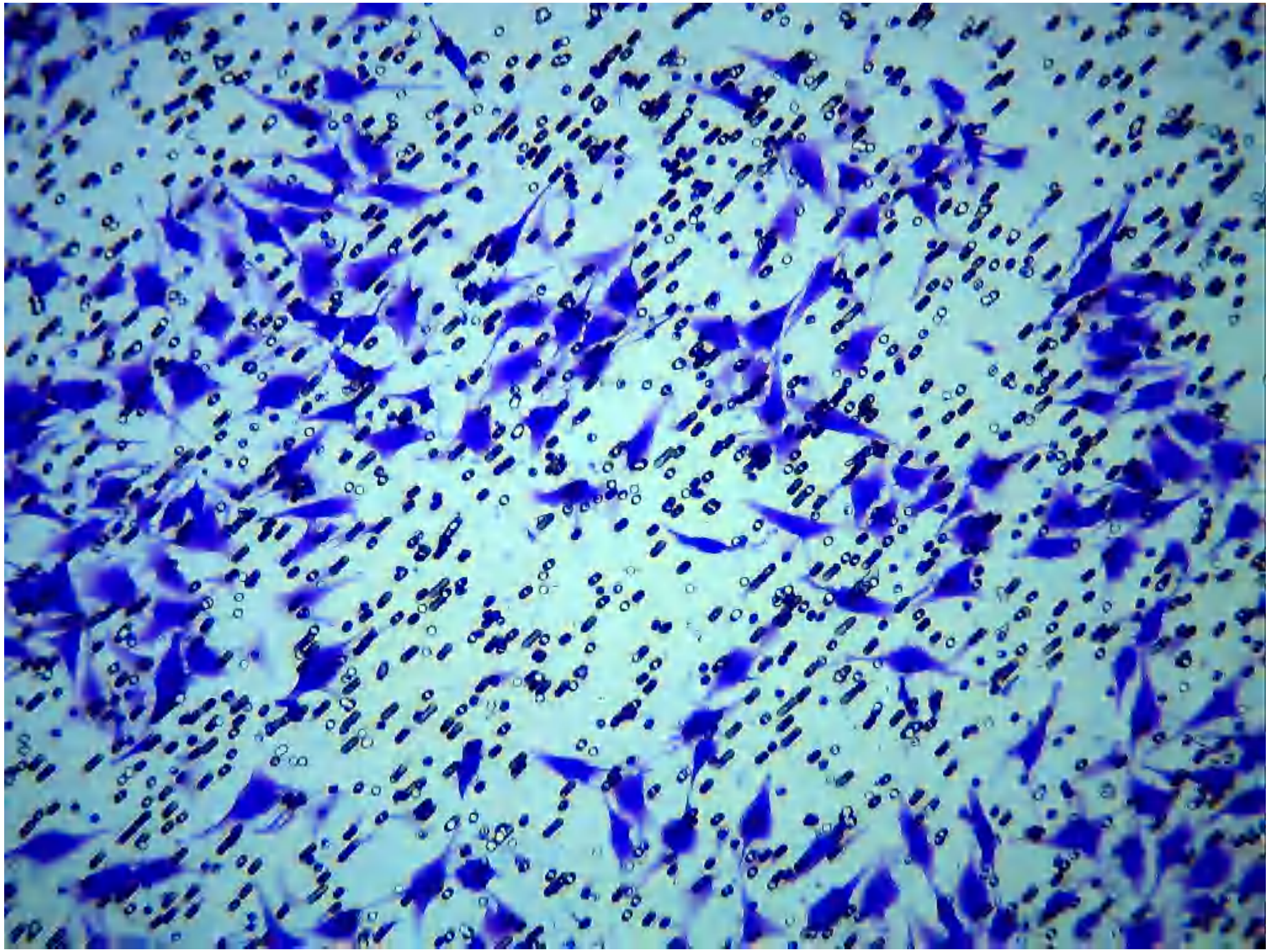

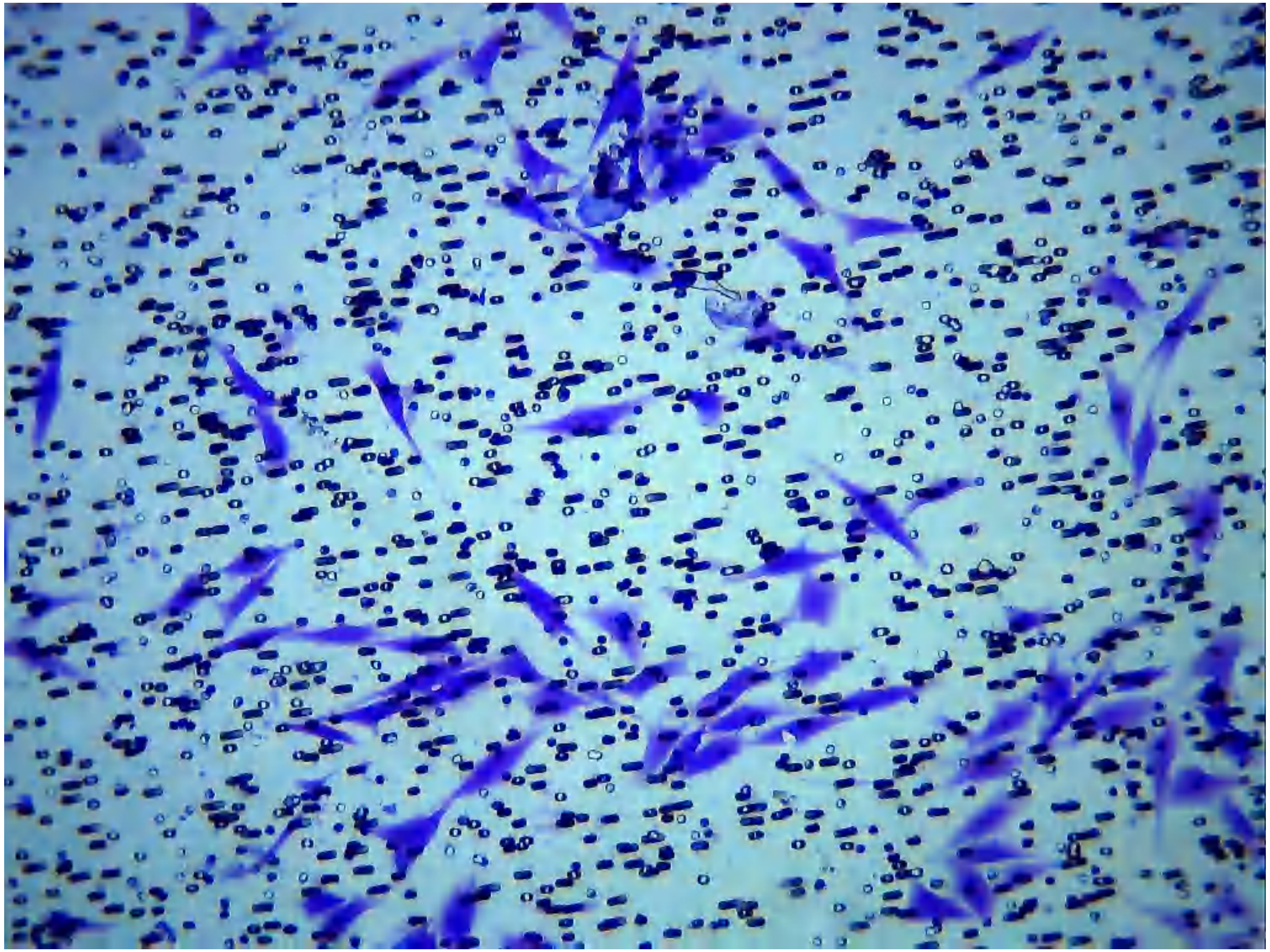

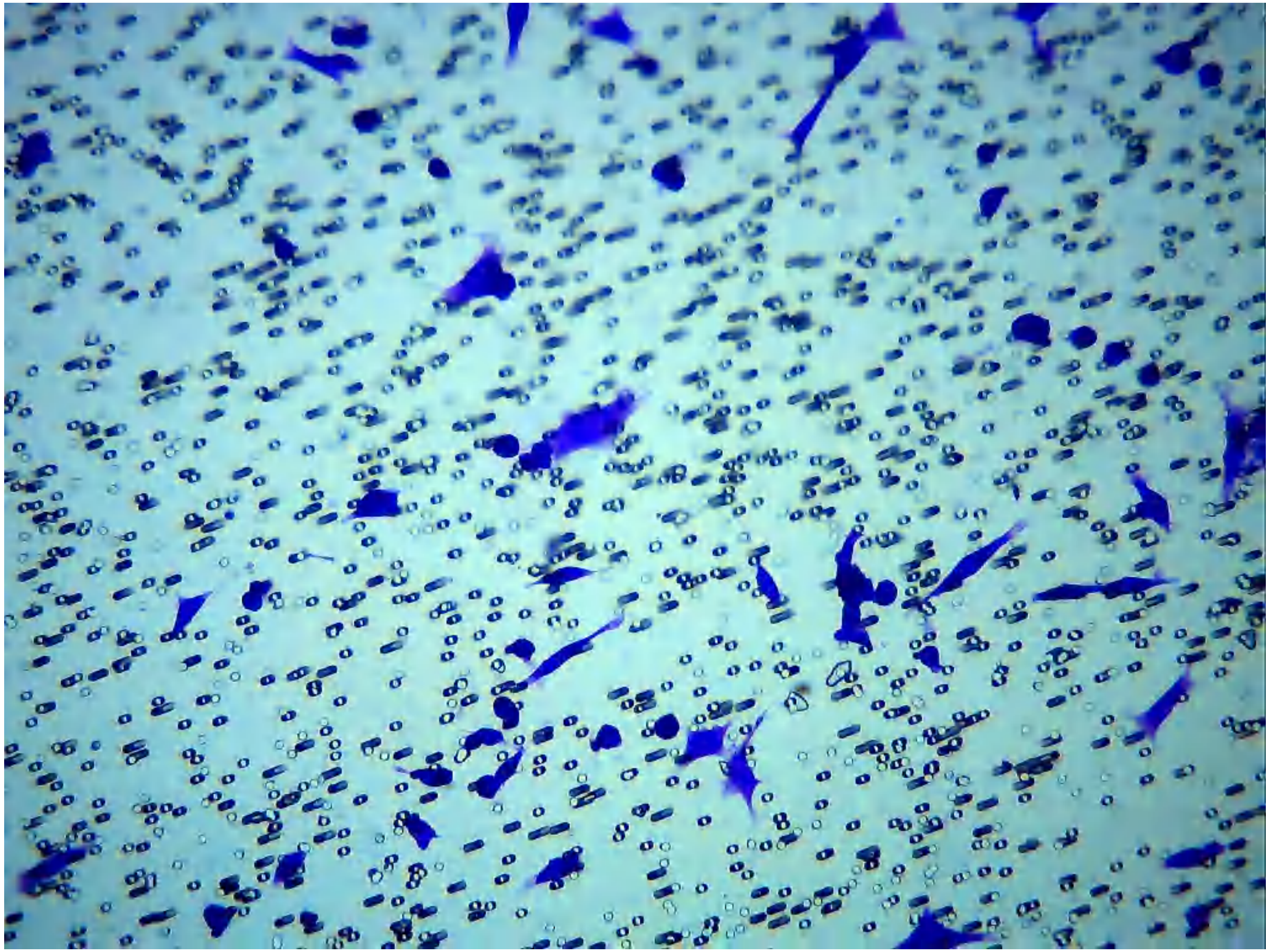

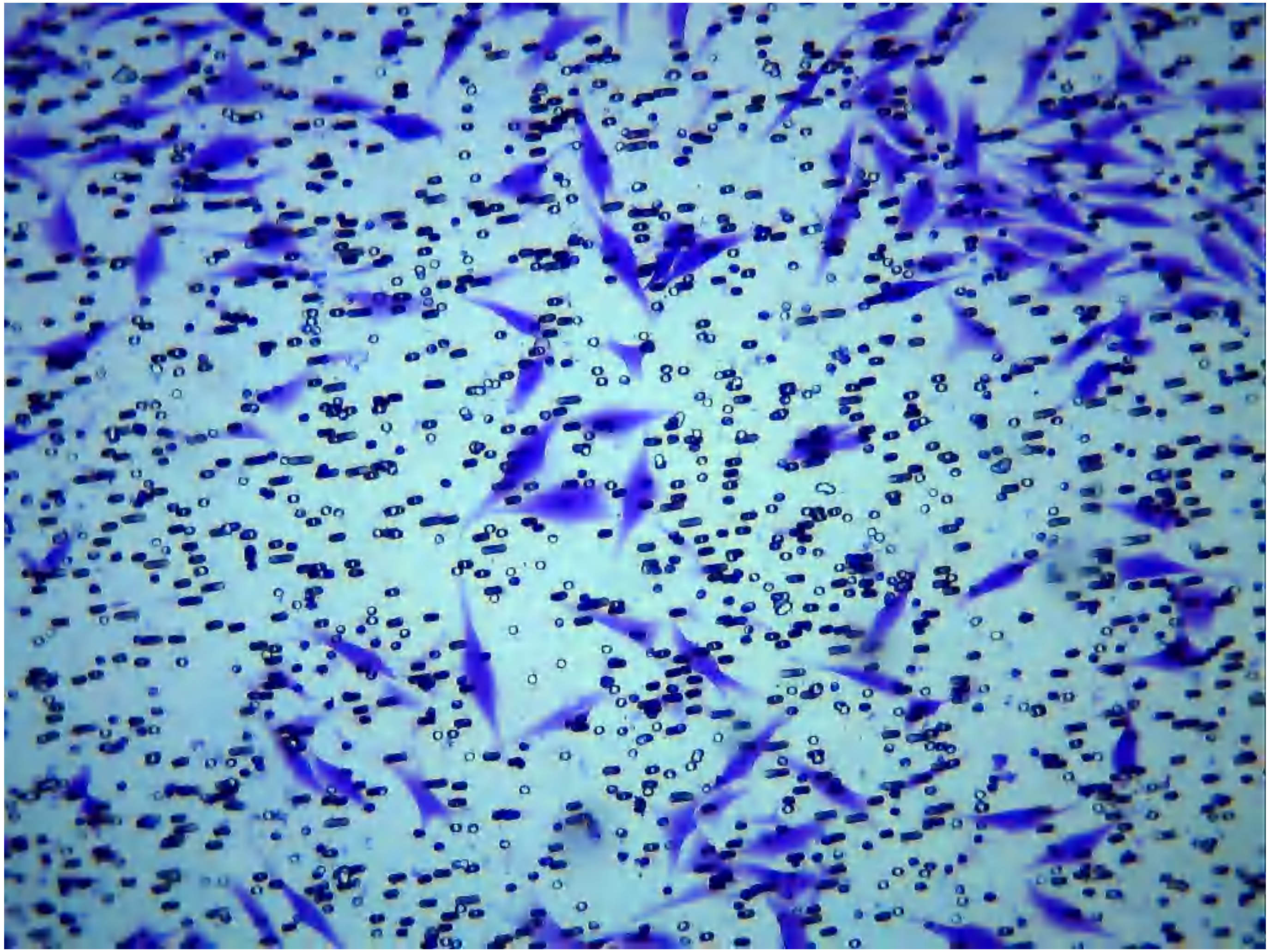

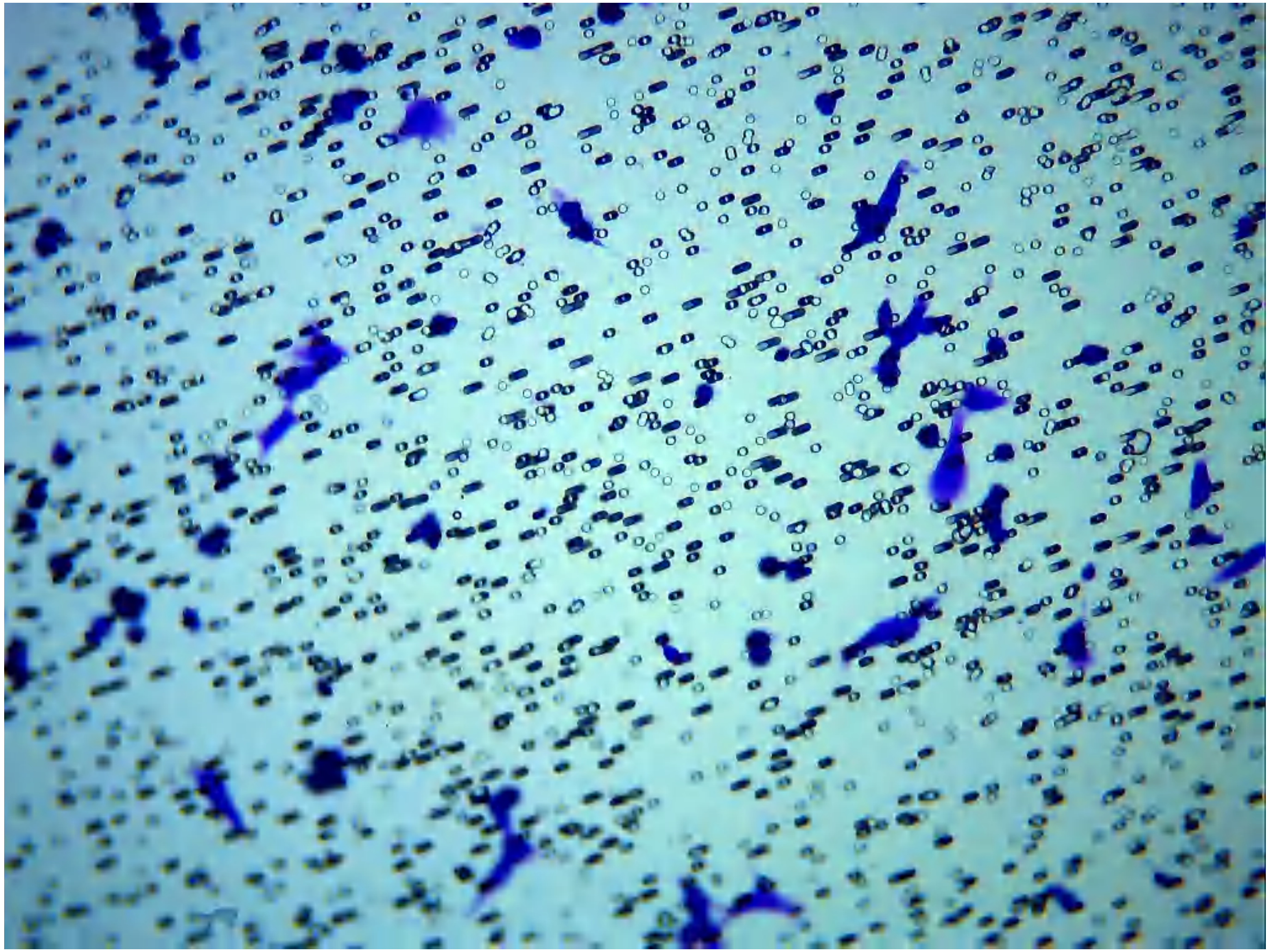

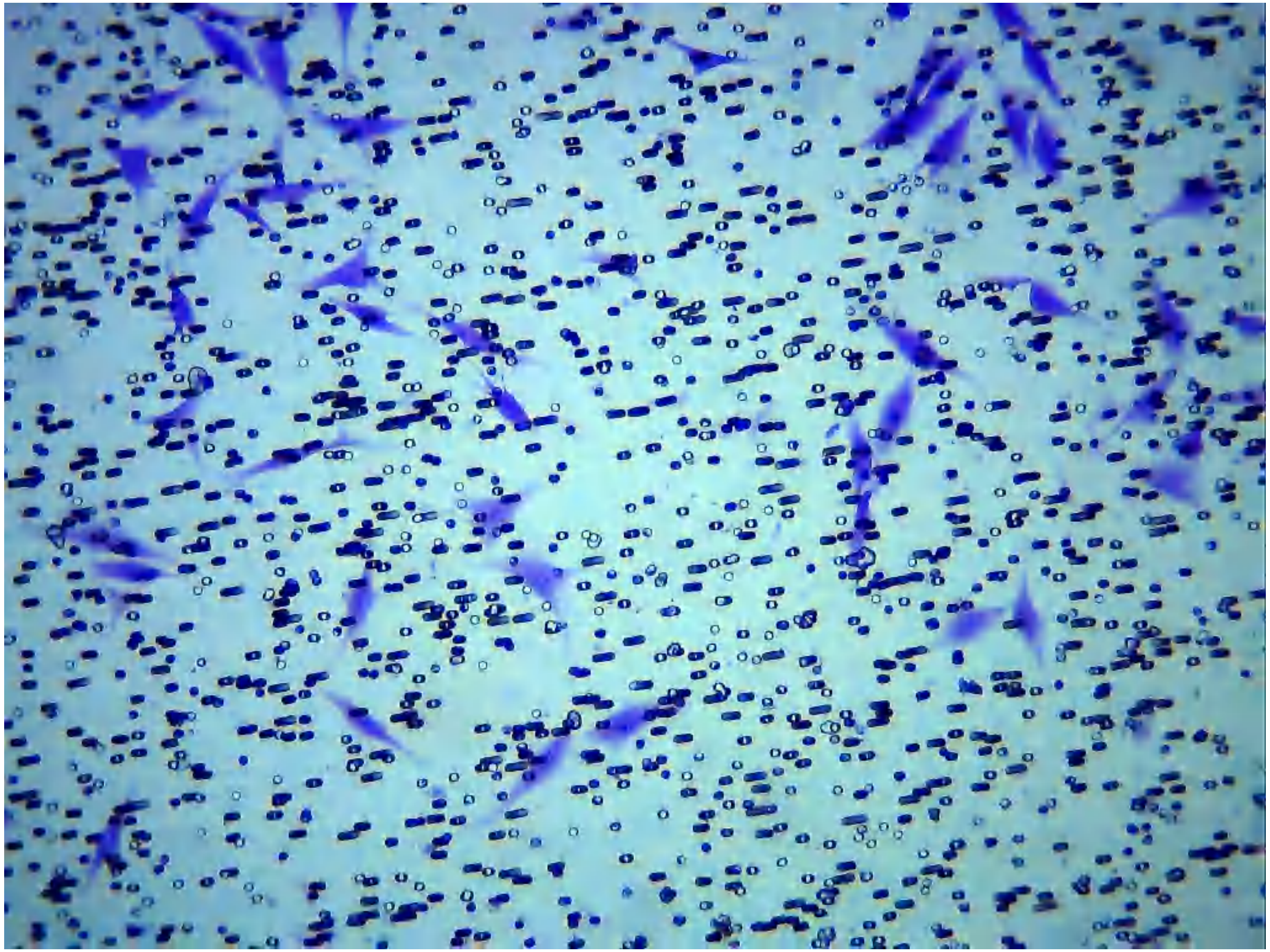

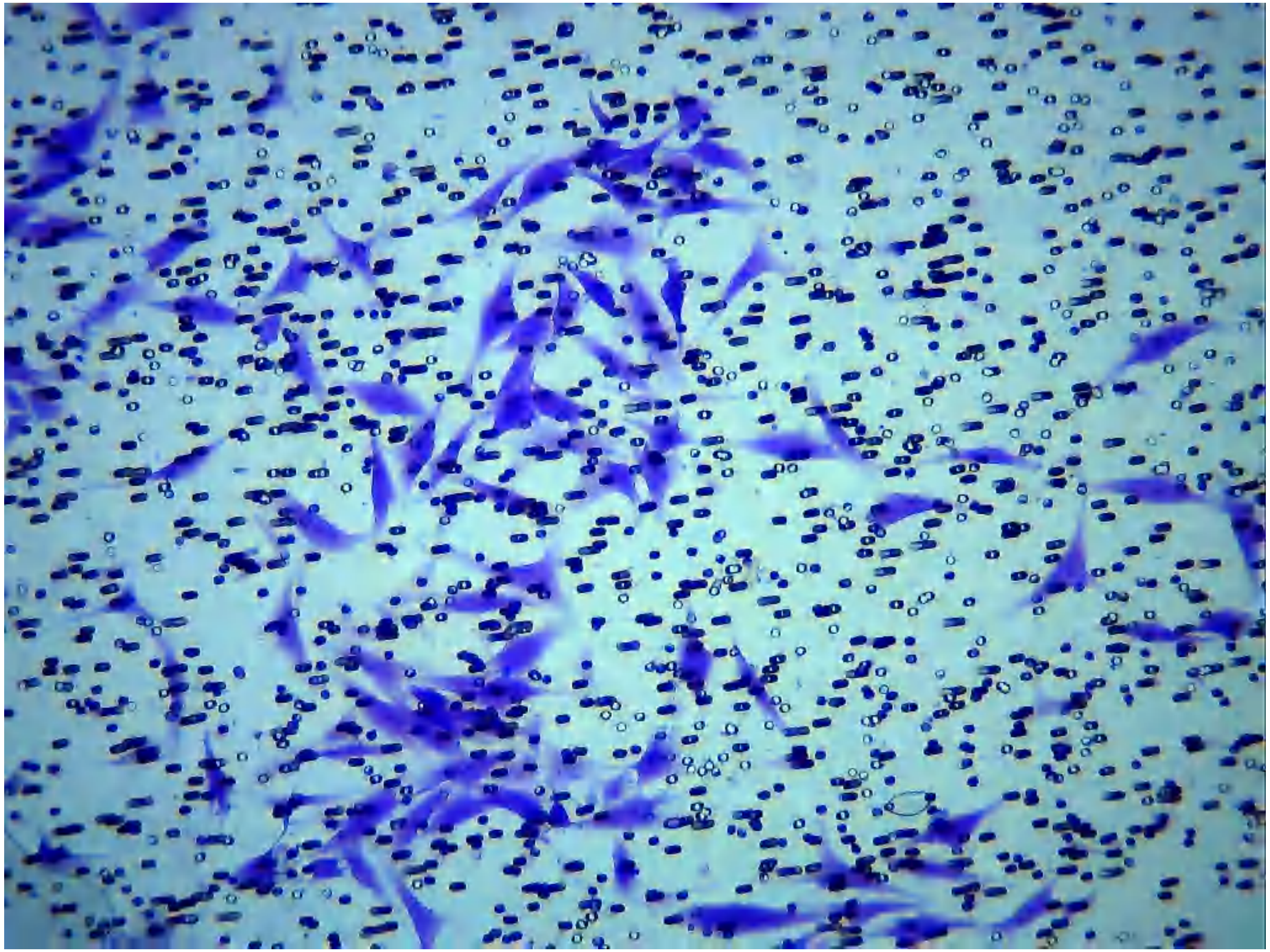

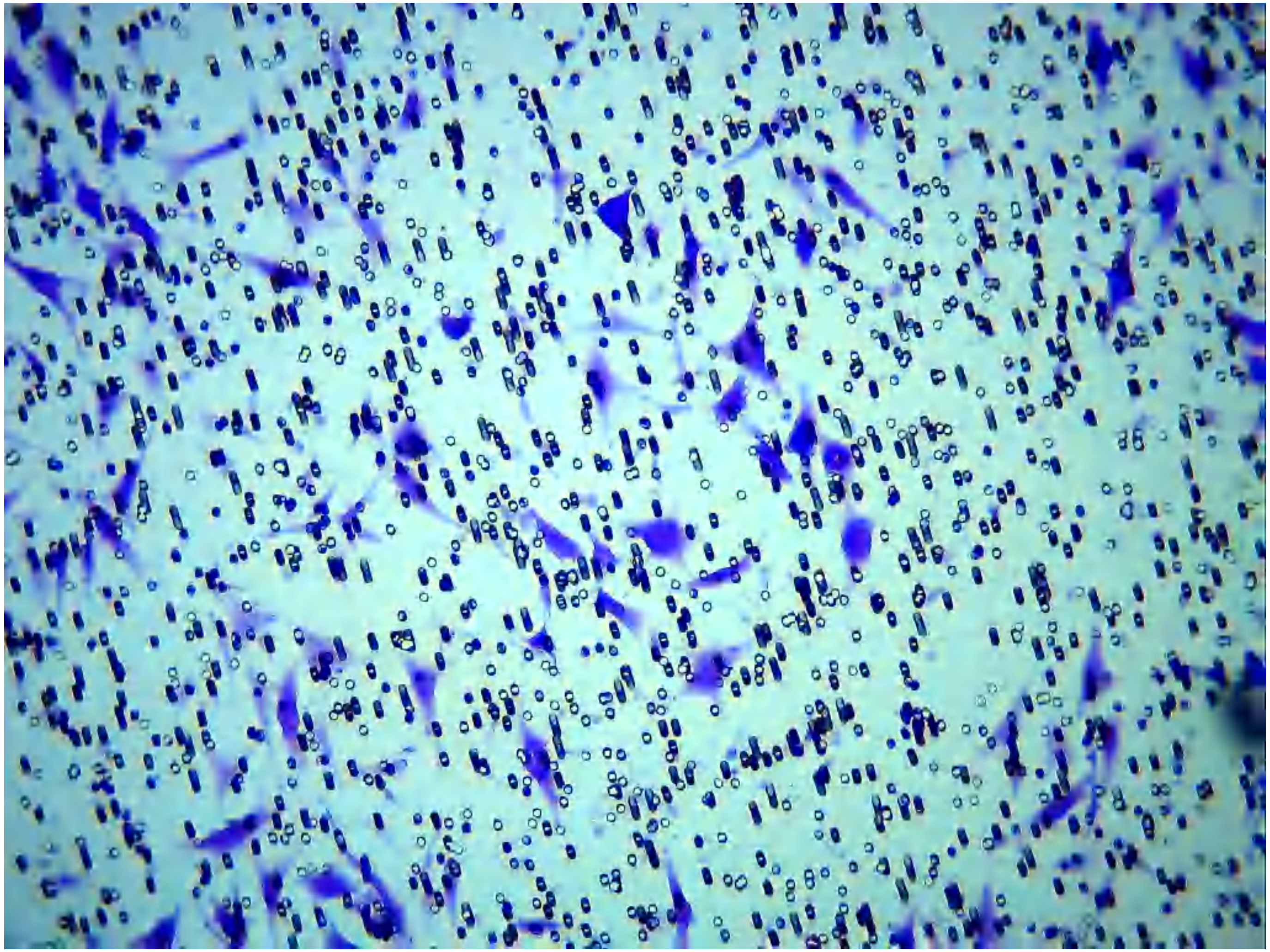

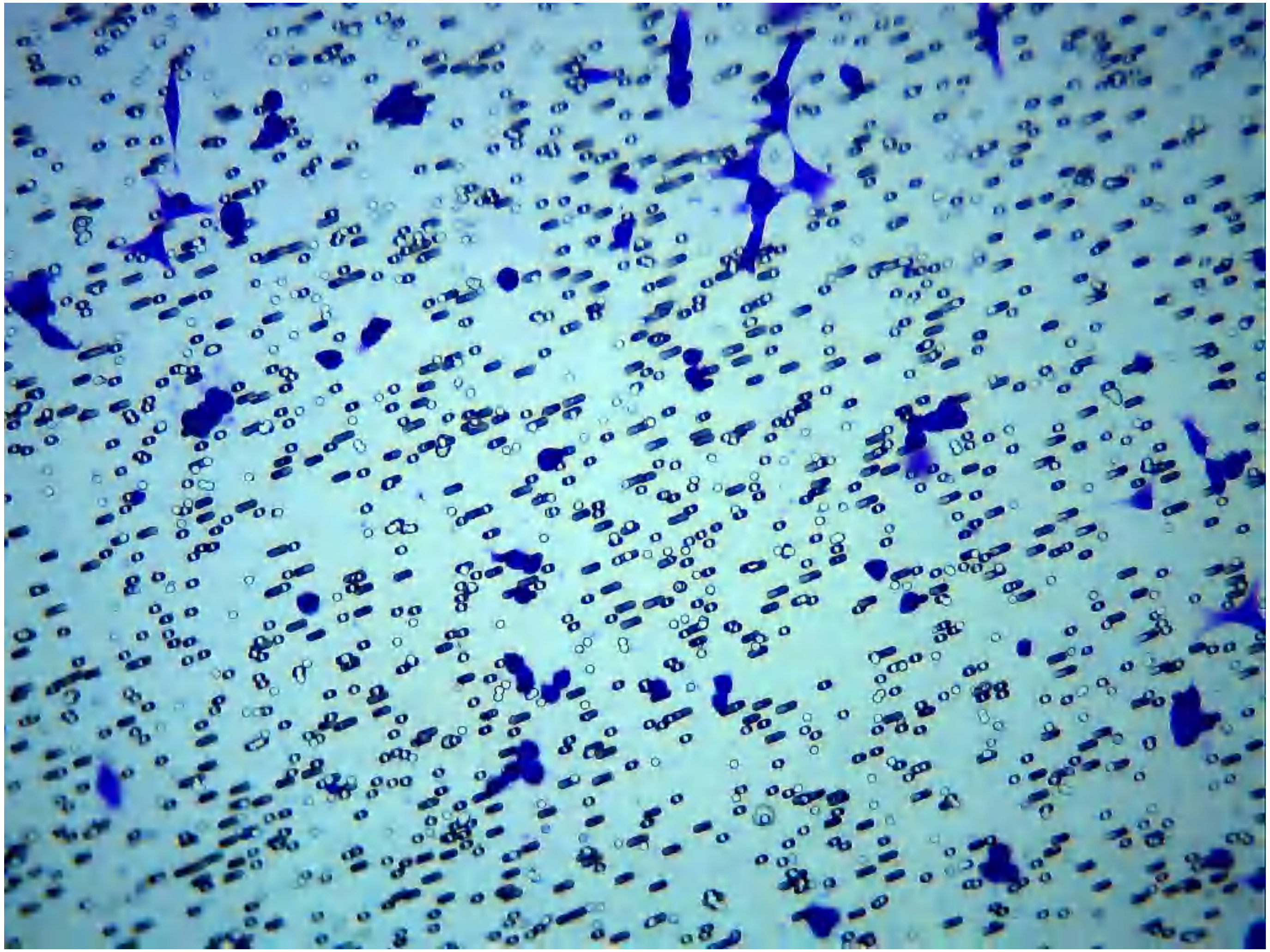

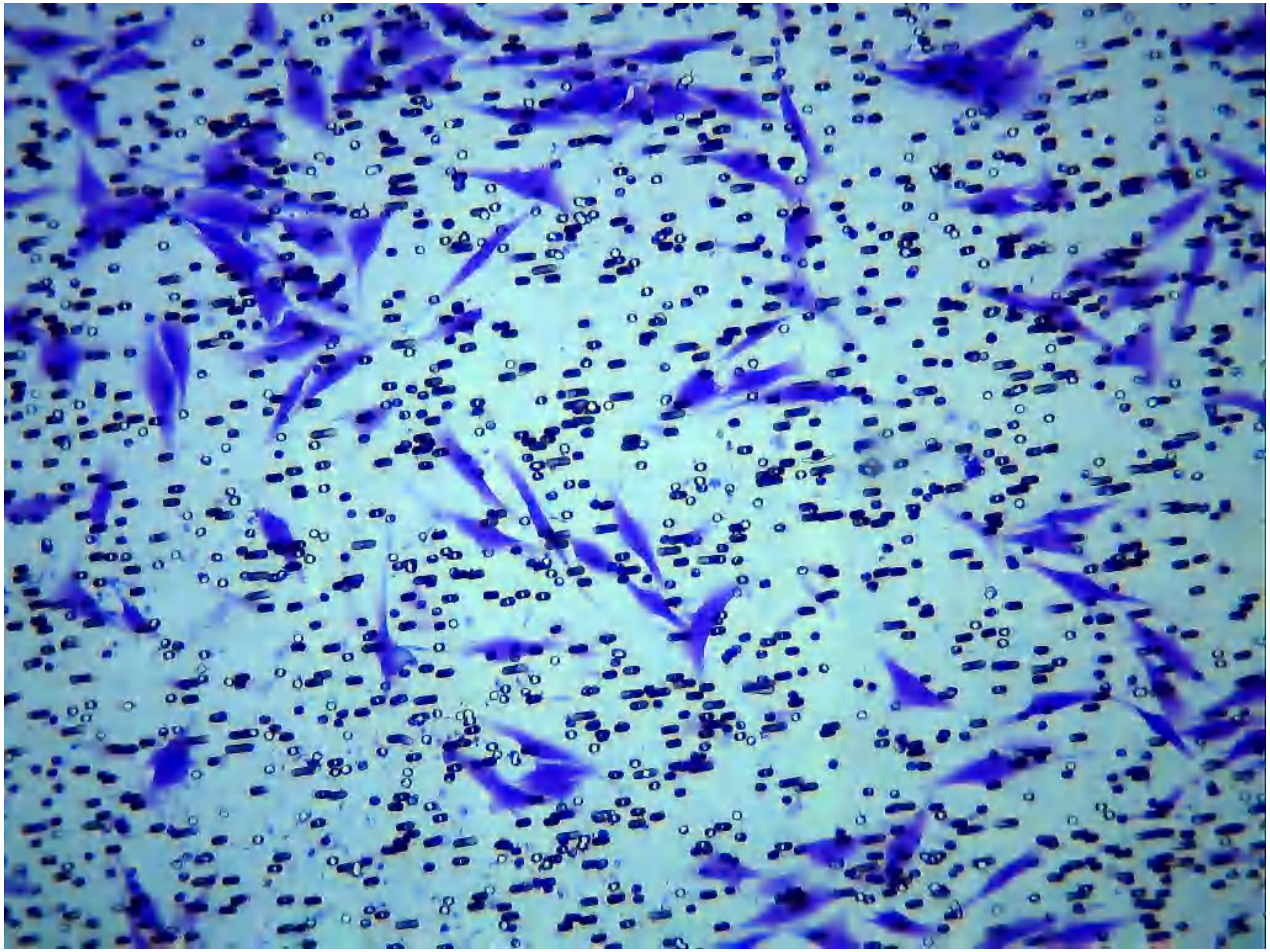

6A

	control			XLGB		
0h	0.267	0.257	0.272	0.25	0.257	0.228
24h	0.506	0.528	0.582	0.49	0.588	0.494
48h	0.906	0.865	0.79	0.846	0.883	0.781
72h	1.343	1.278	1.254	1.318	1.125	1.259

	control			XLGB		
0h	0.287	0.292	0.284	0.294	0.286	0.288
24h	0.597	0.582	0.593	0.571	0.563	0.559
48h	0.953	0.997	0.961	0.879	0.892	0.921
72h	1.302	1.378	1.384	1.323	1.276	1.299

	control			XLGB		
0h	0.312	0.326	0.321	0.322	0.334	0.327
24h	0.622	0.614	0.598	0.587	0.584	0.596
48h	0.933	0.946	0.927	0.899	0.914	0.879
72h	1.365	1.347	1.362	1.267	1.283	1.294

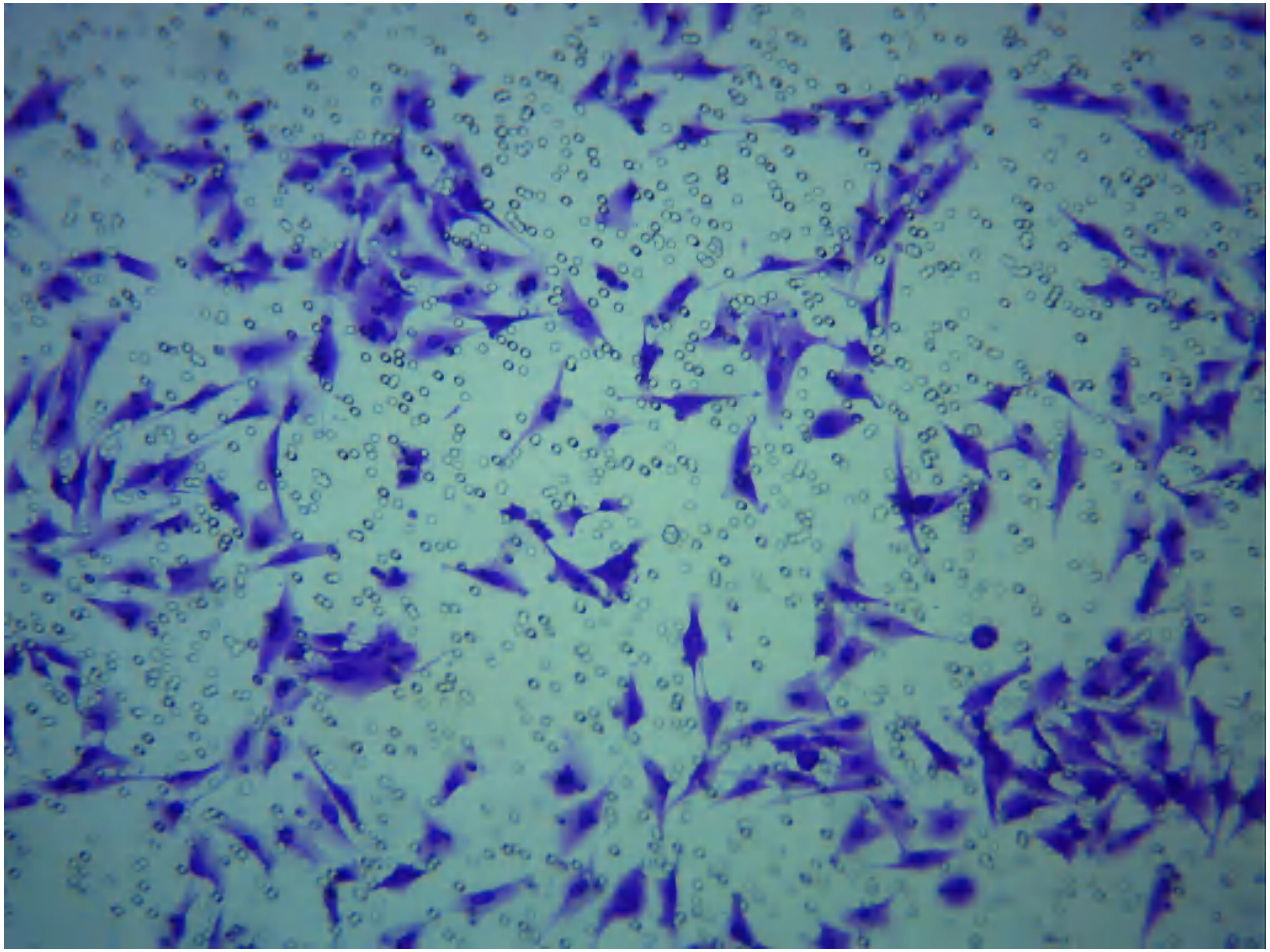

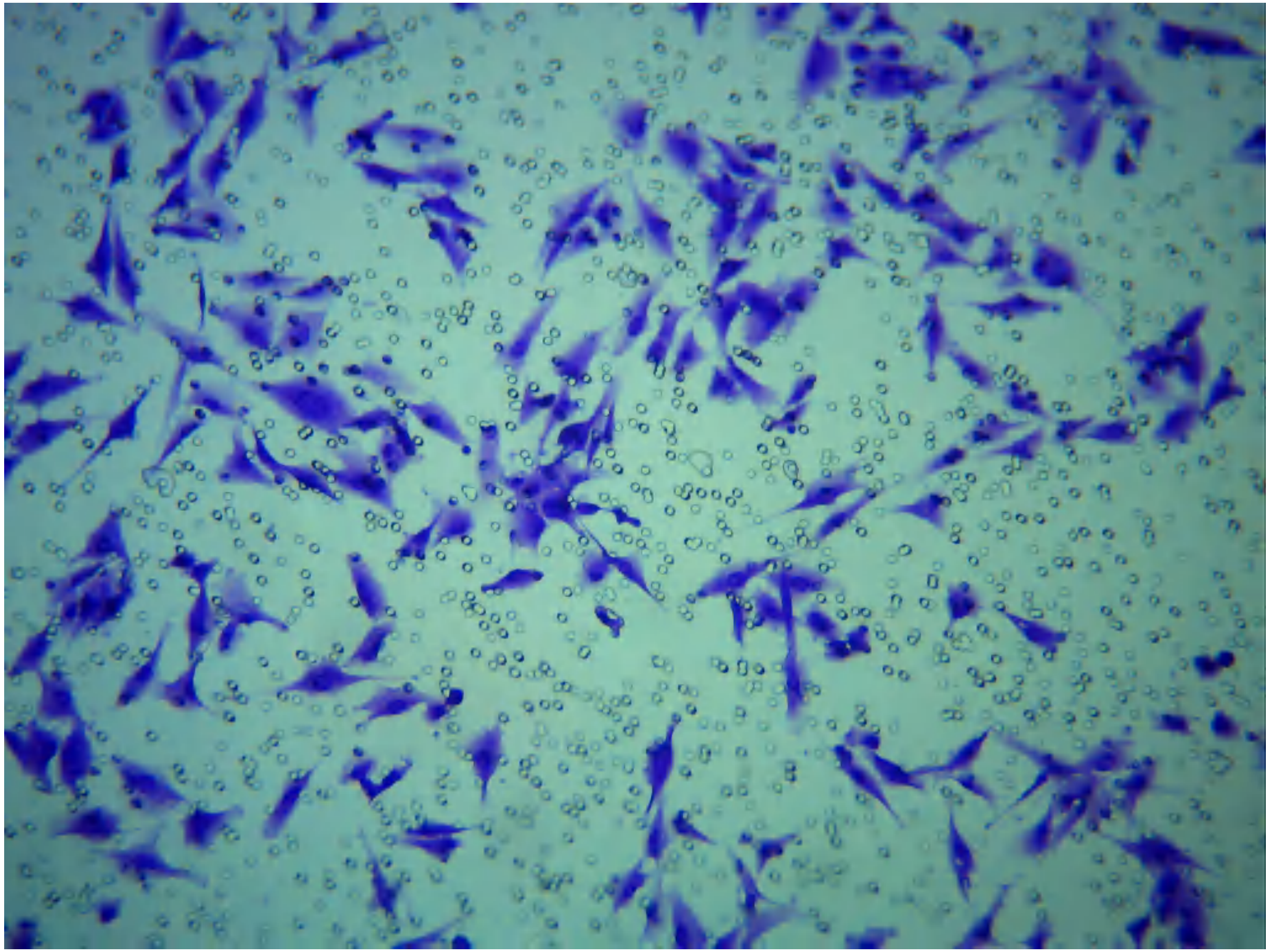

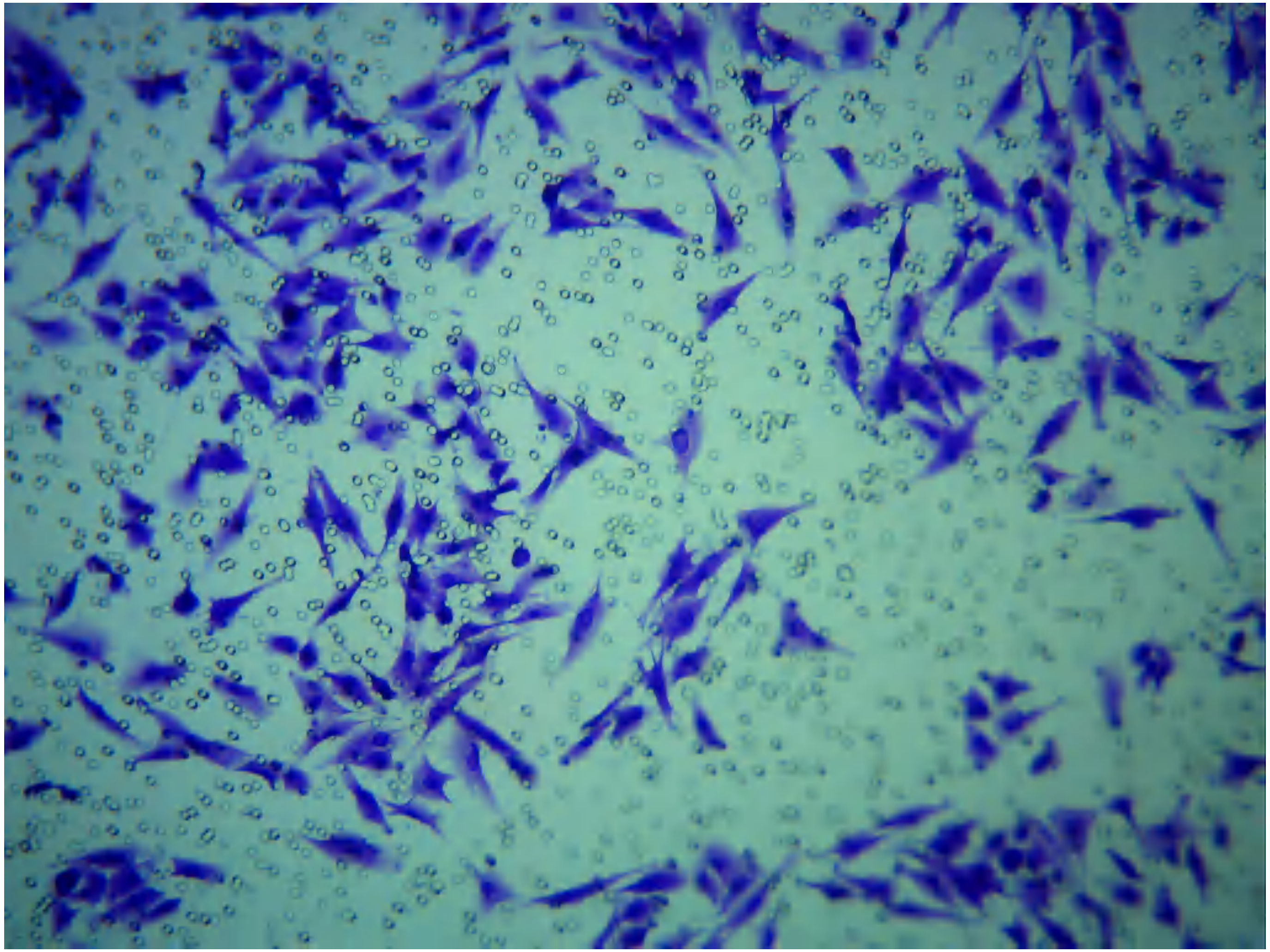

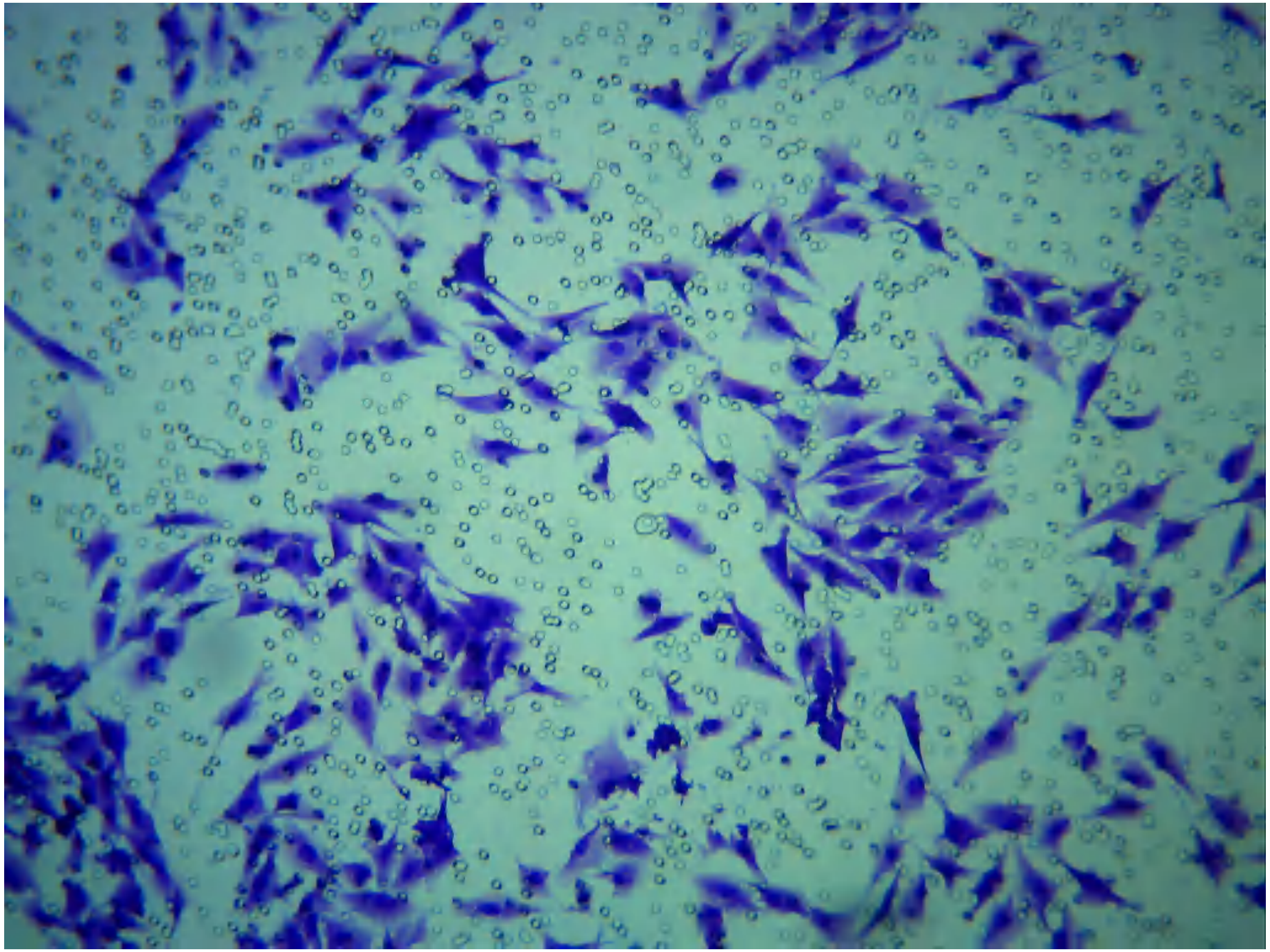

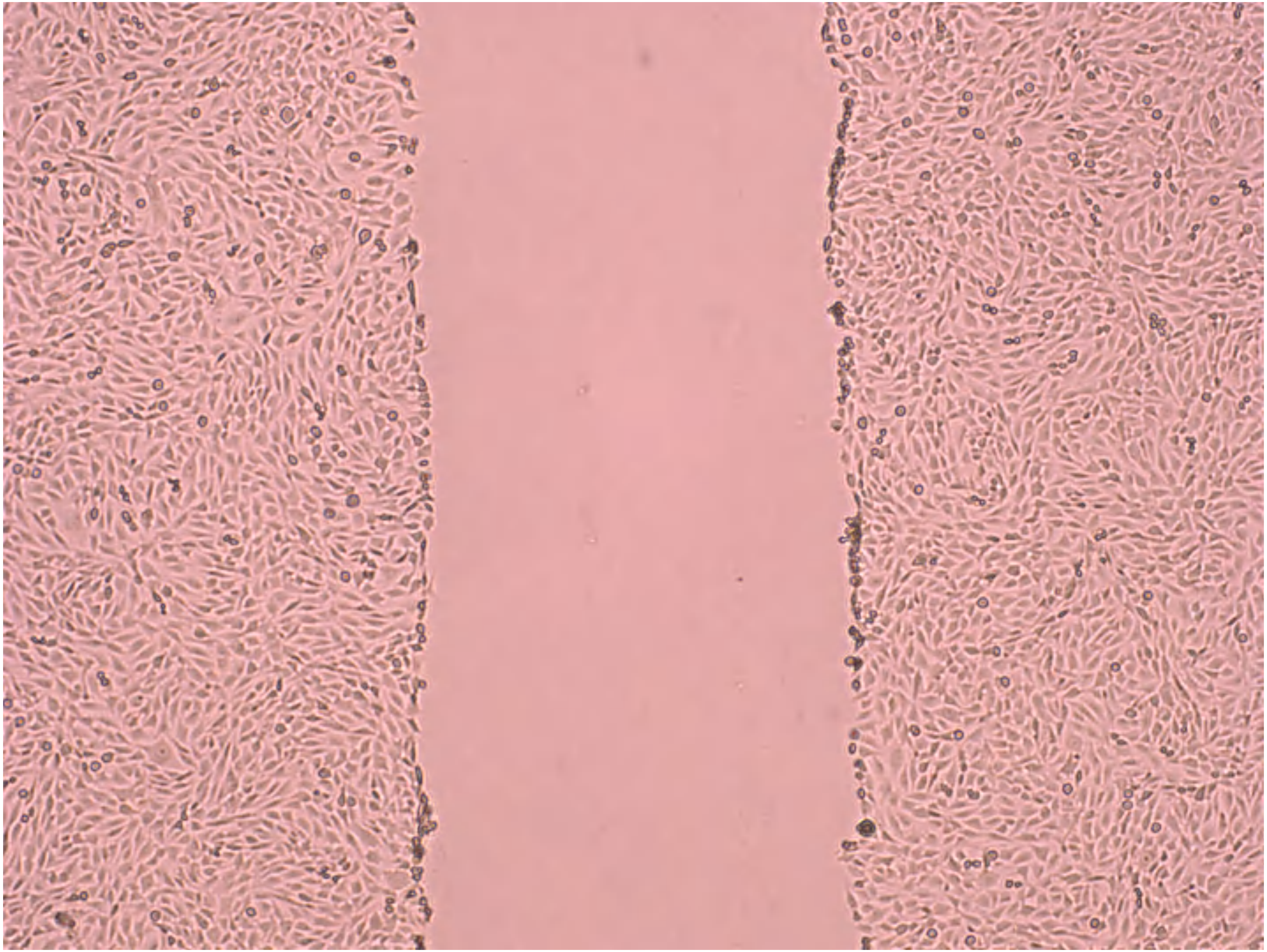

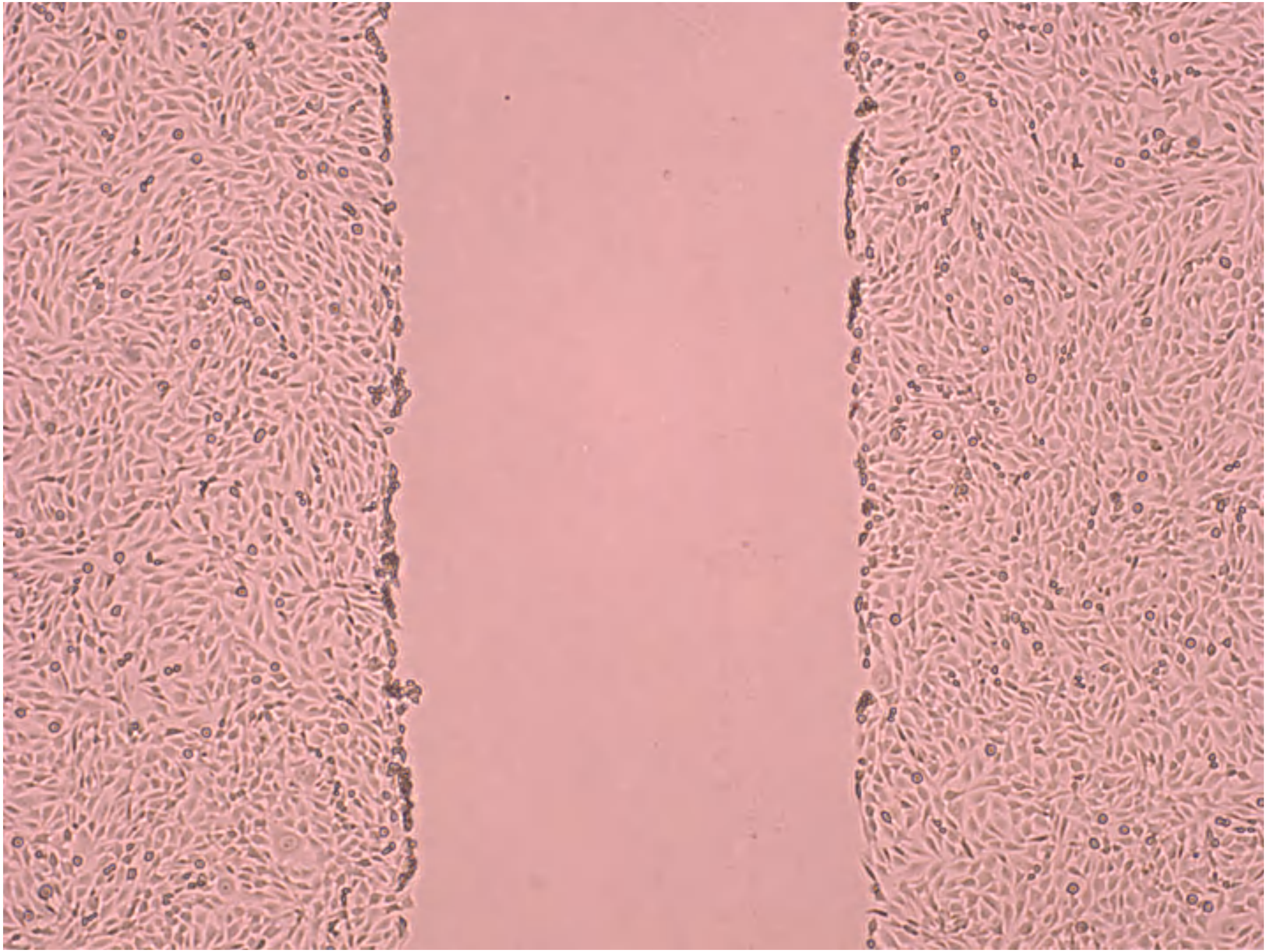

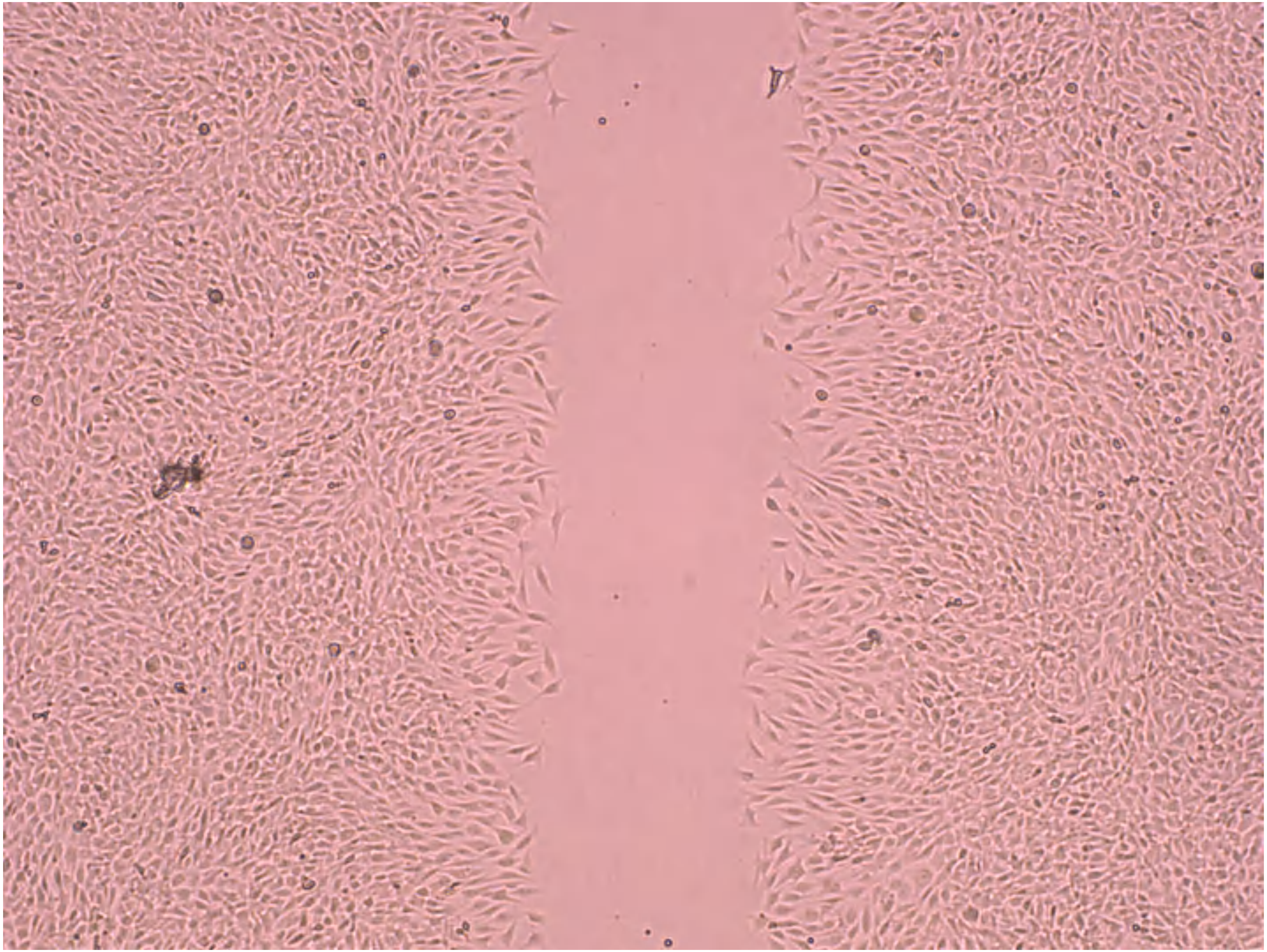

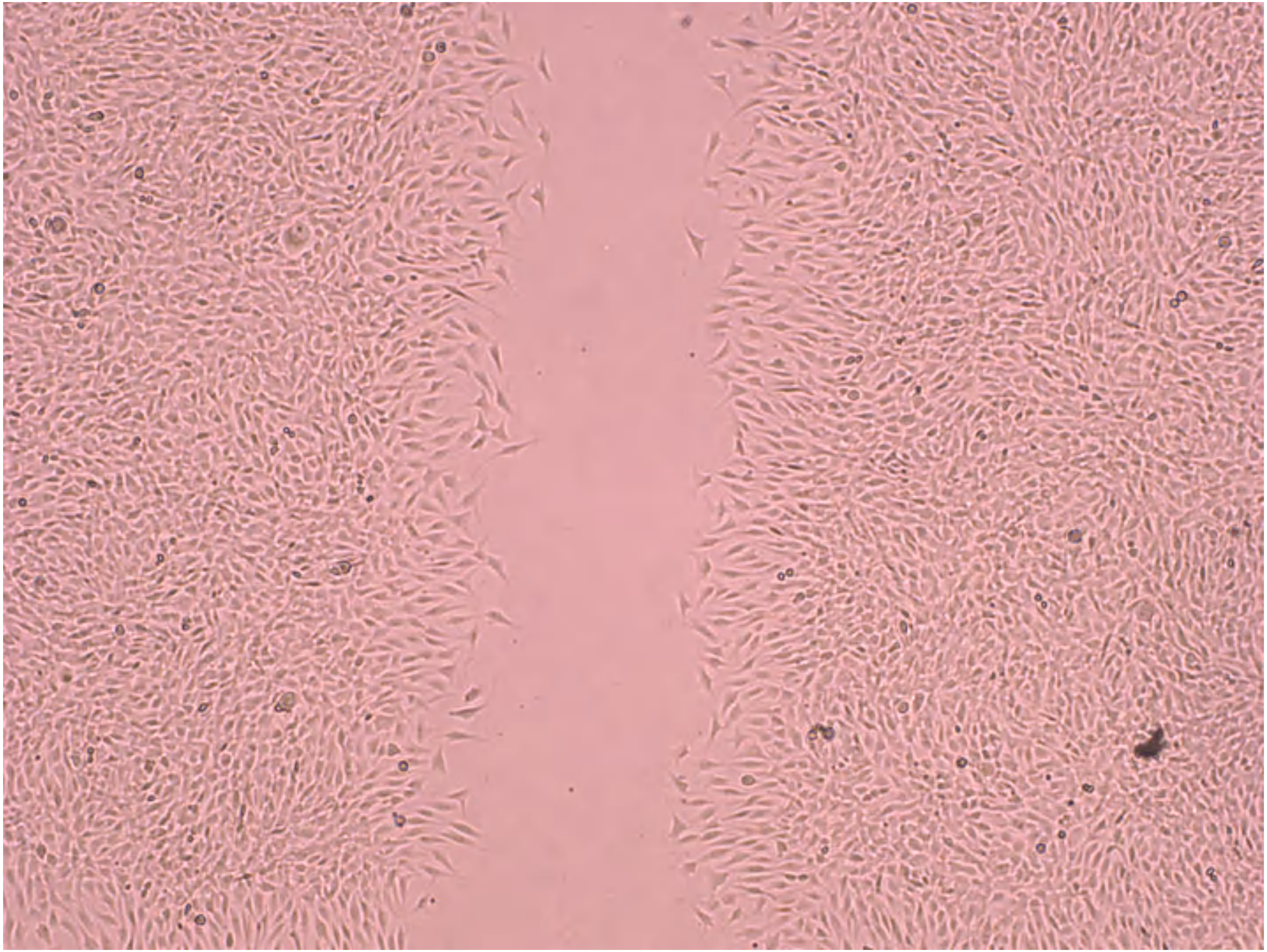

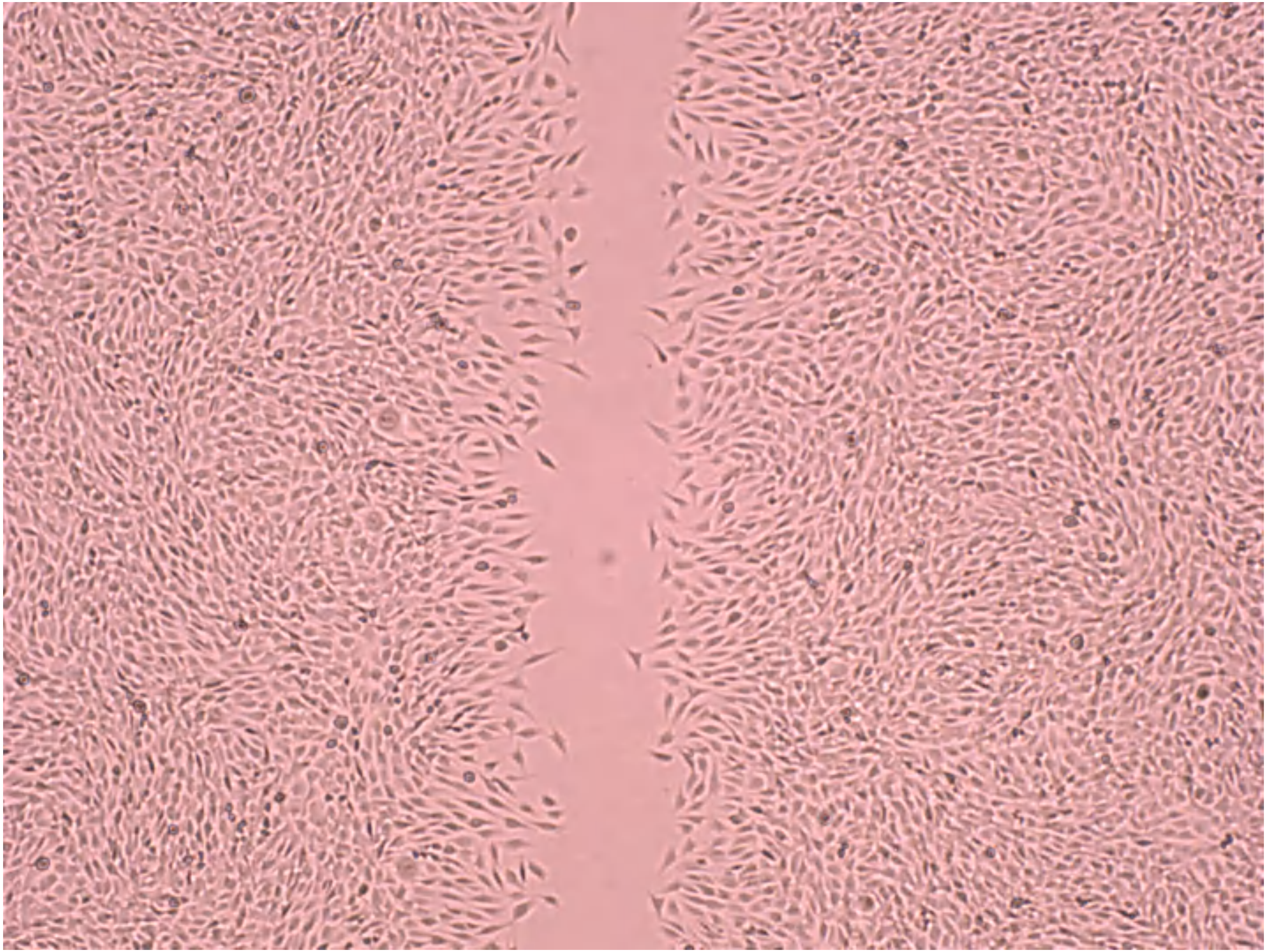

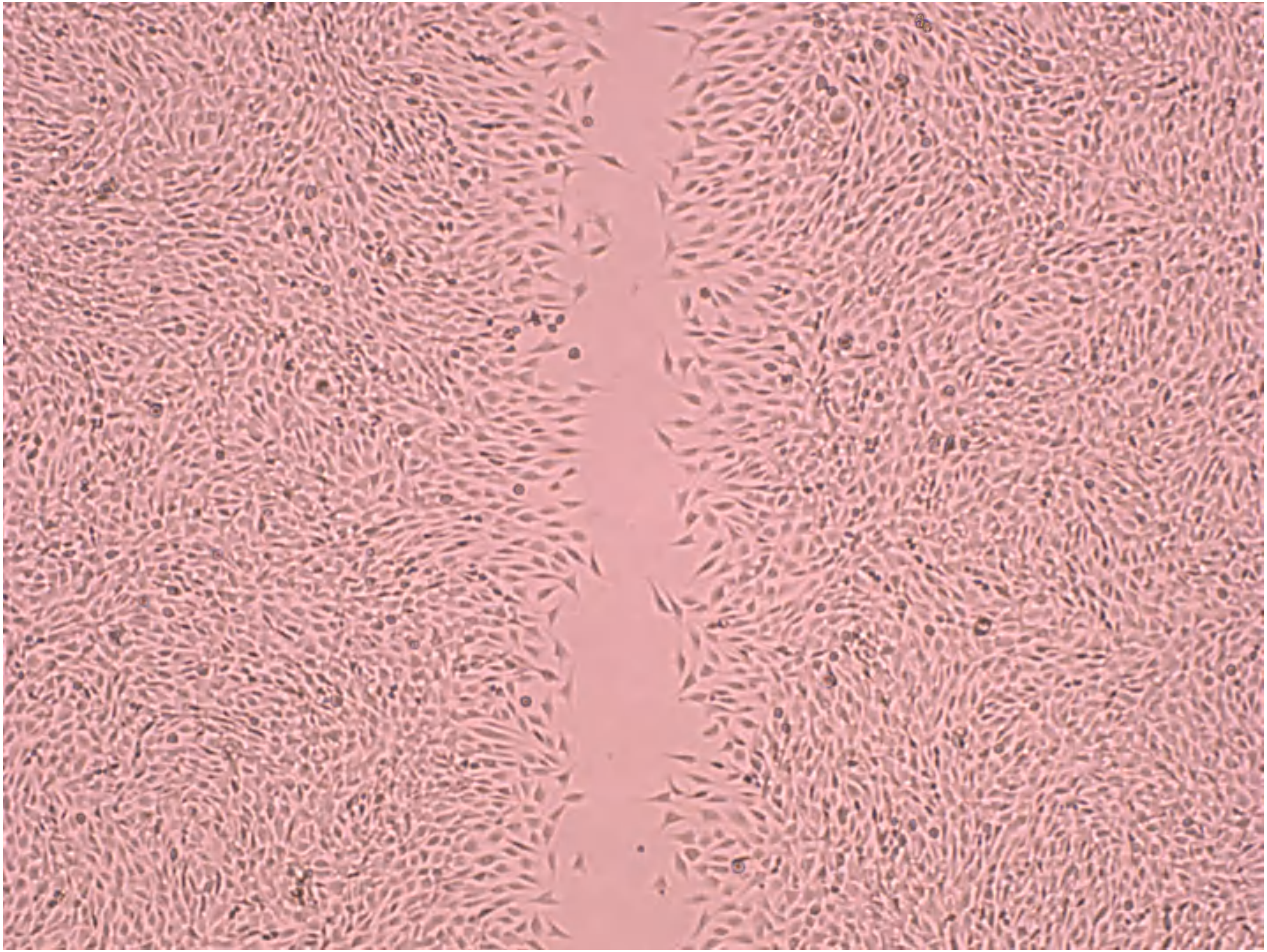

MG63 仙灵

1.NC 2.HG 3.HG+仙灵

凋亡蛋白

Bcl-2

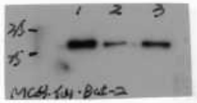

Bax

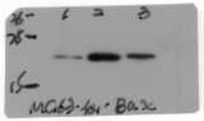

Active Caspase3 p17

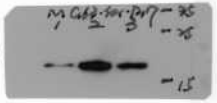

GAPDH

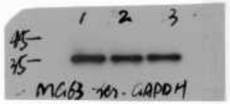

周期蛋白

CDK4

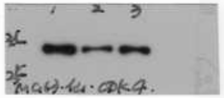

CDK6

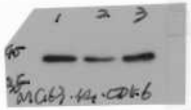

CyclinD1

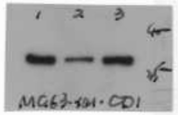

MKAP1

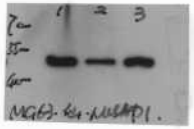

GAPDH

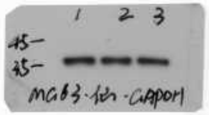

PI3K/AKT通路

AKT

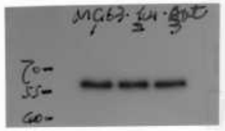

p-AKT

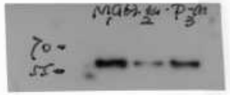

P70

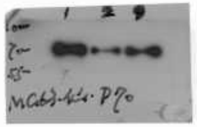

GAPDH

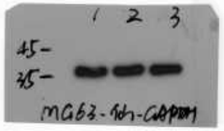

MG63-仙灵2

凋亡蛋白

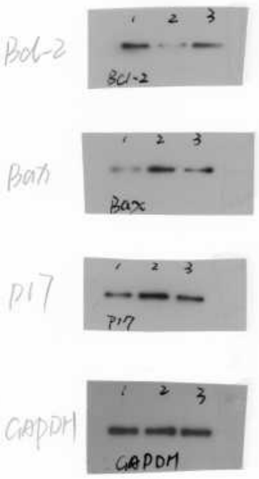

周期蛋白

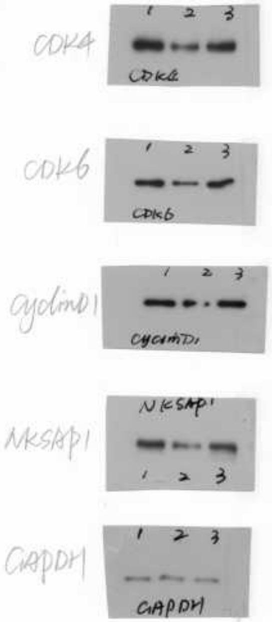

PI3K/AKT

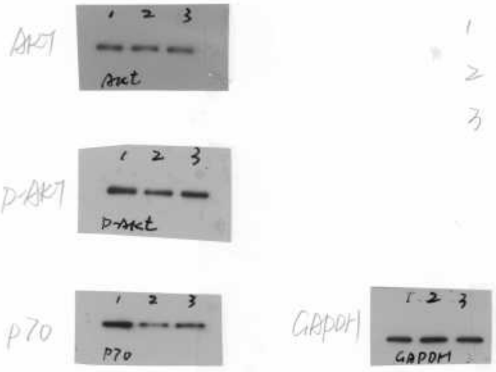

1 NC
2 HG
3 HG+仙灵骨葆

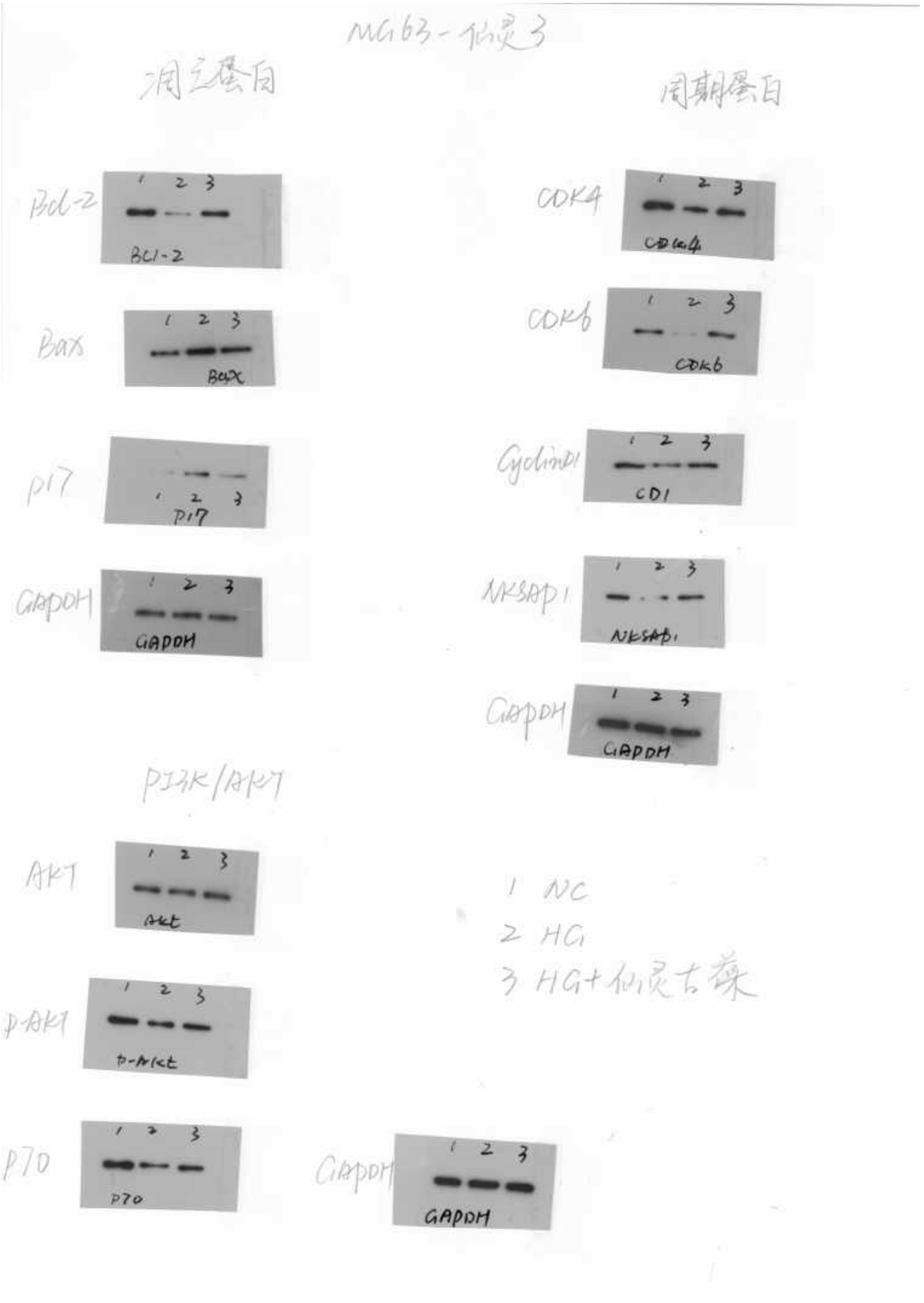

MG63-仙灵3
凋亡蛋白
周期蛋白
Bcl-2
Bax
P17
GAPDH
CDK4
CDK6
Cyclin D1
NKSAP1
GAPDH
PI3K/AKT
AKT
p-AKT
P70
GAPDH
1 NC
2 HG
3 HG+仙灵古葆

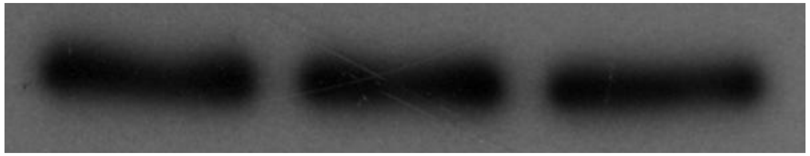

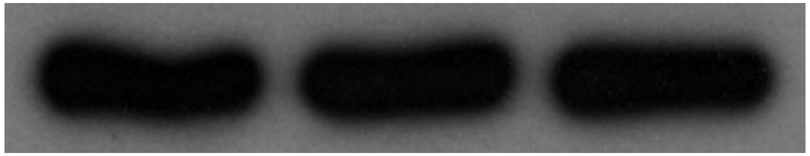

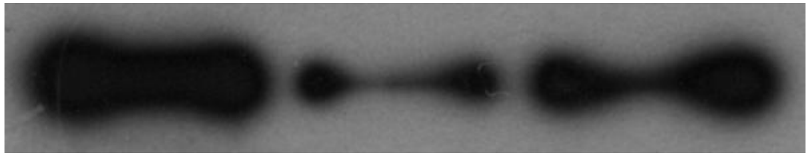

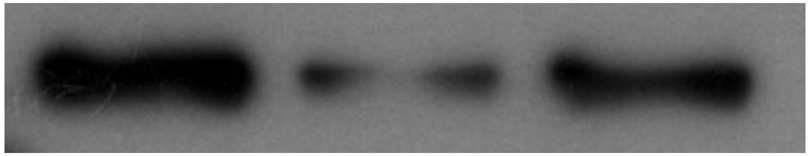

MG63 仙灵

1.NC 2.HG 3.HG+仙灵

凋亡蛋白

Bcl-2
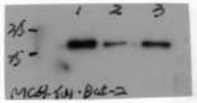

Bax
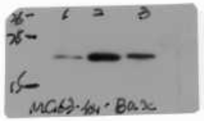

Active Caspase3 p17
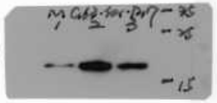

GAPDH
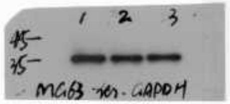

周期蛋白

CDK4
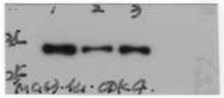

CDK6
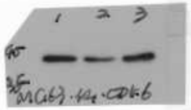

CyclinD1
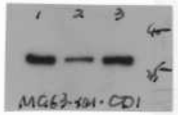

NUSAP1
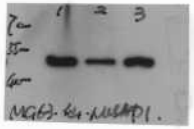

GAPDH
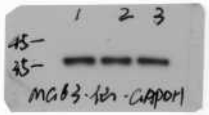

PI3K/AKT通路

AKT
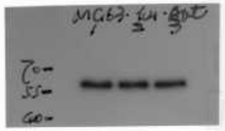

p-AKT
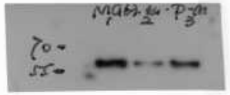

P70
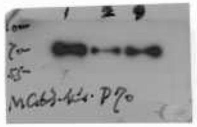

GAPDH
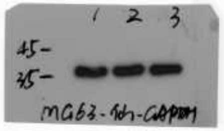

MG63-仙灵2

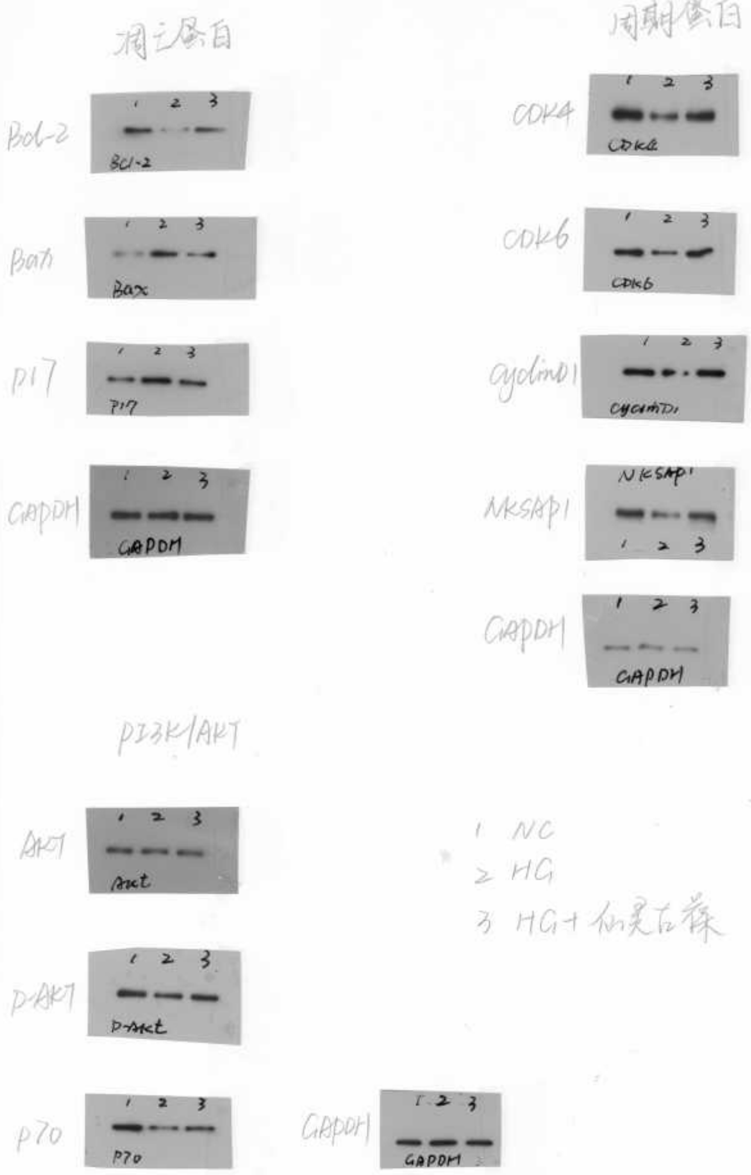

凋亡蛋白
周期蛋白
Bcl-2
1 2 3
BCl-2
Bax
1 2 3
Bax
P17
1 2 3
P17
GAPDH
1 2 3
GAPDH
CDK4
1 2 3
CDK4
CDK6
1 2 3
CDK6
cyclinD1
1 2 3
cyclinD1
NKSAP1
NKSAP1
1 2 3
GAPDH
1 2 3
GAPDH
PI3K/AKT
AKT
1 2 3
Akt
P-AKT
1 2 3
P-Akt
P70
1 2 3
P70
GAPDH
1 2 3
GAPDH
1 NC
2 HG
3 HG+仙灵[illegible]

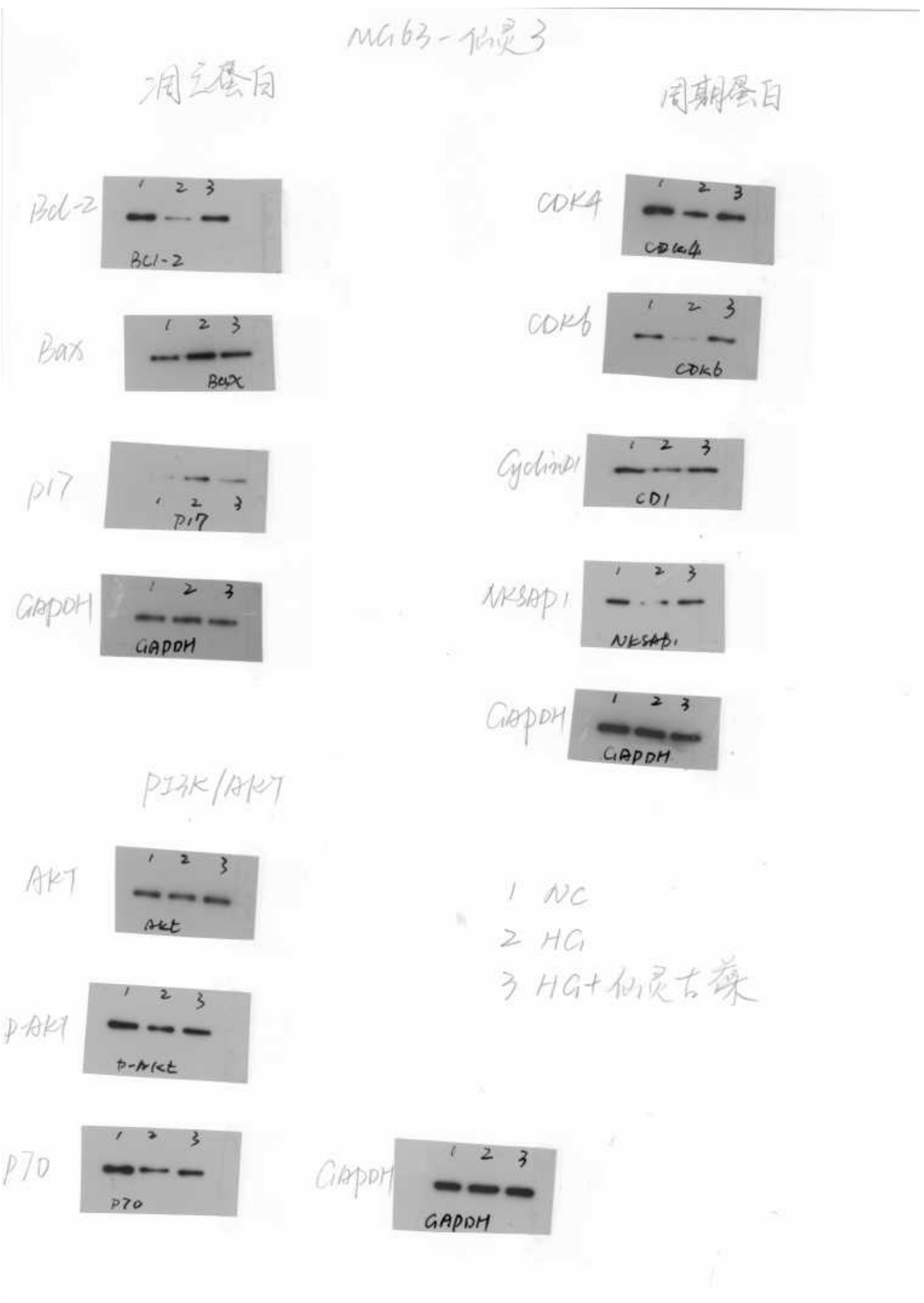

MG63-仙灵3
凋亡蛋白
周期蛋白
Bcl-2
Bax
P17
GAPDH
CDK4
CDK6
CyclinD1
CD1
NKSAP1
PI3K/AKT
AKT
p-AKT
P70
1 NC
2 HG
3 HG+仙灵古葆

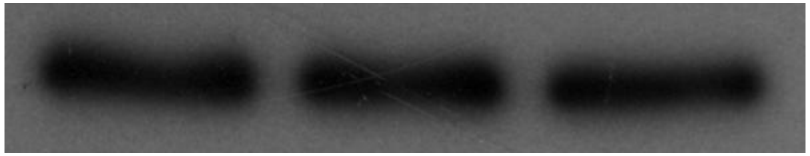

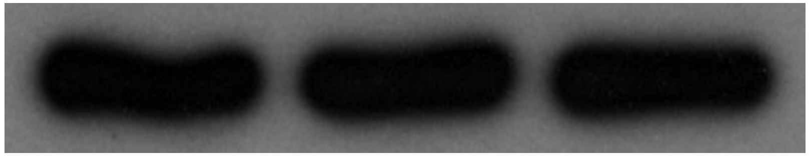

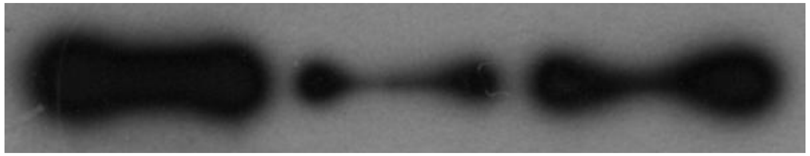

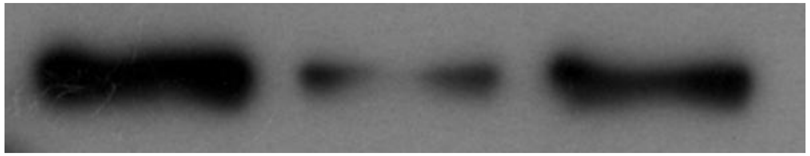

MG63 仙灵

1.NC 2.HG 3.HG+仙灵

凋亡蛋白

Bcl-2

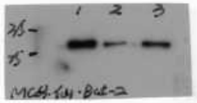

Bax

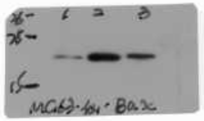

Active Caspase3 p17

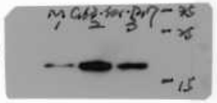

GAPDH

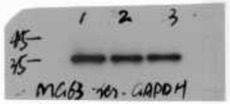

周期蛋白

CDK4

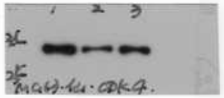

CDK6

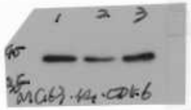

CyclinD1

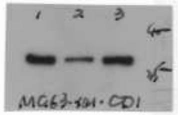

MKAP1

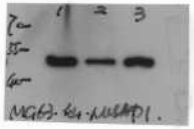

GAPDH

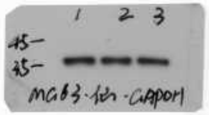

PI3K/AKT通路

AKT

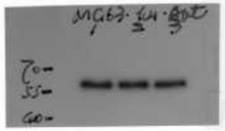

p-AKT

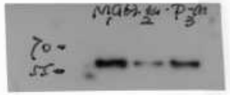

P70

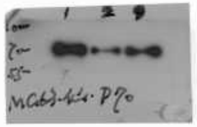

GAPDH

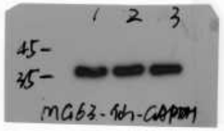

MG63-仙灵2

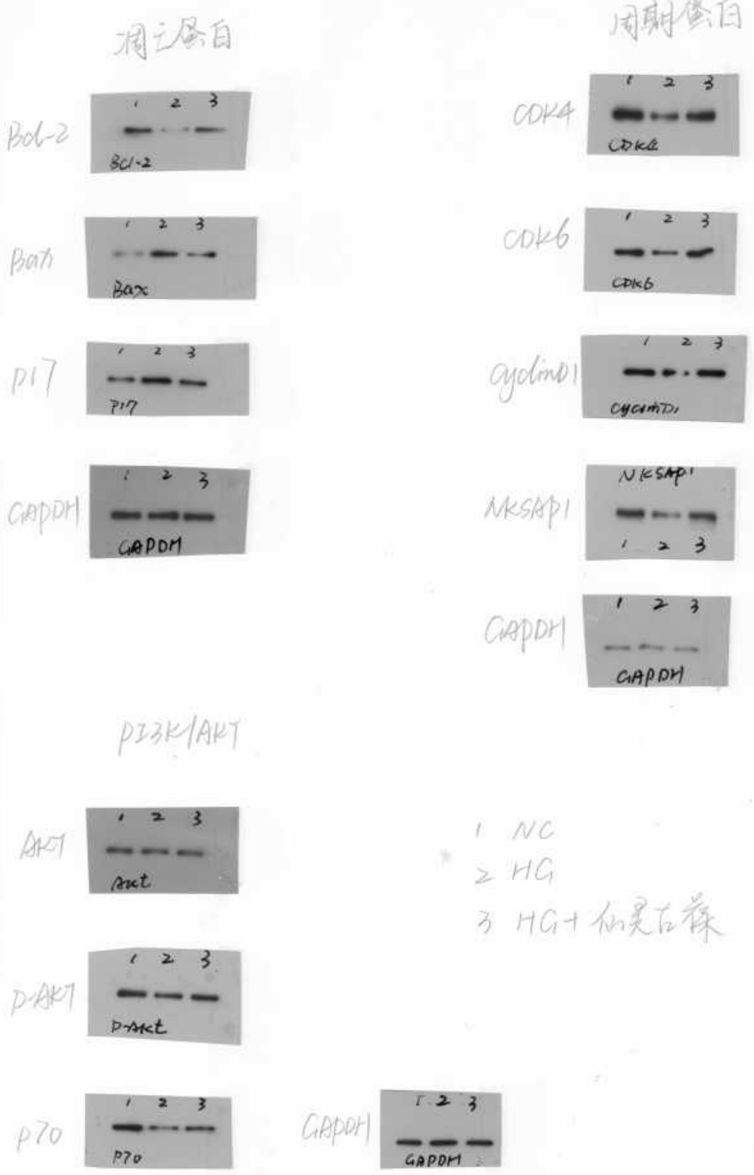
凋亡蛋白
周期蛋白
Bcl-2
1 2 3
Bcl-2
CDK4
1 2 3
CDK4
Bax
1 2 3
Bax
CDK6
1 2 3
CDK6
P17
1 2 3
P17
cyclinD1
1 2 3
cyclinD1
GAPDH
1 2 3
GAPDH
NKSAP1
NKSAP1
1 2 3
GAPDH
1 2 3
GAPDH
PI3K/AKT
AKT
1 2 3
Akt
p-AKT
1 2 3
p-Akt
P70
1 2 3
P70
GAPDH
1 2 3
GAPDH
1 NC
2 HG
3 HG+仙灵骨葆

MG63-仙灵3

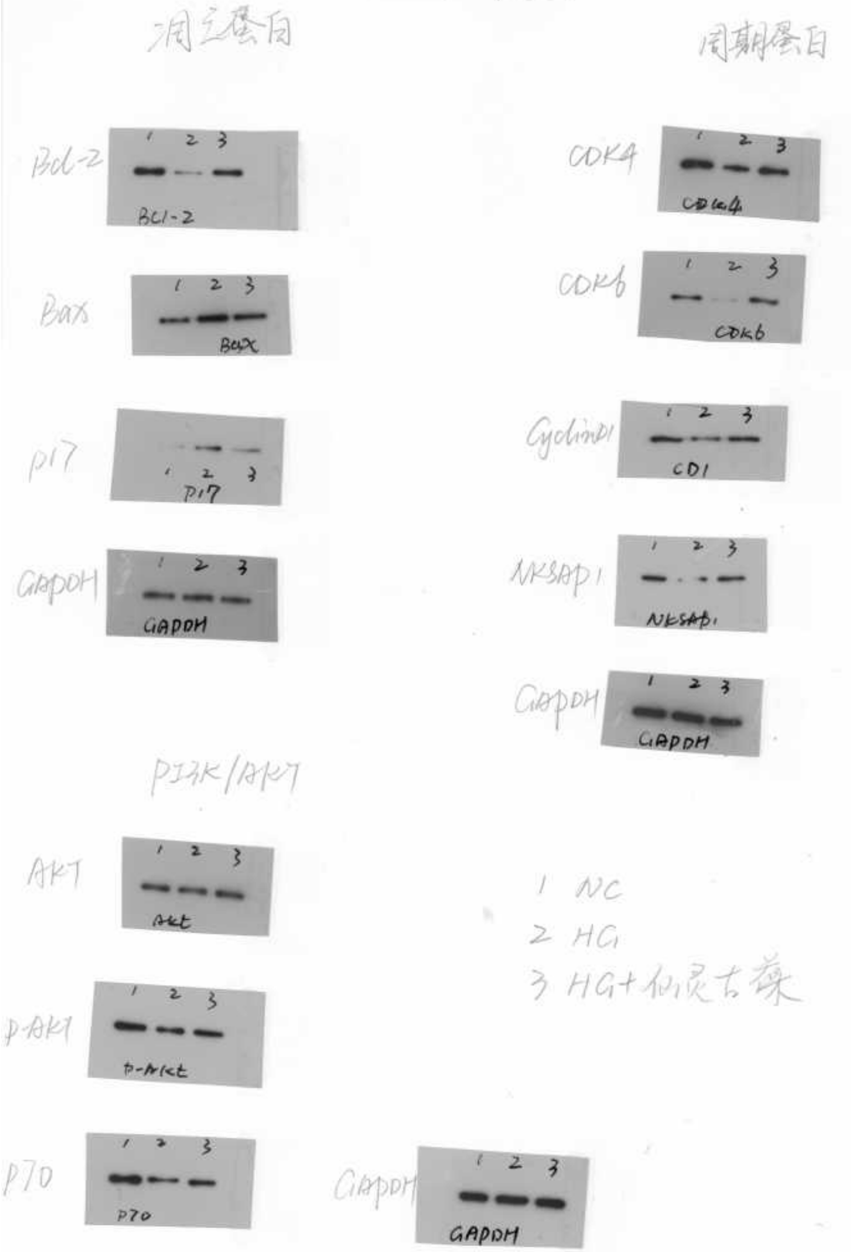

1 NC
2 HG
3 HG+仙灵古葆

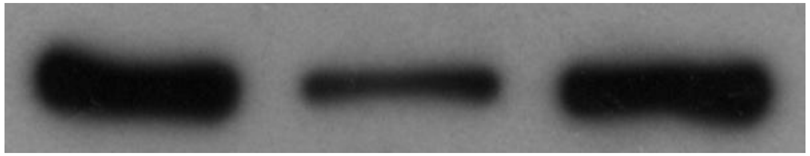

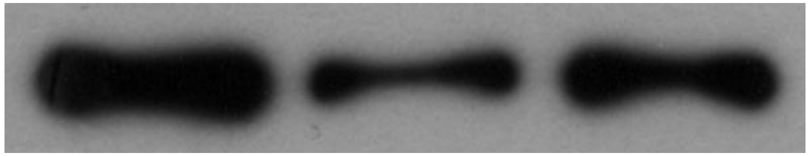

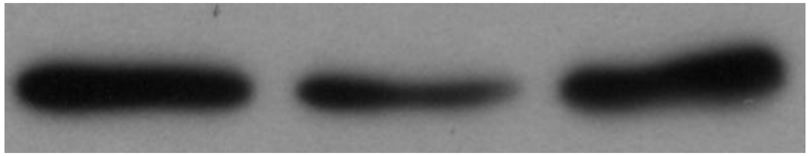

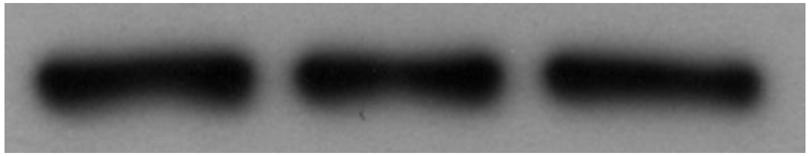

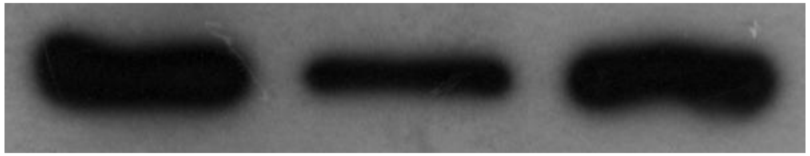

Supplement: S1 Raw images — (PDF) [file pone.0276328.s001.pdf]
