# Supplementary material for: Xianling Gubao attenuates high glucose-induced bone metabolism disorder in MG63 osteoblast-like cells
Source: PLoS One. 2022 Dec 22;17(12):e0276328. doi: 10.1371/journal.pone.0276328 (PMC9778583; doi:10.1371/journal.pone.0276328)
Supplement: Attachment — Submitted filename: Response to Reviewers.docx [file pone.0276328.s002.docx]

Dear Editor,

Thank you for reading and reviewing our manuscript. Those comments are all valuable and very helpful for revising and improving our paper. According to the editor and reviewers’ comments, we have made extensive modifications to our manuscript to make our results convincing, and we wish it to be considered for publication in your journal. The detailed point-by-point responses are listed below.

Reviewer #2: 1. I request authors to do western blot analysis for some of the osteogenesis differentiation-associated markers.

Response: Thank you very much for the comments. Thank you very much. We detected differentiation-associated markers ALP, Runx2 and OCN. The results were showed in Figure 7C.

2. The effect of XLGB on MG63 alone should be studied to conclude that XLGB speciffcally rescue the MG63 from High Glucose treatment from osteoblasts proliferation, migration, differentiation etc and inhibiting the osteoblast apoptosis.

Response: We detected the effect of XLGB on MG63 alone, and the results were showed in Figure 6.

Thank you very much for your reconsidering our revised manuscript for potential publication. According to the reviewer’s comments, we have improved the manuscript extensively. If there are any other modifications we could make, we would like very much to modify them and we really appreciate your help. We look forward to hearing from you regarding our submission. We would be glad to respond to any further questions and comments that you may have.

Sincerely yours

Xiaoqian Zhang
